# Molecular Epidemiology of Bacterial Wilt in the Madagascar Highlands Caused by Andean (Phylotype IIB-1) and African (Phylotype III) Brown Rot Strains of the *Ralstonia solanacearum* Species Complex

**DOI:** 10.3389/fpls.2017.02258

**Published:** 2018-01-15

**Authors:** Santatra Ravelomanantsoa, Christian Vernière, Adrien Rieux, Laurent Costet, Frédéric Chiroleu, Sandrine Arribat, Gilles Cellier, Olivier Pruvost, Stéphane Poussier, Isabelle Robène, Fabien Guérin, Philippe Prior

**Affiliations:** ^1^Unité Mixte de Recherche, Peuplements Végétaux et Bioagresseurs en Milieu Tropical, Centre de Coopération Internationale en Recherche Agronomique pour le Développement, Saint-Pierre, France; ^2^Unité Mixte de Recherche, Peuplements Végétaux et Bioagresseurs en Milieu Tropical, University of Réunion, Saint-Denis, France; ^3^Faculty of Sciences, University of Antananarivo, Antananarivo, Madagascar; ^4^Unité Mixte de Recherche, Biologie et Génétique des Interactions Plante-Parasite, Centre de Coopération Internationale en Recherche Agronomique pour le Développement, Montpellier, France; ^5^Tropical Pests and Diseases Unit, Plant Health Laboratory, Agence Nationale de Sécurité Sanitaire de l’Alimentation, de l’Environnement et du Travail, Saint-Pierre, France; ^6^Unité Mixte de Recherche, Peuplements Végétaux et Bioagresseurs en Milieu Tropical, Institut National de la Recherche Agronomique, Saint-Pierre, France

**Keywords:** *Ralstonia solanacearum*, genetic diversity, MLVA, population structure, Madagascar

## Abstract

The *Ralstonia solanacearum* species complex (RSSC) is a highly diverse cluster of bacterial strains found worldwide, many of which are destructive and cause bacterial wilt (BW) in a wide range of host plants. In 2009, potato production in Madagascar was dramatically affected by several BW epidemics. Controlling this disease is critical for Malagasy potato producers. The first important step toward control is the characterization of strains and their putative origins. The genetic diversity and population structure of the RSSC were investigated in the major potato production areas of the Highlands. A large collection of strains (*n* = 1224) was assigned to RSSC phylotypes based on multiplex polymerase chain reaction (PCR). Phylotypes I and III have been present in Madagascar for a long time but rarely associated with major potato BW outbreaks. The marked increase of BW prevalence was found associated with phylotype IIB sequevar 1 (IIB-1) strains (*n* = 879). This is the first report of phylotype IIB-1 strains in Madagascar. In addition to reference strains, epidemic IIB-1 strains (*n* = 255) were genotyped using the existing MultiLocus Variable-Number Tandem Repeat Analysis (MLVA) scheme RS2-MLVA9, producing 31 haplotypes separated into two related clonal complexes (CCs). One major CC included most of the worldwide haplotypes distributed across wide areas. A regional-scale investigation suggested that phylotype IIB-1 strains were introduced and massively spread via latently infected potato seed tubers. Additionally, the genetic structure of phylotype IIB-1 likely resulted from a bottleneck/founder effect. The population structure of phylotype III, described here for the first time in Madagascar, exhibited a different pattern. Phylotype III strains (*n* = 217) were genotyped using the highly discriminatory MLVA scheme RS3-MLVA16. High genetic diversity was uncovered, with 117 haplotypes grouped into 11 CCs. Malagasy phylotype III strains were highly differentiated from continental African strains, suggesting no recent migration from the continent. Overall, population structure of phylotype III involves individual small CCs that correlate to restricted geographic areas in Madagascar. The evidence suggests, if at all, that African phylotype III strains are not efficiently transmitted through latently infected potato seed tubers.

## Introduction

The soil-borne *Ralstonia solanacearum* species complex (RSSC) is among the plant pathogenic bacteria that are most highly destructive to crops (Mansfield et al., 2012), leading to significant economic losses for growers worldwide and resulting in dramatic consequences for sustainable crop production and food security. The RSSC is a group of pathogens that causes vascular wilt disease in almost 200 plant species (Hayward, 1994) and is hosted as a latent infection in an unusually broad range of 450 plant species in approximately 54 families, including food and high-value cash crops worldwide, such as potatoes, tomatoes, tobacco, ginger, and bananas as well as vegetables and ornamentals (Elphinstone, 2005). The RSSC comprises three species (Safni et al., 2014) and is classified into four major lineages according to their initial geographical origin (Prior and Fegan, 2005): *Ralstonia* *pseudosolanacearum* includes phylotype I from Asia and phylotype III from Africa, *R. solanacearum* includes phylotype II with IIA and IIB subdivisions from the Americas, and *Ralstonia syzygii* contains phylotype IV from Indonesia and likely Japan, the Philippines, Korea, and Australia (Poussier et al., 2000; Fegan and Prior, 2005; Villa et al., 2005; Jeong et al., 2007). The phylotypes are further subdivided into sequevars, each of which comprises great phenotypic diversity (Fegan and Prior, 2005; Wicker et al., 2012). RSSC strains have disparate lifestyles and exhibit pathogenic behavior, such as the ability to survive in heterogeneous niches and asymptomatic plants (Álvarez et al., 2010; CABI, 2016). Thus, in addition to the versatility and diversity of these strains, limited host resistance makes it difficult to manage the RSSC. Currently, there is no effective treatment against the RSSC. Hence, due to its social and economic impact, the RSSC has been extensively investigated to understand aspects of its biology and to engineer optimal and durable strategies to control this destructive plant pathogen (Mansfield et al., 2012).

In Madagascar, bacterial wilt (BW) caused by the RSSC was first reported in tobacco crops in 1936 (Bouriquet, 1946). Well-established and consistently present for many years in major market gardening areas in the Central Highlands and the Western and Eastern Coastal areas, this organism affects tomato, eggplant, tobacco, peanut, bean, and potato, generally at an acceptable level of impact (Rasolofo, 1965). Recently, the Central Highlands of Madagascar have experienced a spectacular potato BW epidemic, causing major damage to potato production. Since 2009, major outbreaks have been reported in the main basin where potato is produced, specifically in the Vakinankaratra region and have become increasingly widespread to other basins in the Central Highlands. These outbreaks are associated with meaningful changes to the BW epidemiological profile. Whereas BW previously occurred primarily during the rainy season on rain-fed potato crops, since 2009, there has been a dramatic increase in its incidence on irrigated potato crops cultivated in rice fields or volcanic soil where the disease was not previously noted (Rabakoarihanta and Rakotondramanana, 1984; Rakotondramanana, 1984; MAEP-UPDR, 2004). Previously, wilting symptoms were typically observed during tuber initiation to flowering; however, symptoms today are spotted very early during vegetative growth. For years, BW was controlled through the use of BW-tolerant resistant cultivars, specifically potato accessions from the International Potato Center (CIP) germplasm collection that were developed by the National Centre for Rural Development and Applied Research, FIFAMANOR. Approximately 10 BW-tolerant potato cultivars were disseminated to farmers. However, these have become susceptible to BW. The extent of the current epidemiological situation has not previously been observed.

Currently, potato BW in the Madagascar Highlands is a national pandemic that Malagasy authorities must control and manage. BW outbreaks are a major concern for stakeholders in agriculture, including potato growers; potato production has fallen by 30%, leading to huge economic losses and directly affecting food security and livelihoods in rural areas. The major, basic potato seed growing fields are infested by the RSSC and thus are unfit for the cultivation of other crops susceptible to BW, and the lack of availability of healthy potato seeds has worsened the situation because farmers use potato seed tubers that are not certified as RSSC-free. Thus, BW has the potential to majorly impact the Malagasy potato trade. To address BW disease, there is an urgent need to identify RSSC strain types that are prevalent in the Central Highlands of Madagascar, specifically those associated with potato BW and those responsible for the current BW epidemics. In addition, further assessment of the epidemiological factors underlying the persistence and emergence of RSSC strains and their spread in potato growing zones is needed.

Historically, strains collected in Madagascar, identified by Lallmahomed et al. (1988) as “Malagasy brown rot,” were assigned to phylotype III (Poussier et al., 2000), named “African brown rot” (Mahbou et al., 2009) and to phylotype I, both of which have been collected from the plains and tray areas first described by Prior et al. (2006, unpublished data). All phylotypes are composed of lineages pathogenic to potatoes, and there is pathological and genetic diversity within and among phylotypes (Cellier and Prior, 2010). BW epidemics result from the emergence of more aggressive strains of existing, indigenous bacteria or cryptic strains that have not yet been characterized or that would have recently jumped to a new host. In addition, epidemics are likely associated with the introduction and spread of aggressive exotic strains such as the regulated quarantine organism (EPPO, 2015) and the select agent (USDA/APHIS, 2005) phylotype IIB sequevar 1 (IIB-1) strains named “Andean brown rot,” to which cultivated varieties of potato may be more susceptible. Furthermore, insights into the major epidemiological traits of this disease, such as inoculum sources and reservoirs, patterns of dispersal and other factors that may influence the spatial dynamics of inoculum dispersal and disease spread, are prerequisites for the design of logical and effective management strategies that limit and control the spread of BW.

Molecular epidemiological approaches have been widely used for animal and human infectious disease and distribution studies (Tompkins, 1994; Foxman, 2010; Lindstedt et al., 2013). Using such molecular epidemiological approaches, clinicians have shed light on diseases and developed more effective prevention strategies compared to conventional epidemiological approaches (Foxman and Riley, 2001). Examples include the investigation of *Mycobacterium tuberculosis* (Kamerbeek et al., 1997; Barnes and Cave, 2003; Van der Spuy and Warren, 2008; Cannas et al., 2016; Jagielski et al., 2016), *Escherichia coli* (Bender et al., 1997; Johnson et al., 2002; Bonacorsi and Bingen, 2005; Grad et al., 2012), and *Influenza A virus* (Palese and Young, 1983; Cox and Bender, 1995; Cox and Subbarao, 2000). Molecular epidemiology has also been applied to plant pathology to investigate host–pathogen interactions, strain phylogenetic backgrounds, reservoirs, and transmission pathways to assess potential control strategies. A few approaches to describe plant bacteria epidemics have been successfully developed, particularly for *Xanthomonas citri* (Pruvost et al., 2014; Vernière et al., 2014; Leduc et al., 2015) and *Erwinia amylovora* (Bühlmann et al., 2014). With the development of microbial genome sequencing, genomic comparisons of pathogens have been conducted for rapid and accurate diagnosis and the characterization of markers suited for epidemiological analyses (Aarestrup et al., 2012; Land et al., 2015). Based on the amplification of specific and highly variable tandem repeat (TR) loci as markers, MultiLocus Variable-Number TR Analysis (MLVA) is a high-throughput genotyping tool that allows the exploration of TR loci polymorphisms between organisms at the intra or inter population level within species (Lindstedt, 2005). Different MLVA schemes based on combinations of variable TR loci were developed to genotype plant pathogenic bacteria of high agricultural significance, including *Xylella fastidiosa* (Coletta-Filho et al., 2001; Francisco et al., 2017), *X. citri* (Bui Thi Ngoc et al., 2009; Pruvost et al., 2014; Vernière et al., 2014), *Pseudomonas syringae* (Gironde and Manceau, 2012; Ciarroni et al., 2015), *Xanthomonas oryzae* (Zhao et al., 2012), *Clavibacter michiganensis* (Zaluga et al., 2013), *E. amylovora* (Bühlmann et al., 2014; Alnaasan et al., 2017), and *Xanthomonas arboricola* (López-Soriano et al., 2016). Several MLVA schemes for genotyping the RSSC have been published: N’Guessan et al. (2013) developed four schemes, specifically, a 13-loci MLVA scheme for phylotype I, a 12-loci MLVA scheme for phylotype II, an 11-loci MLVA scheme for phylotype III, and a six-loci MLVA scheme for phylotype IV. Ravelomanantsoa et al. (2016) published a novel 16-loci MLVA scheme called RS3-MLVA16 that is optimized for phylotype III, and Guinard et al. (2016) developed an seven-loci MLVA scheme to closely monitor the microevolution of phylotype I populations.

In this study, 1224 BW strains, which were collected from isolated wilted plants or minor outbreaks and from the major emerging epidemics observed on potato in the Madagascar Central Highlands, were assigned to RSSC phylotypes by multiplex PCR. The population structure of a subset of phylotype IIB-1 and III strains was studied using previously developed MLVA markers (N’Guessan et al., 2013; Parkinson et al., 2013; Ravelomanantsoa et al., 2016). We showed that the cold-tolerant potato Andean brown rot phylotype IIB-1 was clearly introduced to Madagascar, provoking a major epidemic. Phylotype IIB-1 was previously recognized to be clonal with same epidemiological traits (Graham and Lloyd, 1979; Janse, 1996, 2012; Wenneker et al., 1999; Pradhanang et al., 2000; Allen et al., 2005; Álvarez et al., 2008; Milling et al., 2009; Khoodoo et al., 2010; Clarke et al., 2015; Huerta et al., 2015). Here, we highlighted a small amount of genetic diversity and described the genetic structure of phylotype IIB-1. Additionally, we were interested in African brown rot (*R. pseudosolanacearum* phylotype III) strains whose epidemiological traits are unknown but are of agronomic importance and endemic to Africa and the South-West Indian Ocean islands (Fegan and Prior, 2005). Although both phylotypes have adapted to cool temperatures, molecular epidemiology clearly suggested two different epidemiological situations associated with these two phylotypes.

## Materials and Methods

### Collection of RSSC Strains

RSSC strains were collected in major potato production areas in the temperate Central Highlands. Samples were collected from fields historically associated with frequent BW infections and sites associated with outbreaks that recently occurred within 12 agroecological zones (AEZs). Sampling sites with potato BW lasting for two main growing seasons and with major symptom development were chosen: the season with rain-fed and irrigated crops on hillsides in April 2013 and the rainy season for rain-fed crops on upland soils in December 2013. Foliar epinasty or plant wilting characterized symptomatic plants. In an attempt to reflect the entire infected plant population in each surveyed field, approximately 20–30 georeferenced stem samples, approximately 10 cm in length and above the root collars of symptomatic hosts were randomly collected. To explore RSSC diversity in potato growing areas, stems from putative symptomatic and asymptomatic host species, namely *Solanum lycopersicum* (tomato), *Solanum gilo* (African eggplant), *Solanum nigrum* (black nightshade), *Phaseolus vulgaris* (bean), *Bidens pilosa* (weed), *Capsicum annuum* (pepper), *Pelargonium* sp., *Physalis* sp. (cape gooseberry), *Eucalyptus* sp., and *Manihot esculenta* (cassava) were sampled, as were rhizosphere and irrigation water. All samples were cut with disinfected shears (wiped with 90% alcohol), placed in a labeled plastic bag and stored in a cool box for shipment to the local laboratory. Additional surveys were conducted to address issues of particular interest, primarily focusing on the history of BW at the field and local levels, the origin and trade of potato seeds and cropping pathways and practices.

At the lab, the stem samples were cleaned, dried, and surface decontaminated by wiping them with 90% alcohol and briefly passing them through a flame. Then, the samples were cut into pieces using a sterilized scalpel, placed into tubes, and immersed in pure sterile water for 15 min to recover the bacterial ooze. Five microliters of the bacterial suspension were streaked for isolation on Kelman’s agar plates (Kelman, 1954) and incubated at room temperature (approximately 28°C) for 2 days. Single virulent colonies of RSSC, irregular in shape and pearly white with pink centers, were suspended in pure sterile water in Eppendorf tubes. At the CIRAD-3P laboratory (Saint-Pierre, La Réunion), the bacterial suspensions were cultured in nutrient broth at 28°C for 2 days for culture purification. Then, bacterial cultures were streaked both on Kelman and modified Sequeira agar plates (Poussier et al., 1999) and incubated at 28°C for 2 days. Single colonies were transferred to Sequeira plates and subsequently purified. The pure colonies for each isolate underwent long-term storage at -80°C on Microbank beads (Pro-Lab Diagnostics, Toronto, Canada).

### Strain Phylotyping

A first subset of isolates (CSEQ; *n* = 289) representative of the geographic regions (agricultural zones), agroecological environments (lowland and highlands), known soil types (acidic to calcareous, clay to loam soils), and host diversity (species and potato cultivars) from the 12 surveyed AEZs was selected from the whole collection for sequevar identification. A second subset of phylotypes IIB-1 (*n* = 255) and III (*n* = 217) isolates primarily sampled from potato in Madagascar (CMG) were submitted to MLVA analysis. Because only a limited number of phylotype I strains originated from potato (*n* = 20), this lineage was not further considered for MLVA analysis. A third set of strains called CREF included worldwide reference strains maintained in the RUN collection at CIRAD (Saint-Pierre, La Réunion): CREF-I (*n* = 42), CREF-II (*n* = 30), CREF-III (*n* = 65), and CREF-IV (*n* = 9), referred to as phylotypes I, II, III, and IV in the reference strain collection, respectively. The selected strains were representative of known genotypic diversity, geographic origins, years of isolation and hosts (**Supplementary Table S1**).

Single colonies from a Kelman agar plate were transferred to a modified Sequeira agar plate, and 1-μl loops of colonies grown overnight were suspended in 200 μl of sterile HPLC-grade water and used as templates for PCR amplification. The amplification of the 282-bp universal RSSC-specific DNA fragment and the phylotype-specific 16S–23S ITS DNA fragments (Opina et al., 1997), the 144-bp phylotype I-specific fragment, the 372-bp phylotype II-specific fragment, the 91-bp phylotype III-specific fragment, and the 213-bp phylotype IV-specific fragment, was carried out by phylotype multiplex PCR using a set of six oligonucleotide primers: 759F (5′-GTCGCCGTCAACTCACTTTCC-3′), 760R (5′-GTCGCCGTCAGCAATGCGGAATCG-3′) (Opina et al., 1997); Nmult 21:1F (5′-CGTTGATGAGGCGCGCAATTT-3′), Nmult 21:2F (5′-AAGTTATGGACGGTGGAAGTC-3′); Nmult 23-AF (5′-ATTACGAGAGCAATCGAAAGATT-3′), Nmult 22-InF (5′-ATTGCCAAGACGAGAGAAGTA-3′), and the reverse primer Nmult 22-RR (5′-TCGCTTGACCCTATAACGAGTA-3′) (Fegan and Prior, 2005). PCR amplification was performed in 15-μl reaction volumes with 3 μl of GoTaq Flexi 5× GREEN reaction buffer (Promega), 0.9 μl of MgCl_2_ (25 mM, Promega), 0.3 μl of dNTPs mixture solution (10 mM each, Promega), 1.5 μl of a mix of forward and reverse primers (10×), 0.125 μl of GoTaq^®^ G2 Flexi DNA polymerase solution (5 U/μl, Promega), 1 μl of fresh bacterial suspension, and 15 μl of QSP with sterile HPLC water. The 10× primer mix was composed of 759R and 760F at 4 pmol each; Nmult 21:1F, Nmult 21:2F, Nmult 22-InF, and Nmult 22-RR at 6 pmol each; and Nmult23-AF at 18 pmol. The reaction was performed in a GeneAmp^®^ PCR System 9700 thermal cycler (Applied Biosystems, Foster City, CA, United States) using the following conditions: an initial denaturation step at 96°C for 5 min, 30 cycles of denaturation at 94°C for 15 s, annealing at 59°C for 30 s, and extension at 72°C for 30 s, and a final extension step at 72°C for 10 min. Six microliters of PCR product were loaded into a 1.5% (w/v) SeaKem^®^ LE Agarose gel (Lonza, Basel, Switzerland) in 1× TAE buffer at 110 V for 1 h. After migration, the gels were stained in an ethidium bromide bath and photographed under ultraviolet light using the G-BOX gel imaging system (Syngene, Cambridge, United Kingdom). A 100-bp DNA ladder (Promega, Madison, WI, United States) was used to estimate the molecular size of each amplicon.

Phylotype II strains were further processed to detect IIB-1 brown rot strains using the primer pair 630F/631R (5′-ATACAGAATTCGACCGGCACG-3′ and 5′-AATCACATGCAATTCGCCTACG-3′, respectively), which amplified a 307-bp IIB-1-specific DNA fragment (Fegan et al., 1998). The PCR reaction was carried out in a 15-μl volume, as previously described, with 0.36 μl each of the primers 630 and 631 (10 μM each) and 8.96 μl of sterile HPLC water. The following PCR program was used: initial denaturation at 96°C for 6 min, followed by 30 cycles of 94°C for 15 s, 60°C for 30 s, and 72°C for 30 s, with a final extension step of 72°C for 10 min.

Each phylotype was subdivided into sequevars based on polymorphism of the partial endoglucanase (*egl*) sequence (Fegan and Prior, 2005). Approximately 780-bp was amplified using the primer pair EndoF/EndoR (5′-ATGCATGCCGCTGGTCGCCGC-3′ and 5′-GCGTTGCCCGGCACGAACACC-3′, respectively) (Fegan and Prior, 2005). The PCR protocol was described previously by Ravelomanantsoa et al. (2016). PCR products were sequenced on both strands by Beckman Coulter Genomics Company (Takeley, United Kingdom). All sequences were further aligned using the MUSCLE algorithm (Edgar, 2004) and analyzed using Molecular Evolutionary Genetics Analysis (MEGA 7.0.18) software (Kumar et al., 2016). The sequences were compared to known *egl* sequences in a database to identify the sequevars to which Malagasy strains were assigned. Sequence homology was at least 99%. The database *egl* sequences used for comparison in this study were retrieved from GenBank. The accession numbers of these sequences are listed in **Supplementary Table S2**.

### MLVA Analysis

MLVA was performed as described previously (Ravelomanantsoa et al., 2016). Briefly, MLVA exploits length variations of TR arrays that occur at different TR loci. An allelic profile was created for each strain based on the number of repeats in a set of TR loci (alleles) combined into a string. Each unique profile (or haplotype) was assigned an MLVA type (MT), which was were grouped into a clonal complex (CC). The previously described RS3-MLVA16 scheme (Ravelomanantsoa et al., 2016), was applied to investigate the genetic relationships between phylotype III strains (RS3-MLVA16: RS3L27, RS3L28, RS3L29, RS3L17, RS3L19, RS3L30, RS3L31, RS3L32, RS3L33, RS3L34, RS1L05, RS3L35, RS3L36, RS1L10, RS3L37, and RS1L12). In addition, we selected nine highly polymorphic TR loci from the literature to genotype phylotype IIB-1 strains, specifically five TR loci (RS2AL01, RS2BL21, RS2BL22, RS2BL24, and RS2BL25) from the 12-locus MLVA scheme proposed by N’Guessan et al. (2013), plus four TR loci (L504, L539, L540, and L563) published by Parkinson et al. (2013), to form the MLVA scheme RS2-MLVA9. Characteristics of the TR loci used in this study are presented in **Supplementary Table S3**.

### Population Structure

Analyses were performed with the R v 3.0.2 software package (R Core Team, 2013). The software QGIS V2.0.1-Dufour (QGIS Development Team, 2013) was used to map the geographical distribution of the sampling locations and the corresponding phylotypes. Genetic variation was estimated at three levels: within phylotypes, within populations, and among populations. The populations were arbitrarily divided into AEZs. We calculated the standard measures of genetic diversity for each phylotype and population, including the number of alleles (Na), the proportion of polymorphic loci (P), the number of haplotypes (Hap), and the haplotype diversity (H_E_), using the GenAlEx version 6.5 program (Peakall and Smouse, 2012). The allelic richness by rarefaction (A) (El Mousadik and Petit, 1996) was also estimated using the function “*allelic.richness*” in the “*hierfstat*” package (Goudet, 2014). Clustering of the allelic profiles was performed with PHYLOVIZ software (Francisco et al., 2012), and minimum spanning trees (MST) were built with the goeBURST full MST algorithm using global optimal eBURST (goeBURST) and Euclidean distances. The discriminatory power of the MLVA scheme was evaluated by calculating the Hunter–Gaston index^1^ (HGDI; Hunter and Gaston, 1988; Hunter, 1990). Genetic divergence among populations and among individuals within populations were calculated by analyzing the molecular variance (AMOVA) and calculating the differentiation indices Fst (Nei, 1977) and Rst (Slatkin, 1995). Because TRs are subdivided into microsatellites (1–6 bp repeat units, Goldstein and Schlotterer, 1999) and mini-satellites (more than 6 bp in length) do not evolve at a uniform rate and follow different mutation models (Shriver et al., 1993; Estoup and Angers, 1998), the two indices Fst and Rst were calculated together to test the importance of allele size over allele identity in the estimation of genetic differentiation. AMOVA, Fst and Rst estimates were computed using ARLEQUIN version 3.5 software (Excoffier and Lischer, 2010).

A Bayesian clustering approach implemented in GENELAND software (Guillot et al., 2005, 2008) was applied to spatially delineate and infer the genetic structure of the RSSC within the potato growing areas in the Central Highlands without prior information regarding clusters. The number cluster (K) and the individual posterior cluster membership probabilities were computed assuming a correlated allele frequencies model on the one hand and an uncorrelated model without spatial coordinates on the other (spatially implicit model). We ran 10 independent runs, in which we allowed K to vary from 1 to 20, and the number of MCMC iterations (Markov Chain Monte-Carlo inference of clusters from genotype data) was 200,000 with a thinning of 100. The maximum rate of Poisson processes was set to 100. For each run, the most likely number of clusters was automatically displayed, and the runs with the highest likelihood scores were kept. The posterior probability of cluster membership was computed for each pixel of the spatial domain (500 × 500 pixels). Individuals were assigned unambiguously to the modal cluster when the posterior probability of cluster membership was greater than 0.70.

We estimated genetic diversity within each of the inferred clusters by calculating Na, P, Hap, H_E_, and A. To determine the levels of differentiation within and among the inferred genetic clusters, AMOVA and pairwise Fst and Rst values were calculated using ARLEQUIN version 3.5 software. Survey data were compared with molecular data to better explain BW disease.

## Results

### Three Phylotypes and Eight Sequevars among the RSSC Strains in the Central Highlands of Madagascar

**FIGURE 1:**
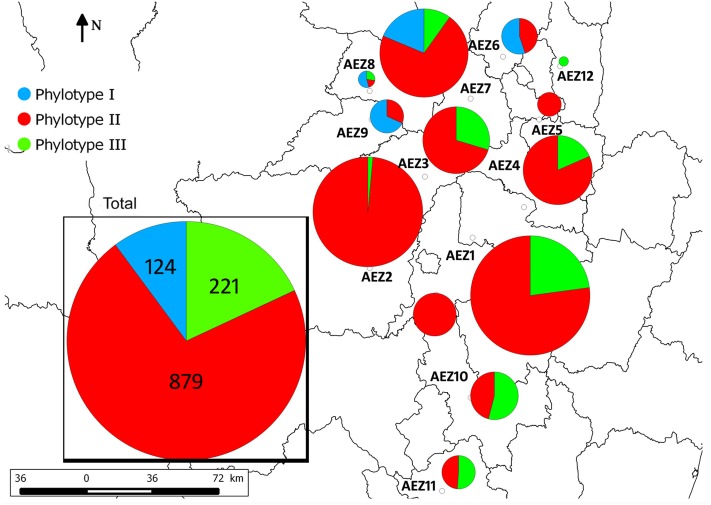
Spatial distribution and frequency of the phylotypes I, II, and III across the 12 AEZs in the Central Highlands of Madagascar.

A total of 1224 isolates were collected from 74 sites across 12 AEZs in predominantly potato growing areas. They were isolated from the rhizosphere soils of potatoes, irrigation water, and from various symptomatic plant species, including *Solanum tuberosum* (potato), *S. lycopersicum* (tomato), *S. gilo* (African eggplant), *P. vulgaris* (bean), *B. pilosa* (weed), *S. nigrum* (black nightshade), *C. annuum* (pepper), *Pelargonium* sp., and symptomless *Physalis* sp. (cape gooseberry). One hundred twenty-four (10%) isolates belonged to phylotype I and were located on the northwestern side of the Central Highlands at an altitude of approximately 1000 to 1600 masl. Two hundred twenty-one (18%) isolates belonged to phylotype III and the remainder (*n* = 879, approximately 72%) belonged to phylotype IIB1 (IIB-1); both were located in most prospected AEZs at an altitude between 1000 to 2000 m. This is the first official report of Andean potato brown rot IIB-1 in Madagascar. The spatial distribution of the three phylotypes across the 12 AEZs is reported **Figure 1**.

Partial *egl* sequences were obtained from a set of 289 representative strains, CSEQ, including 70 strains from phylotype I, 77 strains from phylotype II, and 142 strains from phylotype III (**Supplementary Table S1**). Seven sequevars were identified based on phylogenetic analysis with existing sequences in databases. Among the seven sequevars, sequevar 1 (belonging to phylotype IIB), 18 and 33 (belonging to phylotype I), and 19 (belonging to phylotype III) have been previously identified. The three remaining sequevars are newly identified and were defined as sequevars 58, 59, and 60 (belonging to phylotype III). The known sequevar 46 belonging to phylotype I, previously identified by Prior et al. (2006, unpublished data) from the Malagasy lowland area (27 m), was not recovered in this Highland study. Taken together, seven distinct sequevars have been found to date in the Central Highlands of Madagascar. The newly generated sequences were deposited in the GenBank database under accession numbers MF134833 to MF134835.

### Genetic Diversity Based on MLVA Was Narrow among Introduced Andean Brown Rot Strains (IIB-1) and Broad among Endemic African Brown Rot Strains (III).

MLVA scheme RS2-MLVA9 was applied to 285 IIB-1 strains, including 30 reference strains (CREF-II) and 255 Malagasy representative strains (CMG-II). Phylotype IIB-1 was resolved into 47 haplotypes (P = 94%, A = 1.73, H_E_ = 0.20, HGDI = 0.80): 19 haplotypes were assigned to strains of the CREF-II collection (P = 88%, A = 1.66, H_E_ = 0.24, HGDI = 0.89), and 31 haplotypes were assigned to strains of CMG-II (P = 100%, A = 2.12, H_E_ = 0.19, HGDI = 0.78). MLVA scheme RS3-MLVA16 was used to examine the genetic diversity of 280 phylotype III strains, including a worldwide collection of 63 references (CREF-III) and a collection of 217 Malagasy representative strains (CMG-III). Phylotype III had 163 haplotypes (P = 100%, A = 11, H_E_ = 0.50, HGDI = 0.99): 46 haplotypes in the collection CREF-III (P = 100%, A = 4.88, H_E_ = 0.61, HGDI = 0.95) and 117 haplotypes (P = 81%, A = 2.96, H_E_ = 0.40, HGDI = 0.98) in the collection CMG-III. Globally, genetic diversity among the phylotype III strains (H_E_ ≥ 0.40, A < 11) was higher than phylotype IIB-1 (H_E_ ≤ 0.24, A < 2.12).

### RS2-MLVA9 Clustering Analysis Defined a Closely Related Genetic Group

**FIGURE 2:**
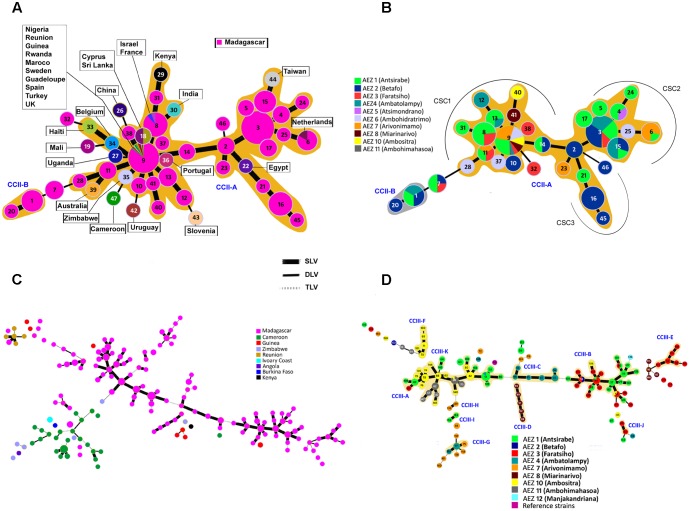
Minimum spanning tree (MST) representation of the MLVA clustering. **(A)** MST of global RSSC phylotype IIB-1 strains (*n* = 285). **(B)** MST of Malagasy RSSC phylotype IIB-1 strains collected in the Central Highlands of Madagascar (*n* = 255). **(C)** MST of African RSSC phylotype III strains (*n* = 280). **(D)** MST of Malagasy RSSC phylotype III strains collected in the Central Highlands of Madagascar (*n* = 217). Each circle represents a unique MLVA haplotype (MT) and its size is proportional to the number of strains having the same MT. The thicker branches link MT differing by only one TR locus (SLV), the thinner branches link MT that differ in 2 TR loci (DLV), and the gray dashed branches link MT that differ in three TR loci. No link is indicative of variation at >4 loci. Color represents the country or the agroecological zone of origin. Halos indicate the distinct clonal complexes (CCs). CSC, clonal sub-complex.

To study the genetic relationships between phylotype IIB-1 haplotypes isolated in the Central Highlands of Madagascar and worldwide haplotypes, a MST based on 47 haplotypes of 285 IIB-1 strains (MST-II_47_) was constructed (**Figure 2**). Strains from Madagascar clearly yielded a major CC (CCII-A), a minor CC (CCII-B) and three singleton haplotypes (different from each MT at more than one TR locus) (**Figure 2B**). Globally these IIB-1 haplotypes were closely related as all of the links between the haplotypes consisted of single or double-locus variations (DLVs). The largest clonal complex, CC-IIA, grouped together haplotypes from the Central Highlands of Madagascar and haplotypes widely distributed throughout the world. Three haplotypes isolated in the Malagasy potato growing areas were shared with strains from other countries (**Figure 2A**). The frequent haplotype MTII-9 was present in some African countries (Nigeria, Guinea, Rwanda), in Europe (Sweden, Spain, and the United Kingdom), in the Mediterranean (Morocco and Turkey), in Guadeloupe (Caribbean), and in the Reunion (Indian Ocean) Islands. Haplotype MTII-8 was present in Israel and France, and haplotype MTII-6 was present in the Netherlands. These results provided strong support for the hypothesis that multiple phylotype IIB-1 strains were introduced, as the IIB-1 brown rot pathogen has never previously been reported in Madagascar. In the Central Highlands of Madagascar, the grouping of a large proportion of phylotype IIB-1 strains into a single complex (MST-II_31_) with a few additional DLVs suggests phylotype IIB-1 strains are epidemiologically linked. A limited number of evolutionary links were missing to structure the whole population as a single CC, likely as a result of incomplete sampling. The star-like structure of the haplotype network where nearly all of the evolutionary steps could be drawn, and the presence of numerous shared haplotypes between strains from different AEZs supports the emergence of recent potato BW-IIB-1 in this region from a few closely related strains as well as their relatively fast geographical expansion across various AEZs (**Figure 2B**). goeBURST identified haplotypes MTII-3 and MTII-9 as the most likely founder haplotypes for the two different sampling dates, April and December, respectively. The structure of the large clonal complex CC-IIA showed three clonal sub-complexes (CSC). The two major clonal sub-complexes CSC1 and CSC2, the founders of which are MTII-9 and MTII-3, respectively, are connected through MTII-2, the predicted founder of the clonal sub-complex CSC3. At the field scale, haplotype diversity showed the coexistence of multiple CCs that differed by two loci (DLV) and singleton haplotypes with DLV from each other.

### MLVA16 Genotyping of Phylotype III Strains Separated Epidemiologically Unrelated Groups

In contrast to phylotype IIB-1, the MST based on 163 haplotypes from 280 phylotype III strains (MST-III_163_) resulted in 14 distinct CCs, most of them separated by more than triple-locus variants (TLVs). All of the delineated CCs consisted of strains from a single country with a single exception, which was composed of a haplotype from Madagascar (MTIII-72) and a haplotype from Reunion Island (MTIII-9) distinguishable at a single locus (**Figure 2C**). In the CMG-III collection, which was made up of 217 Malagasy representative phylotype III strains, 11 CCs (mostly separated by TLVs) and 37 singletons were found among the AEZ strains. Interestingly, the two largest CCs were mainly composed of strains from AEZ 1, 3, 10, and 11 and from AEZ 1, 3, and 10 for CCIII-A and CCIII-B, respectively (**Figure 2D**), which could suggest a common origin or migration events between strains of AEZ 1, 3, and 10. Eight of these CCs have a unique AEZ origin and may reflect local outbreaks. Strains from the same AEZ can be dispersed over different CCs and singletons. These epidemiologically unrelated haplotypes suggest different origins of introduction in these AEZ and/or a long-time of divergence within an AEZ population. Further analysis at the field scale showed a significant proportion of singleton haplotypes related to each other by more than four locus variations, indicating a high within-field genetic diversity of the phylotype III strain population, and could reflect an inoculum of multiple origins and/or differentiation among the field strains over time (**Supplementary Table S1**).

### Contrast between the Spatial Genetic Structures of Phylotype IIB-1 and III Populations in the Central Highlands of Madagascar

**FIGURE 3:**
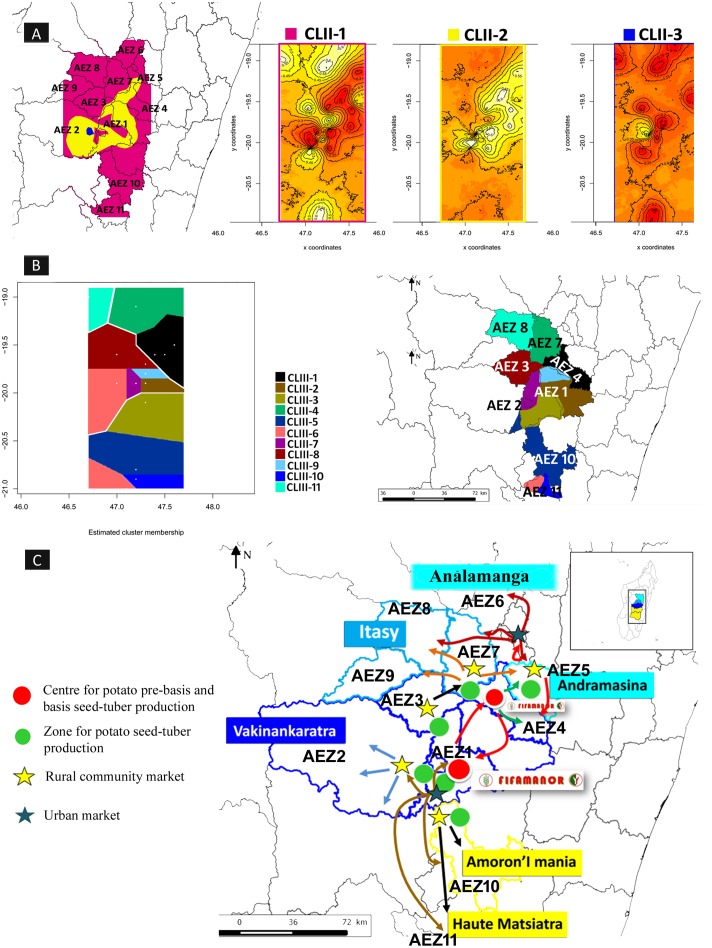
GENELAND analysis of RSSC populations in Central Highlands of Madagascar. **(A)** Spatially explicit estimate of population clusters, and the distribution of each phylotype IIB-1 genetic cluster defined by GENELAND at *K* = 3 from 70% posterior probability assuming uncorrelated allele frequency model (*n* = 255). The highest membership values are in light yellow, and the curves indicate spatial changes in assignment values. **(B)** Spatial distribution and population structure of the RSSC phylotype III strains inferred in GENELAND analyses at *K* = 11 from 70% posterior probability assuming correlated allele frequency (*n* = 217). The black dots indicate the sampling locations. The plot is based on the highest probability run for a given value of *K*. The abscissa and ordinate show the coordinates of sampling locations. **(C)** General flow of potato seed system in the potato growing basins.

**FIGURE 4:**
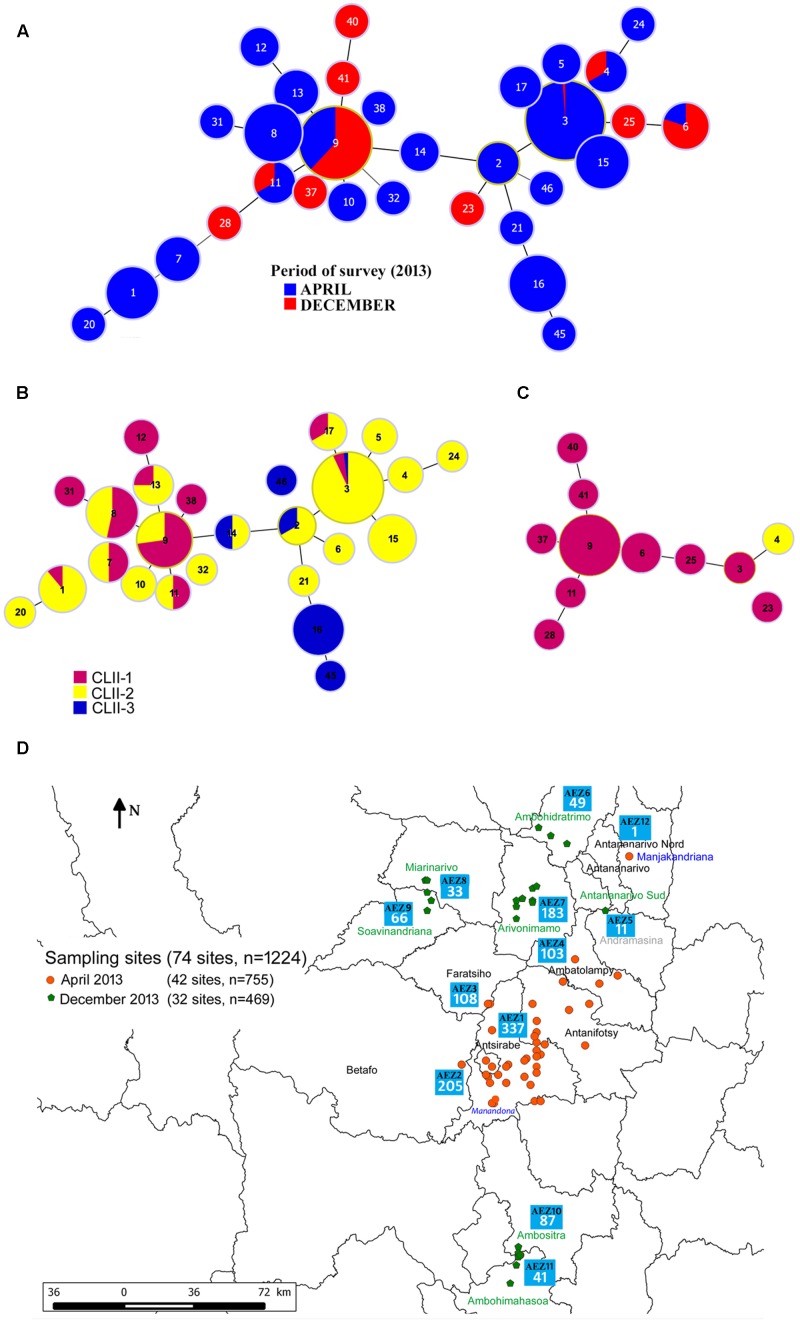
Minimum spanning tree (MST) representation of RSSC phylotype IIB-1 strains during the survey period. **(A)** MST of 255 phylotype IIB-1 strains. **(B)** MST of 206 phylotype IIB-1 strains collected in April 2013. **(C)** MST of 49 phylotype IIB-1 strains collected in December 2013. Each circle represents a unique MLVA haplotype (MT), and its size is proportional to the number of strains with the same MT. Color represents the population clusters computed in GENELAND. **(D)** Distribution of the sampling sites during the two survey sampling periods, April and December 2013.

Using the RS2-MLVA9 data, a MCMC analysis of the population structure using GENELAND indicated that the genetic variation across the CMG-II collection was best represented by three distinct clusters (*K* = 3) from a 70% posterior probability assuming an uncorrelated allele frequency model as the best-fit model for the data. The three inferred genetic clusters, CLII-1 (87 strains, H_E_ = 0.12, 18 MTs, eight polymorphic loci), CLII-2 (148 strains, H_E_ = 0.13, 19 MTs, eight polymorphic loci), and CLII-3 (20 strains, H_E_ = 0.12, six MTs, five polymorphic loci), displayed a low level of genetic diversity. The spatial structure (**Figure 3A**) appeared to be consistent with topographic features characterized by the presence of the Ankaratra Mountain range that extend from north to south in the Central Highlands (**Supplementary Figure S1**). The cluster CLII-1 was localized to AEZs around the Ankaratra Mountains (approximately 1100–1700 m), whereas clusters CLII-2 and CLII-3 were distributed in the Highlands above 1700 m. The clusters were genetically differentiated from one another with an average Fst = 0.47 (*p* < 0.001) and Rst = 0.40 (*p* < 0.001) and AMOVA partitioned 48% (Fst)/41% (Rst) of the total genetic variation within a cluster and 52% (Fst)/59% (Rst) among clusters. The spatial organization of the two clusters CLII-1 and CLII-2 demonstrated the characteristics of populations that had undergone a large expansion across all surveyed AEZs. The cluster CLII-1 covered a huge geographic area reaching from the northwestern portion of the Central Highlands in the Itasy basin (AEZ6, AEZ7, AEZ8), throughout the Vakinankaratra basin (AEZ1, AEZ3, AEZ4), and the southern portion (AEZ10, AEZ11). The cluster CLII-2 occurred in the two major producing basins, Vakinankaratra and Ambohimiadana (AEZ1, AEZ2, AEZ3, AEZ4, and AEZ5), while the third cluster CLII-3 was limited to only one AEZ (AEZ2) (**Figure 3A**). Furthermore, the clusters CLII-1 and CLII-2 occurred and overlapped in AEZ1, AEZ2, and AEZ3, while the clusters CLII-2 and CLII-3 co-occurred in AEZ2. Overall, there were consistencies in the patterns of spatial structure in IIB-1 with GENELAND and the genetic structure in the MSTs (April and December) (**Figures 4B,C**).

Using the RS3-MLVA16 data, GENELAND inferred 11 distinct clusters (*K* = 11) from the CMG-III collection, assuming a correlated allele frequency that was the most appropriate fit for the data. The 11 inferred genetic clusters were significantly genetically differentiated with a global Fst = 0.26 (*p* < 0.001) and Rst = 0.46 (*p* < 0.001): CLIII-1 (18 strains, H_E_ = 0.32, 10 MTs, nine polymorphic loci), CLIII-2 (16 strains, H_E_ = 0.21, seven MTs, eight polymorphic loci), CLIII-3 (seven strains, H_E_ = 0.36, five MTs, 10 polymorphic loci), CLIII-4 (18 strains, H_E_ = 0.41, 14 MTs, 10 polymorphic loci), CLIII-5 (49 strains, H_E_ = 0.30, 24 MTs, 12 polymorphic loci), CLIII-6 (two strains, H_E_ = 0.13, two MTs, two polymorphic loci), CLIII-7 (28 strains, H_E_ = 0.39, 14 MTs, 12 polymorphic loci), CLIII-8 (34 strains, H_E_ = 0.38, 27 MTs, 13 polymorphic loci), CLIII-9 (15 strains, H_E_ = 0.26, nine MTs, 10 polymorphic loci), CLIII-10 (19 strains, H_E_ = 0.07, six MTs, three polymorphic loci), and CLIII-11 (nine strains, H_E_ = 0.41, nine MTs, 11 polymorphic loci). Based on AMOVA, most of the genetic variation in the CMG-III collection was observed within clusters (Fst = 0.74, Rst = 0.56). Spatial organization of the 11 genetic clusters revealed a highly consistent geographic structure, as shown in **Figure 3B**. AEZ1 was infested by clusters CLIII-2, CLIII-3, CLIII-7, and CLIII-9, and AEZ11 contained clusters CLIII-6 and CLIII-10, which were each located in a well-delimited geographic area. The clusters CLIII-1, CLIII-4, CLIII-8, and CLIII-11 occurred in AEZ4, AEZ7, AEZ3, and AEZ8, respectively. Finally, cluster CLIII-5 occurred in AEZ2 and AEZ10.

## Discussion

The genetic diversity and population structure of the RSSC strains prevalent in Malagasy potato growing basins were investigated, and our understanding of potato BW epidemiology was improved. Most studies have investigated the population diversity and biology of plant pathogens to gain better insight into disease etiology (Milgroom and Peever, 2003). Major agronomic areas of the potato growing basins of Madagascar were prospected for RSSC strains at different agroecological scales and strains were typed using MLVA schemes. This article represents the first report on the molecular epidemiology of the phylotype III and is one of the first to use a molecular genotyping method to discriminate phylotype IIB-1 strains. Phylotypes IIB-1 (*R. solanacearum*) and III (*R. pseudosolanacearum*) are two distinct phylogenetic groups with an average nucleotide identity value of 91.3% (Remenant et al., 2010). Our aim was that genotyping be based on techniques offering a maximal typeability (a multilocus TR genotype can be produced for all assayed strains). This precluded using some loci that had been reported by N’Guessan et al. (2013) as present in both phylogenetic groups. Therefore, two distinct genotyping schemes, RS2-MLVA9 (nine loci) and RS3-MLVA16 (16 loci) were used. Variable TR loci are sufficiently reliable to perform fine-scale genotyping despite the risk of homoplasy (Lindstedt, 2005; Lindstedt et al., 2013; Struelens and Brisse, 2013). Combining multiple TR loci in each MLVA scheme allowed increasing their discriminative power and decreasing the effects of homoplasy (Estoup et al., 2002; Reyes et al., 2012). In this study, RS2-MLVA9 showed a good ability at differentiating monomorphic-like phylotype IIB-1. In addition, epidemiological relevance of the two MLVA datasets was shown. Our analyses provide additional knowledge regarding the occurrence of BW disease in the potato growing areas, especially haplotypic diversity of the pathogen, population structure, inoculum sources, putative dispersal patterns, and disease development in the Central Highlands of Madagascar.

### Multiple Phylotypes Coexist in a Single AEZ, a Single Locality, and a Single Field

Phylotypes I, II, and III co-occurred throughout the potato growing basins in the Malagasy Central Highlands. A similar picture of co-occurrence in a single region has been reported in other countries, for example in Cameroon, the southeastern United States and Guatemala (Sanchez Perez et al., 2008; Mahbou et al., 2009; Hong et al., 2012). The three phylotypes co-occurred in single AEZs, such as in AEZ7 as well as in AEZ8, and further co-existed at a single site, as shown at site S50 located in AEZ7 (**Supplementary Table S1**). In addition, co-occurring phylotypes I and II was detected from potato and tomato crops, each located in a single field, as shown in AEZ6 and AEZ7. The combination of phylotypes II and III was also found primarily on potato crops as well as on beans and in water irrigation, such as in AEZ1, AEZ2, AEZ3, AEZ4, AEZ8, AEZ10, and AEZ11. Finally, phylotypes II and III were typically found on peppers cropped in the same field in AEZ8. These observations corroborate the findings of Huerta et al. (2015) regarding the coexistence of different phylotypes in cropping areas.

Andean brown rot IIB-1 strains grow better at cool temperatures (Ciampi et al., 1980; Milling et al., 2009), resulting in a high frequency in the Highlands at 1000–2000 m elevation. However, the rationale for the phylotype I distribution only on the northwestern side of the Central Highlands has yet to be explained because there is no clear-cut evidence suggesting an underlying role for elevation in the effects of temperature on fitness and their geographical distribution. However, questions have been raised regarding the competitive fitness of the phylotype III strains in cropping areas and *in planta* when they coexist with other phylotypes. Additionally, the effects of temperature on the ability of these strains to co-occur and to cause disease must be addressed.

### African Brown Rot Strain Phylotype III Is Not Epidemiologically Active

The RSSC phylotype III population showed a high genetic diversity, suggesting that these strains have probably been present for a long time in Madagascar. This phylotype is genetically structured into 11 distinct clusters that clearly correlated to the geographic location. Supporting evidence was deduced from the MST and GENELAND structuring analyses (**Figure 3B**). In regards to potato supply and trade routes, the geographic structure found in the Malagasy phylotype III populations was not related to any physical barriers in the potato growing basins, as is typically found in most cases (Manel et al., 2003; Frantz et al., 2009; Balkenhol et al., 2015). Because potato tubers can be major carriers of RSSC strains, genetically and geographically separated phylotype III clusters are consistent with the lack of potato tuber inoculum dispersal. We hypothesize that this is due to the inability of this phylotype to be latently transmitted in potato tubers. This hypothesis will be tested by screening for the presence/absence of phylotype III in asymptomatic tubers from infected plants.

Some shared haplotypes were only isolated from *S. tuberosum* (including haplotypes MTIII-55, MTIII-56, MTIII-58, and MTIII-60), and others (MTIII-51 and MTIII-68) were found in various species, e.g., haplotypes MTIII-61, MTIII-62, MTIII-64, MTIII-69, and MTIII-82. The close genetic link between haplotypes found in distant AEZs that clustered in a same CC (for example, between haplotypes MTIII-78, MTIII-49, and MTIII-107 or between haplotypes MTIII-51, MTIII-82, and MTIII-58 in CCIII-A) suggests a dispersal likely promoted by infected plant exchanges. Phylotype III strains were indeed isolated from various solanaceous species (*C. annuum*, *Physalis* sp., *S. lycopersicum*, *S. nigrum*), from weeds (*B. pilosa*) and other species (*Pelargonium* sp.), which can be considered as potential sources of inoculum or reservoirs in the field. This wide host range could likely contribute to the survival and evolution potential of the phylotype III. Isolation of phylotype III from shared water irrigation indicates the latter as a vehicle for transmitting inoculum from one field to another. There have been no published reports that formerly exclude the transmission of phylotype III strains by latently infected potato tubers; and latent infection by phylotype III strains in potato tubers has yet to be analyzed. This information is crucial for disease management.

### Epidemiologically Active Andean Phylotype IIB-1 Potato Brown Rot Strain

A recent study using 17 core genomes of a IIB-1 worldwide collection from isolates sampled over several decades revealed a very limited amount of SNPs. It also suggested a South American origin of this group, which spread to the rest of the world via European Mediterranean countries (Clarke et al., 2015). These authors also noticed that all but one of their isolates from Europe, Africa, and Asia belonged to a single clonal lineage. Our study investigated a smaller spatial scale and confirmed a narrow genetic diversity of IIB-1 strains despite the use of a highly discriminatory genotyping scheme, suggesting a recent introduction of the phylotype IIB-1 in Madagascar. The changing epidemiological profile observed with potato BW in the Central Highlands resulted most likely from the recent emergence of these highly pathogenic strains, which are widely distributed in Madagascar in regions where this pathogenic population was not previously reported. This recent emergence is supported by the major CCII-A strains that are closely related to a minor CCII-B strain as well as a few haplotypes that are DLVs of CCII-A. Only one evolutionary step is missing to attach CCII-B and these singletons to CCII-A, probably the result of sampling bias. This clonal expansion structure (**Figure 2B**) may have initially derived from one or several polyclonal introductions of closely related haplotypes, likely from the same imported potato lot. This is a major contribution on genetic diversity among phylotype IIB-1 strains that was generally recognized in the literature to form a monomorphic group. Within epidemic IIB-1 strains, genetic diversity was thoroughly explored up to the CC level, and CCII-A appears to be genetically structured into three groups scattered among AEZs. CSC1 and CSC2 comprise an over-represented central haplotype from which haplotypes differing at one locus (SLV) are radially linked (haplotypes MT9 and MT3 for CSC1 and CSC2, respectively). Such CC structures may suggest one polyclonal or several mono/polyclonal introduction events in the Malagasy Central Highlands from an original population, followed by subsequent clonal expansion and divergence by mutation, or they could imply a simple pattern of clonal expansion and diversification from a primary founder (Feil et al., 2004).

Moreover, when we analyzed strains according to the survey period (April 2013 vs December 2013), the haplotypes and population structure yielded from the first survey differed strongly from those identified during the second survey, although some haplotypes (MTII-3, MTII-4, MTII-6, MTII-9, and MTII-11) were collected in both surveys (**Figure 4A**). The first survey was performed in the central potato seed production areas (Vakinankaratra region), where basic seeds were multiplied and subsequently marketed for extensive production in the remaining potato producing basins surveyed during the second period (**Figure 4D**). The haplotype MTII-3 was the most frequent (49%) in the first survey. The haplotype MTII-9 was the most frequently (73%) observed haplotype in the second survey, while MTII-3 almost disappeared (only one strain). One hypothesis is that haplotypes present in the original population may be absent in following generations when random events eliminate haplotypes from the main population, resulting in genetic drift in which haplotype frequencies change over generations due to chance (Hartl et al., 1997; Halliburton, 2004). Bottleneck and founder effect events may occur due to the sampling of contaminated potato seed tuber lots during retail sales, which are then used as seeds for planting during the next crop season. As noted above, a high genetic diversity of phylotype IIB-1 strains was observed within a field. Randomly, only a subset of the total diversity in an original potato seed tuber lot may be picked and may produce a new population. In addition, genetic bottleneck and founder events may be related to the choice of potato cultivars and crops grown in an AEZ. The choice of varieties obviously refers to the local environment and conditions in terms of growing cycle and preservation as well as taste and use. A second hypothesis is that haplotypes can spread over a geographical range, and the gene pool may vary due to adaptive ecological reasons that need to be determined (Hartl et al., 1997; Robinson et al., 2010). Such a strong selective ecological effect could explain the disappearance of MTII-3 during the second sampling as this haplotype was sampled in all of the AEZ from the first survey.

Interestingly, based on further analysis of the data, biogeographical features may also shape the current population structure of phylotype IIB-1. Genetic cluster analysis of the whole data set (collection CMG-II) clustered the phylotype IIB-1 strains into three distinct genetic groups, which supports our previous finding. The co-occurrence and large-scale distribution of both CLII-1 and CLII-2 across many AEZs is consistent with a common transmission source such as infected potato tubers, which is very well known for harboring phylotype IIB-1 strains (Graham and Lloyd, 1979; Ciampi et al., 1980; Hooker et al., 1981; French, 1984; Granada, 1988; Elphinstone, 1996; Allen et al., 2001). More interestingly, the spatial structure of the clusters mirrored potato tuber supply as they crossed different AEZs for distribution and trade (**Figure 3C**). In terms of potato seed production and supply chain, the National Centre for Rural Development and Applied Research, FIFAMANOR, located in AEZ1, is primarily in charge of the selection and multiplication of accessions that come from the International Potato Center (Peru and Kenya). FIFAMANOR is also responsible for certifying basic potato seed production and distribution and for selecting seed growers for mass production, particularly in the most important potato seed flow, AEZ1, and in every AEZ throughout the country. Due to the limited supply of certified potato seed tubers, seed growers produce and sell non-certified commercial seeds to farmers for food and commercial production. Farmer-to-farmer and neighbor seed exchanges and self-supplies are also emerging, which may lead to the quick spread of disease. Long-range dispersal of phylotype IIB-1 is probably associated with the distribution processes of potato seed tubers carrying latent infections. Furthermore, distribution routes also play a role in the dispersal of clones to different fields and local areas. At a small-scale level, an intermix of factors such alternate hosts, contaminated soil, water irrigation, and stream runoff, favor short distance dispersal. The observation of *S. lycopersicum* and *P. vulgaris* infections in water irrigation and rhizospheres indicates these are potential reservoirs for phylotype IIB-1 survival, as reported in the literature (Hayward, 1991; Swanepoel, 1992; Janse et al., 2004; Álvarez et al., 2010).

## Conclusion

This is the first molecular epidemiology study comparing two coexisting RSSC phylotypes, phylotype IIB-1 vs phylotype III, throughout the RSSC distribution area in the Central Highlands of Madagascar (**Table 1**).

**Table 1:** Contrasting epidemiological patterns of the two co-occurring RSSC strains: Andean phylotype IIB-1 and African phylotype III.

	Andean phylotype IIB-1	African phylotype III
Genotyping MLVA scheme	RS2-MLVA9	RS3-MVLVA16
Genetic diversity	Haplotype diversity within a major CC	Highly diverse haplotypes within disparate CCs
Genetic relatedness with worldwide haplotypes	Malagasy haplotypes shared with worldwide haplotypes	Highly differentiated from worldwide haplotypes
Genetic relationship between AEZs	Numerous haplotypes spread over the AEZ range	CCs associated with a single geographic origin each
Spatial population structure	Three genetic clusters Population structure mirroring potato tuber supply and shaped by biogeographical features	Eleven genetic clusters Distinct populations according to geographic regions. Population structure shaped by local adaptation
Strains origin and features	Introduced strains and epidemiologically active	Endemic strains specific to a geographic area
Transmission mode	Transmitted through latently infected potato tubers	Not efficiently transmitted through latently infected potato tubers
Dispersion	Multiple contaminations over AEZs resulting from infected potato tuber exchange	Limited to AEZs
Reservoirs	Irrigation water, weeds, crop hosts (beans…)	Irrigation water, weeds, crop hosts (solanaceous crops, pelargonium…)


MLVA schemes RS2-MLVA9 and RS3-MLVA16 genotyped a large collection of RSSC phylotype IIB-1 and phylotype III strains, respectively, and allowed a thorough exploration of the diversity of these two phylotypes giving patterns of their distribution. This allowed us to identify the types of outbreak- and epidemic-associated strains and those associated with recurrent BW in the Central Highlands of Madagascar. The previously developed MLVA scheme RS3-MVA16 (Ravelomanantsoa et al., 2016) allowed the differentiation of geographically distant phylotype III strains and epidemiologically linked related strains at several scales: in potato growing basins and AEZs and at the field scale. On the other hand, the MLVA scheme RS2-MLVA9 opened the possibility of subtyping monomorphic phylotype IIB-1 strains and epidemiologically connected strains at different scales.

Our findings highlight two contrasting epidemiological patterns of Andean brown rot and African brown rot. The recent epidemic BW occurring in the Central Highlands of Madagascar is associated with the introduced phylotype IIB-1. Latently infected potato tubers quickly and widely propagated these strains due to their ability to cause latent infection in potato seeds resulting in a strong epidemic pattern for phylotype IIB-1 strains. Conversely, diverse phylotype III strains were involved in persistent BW disease outbreaks in potato growing basins. Considering the spatial organization of their genetic diversity, the rare transmission of phylotype III strains resulted from potato tuber distribution. However, these strains are permanently locally present in distinct areas. In addition, phylotype IIB-1 strains were described for the first time in Madagascar, providing insight into their population biology. This is the first epidemiological report describing haplotypes within the globally well-known genetically clonal IIB-1.

The MLVA schemes RS2-MLVA9 and RS3-MLVA16, which are well-suited and compatible for genotyping RSSC strains, may be applied as a routine genotyping tool for the fine-scale epidemiological trace-back analysis of Andean brown rot strains (phylotype IIB-1) and African brown rot strains (phylotype III), respectively, and for the surveillance and control of BW in Madagascar and around the world.

## Author Contributions

All authors listed have made substantial contributions to the work. SR, PP, IR, and FG designed the study, wrote the manuscript, analyzed the data, and supervised the project. CV, AR, OP, GC, and SP analyzed the data and revised the final version. SA, LC, and FC collaborated in sampling and molecular investigations.

## Conflict of Interest Statement

The authors declare that the research was conducted in the absence of any commercial or financial relationships that could be construed as a potential conflict of interest.

## Footnotes

1 http://insilico.ehu.es/mini_tools/discriminatory_power/

## Supplementary Material

**FIGURE S1 |** Locations of the potato producing areas in Madagascar and a map of the study areas located in the Central Highlands of Madagascar indicating the general topography of the Ankaratra Mountains and the 12 AEZs.

**TABLE S1 |** List of RSSC strains used in this study and corresponding genotyping data. List of hosts: A, *Anthurium andreanum*; AH, *Arachis hypogaea*; BP, *Bidens pilosa*; CA, *Capsicum annuum*; MA, *Morus alba*; MU, *Musa* sp.; NT, *Nicotiana tabacum*; P, *Pelargonium* sp.; PV, *Phaseolus vulgaris*; S, Rhizosphere; SG, *Solanum gilo*; SL, *Solanum lycopersicum*; SM, *Solanum melongena*; Sma, *Solanum macrocarpon*; SN, *Solanum nigrum*; SO, *Symphytum officinale*; SP, *Solanum panduriforme*; SS, *Solanum scabrum*; ST, *Solanum tuberosum*; W, Water of irrigation; ZO, *Zingiber officinale*; AEZ, Agroecological zone; masl, meters above sea level.

**TABLE S2 |** GenBank accession numbers for the *egl* sequences used in this study. Accession numbers of the sequences retrieved from GenBank are in italics. Accession numbers of new sequences are in bold.

**TABLE S3 |** Description of TR markers, corresponding oligonucleotide primers and multiplex combinations, and PCR conditions used in this study. **(A)** The nomenclature for the TR locus was as follows: marker alias_replicon of origin (ch: chromosome or mp: megaplasmid)_start physical position expressed in kilobases in the genome of strain origin_TR unit size (bp)_amplicon size in the genome of strain origin (bp)_number of repeats (units) (e.g., RS1L12_1774_6bp_196bp_12u) (Le Fléche et al., 2002; N’Guessan et al., 2013). The marker alias is RS (*R. solanacearum*), followed by the phylotype number of origin (1, 2, 3, or 4), and the reference of the locus (e.g., RS3L12). **(B)** All positions are in the genome of the strain origin. Accession numbers: CMR15 chromosome (FP885895), CMR15 megaplasmid (FP885896), Molk2 genome (GCF_000212635.3), UW551 genome (GCF_000167955.1), IPO1609 genome (GCF_001050995.1), and CFBP2957 genome (FP885897.1). **(C)** Fluorescent dye labeled at the 5′ end of the forward primer.
