# Supplementary figures and images for: Molecular Epidemiology of Bacterial Wilt in the Madagascar Highlands Caused by Andean (Phylotype IIB-1) and African (Phylotype III) Brown Rot Strains of the Ralstonia solanacearum Species Complex
Source: Front Plant Sci. 2018 Jan 15;8:2258. doi: 10.3389/fpls.2017.02258 (PMC5775269; doi:10.3389/fpls.2017.02258)

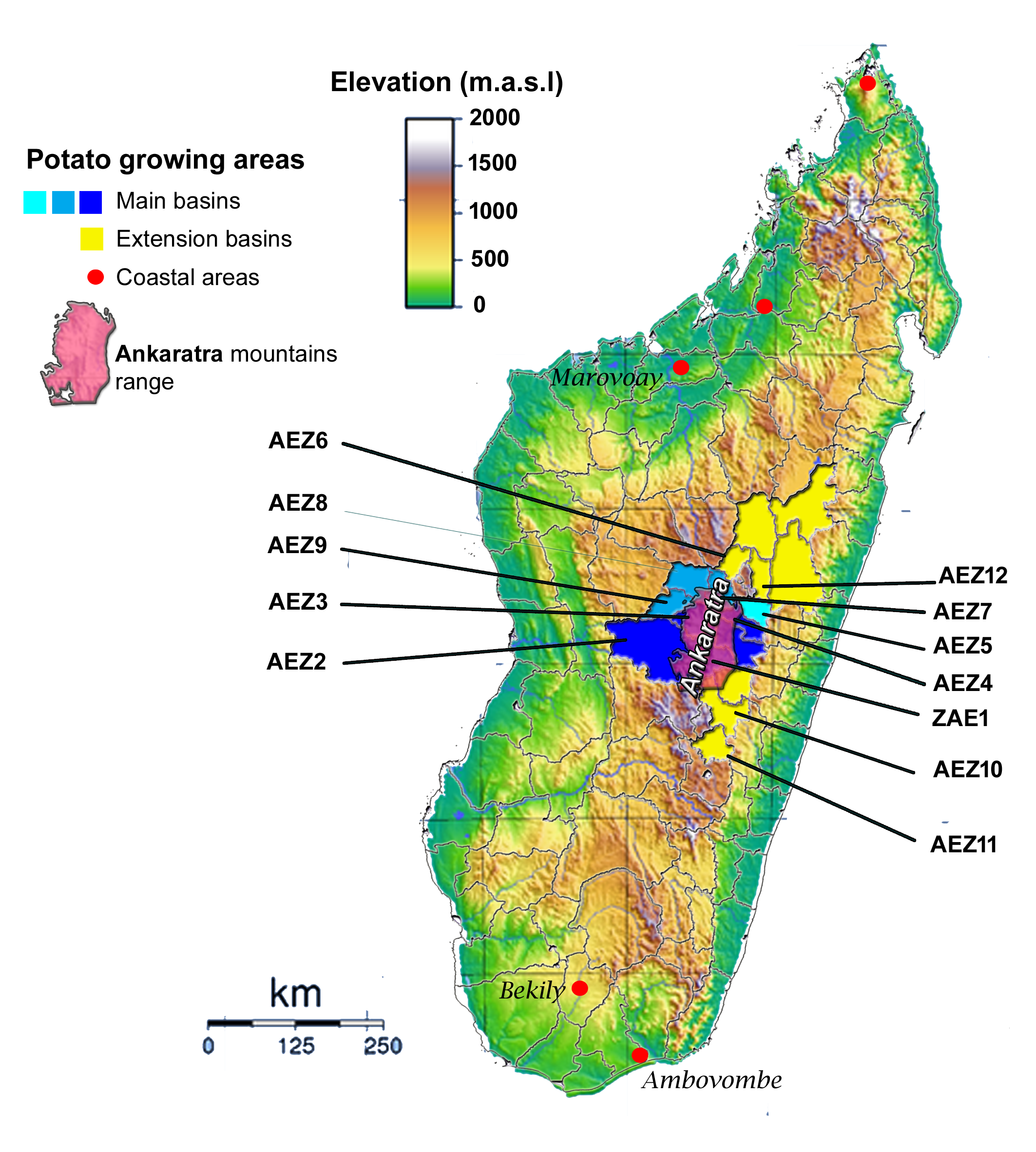

Supplement: Supplementary file 1 [file Image_1.TIF]
