# Supplementary material for: Molecular Epidemiology of Bacterial Wilt in the Madagascar Highlands Caused by Andean (Phylotype IIB-1) and African (Phylotype III) Brown Rot Strains of the Ralstonia solanacearum Species Complex
Source: Front Plant Sci. 2018 Jan 15;8:2258. doi: 10.3389/fpls.2017.02258 (PMC5775269; doi:10.3389/fpls.2017.02258)
Supplement: Supplementary file 2 [file Table_1.DOCX]

**Supplementary Table 1.** **List of *Ralstonia solanacearum* species complex strains used in this study and corresponding genotyping data.** List of hosts: A, *Anthurium andreanum*. AH, *Arachis hypogeaea*. BP, *Bidens pilosa*. CA, *Capsicum annuum*. MA, *Morus alba*. MU, *Musa* sp. NT, *Nicotiana tabacum*. P, *Pelargonium* sp. PV, *Phaseolus vulgaris*. S, Rhizosphere. SG, *Solanum gilo*. SL, *Solanum lycopersicum*. SM, *Solanum melongena*. Sma, *Solanum macrocarpon*. SN, *Solanum nigrum*. SO, *Symphytum officinale*. SP, *Solanum panduraforme*. SS, *Solanum scabrum*. ST, *Solanum tuberosum*. W, Water of irrigation. ZO, *Zingiber officinale*. AEZ, Agroecological zone. m.a.s.l, meter above sea level.

| **RUN ID** | **STRAIN ID** | **YEAR** | **COUNTRY** | **AEZ** | **SITE** | **ELEVATION (m.a.s.l)** | **LATITUDE** | **LONGITUDE** | **PERIOD of SURVEY** | **AVERAGE TEMPERATURE (°C)** | **HOST** | **SEQUEVAR** | **MLVA SCHEME** | **MLVA PROFILE** | **MLVA types** | **CLONAL COMPLEX** | **CLUSTER** | **COLLECTION** |
| --- | --- | --- | --- | --- | --- | --- | --- | --- | --- | --- | --- | --- | --- | --- | --- | --- | --- | --- |
|  |  |  |  |  |  |  |  |  |  |  |  |  |  |  |  |  |  |  |
| **PHYLOTYPE I** | |  |  |  |  |  |  |  |  |  |  |  |  |  |  |  |  |  |
| 44 | ACH92 | 1966 | Australia | - | - | - | - | - |  | - | ZO | 16 | - | - | - | - | - | CREF-I |
| 47 | CIP365 | 1989 | Philippines | - | - | - | - | - |  | - | ST | 45 | - | - | - | - | - | CREF-I |
| 54 | GMI1000 | 1978 | French Guiana | - | - | - | - | - |  | - | SL | 18 | - | - | - | - | - | CREF-I |
| 69 | MAFF211266 | - | Japan | - | - | - | - | - |  | - | SL | 15 | - | - | - | - | - | CREF-I |
| 85 | PSS190 | 1995 | Taiwan | - | - | - | - | - |  | - | SL | 15 | - | - | - | - | - | CREF-I |
| 90 | R288 | - | China | - | - | - | - | - |  | - | MA | 12 | - | - | - | - | - | CREF-I |
| 91 | R292 | - | China | - | - | - | - | - |  | - | MA | 12 | - | - | - | - | - | CREF-I |
| 135 | CFBP7026 | 2005 | Cameroon | - | - | - | - | - |  | - | SL | 44 | - | - | - | - | - | CREF-I |
| 155 | PSS366 | 2003 | Taiwan | - | - | - | - | - |  | - | SL | 15 | - | - | - | - | - | CREF-I |
| 183 | CFBP7046 | 2005 | Cameroon | - | - | - | - | - |  | - | SS | 13 | - | - | - | - | - | CREF-I |
| 215 | CFBP7058 | 2005 | Cameroon | - | - | - | - | - |  | - | SS | 13 | - | - | - | - | - | CREF-I |
| 257 | PSS219 | 1996 | Taiwan | - | - | - | - | - |  | - | SL | 12 | - | - | - | - | - | CREF-I |
| 258 | PSS81 | - | Taiwan | - | - | - | - | - |  | - | SL | 14 | - | - | - | - | - | CREF-I |
| 304 | MAD-001 | 2006 | Madagascar | - | - | 1321 | - | - |  | - | SL | 18 | - | - | - | - | - | CREF-I |
| 305 | MAD-002 | 2006 | Madagascar | - | - | 1321 | - | - |  | - | SL | 18 | - | - | - | - | - | CREF-I |
| 306 | MAD-003 | 2006 | Madagascar | - | - | 15 | - | - |  | - | CA | 18 | - | - | - | - | - | CREF-I |
| 309 | MAD-006 | 2006 | Madagascar | - | - | 15 | - | - |  | - | CA | 18 | - | - | - | - | - | CREF-I |
| 312 | MAD-009 | 2006 | Madagascar | - | - | 21 | - | - |  | - | SM | 18 | - | - | - | - | - | CREF-I |
| 317 | MAD-014 | 2006 | Madagascar | - | - | 30 | - | - |  | - | SS | 18 | - | - | - | - | - | CREF-I |
| 318 | MAD-015 | 2006 | Madagascar | - | - | 30 | - | - |  | - | SM | 18 | - | - | - | - | - | CREF-I |
| 319 | MAD-016 | 2006 | Madagascar | - | - | - | - | - |  | - | CA | 46 | - | - | - | - | - | CREF-I |
| 320 | MAD-017 | 2006 | Madagascar | - | - | 27 | - | - |  | - | CA | 46 | - | - | - | - | - | CREF-I |
| 328 | MAD-025 | 2006 | Madagascar | - | - | 15 | - | - |  | - | CA | 18 | - | - | - | - | - | CREF-I |
| 337 | P11 | - | China | - | - | - | - | - |  | - | AH | 17 | - | - | - | - | - | CREF-I |
| 343 | M02 | - | China | - | - | - | - | - |  | - | MA | 48 | - | - | - | - | - | CREF-I |
| 466 | JS759 | 1968 | Trinidad | - | - | - | - | - |  | - | MU | 18 | - | - | - | - | - | CREF-I |
| 471 | JT519 | 1993 | Reunion | - | - | - | - | - |  | - | P | 31 | - | - | - | - | - | CREF-I |
| 597 | GMI8254 | - | Indonesia | - | - | - | - | - |  | - | SL | 47 | - | - | - | - | - | CREF-I |
| 608 | JT523 | - | Reunion | - | - | - | - | - |  | - | ST | 13 | - | - | - | - | - | CREF-I |
| 1389 | CMR22 | 2005 | Cameroon | - | - | - | - | - |  | - | SL | 18 | - | - | - | - | - | CREF-I |
| 1528 | UW558 | 2004 | Guatemala | - | - | - | - | - |  | - | SL | 14 | - | - | - | - | - | CREF-I |
| 1798 | CIV61 | 2010 | Ivory Coast | - | - | - | - | - |  | - | SM | 13 | - | - | - | - | - | CREF-I |
| 1801 | CIV64 | 2010 | Ivory Coast | - | - | - | - | - |  | - | CA | 31 | - | - | - | - | - | CREF-I |
| 1833 | CIV96 | 2010 | Ivory Coast | - | - | - | - | - |  | - | SM | 44 | - | - | - | - | - | CREF-I |
| 1880 | CIV124 | 2011 | Ivory Coast | - | - | - | - | - |  | - | SL | 46 | - | - | - | - | - | CREF-I |
| 1919 | CIV163 | 2011 | Ivory Coast | - | - | - | - | - |  | - | CA | 14 | - | - | - | - | - | CREF-I |
| 1948 | E02PO10 | 2011 | French Guiana | - | - | - | - | - |  | - | CA | 17 | - | - | - | - | - | CREF-I |
| 2177 | E'4T3 | 2012 | French Guiana | - | - | - | - | - |  | - | SL | 47 | - | - | - | - | - | CREF-I |
| 2231 | E'8B13P4 | 2012 | French Guiana | - | - | - | - | - |  | - | SL | 17 | - | - | - | - | - | CREF-I |
| 2241 | E8B11P7 | 2012 | French Guiana | - | - | - | - | - |  | - | SL | 14 | - | - | - | - | - | CREF-I |
| 3075 | - | 2013 | Madagascar | AEZ7 | S52 | 1340 | -19,01 | 47,27 | DECEMBER | 21.9 | SL | - | - | - | - | - | - | **-** |
| 3076 | - | 2013 | Madagascar | AEZ7 | S52 | 1340 | -19,01 | 47,27 | DECEMBER | 21.9 | SL | - | - | - | - | - | - | **-** |
| 3077 | - | 2013 | Madagascar | AEZ7 | S52 | 1340 | -19,01 | 47,27 | DECEMBER | 21.9 | SL | 18 | - | - | - | - | - | **-** |
| 3078 | - | 2013 | Madagascar | AEZ7 | S52 | 1340 | -19,01 | 47,27 | DECEMBER | 21.9 | SL | 18 | - | - | - | - | - | **-** |
| 3080 | - | 2013 | Madagascar | AEZ7 | S52 | 1340 | -19,01 | 47,27 | DECEMBER | 21.9 | SL | 18 | - | - | - | - | - | **-** |
| 3082 | - | 2013 | Madagascar | AEZ7 | S52 | 1340 | -19,01 | 47,27 | DECEMBER | 21.9 | SL | 18 | - | - | - | - | - | **-** |
| 3083 | - | 2013 | Madagascar | AEZ7 | S52 | 1340 | -19,01 | 47,27 | DECEMBER | 21.9 | SL | 18 | - | - | - | - | - | **-** |
| 3084 | - | 2013 | Madagascar | AEZ7 | S52 | 1340 | -19,01 | 47,27 | DECEMBER | 21.9 | SL | 18 | - | - | - | - | - | **-** |
| 3085 | - | 2013 | Madagascar | AEZ7 | S52 | 1340 | -19,01 | 47,27 | DECEMBER | 21.9 | SL | 18 | - | - | - | - | - | **-** |
| 3086 | - | 2013 | Madagascar | AEZ7 | S52 | 1340 | -19,01 | 47,27 | DECEMBER | 21.9 | SL | - | - | - | - | - | - | **-** |
| 3087 | - | 2013 | Madagascar | AEZ7 | S52 | 1340 | -19,01 | 47,27 | DECEMBER | 21.9 | SL | - | - | - | - | - | - | **-** |
| 3089 | - | 2013 | Madagascar | AEZ7 | S52 | 1340 | -19,01 | 47,27 | DECEMBER | 21.9 | SL | 18 | - | - | - | - | - | **-** |
| 3108 | - | 2013 | Madagascar | AEZ7 | S52 | 1340 | -19,01 | 47,27 | DECEMBER | 21.9 | SL | 18 | - | - | - | - | - | **-** |
| 3109 | - | 2013 | Madagascar | AEZ7 | S52 | 1340 | -19,01 | 47,27 | DECEMBER | 21.9 | SL | 18 | - | - | - | - | - | **-** |
| 3110 | - | 2013 | Madagascar | AEZ7 | S52 | 1340 | -19,01 | 47,27 | DECEMBER | 21.9 | SL | 18 | - | - | - | - | - | **-** |
| 3111 | - | 2013 | Madagascar | AEZ7 | S52 | 1340 | -19,01 | 47,27 | DECEMBER | 21.9 | SL | 18 | - | - | - | - | - | **-** |
| 3112 | - | 2013 | Madagascar | AEZ7 | S52 | 1340 | -19,01 | 47,27 | DECEMBER | 21.9 | SL | 18 | - | - | - | - | - | **-** |
| 3113 | - | 2013 | Madagascar | AEZ7 | S52 | 1340 | -19,01 | 47,27 | DECEMBER | 21.9 | SL | 18 | - | - | - | - | - | **-** |
| 3114 | - | 2013 | Madagascar | AEZ7 | S52 | 1340 | -19,01 | 47,27 | DECEMBER | 21.9 | SL | 18 | - | - | - | - | - | **-** |
| 3115 | - | 2013 | Madagascar | AEZ7 | S52 | 1340 | -19,01 | 47,27 | DECEMBER | 21.9 | SL | - | - | - | - | - | - | **-** |
| 3116 | - | 2013 | Madagascar | AEZ7 | S52 | 1340 | -19,01 | 47,27 | DECEMBER | 21.9 | SL | - | - | - | - | - | - | **-** |
| 3117 | - | 2013 | Madagascar | AEZ7 | S52 | 1340 | -19,01 | 47,27 | DECEMBER | 21.9 | SL | - | - | - | - | - | - | **-** |
| 3118 | - | 2013 | Madagascar | AEZ7 | S52 | 1340 | -19,01 | 47,27 | DECEMBER | 21.9 | SL | - | - | - | - | - | - | **-** |
| 3119 | - | 2013 | Madagascar | AEZ7 | S52 | 1340 | -19,01 | 47,27 | DECEMBER | 21.9 | SL | - | - | - | - | - | - | **-** |
| 3120 | - | 2013 | Madagascar | AEZ7 | S52 | 1340 | -19,01 | 47,27 | DECEMBER | 21.9 | SL | 18 | - | - | - | - | - | **-** |
| 3121 | - | 2013 | Madagascar | AEZ7 | S52 | 1340 | -19,01 | 47,27 | DECEMBER | 21.9 | SL | - | - | - | - | - | - | **-** |
| 3122 | - | 2013 | Madagascar | AEZ7 | S52 | 1340 | -19,01 | 47,27 | DECEMBER | 21.9 | SL | - | - | - | - | - | - | **-** |
| 3124 | - | 2013 | Madagascar | AEZ7 | S52 | 1340 | -19,01 | 47,27 | DECEMBER | 21.9 | SL | - | - | - | - | - | - | **-** |
| 3125 | - | 2013 | Madagascar | AEZ7 | S52 | 1340 | -19,01 | 47,27 | DECEMBER | 21.9 | SL | - | - | - | - | - | - | **-** |
| 3126 | - | 2013 | Madagascar | AEZ7 | S52 | 1340 | -19,01 | 47,27 | DECEMBER | 21.9 | SL | - | - | - | - | - | - | **-** |
| 3128 | - | 2013 | Madagascar | AEZ6 | S9 | 1252 | -18,76 | 47,34 | DECEMBER | 20.2 | SL | - | - | - | - | - | - | **-** |
| 3129 | - | 2013 | Madagascar | AEZ6 | S9 | 1252 | -18,76 | 47,34 | DECEMBER | 20.2 | SL | 18 | - | - | - | - | - | **-** |
| 3130 | - | 2013 | Madagascar | AEZ6 | S9 | 1252 | -18,76 | 47,34 | DECEMBER | 20.2 | SL | - | - | - | - | - | - | **-** |
| 3131 | - | 2013 | Madagascar | AEZ6 | S9 | 1252 | -18,76 | 47,34 | DECEMBER | 20.2 | SL | - | - | - | - | - | - | **-** |
| 3134 | - | 2013 | Madagascar | AEZ6 | S9 | 1252 | -18,76 | 47,34 | DECEMBER | 20.2 | SL | 18 | - | - | - | - | - | **-** |
| 3135 | - | 2013 | Madagascar | AEZ6 | S9 | 1252 | -18,76 | 47,34 | DECEMBER | 20.2 | SL | - | - | - | - | - | - | **-** |
| 3136 | - | 2013 | Madagascar | AEZ6 | S9 | 1252 | -18,76 | 47,34 | DECEMBER | 20.2 | SL | 18 | - | - | - | - | - | **-** |
| 3137 | - | 2013 | Madagascar | AEZ6 | S9 | 1252 | -18,76 | 47,34 | DECEMBER | 20.2 | SL | 18 | - | - | - | - | - | **-** |
| 3138 | - | 2013 | Madagascar | AEZ6 | S9 | 1252 | -18,76 | 47,34 | DECEMBER | 20.2 | SL | - | - | - | - | - | - | **-** |
| 3142 | - | 2013 | Madagascar | AEZ7 | S52 | 1340 | -19,01 | 47,27 | DECEMBER | 21.9 | SL | 18 | - | - | - | - | - | **-** |
| 3148 | - | 2013 | Madagascar | AEZ6 | S7 | 1251 | -18,72 | 47,28 | DECEMBER | 20.2 | ST | 33 | - | - | - | - | - | **-** |
| 3149 | - | 2013 | Madagascar | AEZ6 | S7 | 1251 | -18,72 | 47,28 | DECEMBER | 20.2 | ST | 33 | - | - | - | - | - | **-** |
| 3151 | - | 2013 | Madagascar | AEZ6 | S7 | 1251 | -18,72 | 47,28 | DECEMBER | 20.2 | ST | 33 | - | - | - | - | - | **-** |
| 3159 | - | 2013 | Madagascar | AEZ6 | S8 | 1266 | -18,80 | 47,42 | DECEMBER | 20.2 | SL | 18 | - | - | - | - | - | **-** |
| 3162 | - | 2013 | Madagascar | AEZ6 | S8 | 1266 | -18,80 | 47,42 | DECEMBER | 20.2 | ST | 18 | - | - | - | - | - | **-** |
| 3163 | - | 2013 | Madagascar | AEZ6 | S8 | 1266 | -18,80 | 47,42 | DECEMBER | 20.2 | ST | 18 | - | - | - | - | - | **-** |
| 3164 | - | 2013 | Madagascar | AEZ6 | S8 | 1266 | -18,80 | 47,42 | DECEMBER | 20.2 | ST | 18 | - | - | - | - | - | **-** |
| 3165 | - | 2013 | Madagascar | AEZ6 | S8 | 1266 | -18,80 | 47,42 | DECEMBER | 20.2 | ST | - | - | - | - | - | - | **-** |
| 3168 | - | 2013 | Madagascar | AEZ6 | S8 | 1266 | -18,80 | 47,42 | DECEMBER | 20.2 | ST | - | - | - | - | - | - | **-** |
| 3170 | - | 2013 | Madagascar | AEZ6 | S8 | 1266 | -18,80 | 47,42 | DECEMBER | 20.2 | ST | - | - | - | - | - | - | **-** |
| 3171 | - | 2013 | Madagascar | AEZ6 | S8 | 1266 | -18,80 | 47,42 | DECEMBER | 20.2 | SN | 18 | - | - | - | - | - | **-** |
| 3180 | - | 2013 | Madagascar | AEZ7 | S51 | 1477 | -19,08 | 47,25 | DECEMBER | 21.9 | ST | 18 | - | - | - | - | - | **-** |
| 3208 | - | 2013 | Madagascar | AEZ7 | S50 | 1433 | -19,09 | 47,25 | DECEMBER | 21.9 | ST | 18 | - | - | - | - | - | **-** |
| 3210 | - | 2013 | Madagascar | AEZ8 | S70 | 1176 | -18,98 | 46,72 | DECEMBER | 21.3 | SL | 18 | - | - | - | - | - | **-** |
| 3211 | - | 2013 | Madagascar | AEZ8 | S70 | 1176 | -18,98 | 46,72 | DECEMBER | 21.3 | SL | 33 | - | - | - | - | - | **-** |
| 3212 | - | 2013 | Madagascar | AEZ8 | S70 | 1176 | -18,98 | 46,72 | DECEMBER | 21.3 | SL | - | - | - | - | - | - | **-** |
| 3213 | - | 2013 | Madagascar | AEZ8 | S70 | 1176 | -18,98 | 46,72 | DECEMBER | 21.3 | SL | 18 | - | - | - | - | - | **-** |
| 3214 | - | 2013 | Madagascar | AEZ8 | S70 | 1176 | -18,98 | 46,72 | DECEMBER | 21.3 | SL | 18 | - | - | - | - | - | **-** |
| 3215 | - | 2013 | Madagascar | AEZ8 | S70 | 1176 | -18,98 | 46,72 | DECEMBER | 21.3 | SL | - | - | - | - | - | - | **-** |
| 3216 | - | 2013 | Madagascar | AEZ8 | S70 | 1176 | -18,98 | 46,72 | DECEMBER | 21.3 | SL | 33 | - | - | - | - | - | **-** |
| 3217 | - | 2013 | Madagascar | AEZ8 | S70 | 1176 | -18,98 | 46,72 | DECEMBER | 21.3 | SL | - | - | - | - | - | - | **-** |
| 3218 | - | 2013 | Madagascar | AEZ8 | S70 | 1176 | -18,98 | 46,72 | DECEMBER | 21.3 | SL | - | - | - | - | - | - | **-** |
| 3219 | - | 2013 | Madagascar | AEZ8 | S70 | 1176 | -18,98 | 46,72 | DECEMBER | 21.3 | SL | 18 | - | - | - | - | - | **-** |
| 3220 | - | 2013 | Madagascar | AEZ8 | S70 | 1176 | -18,98 | 46,72 | DECEMBER | 21.3 | SL | - | - | - | - | - | - | **-** |
| 3221 | - | 2013 | Madagascar | AEZ8 | S70 | 1176 | -18,98 | 46,72 | DECEMBER | 21.3 | SL | - | - | - | - | - | - | **-** |
| 3222 | - | 2013 | Madagascar | AEZ8 | S70 | 1176 | -18,98 | 46,72 | DECEMBER | 21.3 | SL | 18 | - | - | - | - | - | **-** |
| 3223 | - | 2013 | Madagascar | AEZ8 | S70 | 1176 | -18,98 | 46,72 | DECEMBER | 21.3 | SL | - | - | - | - | - | - | **-** |
| 3224 | - | 2013 | Madagascar | AEZ8 | S70 | 1176 | -18,98 | 46,72 | DECEMBER | 21.3 | SL | - | - | - | - | - | - | **-** |
| 3226 | - | 2013 | Madagascar | AEZ8 | S70 | 1176 | -18,98 | 46,72 | DECEMBER | 21.3 | SL | - | - | - | - | - | - | **-** |
| 3228 | - | 2013 | Madagascar | AEZ8 | S70 | 1176 | -18,98 | 46,72 | DECEMBER | 21.3 | SL | - | - | - | - | - | - | **-** |
| 3243 | - | 2013 | Madagascar | AEZ8 | S71 | 1194 | -18,98 | 46,73 | DECEMBER | 21.3 | CA | 18 | - | - | - | - | - | **-** |
| 3245 | - | 2013 | Madagascar | AEZ9 | S73 | 1256 | -19,08 | 46,75 | DECEMBER | 19.4 | SG | - | - | - | - | - | - | **-** |
| 3246 | - | 2013 | Madagascar | AEZ9 | S73 | 1256 | -19,08 | 46,75 | DECEMBER | 19.4 | SG | - | - | - | - | - | - | **-** |
| 3247 | - | 2013 | Madagascar | AEZ9 | S73 | 1256 | -19,08 | 46,75 | DECEMBER | 19.4 | SG | 18 | - | - | - | - | - | **-** |
| 3256 | - | 2013 | Madagascar | AEZ9 | S74 | 1377 | -19,13 | 46,73 | DECEMBER | 19.4 | ST | 18 | - | - | - | - | - | **-** |
| 3257 | - | 2013 | Madagascar | AEZ9 | S74 | 1377 | -19,13 | 46,73 | DECEMBER | 19.4 | ST | 18 | - | - | - | - | - | **-** |
| 3264 | - | 2013 | Madagascar | AEZ9 | S72 | 1230 | -19,04 | 46,73 | DECEMBER | 19.4 | SG | 18 | - | - | - | - | - | **-** |
| 3265 | - | 2013 | Madagascar | AEZ9 | S72 | 1230 | -19,04 | 46,73 | DECEMBER | 19.4 | SG | - | - | - | - | - | - | **-** |
| 3266 | - | 2013 | Madagascar | AEZ9 | S72 | 1230 | -19,04 | 46,73 | DECEMBER | 19.4 | SG | - | - | - | - | - | - | **-** |
| 3267 | - | 2013 | Madagascar | AEZ9 | S72 | 1230 | -19,04 | 46,73 | DECEMBER | 19.4 | SG | - | - | - | - | - | - | **-** |
| 3268 | - | 2013 | Madagascar | AEZ9 | S72 | 1230 | -19,04 | 46,73 | DECEMBER | 19.4 | SG | - | - | - | - | - | - | **-** |
| 3269 | - | 2013 | Madagascar | AEZ9 | S72 | 1230 | -19,04 | 46,73 | DECEMBER | 19.4 | SG | - | - | - | - | - | - | **-** |
| 3270 | - | 2013 | Madagascar | AEZ9 | S72 | 1230 | -19,04 | 46,73 | DECEMBER | 19.4 | SG | - | - | - | - | - | - | **-** |
| 3271 | - | 2013 | Madagascar | AEZ9 | S72 | 1230 | -19,04 | 46,73 | DECEMBER | 19.4 | SG | - | - | - | - | - | - | **-** |
| 3272 | - | 2013 | Madagascar | AEZ9 | S72 | 1230 | -19,04 | 46,73 | DECEMBER | 19.4 | SG | - | - | - | - | - | - | **-** |
| 3273 | - | 2013 | Madagascar | AEZ9 | S72 | 1230 | -19,04 | 46,73 | DECEMBER | 19.4 | SG | - | - | - | - | - | - | **-** |
| 3274 | - | 2013 | Madagascar | AEZ9 | S72 | 1230 | -19,04 | 46,73 | DECEMBER | 19.4 | SG | - | - | - | - | - | - | **-** |
| 3275 | - | 2013 | Madagascar | AEZ9 | S72 | 1230 | -19,04 | 46,73 | DECEMBER | 19.4 | SG | 18 | - | - | - | - | - | **-** |
| 3276 | - | 2013 | Madagascar | AEZ9 | S72 | 1230 | -19,04 | 46,73 | DECEMBER | 19.4 | SG | - | - | - | - | - | - | **-** |
| 3277 | - | 2013 | Madagascar | AEZ9 | S72 | 1230 | -19,04 | 46,73 | DECEMBER | 19.4 | SG | 18 | - | - | - | - | - | **-** |
| 3278 | - | 2013 | Madagascar | AEZ9 | S72 | 1230 | -19,04 | 46,73 | DECEMBER | 19.4 | SG | 18 | - | - | - | - | - | **-** |
| 3279 | - | 2013 | Madagascar | AEZ9 | S72 | 1230 | -19,04 | 46,73 | DECEMBER | 19.4 | SG | 18 | - | - | - | - | - | **-** |
| 3280 | - | 2013 | Madagascar | AEZ9 | S72 | 1230 | -19,04 | 46,73 | DECEMBER | 19.4 | SG | 18 | - | - | - | - | - | **-** |
| 3281 | - | 2013 | Madagascar | AEZ9 | S72 | 1230 | -19,04 | 46,73 | DECEMBER | 19.4 | SG | 18 | - | - | - | - | - | **-** |
| 3282 | - | 2013 | Madagascar | AEZ9 | S72 | 1230 | -19,04 | 46,73 | DECEMBER | 19.4 | SG | - | - | - | - | - | - | **-** |
| 3283 | - | 2013 | Madagascar | AEZ9 | S72 | 1230 | -19,04 | 46,73 | DECEMBER | 19.4 | SG | - | - | - | - | - | - | **-** |
| 3284 | - | 2013 | Madagascar | AEZ9 | S72 | 1230 | -19,04 | 46,73 | DECEMBER | 19.4 | SG | 18 | - | - | - | - | - | **-** |
| 3285 | - | 2013 | Madagascar | AEZ9 | S72 | 1230 | -19,04 | 46,73 | DECEMBER | 19.4 | SG | 18 | - | - | - | - | - | **-** |
| 3286 | - | 2013 | Madagascar | AEZ6 | S7 | 1251 | -18,72 | 47,28 | DECEMBER | 20.2 | ST | 18 | - | - | - | - | - | **-** |
| 3287 | - | 2013 | Madagascar | AEZ6 | S7 | 1251 | -18,72 | 47,28 | DECEMBER | 20.2 | ST | - | - | - | - | - | - | **-** |
| 3288 | - | 2013 | Madagascar | AEZ6 | S7 | 1251 | -18,72 | 47,28 | DECEMBER | 20.2 | ST | 18 | - | - | - | - | - | **-** |
| 3289 | - | 2013 | Madagascar | AEZ6 | S7 | 1251 | -18,72 | 47,28 | DECEMBER | 20.2 | ST | 18 | - | - | - | - | - | **-** |
| 3290 | - | 2013 | Madagascar | AEZ6 | S7 | 1251 | -18,72 | 47,28 | DECEMBER | 20.2 | ST | 18 | - | - | - | - | - | **-** |
| 3291 | - | 2013 | Madagascar | AEZ6 | S7 | 1251 | -18,72 | 47,28 | DECEMBER | 20.2 | ST | 18 | - | - | - | - | - | **-** |
| 3292 | - | 2013 | Madagascar | AEZ6 | S7 | 1251 | -18,72 | 47,28 | DECEMBER | 20.2 | ST | 18 | - | - | - | - | - | **-** |
| 3457 | - | 2013 | Madagascar | AEZ9 | S73 | 1256 | -19,08 | 46,75 | DECEMBER | 19.4 | SG | - | - | - | - | - | - | **-** |
| 3458 | - | 2013 | Madagascar | AEZ9 | S73 | 1256 | -19,08 | 46,75 | DECEMBER | 19.4 | SG | 18 | - | - | - | - | - | **-** |
| 3459 | - | 2013 | Madagascar | AEZ9 | S73 | 1256 | -19,08 | 46,75 | DECEMBER | 19.4 | SG | 18 | - | - | - | - | - | **-** |
| 3460 | - | 2013 | Madagascar | AEZ9 | S73 | 1256 | -19,08 | 46,75 | DECEMBER | 19.4 | SG | 18 | - | - | - | - | - | **-** |
| 3461 | - | 2013 | Madagascar | AEZ9 | S73 | 1256 | -19,08 | 46,75 | DECEMBER | 19.4 | SG | 18 | - | - | - | - | - | **-** |
| 3462 | - | 2013 | Madagascar | AEZ9 | S73 | 1256 | -19,08 | 46,75 | DECEMBER | 19.4 | SG | 18 | - | - | - | - | - | **-** |
| 3463 | - | 2013 | Madagascar | AEZ9 | S73 | 1256 | -19,08 | 46,75 | DECEMBER | 19.4 | SG | - | - | - | - | - | - | **-** |
| 3464 | - | 2013 | Madagascar | AEZ9 | S73 | 1256 | -19,08 | 46,75 | DECEMBER | 19.4 | SG | 18 | - | - | - | - | - | **-** |
| 3465 | - | 2013 | Madagascar | AEZ9 | S73 | 1256 | -19,08 | 46,75 | DECEMBER | 19.4 | SG | - | - | - | - | - | - | **-** |
| 3466 | - | 2013 | Madagascar | AEZ9 | S73 | 1256 | -19,08 | 46,75 | DECEMBER | 19.4 | SG | 18 | - | - | - | - | - | **-** |
| 3467 | - | 2013 | Madagascar | AEZ9 | S73 | 1256 | -19,08 | 46,75 | DECEMBER | 19.4 | SG | 18 | - | - | - | - | - | **-** |
| 3468 | - | 2013 | Madagascar | AEZ9 | S73 | 1256 | -19,08 | 46,75 | DECEMBER | 19.4 | SG | 18 | - | - | - | - | - | **-** |
| 3469 | - | 2013 | Madagascar | AEZ9 | S73 | 1256 | -19,08 | 46,75 | DECEMBER | 19.4 | SG | 18 | - | - | - | - | - | **-** |
| 3470 | - | 2013 | Madagascar | AEZ9 | S73 | 1256 | -19,08 | 46,75 | DECEMBER | 19.4 | SG | 18 | - | - | - | - | - | **-** |
| 3471 | - | 2013 | Madagascar | AEZ9 | S73 | 1256 | -19,08 | 46,75 | DECEMBER | 19.4 | SG | 18 | - | - | - | - | - | **-** |
| 3472 | - | 2013 | Madagascar | AEZ9 | S73 | 1256 | -19,08 | 46,75 | DECEMBER | 19.4 | SG | 18 | - | - | - | - | - | **-** |
| 3480 | - | 2013 | Madagascar | AEZ9 | S72 | 1230 | -19,04 | 46,73 | DECEMBER | 19.4 | SG | 18 | - | - | - | - | - | **-** |
| 3484 | - | 2013 | Madagascar | AEZ9 | S72 | 1230 | -19,04 | 46,73 | DECEMBER | 19.4 | SG | - | - | - | - | - | - | **-** |
| 3507 | - | 2013 | Madagascar | AEZ7 | S52 | 1340 | -19,01 | 47,27 | DECEMBER | 21.9 | SL | 18 | - | - | - | - | - | **-** |
| 3647 | 36MAR | 2014 | Bénin | - | - | - | - | - |  | - | Sma | 31 | - | - | - | - | - | CREF-I |
| 5277 | - | 2015 | Reunion | - | - | - | - | - |  | - | ST | 31 | - | - | - | - | - | CREF-I |
|  |  |  |  |  |  |  |  |  |  |  |  |  |  |  |  |  |  |  |
| **PHYLOTYPE IIB-1** | | |  |  |  |  |  |  |  |  |  |  |  |  |  |  |  |  |
| 1 | IPO1609 | 1995 | Netherlands | - | - | - | - | - |  | - | ST | 1 | RS2-MLVA9 | 4-5-6-13-11-4-9-7-12 | MTII-6 | CCII-B | - | CREF-II |
| 41 | CIP117 | 1968 | Nigeria | - | - | - | - | - |  | - | ST | 1 | RS2-MLVA9 | 4-5-6-13-11-4-9-7-8 | MTII-9 | CCII-B | - | CREF-II |
| 147 | CFBP7029 | 2005 | Cameroon | - | - | - | - | - |  | - | SL | 1 | RS2-MLVA9 | 4-0-6-13-12-4-10-7-8 | MTII-9 | CCII-B | - | CREF-II |
| 160 | JT516 | 1993 | Reunion | - | - | - | - | - |  | - | ST | 1 | RS2-MLVA9 | 4-5-6-13-11-4-9-7-8 | MTII-44 | CCII-B | - | CREF-II |
| 256 | PSS525 | 2000 | Taiwan | - | - | - | - | - |  | - | ST | 1 | RS2-MLVA9 | 4-6-4-13-11-4-9-7-9 | MTII-26 | CCII-B | - | CREF-II |
| 336 | PO41 | - | China | - | - | - | - | - |  | - | ST | 1 | RS2-MLVA9 | 4-5-6-8-11-4-9-6-5 | MTII-34 | CCII-B | - | CREF-II |
| 371 | DGBBC1181 | - | Belgium | - | - | - | - | - |  | - | ST | 1 | RS2-MLVA9 | 4-5-5-13-11-4-9-7-8 | MTII-48 | CCII-B | - | CREF-II |
| 372 | MO7/02 | - | Mali | - | - | - | - | - |  | - | ST | 1 | RS2-MLVA9 | 4-5-5-13-12-4-9-9-8 | MTII-9 | CCII-B | - | CREF-II |
| 374 | RTG5 | - | Guinea | - | - | - | - | - |  | - | ST | 1 | RS2-MLVA9 | 4-5-6-13-11-4-9-7-8 | MTII-42 | CCII-B | - | CREF-II |
| 439 | RM | 2003 | Uruguay | - | - | - | - | - |  | - | ST | 1 | RS2-MLVA9 | 4-7-6-12-11-4-9-8-8 | MTII-29 | CCII-B | - | CREF-II |
| 449 | UW551 | 2003 | Kenya | - | - | - | - | - |  | - | P | 1 | RS2-MLVA9 | 4-0-6-13-11-4-9-8-8 | MTII-35 | CCII-B | - | CREF-II |
| 469 | JT572 | 1998 | Zimbabwe | - | - | - | - | - |  | - | ST | 1 | RS2-MLVA9 | 4-5-6-13-11-4-10-7-8 | MTII-9 | CCII-B | - | CREF-II |
| 470 | JT573 | 1998 | Rwanda | - | - | - | - | - |  | - | ST | 1 | RS2-MLVA9 | 4-5-6-13-11-4-9-7-8 | MTII-8 | CCII-B | - | CREF-II |
| 573 | LMG2300 | 1954 | Israel | - | - | - | - | - |  | - | ST | 1 | RS2-MLVA9 | 4-4-6-13-11-4-9-7-8 | MTII-18 | CCII-B | - | CREF-II |
| 575 | LMG17139 | 1963 | Cyprus | - | - | - | - | - |  | - | ST | 1 | RS2-MLVA9 | 4-5-6-13-12-4-9-7-8 | MTII-27 | CCII-B | - | CREF-II |
| 576 | LMG6973 | 1985 | Uganda | - | - | - | - | - |  | - | ST | 1 | RS2-MLVA9 | 4-5-7-13-11-4-9-7-7 | MTII-9 | CCII-B | - | CREF-II |
| 621 | PD441 | 1984 | Sweden | - | - | - | - | - |  | - | ST | 1 | RS2-MLVA9 | 4-5-6-13-11-4-9-7-8 | MTII-9 | CCII-B | - | CREF-II |
| 630 | CFBP3863 | 1995 | Maroco | - | - | - | - | - |  | - | ST | 1 | RS2-MLVA9 | 4-5-6-13-11-4-9-7-8 | MTII-22 | CCII-B | - | CREF-II |
| 631 | CFBP3583 | 1995 | Egypt | - | - | - | - | - |  | - | ST | 1 | RS2-MLVA9 | 4-4-6-13-11-4-9-7-10 | MTII-8 | CCII-B | - | CREF-II |
| 632 | CFBP4788 | 1995 | France | - | - | - | - | - |  | - | SL | 1 | RS2-MLVA9 | 4-4-6-13-11-4-9-7-8 | MTII-36 | CCII-B | - | CREF-II |
| 633 | CFBP4787 | 1995 | Portugal | - | - | - | - | - |  | - | ST | 1 | RS2-MLVA9 | 4-5-6-13-11-4-2-7-8 | MTII-9 | CCII-B | - | CREF-II |
| 652 | LNPV27.43 | 2003 | Guadeloupe | - | - | - | - | - |  | - | A | 1 | RS2-MLVA9 | 4-5-6-13-11-4-9-7-8 | MTII-39 | CCII-B | - | CREF-II |
| 680 | CFBP1417 | 1965 | Australia | - | - | - | - | - |  | - | ST | 1 | RS2-MLVA9 | 4-5-6-14-11-4-9-8-8 | MTII-33 | CCII-B | - | CREF-II |
| 682 | CFBP1810 | 1976 | Reunion | - | - | - | - | - |  | - | ST | 1 | RS2-MLVA9 | 4-5-4-13-11-4-9-7-8 | MTII-49 | CCII-B | - | CREF-II |
| 704 | CFBP7101 | 1996 | Spain | - | - | - | - | - |  | - | ST | 1 | RS2-MLVA9 | 4-5-6-13-11-4-9-7-8 | MTII-9 | CCII-B | - | CREF-II |
| 867 | CFBP4585 | 1995 | Turkey | - | - | - | - | - |  | - | ST | 1 | RS2-MLVA9 | 4-5-6-13-11-4-9-7-8 | MTII-9 | CCII-B | - | CREF-II |
| 870 | CFBP4588 | 1996 | United Kingdom | - | - | - | - | - |  | - | ST | 1 | RS2-MLVA9 | 4-5-6-13-11-4-9-7-8 | MTII-9 | CCII-B | - | CREF-II |
| 891 | CFBP4609 | 1997 | Slovenia | - | - | - | - | - |  | - | ST | 1 | RS2-MLVA9 | 4-7-6-13-11-5-10-7-8 | MTII-43 | CCII-B | - | CREF-II |
| 903 | JS925 | - | Sri Lanka | - | - | - | - | - |  | - | ST | 1 | RS2-MLVA9 | 4-5-6-13-12-4-9-7-8 | MTII-18 | CCII-B | - | CREF-II |
| 906 | JS926 | - | India | - | - | - | - | - |  |  | ST | 1 | RS2-MLVA9 | 4-4-5-13-11-4-9-7-8 | MTII-30 | CCII-B | - | CREF-II |
| 2243 | - | 2013 | Madagascar | AEZ1 | S43 | 1605 | -20,05 | 47,07 | AVRIL | 17.7 | S | 1 | - | - | - | - | - | CSEQ |
| 2244 | - | 2013 | Madagascar | AEZ1 | S43 | 1605 | -20,05 | 47,07 | AVRIL | 17.7 | S | 1 | RS2-MLVA9 | 4-6-6-13-11-4-9-7-10 | MTII-3 | CCII-A | CLII-2 | CSEQ, CMGII |
| 2245 | - | 2013 | Madagascar | AEZ1 | S43 | 1605 | -20,05 | 47,07 | AVRIL | 17.7 | S | 1 | - | - | - | - | - | CSEQ |
| 2246 | - | 2013 | Madagascar | AEZ1 | S43 | 1605 | -20,05 | 47,07 | AVRIL | 17.7 | S | 1 | - | - | - | - | - | CSEQ |
| 2247 | - | 2013 | Madagascar | AEZ1 | S43 | 1605 | -20,05 | 47,07 | AVRIL | 17.7 | S | 1 | - | - | - | - | - | CSEQ |
| 2248 | - | 2013 | Madagascar | AEZ1 | S43 | 1605 | -20,05 | 47,07 | AVRIL | 17.7 | S | 1 | - | - | - | - | - | CSEQ |
| 2249 | - | 2013 | Madagascar | AEZ1 | S43 | 1605 | -20,05 | 47,07 | AVRIL | 17.7 | S | 1 | - | - | - | - | - | CSEQ |
| 2250 | - | 2013 | Madagascar | AEZ1 | S43 | 1605 | -20,05 | 47,07 | AVRIL | 17.7 | S | 1 | - | - | - | - | - | CSEQ |
| 2251 | - | 2013 | Madagascar | AEZ1 | S43 | 1605 | -20,05 | 47,07 | AVRIL | 17.7 | S | 1 | RS2-MLVA9 | 4-5-6-13-11-4-9-7-9 | MTII-14 | CCII-A | CLII-2 | CSEQ, CMGII |
| 2252 | - | 2013 | Madagascar | AEZ1 | S43 | 1605 | -20,05 | 47,07 | AVRIL | 17.7 | S | 1 | - | - | - | - | - | CSEQ |
| 2253 | - | 2013 | Madagascar | AEZ1 | S43 | 1605 | -20,05 | 47,07 | AVRIL | 17.7 | S | 1 | - | - | - | - | - | CSEQ |
| 2254 | - | 2013 | Madagascar | AEZ1 | S38 | 1605 | -20,05 | 47,07 | AVRIL | 17.7 | S | 1 | - | - | - | - | - | CSEQ |
| 2255 | - | 2013 | Madagascar | AEZ1 | S38 | 1605 | -20,05 | 47,07 | AVRIL | 17.7 | S | 1 | - | - | - | - | - | CSEQ |
| 2256 | - | 2013 | Madagascar | AEZ1 | S38 | 1605 | -20,05 | 47,07 | AVRIL | 17.7 | S | 1 | RS2-MLVA9 | 4-6-6-13-11-4-9-7-10 | MTII-3 | CCII-A | CLII-2 | CSEQ, CMGII |
| 2257 | - | 2013 | Madagascar | AEZ1 | S38 | 1605 | -20,05 | 47,07 | AVRIL | 17.7 | S | 1 | - | - | - | - | - | CSEQ |
| 2258 | - | 2013 | Madagascar | AEZ1 | S38 | 1605 | -20,05 | 47,07 | AVRIL | 17.7 | S | 1 | - | - | - | - | - | CSEQ |
| 2259 | - | 2013 | Madagascar | AEZ1 | S38 | 1605 | -20,05 | 47,07 | AVRIL | 17.7 | S | 1 | - | - | - | - | - | CSEQ |
| 2260 | - | 2013 | Madagascar | AEZ1 | S38 | 1605 | -20,05 | 47,07 | AVRIL | 17.7 | S | 1 | - | - | - | - | - | CSEQ |
| 2261 | - | 2013 | Madagascar | AEZ1 | S38 | 1605 | -20,05 | 47,07 | AVRIL | 17.7 | S | 1 | - | - | - | - | - | CSEQ |
| 2262 | - | 2013 | Madagascar | AEZ1 | S38 | 1605 | -20,05 | 47,07 | AVRIL | 17.7 | S | 1 | - | - | - | - | - | CSEQ |
| 2263 | - | 2013 | Madagascar | AEZ1 | S38 | 1605 | -20,05 | 47,07 | AVRIL | 17.7 | S | 1 | RS2-MLVA9 | 4-7-6-13-11-4-9-7-10 | MTII-5 | CCII-A | CLII-2 | CSEQ, CMGII |
| 2264 | - | 2013 | Madagascar | AEZ1 | S38 | 1605 | -20,05 | 47,07 | AVRIL | 17.7 | S | 1 | - | - | - | - | - | CSEQ |
| 2265 | - | 2013 | Madagascar | AEZ1 | S26 | 1650 | -19,80 | 47,52 | AVRIL | 17.7 | ST | 1 | - | - | - | - | - | CSEQ |
| 2266 | - | 2013 | Madagascar | AEZ1 | S26 | 1650 | -20,05 | 47,07 | AVRIL | 17.7 | ST | 1 | - | - | - | - | - | CSEQ |
| 2267 | - | 2013 | Madagascar | AEZ1 | S26 | 1650 | -19,80 | 47,52 | AVRIL | 17.7 | ST | 1 | RS2-MLVA9 | 4-6-6-13-11-4-9-7-10 | MTII-3 | CCII-A | CLII-2 | CSEQ, CMGII |
| 2268 | - | 2013 | Madagascar | AEZ1 | S26 | 1650 | -19,80 | 47,52 | AVRIL | 17.7 | ST | 1 | RS2-MLVA9 | 4-6-6-13-11-4-9-7-8 | MTII-13 | CCII-A | CLII-2 | CSEQ, CMGII |
| 2269 | - | 2013 | Madagascar | AEZ1 | S26 | 1650 | -19,80 | 47,52 | AVRIL | 17.7 | ST | 1 | - | - | - | - | - | CSEQ |
| 2270 | - | 2013 | Madagascar | AEZ1 | S26 | 1650 | -19,80 | 47,52 | AVRIL | 17.7 | ST | 1 | - | - | - | - | - | CSEQ |
| 2271 | - | 2013 | Madagascar | AEZ1 | S26 | 1650 | -19,80 | 47,52 | AVRIL | 17.7 | ST | 1 | - | - | - | - | - | CSEQ |
| 2272 | - | 2013 | Madagascar | AEZ1 | S26 | 1650 | -19,80 | 47,52 | AVRIL | 17.7 | ST | 1 | RS2-MLVA9 | 4-6-6-13-11-4-9-7-8 | MTII-13 | CCII-A | CLII-2 | CSEQ, CMGII |
| 2273 | - | 2013 | Madagascar | AEZ1 | S43 | 1605 | -20,05 | 47,07 | AVRIL | 17.7 | S | 1 | - | - | - | - | - | CSEQ |
| 2274 | - | 2013 | Madagascar | AEZ1 | S43 | 1605 | -20,05 | 47,07 | AVRIL | 17.7 | S | 1 | - | - | - | - | - | CSEQ |
| 2275 | - | 2013 | Madagascar | AEZ2 | S61 | 1609 | -19,98 | 47,04 | AVRIL | 17.5 | ST | - | RS2-MLVA9 | 4-6-6-13-11-4-9-7-9 | MTII-15 | CCII-A | CLII-2 | CMGII |
| 2277 | - | 2013 | Madagascar | AEZ1 | S36 | 1665 | -19,84 | 47,29 | AVRIL | 17.7 | ST | - | - | - | - | - | - | - |
| 2282 | - | 2013 | Madagascar | AEZ1 | S36 | 1665 | -19,84 | 47,29 | AVRIL | 17.7 | ST | - | - | - | - | - | - | - |
| 2283 | - | 2013 | Madagascar | AEZ1 | S36 | 1665 | -19,84 | 47,29 | AVRIL | 17.7 | ST | - | - | - | - | - | - | - |
| 2284 | - | 2013 | Madagascar | AEZ1 | S36 | 1665 | -19,90 | 47,27 | AVRIL | 17.7 | ST | - | RS2-MLVA9 | 1-5-6-13-11-1-9-7-8 | MTII-7 | CCII-A | CLII-1 | CMGII |
| 2286 | - | 2013 | Madagascar | AEZ1 | S35 | 1657 | -19,93 | 47,27 | AVRIL | 17.7 | ST | - | - | - | - | - | - | - |
| 2295 | - | 2013 | Madagascar | AEZ1 | S35 | 1657 | -19,93 | 47,27 | AVRIL | 17.7 | ST | - | - | - | - | - | - | - |
| 2297 | - | 2013 | Madagascar | AEZ1 | S35 | 1657 | -19,93 | 47,27 | AVRIL | 17.7 | ST | - | - | - | - | - | - | - |
| 2299 | - | 2013 | Madagascar | AEZ1 | S35 | 1657 | -19,93 | 47,27 | AVRIL | 17.7 | ST | - | - | - | - | - | - | - |
| 2300 | - | 2013 | Madagascar | AEZ1 | S35 | 1657 | -19,93 | 47,27 | AVRIL | 17.7 | ST | - | RS2-MLVA9 | 4-6-6-13-11-4-9-7-9 | MTII-15 | CCII-A | CLII-2 | CMGII |
| 2302 | - | 2013 | Madagascar | AEZ1 | S35 | 1657 | -19,93 | 47,27 | AVRIL | 17.7 | ST | - | - | - | - | - | - | - |
| 2303 | - | 2013 | Madagascar | AEZ1 | S35 | 1657 | -19,93 | 47,27 | AVRIL | 17.7 | ST | - | - | - | - | - | - | - |
| 2307 | - | 2013 | Madagascar | AEZ2 | S61 | 1609 | -19,98 | 47,04 | AVRIL | 18.3 | ST | - | RS2-MLVA9 | 4-6-6-13-11-4-9-7-10 | MTII-3 | CCII-A | CLII-2 | CMGII |
| 2313 | - | 2013 | Madagascar | AEZ1 | S37 | 1678 | -19,73 | 47,27 | AVRIL | 17.7 | ST | - | - | - | - | - | - | - |
| 2314 | - | 2013 | Madagascar | AEZ1 | S37 | 1678 | -19,73 | 47,27 | AVRIL | 17.7 | ST | - | RS2-MLVA9 | 4-5-6-13-11-4-9-7-8 | MTII-9 | CCII-A | CLII-1 | CMGII |
| 2315 | - | 2013 | Madagascar | AEZ1 | S37 | 1678 | -19,73 | 47,27 | AVRIL | 17.7 | ST | - | - | - | - | - | - | - |
| 2316 | - | 2013 | Madagascar | AEZ1 | S37 | 1678 | -19,73 | 47,27 | AVRIL | 17.7 | ST | 1 | RS2-MLVA9 | 4-5-6-14-11-4-9-7-8 | MTII-11 | CCII-A | CLII-1 | CSEQ, CMGII |
| 2317 | - | 2013 | Madagascar | AEZ1 | S37 | 1678 | -19,73 | 47,27 | AVRIL | 17.7 | ST | - | - | - | - | - | - | - |
| 2319 | - | 2013 | Madagascar | AEZ1 | S33 | 1660 | -19,87 | 47,21 | AVRIL | 17.7 | ST | - | - | - | - | - | - | - |
| 2321 | - | 2013 | Madagascar | AEZ1 | S33 | 1660 | -19,87 | 47,21 | AVRIL | 17.7 | ST | - | - | - | - | - | - | - |
| 2323 | - | 2013 | Madagascar | AEZ2 | S61 | 1609 | -19,98 | 47,04 | AVRIL | 18.3 | ST | - | - | - | - | - | - | - |
| 2324 | - | 2013 | Madagascar | AEZ2 | S61 | 1609 | -19,98 | 47,04 | AVRIL | 18.3 | ST | - | - | - | - | - | - | - |
| 2325 | - | 2013 | Madagascar | AEZ1 | S33 | 1660 | -19,87 | 47,21 | AVRIL | 17.7 | ST | - | RS2-MLVA9 | 4-5-6-13-11-4-9-7-8 | MTII-9 | CCII-A | CLII-1 | CMGII |
| 2326 | - | 2013 | Madagascar | AEZ1 | S33 | 1660 | -19,87 | 47,21 | AVRIL | 17.7 | ST | - | - | - | - | - | - | - |
| 2328 | - | 2013 | Madagascar | AEZ1 | S33 | 1660 | -19,87 | 47,21 | AVRIL | 17.7 | ST | - | - | - | - | - | - | - |
| 2331 | - | 2013 | Madagascar | AEZ2 | S61 | 1609 | -19,98 | 47,04 | AVRIL | 18.3 | ST | - | - | - | - | - | - | - |
| 2332 | - | 2013 | Madagascar | AEZ1 | S33 | 1660 | -19,87 | 47,21 | AVRIL | 17.7 | ST | - | - | - | - | - | - | **-** |
| 2333 | - | 2013 | Madagascar | AEZ1 | S33 | 1660 | -19,87 | 47,21 | AVRIL | 17.7 | ST | - | - | - | - | - | - | **-** |
| 2336 | - | 2013 | Madagascar | AEZ2 | S61 | 1609 | -19,98 | 47,04 | AVRIL | 18.3 | ST | - | - | - | - | - | - | **-** |
| 2337 | - | 2013 | Madagascar | AEZ1 | S33 | 1660 | -19,87 | 47,21 | AVRIL | 17.7 | ST | - | - | - | - | - | - | **-** |
| 2338 | - | 2013 | Madagascar | AEZ1 | S33 | 1660 | -19,87 | 47,21 | AVRIL | 17.7 | ST | - | RS2-MLVA9 | 4-6-6-13-11-4-9-7-8 | MTII-13 | CCII-A | CLII-1 | CMGII |
| 2339 | - | 2013 | Madagascar | AEZ1 | S33 | 1660 | -19,87 | 47,21 | AVRIL | 17.7 | ST | - | - | - | - | - | - | **-** |
| 2341 | - | 2013 | Madagascar | AEZ2 | S61 | 1609 | -19,98 | 47,04 | AVRIL | 18.3 | ST | - | - | - | - | - | - | **-** |
| 2342 | - | 2013 | Madagascar | AEZ1 | S34 | 1663 | -19,86 | 47,22 | AVRIL | 17.7 | ST | - | - | - | - | - | - | **-** |
| 2343 | - | 2013 | Madagascar | AEZ1 | S34 | 1663 | -19,86 | 47,22 | AVRIL | 17.7 | ST | - | - | - | - | - | - | **-** |
| 2346 | - | 2013 | Madagascar | AEZ1 | S34 | 1663 | -19,86 | 47,22 | AVRIL | 17.7 | ST | - | - | - | - | - | - | **-** |
| 2348 | - | 2013 | Madagascar | AEZ1 | S34 | 1663 | -19,86 | 47,22 | AVRIL | 17.7 | ST | - | - | - | - | - | - | **-** |
| 2350 | - | 2013 | Madagascar | AEZ1 | S34 | 1663 | -19,86 | 47,22 | AVRIL | 17.7 | ST | - | - | - | - | - | - | **-** |
| 2351 | - | 2013 | Madagascar | AEZ1 | S34 | 1663 | -19,86 | 47,22 | AVRIL | 17.7 | ST | - | RS2-MLVA9 | 4-4-6-13-11-4-9-7-8 | MTII-8 | CCII-A | CLII-2 | CMGII |
| 2353 | - | 2013 | Madagascar | AEZ1 | S34 | 1663 | -19,86 | 47,22 | AVRIL | 17.7 | ST | - | - | - | - | - | - | **-** |
| 2356 | - | 2013 | Madagascar | AEZ1 | S34 | 1663 | -19,86 | 47,22 | AVRIL | 17.7 | ST | - | - | - | - | - | - | **-** |
| 2359 | - | 2013 | Madagascar | AEZ1 | S34 | 1663 | -19,86 | 47,22 | AVRIL | 17.7 | ST | - | - | - | - | - | - | **-** |
| 2360 | - | 2013 | Madagascar | AEZ1 | S34 | 1663 | -19,86 | 47,22 | AVRIL | 17.7 | ST | - | RS2-MLVA9 | 4-6-6-13-11-4-9-7-10 | MTII-3 | CCII-A | CLII-2 | CMGII |
| 2361 | - | 2013 | Madagascar | AEZ1 | S34 | 1663 | -19,86 | 47,22 | AVRIL | 17.7 | ST | - | - | - | - | - | - | **-** |
| 2362 | - | 2013 | Madagascar | AEZ1 | S34 | 1663 | -19,86 | 47,22 | AVRIL | 17.7 | ST | - | - | - | - | - | - | **-** |
| 2363 | - | 2013 | Madagascar | AEZ1 | S44 | 1659 | -19,79 | 47,31 | AVRIL | 17.7 | ST | - | - | - | - | - | - | **-** |
| 2364 | - | 2013 | Madagascar | AEZ1 | S44 | 1659 | -19,79 | 47,31 | AVRIL | 17.7 | ST | - | - | - | - | - | - | **-** |
| 2365 | - | 2013 | Madagascar | AEZ1 | S44 | 1659 | -19,79 | 47,31 | AVRIL | 17.7 | ST | - | RS2-MLVA9 | 4-4-6-13-11-4-9-8-8 | MTII-31 | CCII-A | CLII-1 | CMGII |
| 2366 | - | 2013 | Madagascar | AEZ1 | S44 | 1659 | -19,79 | 47,31 | AVRIL | 17.7 | ST | - | - | - | - | - | - | **-** |
| 2367 | - | 2013 | Madagascar | AEZ2 | S57 | 1531 | -19,94 | 47,02 | AVRIL | 18.3 | ST | - | - | - | - | - | - | **-** |
| 2368 | - | 2013 | Madagascar | AEZ1 | S44 | 1659 | -19,79 | 47,31 | AVRIL | 17.7 | ST | - | - | - | - | - | - | **-** |
| 2369 | - | 2013 | Madagascar | AEZ1 | S44 | 1659 | -19,79 | 47,31 | AVRIL | 17.7 | ST | - | - | - | - | - | - | **-** |
| 2371 | - | 2013 | Madagascar | AEZ2 | S61 | 1609 | -19,98 | 47,04 | AVRIL | 18.3 | ST | - | - | - | - | - | - | **-** |
| 2379 | - | 2013 | Madagascar | AEZ1 | S44 | 1659 | -19,79 | 47,31 | AVRIL | 17.7 | ST | - | - | - | - | - | - | **-** |
| 2380 | - | 2013 | Madagascar | AEZ1 | S44 | 1659 | -19,79 | 47,31 | AVRIL | 17.7 | ST | - | - | - | - | - | - | **-** |
| 2381 | - | 2013 | Madagascar | AEZ1 | S44 | 1659 | -19,79 | 47,31 | AVRIL | 17.7 | ST | - | - | - | - | - | - | **-** |
| 2382 | - | 2013 | Madagascar | AEZ1 | S44 | 1659 | -19,79 | 47,31 | AVRIL | 17.7 | ST | 1 | RS2-MLVA9 | 4-4-6-13-11-4-9-7-8 | MTII-8 | CCII-A | CLII-1 | CSEQ, CMGII |
| 2384 | - | 2013 | Madagascar | AEZ1 | S44 | 1659 | -19,79 | 47,31 | AVRIL | 17.7 | ST | - | - | - | - | - | - | **-** |
| 2385 | - | 2013 | Madagascar | AEZ1 | S44 | 1659 | -19,79 | 47,31 | AVRIL | 17.7 | ST | - | - | - | - | - | - | **-** |
| 2387 | - | 2013 | Madagascar | AEZ2 | S59 | 1605 | -19,94 | 47,08 | AVRIL | 18.3 | ST | - | - | - | - | - | - | **-** |
| 2388 | - | 2013 | Madagascar | AEZ1 | S44 | 1659 | -19,79 | 47,31 | AVRIL | 17.7 | ST | - | - | - | - | - | - | **-** |
| 2390 | - | 2013 | Madagascar | AEZ1 | S44 | 1659 | -19,79 | 47,31 | AVRIL | 17.7 | ST | - | - | - | - | - | - | **-** |
| 2391 | - | 2013 | Madagascar | AEZ2 | S63 | 1611 | -19,98 | 47,12 | AVRIL | 18.3 | ST | - | RS2-MLVA9 | 4-6-6-13-11-4-9-7-10 | MTII-3 | CCII-A | CLII-2 | CMGII |
| 2392 | - | 2013 | Madagascar | AEZ2 | S63 | 1611 | -19,98 | 47,12 | AVRIL | 18.3 | ST | - | RS2-MLVA9 | 4-6-6-13-11-4-9-7-10 | MTII-3 | CCII-A | CLII-2 | CMGII |
| 2393 | - | 2013 | Madagascar | AEZ2 | S63 | 1611 | -19,98 | 47,12 | AVRIL | 18.3 | ST | - | RS2-MLVA9 | 4-6-6-13-11-4-9-7-10 | MTII-3 | CCII-A | CLII-2 | CMGII |
| 2394 | - | 2013 | Madagascar | AEZ2 | S63 | 1611 | -19,98 | 47,12 | AVRIL | 18.3 | ST | - | RS2-MLVA9 | 4-6-6-13-11-4-9-7-10 | MTII-3 | CCII-A | CLII-2 | CMGII |
| 2395 | - | 2013 | Madagascar | AEZ2 | S63 | 1611 | -19,98 | 47,12 | AVRIL | 18.3 | ST | - | RS2-MLVA9 | 4-6-6-13-11-4-9-7-10 | MTII-3 | CCII-A | CLII-2 | CMGII |
| 2396 | - | 2013 | Madagascar | AEZ2 | S63 | 1611 | -19,98 | 47,12 | AVRIL | 18.3 | ST | - | RS2-MLVA9 | 4-6-6-13-11-4-9-7-10 | MTII-3 | CCII-A | CLII-2 | CMGII |
| 2397 | - | 2013 | Madagascar | AEZ2 | S63 | 1611 | -19,98 | 47,12 | AVRIL | 18.3 | ST | - | RS2-MLVA9 | 4-6-6-13-11-4-9-7-10 | MTII-3 | CCII-A | CLII-2 | CMGII |
| 2398 | - | 2013 | Madagascar | AEZ2 | S63 | 1611 | -19,98 | 47,12 | AVRIL | 18.3 | ST | - | RS2-MLVA9 | 4-5-6-13-11-4-9-7-10 | MTII-2 | CCII-A | CLII-2 | CMGII |
| 2399 | - | 2013 | Madagascar | AEZ2 | S63 | 1611 | -19,98 | 47,12 | AVRIL | 18.3 | ST | 1 | RS2-MLVA9 | 4-6-6-13-11-4-9-7-10 | MTII-3 | CCII-A | CLII-2 | CSEQ, CMGII |
| 2400 | - | 2013 | Madagascar | AEZ2 | S63 | 1611 | -19,98 | 47,12 | AVRIL | 18.3 | ST | - | RS2-MLVA9 | 4-6-6-13-11-4-9-7-10 | MTII-3 | CCII-A | CLII-2 | CMGII |
| 2401 | - | 2013 | Madagascar | AEZ2 | S63 | 1611 | -19,98 | 47,12 | AVRIL | 18.3 | ST | - | RS2-MLVA9 | 4-6-6-13-11-4-9-7-10 | MTII-3 | CCII-A | CLII-2 | CMGII |
| 2402 | - | 2013 | Madagascar | AEZ2 | S63 | 1611 | -19,98 | 47,12 | AVRIL | 18.3 | ST | - | RS2-MLVA9 | 4-6-6-13-11-4-9-7-10 | MTII-3 | CCII-A | CLII-2 | CMGII |
| 2403 | - | 2013 | Madagascar | AEZ2 | S63 | 1611 | -19,98 | 47,12 | AVRIL | 18.3 | ST | - | RS2-MLVA9 | 4-6-6-13-11-4-9-7-10 | MTII-3 | CCII-A | CLII-2 | CMGII |
| 2404 | - | 2013 | Madagascar | AEZ2 | S63 | 1611 | -19,98 | 47,12 | AVRIL | 18.3 | ST | - | RS2-MLVA9 | 4-6-6-13-11-4-9-7-10 | MTII-3 | CCII-A | CLII-2 | CMGII |
| 2405 | - | 2013 | Madagascar | AEZ2 | S63 | 1611 | -19,98 | 47,12 | AVRIL | 18.3 | ST | - | RS2-MLVA9 | 4-6-6-13-11-4-9-7-10 | MTII-3 | CCII-A | CLII-2 | CMGII |
| 2406 | - | 2013 | Madagascar | AEZ2 | S63 | 1611 | -19,98 | 47,12 | AVRIL | 18.3 | ST | - | RS2-MLVA9 | 4-6-6-13-11-4-9-7-10 | MTII-3 | CCII-A | CLII-2 | CMGII |
| 2407 | - | 2013 | Madagascar | AEZ2 | S63 | 1611 | -19,98 | 47,12 | AVRIL | 18.3 | ST | - | RS2-MLVA9 | 4-6-6-13-11-4-9-7-10 | MTII-3 | CCII-A | CLII-2 | CMGII |
| 2408 | - | 2013 | Madagascar | AEZ2 | S63 | 1611 | -19,98 | 47,12 | AVRIL | 18.3 | ST | - | RS2-MLVA9 | 4-6-6-13-11-4-9-7-10 | MTII-3 | CCII-A | CLII-2 | CMGII |
| 2409 | - | 2013 | Madagascar | AEZ2 | S63 | 1611 | -19,98 | 47,12 | AVRIL | 18.3 | ST | - | RS2-MLVA9 | 4-6-6-13-11-4-9-7-10 | MTII-3 | CCII-A | CLII-2 | CMGII |
| 2410 | - | 2013 | Madagascar | AEZ2 | S63 | 1611 | -19,98 | 47,12 | AVRIL | 18.3 | ST | - | RS2-MLVA9 | 4-6-6-13-11-4-9-7-10 | MTII-3 | CCII-A | CLII-2 | CMGII |
| 2411 | - | 2013 | Madagascar | AEZ2 | S62 | 1629 | -19,89 | 47,13 | AVRIL | 18.3 | ST | - | RS2-MLVA9 | 1-6-6-13-11-1-9-7-10 | MTII-1 | CCII-B | CLII-2 | CMGII |
| 2412 | - | 2013 | Madagascar | AEZ2 | S62 | 1629 | -19,89 | 47,13 | AVRIL | 18.3 | ST | - | RS2-MLVA9 | 1-5-6-13-11-1-9-7-8 | MTII-7 | CCII-A | CLII-2 | CMGII |
| 2413 | - | 2013 | Madagascar | AEZ2 | S62 | 1629 | -19,89 | 47,13 | AVRIL | 18.3 | ST | - | RS2-MLVA9 | 1-6-6-13-11-1-9-7-10 | MTII-1 | CCII-A | CLII-2 | CMGII |
| 2414 | - | 2013 | Madagascar | AEZ2 | S62 | 1629 | -19,89 | 47,13 | AVRIL | 18.3 | ST | 1 | RS2-MLVA9 | 4-6-6-13-11-4-9-7-10 | MTII-3 | CCII-A | CLII-2 | CSEQ, CMGII |
| 2415 | - | 2013 | Madagascar | AEZ2 | S62 | 1629 | -19,89 | 47,13 | AVRIL | 18.3 | ST | - | RS2-MLVA9 | 4-6-6-13-11-4-9-7-10 | MTII-3 | CCII-A | CLII-2 | CMGII |
| 2416 | - | 2013 | Madagascar | AEZ2 | S62 | 1629 | -19,89 | 47,13 | AVRIL | 18.3 | ST | - | RS2-MLVA9 | 4-6-6-13-11-4-9-7-9 | MTII-15 | CCII-A | CLII-2 | CMGII |
| 2417 | - | 2013 | Madagascar | AEZ2 | S62 | 1629 | -19,89 | 47,13 | AVRIL | 18.3 | ST | - | RS2-MLVA9 | 4-6-6-13-11-4-9-7-9 | MTII-15 | CCII-A | CLII-2 | CMGII |
| 2418 | - | 2013 | Madagascar | AEZ2 | S62 | 1629 | -19,89 | 47,13 | AVRIL | 18.3 | ST | - | RS2-MLVA9 | 4-5-6-13-11-4-9-8-8 | MTII-10 | CCII-A | CLII-2 | CMGII |
| 2419 | - | 2013 | Madagascar | AEZ2 | S62 | 1629 | -19,89 | 47,13 | AVRIL | 18.3 | ST | - | - | - | - | - | - | **-** |
| 2420 | - | 2013 | Madagascar | AEZ2 | S62 | 1629 | -19,89 | 47,13 | AVRIL | 18.3 | ST | - | RS2-MLVA9 | 4-6-6-13-11-4-9-7-10 | MTII-3 | CCII-A | CLII-2 | CMGII |
| 2421 | - | 2013 | Madagascar | AEZ2 | S62 | 1629 | -19,89 | 47,13 | AVRIL | 18.3 | ST | - | RS2-MLVA9 | 4-5-6-13-11-4-9-8-8 | MTII-10 | CCII-A | CLII-2 | CMGII |
| 2422 | - | 2013 | Madagascar | AEZ2 | S62 | 1629 | -19,89 | 47,13 | AVRIL | 18.3 | ST | - | RS2-MLVA9 | 1-6-6-11-11-1-9-7-10 | MTII-20 | CCII-B | CLII-2 | CMGII |
| 2423 | - | 2013 | Madagascar | AEZ2 | S62 | 1629 | -19,89 | 47,13 | AVRIL | 18.3 | ST | - | RS2-MLVA9 | 4-6-6-13-11-4-9-7-10 | MTII-3 | CCII-A | CLII-2 | CMGII |
| 2424 | - | 2013 | Madagascar | AEZ2 | S62 | 1629 | -19,89 | 47,13 | AVRIL | 18.3 | ST | - | RS2-MLVA9 | 4-5-6-13-11-4-9-7-10 | MTII-2 | CCII-A | CLII-2 | CMGII |
| 2425 | - | 2013 | Madagascar | AEZ2 | S62 | 1629 | -19,89 | 47,13 | AVRIL | 18.3 | ST | - | RS2-MLVA9 | 4-6-6-13-11-4-9-7-10 | MTII-3 | CCII-A | CLII-2 | CMGII |
| 2426 | - | 2013 | Madagascar | AEZ2 | S62 | 1629 | -19,89 | 47,13 | AVRIL | 18.3 | ST | - | RS2-MLVA9 | 4-6-6-13-11-4-9-7-10 | MTII-3 | CCII-A | CLII-2 | CMGII |
| 2427 | - | 2013 | Madagascar | AEZ2 | S62 | 1629 | -19,89 | 47,13 | AVRIL | 18.3 | ST | - | RS2-MLVA9 | 1-6-6-13-11-1-9-7-10 | MTII-1 | CCII-A | CLII-2 | CMGII |
| 2428 | - | 2013 | Madagascar | AEZ2 | S62 | 1629 | -19,89 | 47,13 | AVRIL | 18.3 | ST | - | RS2-MLVA9 | 4-6-6-13-11-4-9-7-10 | MTII-3 | CCII-A | CLII-2 | CMGII |
| 2429 | - | 2013 | Madagascar | AEZ2 | S62 | 1629 | -19,89 | 47,13 | AVRIL | 18.3 | ST | - | RS2-MLVA9 | 4-6-6-13-11-4-9-7-10 | MTII-3 | CCII-A | CLII-2 | CMGII |
| 2430 | - | 2013 | Madagascar | AEZ2 | S62 | 1629 | -19,89 | 47,13 | AVRIL | 18.3 | ST | - | RS2-MLVA9 | 4-6-6-13-11-4-9-7-10 | MTII-3 | CCII-A | CLII-2 | CMGII |
| 2431 | - | 2013 | Madagascar | AEZ2 | S62 | 1629 | -19,89 | 47,13 | AVRIL | 18.3 | ST | - | RS2-MLVA9 | 4-6-6-13-11-4-9-7-10 | MTII-3 | CCII-A | CLII-2 | CMGII |
| 2432 | - | 2013 | Madagascar | AEZ2 | S62 | 1629 | -19,89 | 47,13 | AVRIL | 18.3 | ST | - | RS2-MLVA9 | 4-6-6-13-11-4-9-7-10 | MTII-3 | CCII-A | CLII-2 | CMGII |
| 2433 | - | 2013 | Madagascar | AEZ2 | S62 | 1629 | -19,89 | 47,13 | AVRIL | 18.3 | ST | - | RS2-MLVA9 | 4-6-6-13-11-4-9-7-10 | MTII-3 | CCII-A | CLII-2 | CMGII |
| 2434 | - | 2013 | Madagascar | AEZ2 | S64 | 1618 | -19,89 | 46,90 | AVRIL | 18.3 | ST | - | - | - | - | - | - | **-** |
| 2435 | - | 2013 | Madagascar | AEZ2 | S64 | 1618 | -19,89 | 46,90 | AVRIL | 18.3 | ST | - | - | - | - | - | - | **-** |
| 2436 | - | 2013 | Madagascar | AEZ2 | S64 | 1618 | -19,89 | 46,90 | AVRIL | 18.3 | ST | - | - | - | - | - | - | **-** |
| 2437 | - | 2013 | Madagascar | AEZ2 | S64 | 1618 | -19,89 | 46,90 | AVRIL | 18.3 | ST | - | - | - | - | - | - | **-** |
| 2438 | - | 2013 | Madagascar | AEZ2 | S64 | 1618 | -19,89 | 46,90 | AVRIL | 18.3 | ST | - | - | - | - | - | - | **-** |
| 2439 | - | 2013 | Madagascar | AEZ2 | S64 | 1618 | -19,89 | 46,90 | AVRIL | 18.3 | ST | - | - | - | - | - | - | **-** |
| 2440 | - | 2013 | Madagascar | AEZ2 | S64 | 1618 | -19,89 | 46,90 | AVRIL | 18.3 | ST | - | - | - | - | - | - | **-** |
| 2441 | - | 2013 | Madagascar | AEZ2 | S64 | 1618 | -19,89 | 46,90 | AVRIL | 18.3 | ST | - | - | - | - | - | - | **-** |
| 2442 | - | 2013 | Madagascar | AEZ2 | S64 | 1618 | -19,89 | 46,90 | AVRIL | 18.3 | ST | - | - | - | - | - | - | **-** |
| 2443 | - | 2013 | Madagascar | AEZ2 | S64 | 1618 | -19,89 | 46,90 | AVRIL | 18.3 | ST | - | - | - | - | - | - | **-** |
| 2444 | - | 2013 | Madagascar | AEZ2 | S64 | 1618 | -19,89 | 46,90 | AVRIL | 18.3 | ST | - | - | - | - | - | - | **-** |
| 2445 | - | 2013 | Madagascar | AEZ2 | S64 | 1618 | -19,89 | 46,90 | AVRIL | 18.3 | ST | - | - | - | - | - | - | **-** |
| 2446 | - | 2013 | Madagascar | AEZ2 | S64 | 1618 | -19,89 | 46,90 | AVRIL | 18.3 | ST | - | - | - | - | - | - | **-** |
| 2447 | - | 2013 | Madagascar | AEZ2 | S64 | 1618 | -19,89 | 46,90 | AVRIL | 18.3 | ST | - | - | - | - | - | - | **-** |
| 2448 | - | 2013 | Madagascar | AEZ2 | S64 | 1618 | -19,89 | 46,90 | AVRIL | 18.3 | ST | - | - | - | - | - | - | **-** |
| 2449 | - | 2013 | Madagascar | AEZ2 | S64 | 1618 | -19,89 | 46,90 | AVRIL | 18.3 | ST | - | - | - | - | - | - | **-** |
| 2450 | - | 2013 | Madagascar | AEZ2 | S64 | 1618 | -19,89 | 46,90 | AVRIL | 18.3 | ST | - | - | - | - | - | - | **-** |
| 2451 | - | 2013 | Madagascar | AEZ2 | S64 | 1618 | -19,89 | 46,90 | AVRIL | 18.3 | ST | - | - | - | - | - | - | **-** |
| 2452 | - | 2013 | Madagascar | AEZ2 | S64 | 1618 | -19,89 | 46,90 | AVRIL | 18.3 | ST | - | - | - | - | - | - | **-** |
| 2453 | - | 2013 | Madagascar | AEZ2 | S64 | 1618 | -19,89 | 46,90 | AVRIL | 18.3 | ST | - | - | - | - | - | - | **-** |
| 2454 | - | 2013 | Madagascar | AEZ2 | S64 | 1618 | -19,89 | 46,90 | AVRIL | 18.3 | ST | - | - | - | - | - | - | **-** |
| 2455 | - | 2013 | Madagascar | AEZ2 | S64 | 1618 | -19,89 | 46,90 | AVRIL | 18.3 | ST | - | - | - | - | - | - | **-** |
| 2456 | - | 2013 | Madagascar | AEZ1 | S36 | 1665 | -19,84 | 47,29 | AVRIL | 17.7 | ST | - | RS2-MLVA9 | 4-6-6-13-11-4-9-7-10 | MTII-3 | CCII-A | CLII-2 | CMGII |
| 2457 | - | 2013 | Madagascar | AEZ1 | S36 | 1665 | -19,84 | 47,29 | AVRIL | 17.7 | ST | 1 | RS2-MLVA9 | 4-6-6-13-11-4-9-7-10 | MTII-3 | CCII-A | CLII-2 | CSEQ, CMGII |
| 2458 | - | 2013 | Madagascar | AEZ2 | S61 | 1609 | -19,98 | 47,04 | AVRIL | 18.3 | ST | - | - | - | - | - | - | **-** |
| 2459 | - | 2013 | Madagascar | AEZ2 | S61 | 1609 | -19,98 | 47,04 | AVRIL | 18.3 | ST | - | - | - | - | - | - | **-** |
| 2460 | - | 2013 | Madagascar | AEZ2 | S61 | 1609 | -19,98 | 47,04 | AVRIL | 18.3 | ST | - | - | - | - | - | - | **-** |
| 2461 | - | 2013 | Madagascar | AEZ2 | S61 | 1609 | -19,98 | 47,04 | AVRIL | 18.3 | ST | - | - | - | - | - | - | **-** |
| 2462 | - | 2013 | Madagascar | AEZ2 | S61 | 1609 | -19,98 | 47,04 | AVRIL | 18.3 | ST | - | - | - | - | - | - | **-** |
| 2463 | - | 2013 | Madagascar | AEZ2 | S61 | 1609 | -19,98 | 47,04 | AVRIL | 18.3 | ST | - | - | - | - | - | - | **-** |
| 2464 | - | 2013 | Madagascar | AEZ2 | S61 | 1609 | -19,98 | 47,04 | AVRIL | 18.3 | ST | - | - | - | - | - | - | **-** |
| 2465 | - | 2013 | Madagascar | AEZ2 | S61 | 1609 | -19,98 | 47,04 | AVRIL | 18.3 | ST | - | - | - | - | - | - | **-** |
| 2466 | - | 2013 | Madagascar | AEZ2 | S61 | 1609 | -19,98 | 47,04 | AVRIL | 18.3 | ST | - | - | - | - | - | - | **-** |
| 2467 | - | 2013 | Madagascar | AEZ2 | S61 | 1609 | -19,98 | 47,04 | AVRIL | 18.3 | ST | - | - | - | - | - | - | **-** |
| 2468 | - | 2013 | Madagascar | AEZ2 | S61 | 1609 | -19,98 | 47,04 | AVRIL | 18.3 | ST | - | - | - | - | - | - | **-** |
| 2469 | - | 2013 | Madagascar | AEZ2 | S61 | 1609 | -19,98 | 47,04 | AVRIL | 18.3 | ST | - | - | - | - | - | - | **-** |
| 2470 | - | 2013 | Madagascar | AEZ2 | S60 | 1604 | -19,90 | 47,05 | AVRIL | 18.3 | ST | - | - | - | - | - | - | **-** |
| 2471 | - | 2013 | Madagascar | AEZ2 | S60 | 1604 | -19,90 | 47,05 | AVRIL | 18.3 | ST | - | - | - | - | - | - | **-** |
| 2472 | - | 2013 | Madagascar | AEZ2 | S60 | 1604 | -19,90 | 47,05 | AVRIL | 18.3 | ST | - | - | - | - | - | - | **-** |
| 2473 | - | 2013 | Madagascar | AEZ2 | S60 | 1604 | -19,90 | 47,05 | AVRIL | 18.3 | ST | - | - | - | - | - | - | **-** |
| 2475 | - | 2013 | Madagascar | AEZ2 | S60 | 1604 | -19,90 | 47,05 | AVRIL | 18.3 | ST | - | - | - | - | - | - | **-** |
| 2476 | - | 2013 | Madagascar | AEZ2 | S60 | 1604 | -19,90 | 47,05 | AVRIL | 18.3 | ST | - | - | - | - | - | - | **-** |
| 2477 | - | 2013 | Madagascar | AEZ2 | S60 | 1604 | -19,90 | 47,05 | AVRIL | 18.3 | ST | - | RS2-MLVA9 | 4-6-6-13-11-4-9-7-10 | MTII-3 | CCII-A | CLII-2 | CMGII |
| 2478 | - | 2013 | Madagascar | AEZ2 | S60 | 1604 | -19,90 | 47,05 | AVRIL | 18.3 | ST | - | - | - | - | - | - | **-** |
| 2479 | - | 2013 | Madagascar | AEZ2 | S60 | 1604 | -19,90 | 47,05 | AVRIL | 18.3 | ST | - | - | - | - | - | - | **-** |
| 2480 | - | 2013 | Madagascar | AEZ2 | S60 | 1604 | -19,90 | 47,05 | AVRIL | 18.3 | ST | - | - | - | - | - | - | **-** |
| 2481 | - | 2013 | Madagascar | AEZ2 | S60 | 1604 | -19,90 | 47,05 | AVRIL | 18.3 | ST | - | - | - | - | - | - | **-** |
| 2482 | - | 2013 | Madagascar | AEZ2 | S60 | 1604 | -19,90 | 47,05 | AVRIL | 18.3 | ST | - | - | - | - | - | - | **-** |
| 2483 | - | 2013 | Madagascar | AEZ2 | S60 | 1604 | -19,90 | 47,05 | AVRIL | 18.3 | ST | - | - | - | - | - | - | **-** |
| 2484 | - | 2013 | Madagascar | AEZ2 | S60 | 1604 | -19,90 | 47,05 | AVRIL | 18.3 | ST | - | - | - | - | - | - | **-** |
| 2485 | - | 2013 | Madagascar | AEZ2 | S60 | 1604 | -19,90 | 47,05 | AVRIL | 18.3 | ST | - | - | - | - | - | - | **-** |
| 2486 | - | 2013 | Madagascar | AEZ2 | S60 | 1604 | -19,90 | 47,05 | AVRIL | 18.3 | ST | - | - | - | - | - | - | **-** |
| 2487 | - | 2013 | Madagascar | AEZ2 | S60 | 1604 | -19,90 | 47,05 | AVRIL | 18.3 | ST | - | - | - | - | - | - | **-** |
| 2488 | - | 2013 | Madagascar | AEZ2 | S56 | 1599 | -19,87 | 47,02 | AVRIL | 18.3 | ST | - | - | - | - | - | - | **-** |
| 2489 | - | 2013 | Madagascar | AEZ2 | S56 | 1599 | -19,87 | 47,02 | AVRIL | 18.3 | ST | - | - | - | - | - | - | **-** |
| 2490 | - | 2013 | Madagascar | AEZ2 | S56 | 1599 | -19,87 | 47,02 | AVRIL | 18.3 | ST | - | - | - | - | - | - | **-** |
| 2491 | - | 2013 | Madagascar | AEZ2 | S56 | 1599 | -19,87 | 47,02 | AVRIL | 18.3 | ST | - | - | - | - | - | - | **-** |
| 2492 | - | 2013 | Madagascar | AEZ2 | S56 | 1599 | -19,87 | 47,02 | AVRIL | 18.3 | ST | - | - | - | - | - | - | **-** |
| 2493 | - | 2013 | Madagascar | AEZ2 | S56 | 1599 | -19,87 | 47,02 | AVRIL | 18.3 | ST | - | - | - | - | - | - | **-** |
| 2494 | - | 2013 | Madagascar | AEZ2 | S56 | 1599 | -19,87 | 47,02 | AVRIL | 18.3 | ST | - | - | - | - | - | - | **-** |
| 2495 | - | 2013 | Madagascar | AEZ2 | S56 | 1599 | -19,87 | 47,02 | AVRIL | 18.3 | ST | - | - | - | - | - | - | **-** |
| 2496 | - | 2013 | Madagascar | AEZ2 | S56 | 1599 | -19,87 | 47,02 | AVRIL | 18.3 | ST | - | - | - | - | - | - | **-** |
| 2497 | - | 2013 | Madagascar | AEZ2 | S56 | 1599 | -19,87 | 47,02 | AVRIL | 18.3 | ST | - | - | - | - | - | - | **-** |
| 2498 | - | 2013 | Madagascar | AEZ2 | S56 | 1599 | -19,87 | 47,02 | AVRIL | 18.3 | ST | - | - | - | - | - | - | **-** |
| 2499 | - | 2013 | Madagascar | AEZ2 | S56 | 1599 | -19,87 | 47,02 | AVRIL | 18.3 | ST | - | - | - | - | - | - | **-** |
| 2500 | - | 2013 | Madagascar | AEZ2 | S56 | 1599 | -19,87 | 47,02 | AVRIL | 18.3 | ST | - | - | - | - | - | - | **-** |
| 2501 | - | 2013 | Madagascar | AEZ2 | S56 | 1599 | -19,87 | 47,02 | AVRIL | 18.3 | ST | - | - | - | - | - | - | **-** |
| 2502 | - | 2013 | Madagascar | AEZ2 | S56 | 1599 | -19,87 | 47,02 | AVRIL | 18.3 | SL | - | - | - | - | - | - | **-** |
| 2503 | - | 2013 | Madagascar | AEZ2 | S56 | 1599 | -19,87 | 47,02 | AVRIL | 18.3 | ST | - | - | - | - | - | - | **-** |
| 2504 | - | 2013 | Madagascar | AEZ2 | S56 | 1599 | -19,87 | 47,02 | AVRIL | 18.3 | ST | - | - | - | - | - | - | **-** |
| 2505 | - | 2013 | Madagascar | AEZ2 | S56 | 1599 | -19,87 | 47,02 | AVRIL | 18.3 | ST | - | - | - | - | - | - | **-** |
| 2506 | - | 2013 | Madagascar | AEZ2 | S56 | 1599 | -19,87 | 47,02 | AVRIL | 18.3 | ST | - | - | - | - | - | - | **-** |
| 2507 | - | 2013 | Madagascar | AEZ2 | S56 | 1599 | -19,87 | 47,02 | AVRIL | 18.3 | ST | - | - | - | - | - | - | **-** |
| 2508 | - | 2013 | Madagascar | AEZ2 | S56 | 1599 | -19,87 | 47,02 | AVRIL | 18.3 | ST | - | - | - | - | - | - | **-** |
| 2509 | - | 2013 | Madagascar | AEZ2 | S56 | 1599 | -19,87 | 47,02 | AVRIL | 18.3 | ST | - | - | - | - | - | - | **-** |
| 2511 | - | 2013 | Madagascar | AEZ2 | S59 | 1605 | -19,94 | 47,08 | AVRIL | 18.3 | ST | - | - | - | - | - | - | **-** |
| 2512 | - | 2013 | Madagascar | AEZ2 | S59 | 1605 | -19,94 | 47,08 | AVRIL | 18.3 | ST | - | - | - | - | - | - | **-** |
| 2513 | - | 2013 | Madagascar | AEZ2 | S59 | 1605 | -19,94 | 47,08 | AVRIL | 18.3 | ST | - | - | - | - | - | - | **-** |
| 2515 | - | 2013 | Madagascar | AEZ2 | S59 | 1605 | -19,94 | 47,08 | AVRIL | 18.3 | ST | - | - | - | - | - | - | **-** |
| 2516 | - | 2013 | Madagascar | AEZ2 | S59 | 1605 | -19,94 | 47,08 | AVRIL | 18.3 | ST | - | - | - | - | - | - | **-** |
| 2517 | - | 2013 | Madagascar | AEZ2 | S59 | 1605 | -19,94 | 47,08 | AVRIL | 18.3 | ST | - | - | - | - | - | - | **-** |
| 2518 | - | 2013 | Madagascar | AEZ2 | S59 | 1605 | -19,94 | 47,08 | AVRIL | 18.3 | ST | - | - | - | - | - | - | **-** |
| 2519 | - | 2013 | Madagascar | AEZ2 | S59 | 1605 | -19,94 | 47,08 | AVRIL | 18.3 | ST | - | - | - | - | - | - | **-** |
| 2520 | - | 2013 | Madagascar | AEZ2 | S59 | 1605 | -19,94 | 47,08 | AVRIL | 18.3 | ST | - | - | - | - | - | - | **-** |
| 2521 | - | 2013 | Madagascar | AEZ2 | S59 | 1605 | -19,94 | 47,08 | AVRIL | 18.3 | ST | - | - | - | - | - | - | **-** |
| 2522 | - | 2013 | Madagascar | AEZ2 | S59 | 1605 | -19,94 | 47,08 | AVRIL | 18.3 | ST | - | - | - | - | - | - | **-** |
| 2523 | - | 2013 | Madagascar | AEZ2 | S59 | 1605 | -19,94 | 47,08 | AVRIL | 18.3 | ST | - | - | - | - | - | - | **-** |
| 2524 | - | 2013 | Madagascar | AEZ2 | S58 | 1518 | -19,95 | 47,03 | AVRIL | 18.3 | ST | - | - | - | - | - | - | **-** |
| 2525 | - | 2013 | Madagascar | AEZ2 | S58 | 1518 | -19,95 | 47,03 | AVRIL | 18.3 | ST | - | - | - | - | - | - | **-** |
| 2526 | - | 2013 | Madagascar | AEZ2 | S58 | 1518 | -19,95 | 47,03 | AVRIL | 18.3 | ST | - | - | - | - | - | - | **-** |
| 2527 | - | 2013 | Madagascar | AEZ4 | S2 | 1593 | -19,45 | 47,67 | AVRIL | 17.3 | ST | - | - | - | - | - | - | **-** |
| 2528 | - | 2013 | Madagascar | AEZ4 | S2 | 1593 | -19,45 | 47,67 | AVRIL | 17.3 | ST | - | - | - | - | - | - | **-** |
| 2529 | - | 2013 | Madagascar | AEZ4 | S2 | 1593 | -19,45 | 47,67 | AVRIL | 17.3 | ST | - | RS2-MLVA9 | 4-6-6-13-11-4-10-7-8 | MTII-12 | CCII-A | CLII-1 | CMGII |
| 2530 | - | 2013 | Madagascar | AEZ4 | S2 | 1593 | -19,45 | 47,67 | AVRIL | 17.3 | ST | - | - | - | - | - | - | **-** |
| 2531 | - | 2013 | Madagascar | AEZ4 | S2 | 1593 | -19,45 | 47,67 | AVRIL | 17.3 | ST | - | - | - | - | - | - | **-** |
| 2532 | - | 2013 | Madagascar | AEZ4 | S2 | 1593 | -19,45 | 47,67 | AVRIL | 17.3 | ST | 1 | - | - | - | - | - | **-** |
| 2533 | - | 2013 | Madagascar | AEZ4 | S2 | 1593 | -19,45 | 47,67 | AVRIL | 17.3 | ST | - | - | - | - | - | - | **-** |
| 2534 | - | 2013 | Madagascar | AEZ4 | S2 | 1593 | -19,45 | 47,67 | AVRIL | 17.3 | ST | - | RS2-MLVA9 | 4-6-6-13-11-4-10-7-8 | MTII-12 | CCII-A | CLII-1 | CMGII |
| 2535 | - | 2013 | Madagascar | AEZ4 | S2 | 1593 | -19,45 | 47,67 | AVRIL | 17.3 | ST | - | - | - | - | - | - | **-** |
| 2536 | - | 2013 | Madagascar | AEZ4 | S2 | 1593 | -19,45 | 47,67 | AVRIL | 17.3 | ST | - | - | - | - | - | - | **-** |
| 2537 | - | 2013 | Madagascar | AEZ4 | S2 | 1593 | -19,45 | 47,67 | AVRIL | 17.3 | ST | - | - | - | - | - | - | **-** |
| 2538 | - | 2013 | Madagascar | AEZ4 | S2 | 1593 | -19,45 | 47,67 | AVRIL | 17.3 | ST | - | - | - | - | - | - | **-** |
| 2539 | - | 2013 | Madagascar | AEZ4 | S6 | 1594 | -19,48 | 47,40 | AVRIL | 17.3 | ST | - | RS2-MLVA9 | 4-6-6-13-11-4-9-7-10 | MTII-3 | CCII-A | CLII-2 | CMGII |
| 2540 | - | 2013 | Madagascar | AEZ4 | S6 | 1594 | -19,48 | 47,40 | AVRIL | 17.3 | ST | - | RS2-MLVA9 | 4-6-6-13-11-4-9-7-10 | MTII-3 | CCII-A | CLII-2 | CMGII |
| 2541 | - | 2013 | Madagascar | AEZ4 | S6 | 1594 | -19,48 | 47,40 | AVRIL | 17.3 | ST | - | RS2-MLVA9 | 4-6-6-13-11-4-9-7-10 | MTII-3 | CCII-A | CLII-2 | CMGII |
| 2542 | - | 2013 | Madagascar | AEZ4 | S6 | 1594 | -19,48 | 47,40 | AVRIL | 17.3 | ST | - | RS2-MLVA9 | 4-6-6-13-11-4-9-7-10 | MTII-3 | CCII-A | CLII-2 | CMGII |
| 2543 | - | 2013 | Madagascar | AEZ4 | S6 | 1594 | -19,48 | 47,40 | AVRIL | 17.3 | ST | - | RS2-MLVA9 | 4-6-6-13-11-4-9-7-10 | MTII-3 | CCII-A | CLII-2 | CMGII |
| 2544 | - | 2013 | Madagascar | AEZ4 | S6 | 1594 | -19,48 | 47,40 | AVRIL | 17.3 | ST | - | RS2-MLVA9 | 4-6-6-13-11-4-9-7-10 | MTII-3 | CCII-A | CLII-2 | CMGII |
| 2545 | - | 2013 | Madagascar | AEZ4 | S6 | 1594 | -19,48 | 47,40 | AVRIL | 17.3 | ST | - | RS2-MLVA9 | 4-6-6-13-11-4-9-7-10 | MTII-3 | CCII-A | CLII-2 | CMGII |
| 2546 | - | 2013 | Madagascar | AEZ4 | S6 | 1594 | -19,48 | 47,40 | AVRIL | 17.3 | ST | - | RS2-MLVA9 | 4-6-6-13-11-4-9-7-10 | MTII-3 | CCII-A | CLII-2 | CMGII |
| 2547 | - | 2013 | Madagascar | AEZ4 | S6 | 1594 | -19,48 | 47,40 | AVRIL | 17.3 | ST | - | RS2-MLVA9 | 4-6-6-13-11-4-9-7-10 | MTII-3 | CCII-A | CLII-2 | CMGII |
| 2548 | - | 2013 | Madagascar | AEZ4 | S6 | 1594 | -19,48 | 47,40 | AVRIL | 17.3 | ST | 1 | RS2-MLVA9 | 4-6-6-13-11-4-9-7-10 | MTII-3 | CCII-A | CLII-2 | CSEQ, CMGII |
| 2549 | - | 2013 | Madagascar | AEZ4 | S6 | 1594 | -19,48 | 47,40 | AVRIL | 17.3 | ST | - | RS2-MLVA9 | 4-6-6-13-11-4-9-7-10 | MTII-3 | CCII-A | CLII-2 | CMGII |
| 2550 | - | 2013 | Madagascar | AEZ4 | S6 | 1594 | -19,48 | 47,40 | AVRIL | 17.3 | ST | - | RS2-MLVA9 | 4-6-6-13-11-4-9-7-10 | MTII-3 | CCII-A | CLII-2 | CMGII |
| 2551 | - | 2013 | Madagascar | AEZ4 | S6 | 1594 | -19,48 | 47,40 | AVRIL | 17.3 | ST | - | RS2-MLVA9 | 4-6-6-13-11-4-9-7-10 | MTII-3 | CCII-A | CLII-2 | CMGII |
| 2552 | - | 2013 | Madagascar | AEZ4 | S6 | 1594 | -19,48 | 47,40 | AVRIL | 17.3 | ST | - | RS2-MLVA9 | 4-6-6-13-11-4-9-7-8 | MTII-13 | CCII-A | CLII-2 | CMGII |
| 2553 | - | 2013 | Madagascar | AEZ4 | S6 | 1594 | -19,48 | 47,40 | AVRIL | 17.3 | ST | - | RS2-MLVA9 | 4-6-6-13-11-4-9-7-9 | MTII-15 | CCII-A | CLII-2 | CMGII |
| 2554 | - | 2013 | Madagascar | AEZ4 | S6 | 1594 | -19,48 | 47,40 | AVRIL | 17.3 | ST | - | RS2-MLVA9 | 4-6-6-13-11-4-9-7-9 | MTII-15 | CCII-A | CLII-2 | CMGII |
| 2555 | - | 2013 | Madagascar | AEZ4 | S6 | 1594 | -19,48 | 47,40 | AVRIL | 17.3 | ST | - | RS2-MLVA9 | 4-6-6-13-11-4-9-7-10 | MTII-3 | CCII-A | CLII-2 | CMGII |
| 2556 | - | 2013 | Madagascar | AEZ4 | S6 | 1594 | -19,48 | 47,40 | AVRIL | 17.3 | ST | - | RS2-MLVA9 | 4-6-6-13-11-4-9-7-10 | MTII-3 | CCII-A | CLII-2 | CMGII |
| 2557 | - | 2013 | Madagascar | AEZ4 | S6 | 1594 | -19,48 | 47,40 | AVRIL | 17.3 | ST | - | RS2-MLVA9 | 4-6-6-13-11-4-9-7-9 | MTII-15 | CCII-A | CLII-2 | CMGII |
| 2559 | - | 2013 | Madagascar | AEZ4 | S3 | 1569 | -19,62 | 47,43 | AVRIL | 17.3 | ST | - | - | - | - | - | - | **-** |
| 2560 | - | 2013 | Madagascar | AEZ4 | S3 | 1569 | -19,62 | 47,43 | AVRIL | 17.3 | ST | - | - | - | - | - | - | **-** |
| 2561 | - | 2013 | Madagascar | AEZ4 | S3 | 1569 | -19,62 | 47,43 | AVRIL | 17.3 | ST | - | - | - | - | - | - | **-** |
| 2562 | - | 2013 | Madagascar | AEZ4 | S3 | 1569 | -19,62 | 47,43 | AVRIL | 17.3 | ST | - | - | - | - | - | - | **-** |
| 2563 | - | 2013 | Madagascar | AEZ4 | S3 | 1569 | -19,62 | 47,43 | AVRIL | 17.3 | ST | - | - | - | - | - | - | **-** |
| 2564 | - | 2013 | Madagascar | AEZ4 | S3 | 1569 | -19,62 | 47,43 | AVRIL | 17.3 | ST | - | - | - | - | - | - | **-** |
| 2566 | - | 2013 | Madagascar | AEZ4 | S3 | 1569 | -19,62 | 47,43 | AVRIL | 17.3 | ST | - | - | - | - | - | - | **-** |
| 2567 | - | 2013 | Madagascar | AEZ4 | S3 | 1569 | -19,62 | 47,43 | AVRIL | 17.3 | ST | - | - | - | - | - | - | **-** |
| 2568 | - | 2013 | Madagascar | AEZ4 | S3 | 1569 | -19,62 | 47,43 | AVRIL | 17.3 | ST | - | - | - | - | - | - | **-** |
| 2569 | - | 2013 | Madagascar | AEZ4 | S3 | 1569 | -19,62 | 47,43 | AVRIL | 17.3 | ST | - | - | - | - | - | - | **-** |
| 2570 | - | 2013 | Madagascar | AEZ4 | S3 | 1569 | -19,62 | 47,43 | AVRIL | 17.3 | ST | - | - | - | - | - | - | **-** |
| 2571 | - | 2013 | Madagascar | AEZ4 | S3 | 1569 | -19,62 | 47,43 | AVRIL | 17.3 | ST | - | - | - | - | - | - | **-** |
| 2572 | - | 2013 | Madagascar | AEZ4 | S3 | 1569 | -19,62 | 47,43 | AVRIL | 17.3 | ST | - | - | - | - | - | - | **-** |
| 2573 | - | 2013 | Madagascar | AEZ4 | S4 | 1596 | -19,49 | 47,58 | AVRIL | 17.3 | ST | 1 | RS2-MLVA9 | 4-5-6-13-11-4-9-7-8 | MTII-9 | CCII-A | CLII-1 | CSEQ, CMGII |
| 2588 | - | 2013 | Madagascar | AEZ4 | S5 | 1573 | -19,59 | 47,53 | AVRIL | 17.3 | ST | - | RS2-MLVA9 | 4-6-6-13-11-4-9-7-9 | MTII-15 | CCII-A | CLII-2 | CMGII |
| 2589 | - | 2013 | Madagascar | AEZ4 | S5 | 1573 | -19,59 | 47,53 | AVRIL | 17.3 | ST | - | RS2-MLVA9 | 4-6-6-13-11-4-9-7-9 | MTII-15 | CCII-A | CLII-2 | CMGII |
| 2590 | - | 2013 | Madagascar | AEZ4 | S5 | 1573 | -19,59 | 47,53 | AVRIL | 17.3 | ST | - | RS2-MLVA9 | 4-6-6-13-11-4-9-7-10 | MTII-3 | CCII-A | CLII-2 | CMGII |
| 2591 | - | 2013 | Madagascar | AEZ4 | S5 | 1573 | -19,59 | 47,53 | AVRIL | 17.3 | ST | - | RS2-MLVA9 | 4-6-6-13-11-4-9-7-10 | MTII-3 | CCII-A | CLII-2 | CMGII |
| 2593 | - | 2013 | Madagascar | AEZ4 | S5 | 1573 | -19,59 | 47,53 | AVRIL | 17.3 | ST | - | RS2-MLVA9 | 4-6-6-13-11-4-9-7-10 | MTII-3 | CCII-A | CLII-2 | CMGII |
| 2595 | - | 2013 | Madagascar | AEZ4 | S5 | 1573 | -19,59 | 47,53 | AVRIL | 17.3 | ST | - | RS2-MLVA9 | 4-6-6-13-11-4-9-7-10 | MTII-3 | CCII-A | CLII-2 | CMGII |
| 2596 | - | 2013 | Madagascar | AEZ4 | S5 | 1573 | -19,59 | 47,53 | AVRIL | 17.3 | ST | - | RS2-MLVA9 | 4-6-6-13-11-4-9-7-10 | MTII-3 | CCII-A | CLII-2 | CMGII |
| 2597 | - | 2013 | Madagascar | AEZ4 | S5 | 1573 | -19,59 | 47,53 | AVRIL | 17.3 | ST | - | RS2-MLVA9 | 1-6-6-13-11-1-9-7-10 | MTII-1 | CCII-A | CLII-2 | CMGII |
| 2598 | - | 2013 | Madagascar | AEZ4 | S5 | 1573 | -19,59 | 47,53 | AVRIL | 17.3 | ST | 1 | RS2-MLVA9 | 4-6-6-13-11-4-9-7-10 | MTII-3 | CCII-A | CLII-2 | CSEQ, CMGII |
| 2599 | - | 2013 | Madagascar | AEZ4 | S5 | 1573 | -19,59 | 47,53 | AVRIL | 17.3 | ST | - | RS2-MLVA9 | 4-6-6-13-11-4-9-7-10 | MTII-3 | CCII-A | CLII-2 | CMGII |
| 2600 | - | 2013 | Madagascar | AEZ4 | S5 | 1573 | -19,59 | 47,53 | AVRIL | 17.3 | ST | - | RS2-MLVA9 | 4-6-6-13-11-4-9-7-10 | MTII-3 | CCII-A | CLII-2 | CMGII |
| 2601 | - | 2013 | Madagascar | AEZ4 | S5 | 1573 | -19,59 | 47,53 | AVRIL | 17.3 | ST | - | RS2-MLVA9 | 4-6-6-13-11-4-9-7-10 | MTII-3 | CCII-A | CLII-2 | CMGII |
| 2602 | - | 2013 | Madagascar | AEZ4 | S5 | 1573 | -19,59 | 47,53 | AVRIL | 17.3 | ST | - | RS2-MLVA9 | 4-6-6-13-11-4-9-7-10 | MTII-3 | CCII-A | CLII-2 | CMGII |
| 2603 | - | 2013 | Madagascar | AEZ4 | S5 | 1573 | -19,59 | 47,53 | AVRIL | 17.3 | ST | - | RS2-MLVA9 | 4-6-6-13-11-4-9-7-10 | MTII-3 | CCII-A | CLII-2 | CMGII |
| 2604 | - | 2013 | Madagascar | AEZ4 | S5 | 1573 | -19,59 | 47,53 | AVRIL | 17.3 | ST | - | RS2-MLVA9 | 4-6-6-13-11-4-9-7-10 | MTII-3 | CCII-A | CLII-2 | CMGII |
| 2605 | - | 2013 | Madagascar | AEZ4 | S5 | 1573 | -19,59 | 47,53 | AVRIL | 17.3 | ST | - | RS2-MLVA9 | 4-5-6-13-11-4-9-7-8 | MTII-9 | CCII-A | CLII-2 | CMGII |
| 2606 | - | 2013 | Madagascar | AEZ4 | S5 | 1573 | -19,59 | 47,53 | AVRIL | 17.3 | ST | - | RS2-MLVA9 | 4-6-6-13-11-4-9-7-10 | MTII-3 | CCII-A | CLII-2 | CMGII |
| 2607 | - | 2013 | Madagascar | AEZ4 | S5 | 1573 | -19,59 | 47,53 | AVRIL | 17.3 | PV | - | RS2-MLVA9 | 4-6-6-13-11-4-9-7-10 | MTII-3 | CCII-A | CLII-2 | CMGII |
| 2608 | - | 2013 | Madagascar | AEZ4 | S5 | 1573 | -19,59 | 47,53 | AVRIL | 17.3 | SL | - | RS2-MLVA9 | 1-6-6-13-11-1-9-7-10 | MTII-1 | CCII-A | CLII-2 | CMGII |
| 2609 | - | 2013 | Madagascar | AEZ3 | S65 | 1929 | -19,72 | 47,05 | AVRIL | 17.5 | ST | - | - | - | - | - | - | **-** |
| 2610 | - | 2013 | Madagascar | AEZ3 | S65 | 1929 | -19,72 | 47,05 | AVRIL | 17.5 | ST | - | - | - | - | - | - | **-** |
| 2611 | - | 2013 | Madagascar | AEZ2 | S58 | 1518 | -19,95 | 47,03 | AVRIL | 18.3 | ST | - | - | - | - | - | - | **-** |
| 2612 | - | 2013 | Madagascar | AEZ2 | S61 | 1609 | -19,98 | 47,04 | AVRIL | 18.3 | ST | - | - | - | - | - | - | **-** |
| 2613 | - | 2013 | Madagascar | AEZ2 | S61 | 1609 | -19,98 | 47,04 | AVRIL | 18.3 | ST | - | - | - | - | - | - | **-** |
| 2614 | - | 2013 | Madagascar | AEZ2 | S58 | 1518 | -19,95 | 47,03 | AVRIL | 18.3 | ST | - | - | - | - | - | - | **-** |
| 2615 | - | 2013 | Madagascar | AEZ2 | S58 | 1518 | -19,95 | 47,03 | AVRIL | 18.3 | ST | - | - | - | - | - | - | **-** |
| 2616 | - | 2013 | Madagascar | AEZ2 | S58 | 1518 | -19,95 | 47,03 | AVRIL | 18.3 | ST | - | - | - | - | - | - | **-** |
| 2617 | - | 2013 | Madagascar | AEZ2 | S58 | 1518 | -19,95 | 47,03 | AVRIL | 18.3 | ST | - | - | - | - | - | - | **-** |
| 2618 | - | 2013 | Madagascar | AEZ2 | S58 | 1518 | -19,95 | 47,03 | AVRIL | 18.3 | ST | - | - | - | - | - | - | **-** |
| 2619 | - | 2013 | Madagascar | AEZ2 | S58 | 1518 | -19,95 | 47,03 | AVRIL | 18.3 | ST | - | - | - | - | - | - | **-** |
| 2620 | - | 2013 | Madagascar | AEZ2 | S58 | 1518 | -19,95 | 47,03 | AVRIL | 18.3 | ST | - | - | - | - | - | - | **-** |
| 2621 | - | 2013 | Madagascar | AEZ2 | S58 | 1518 | -19,95 | 47,03 | AVRIL | 18.3 | ST | - | - | - | - | - | - | **-** |
| 2622 | - | 2013 | Madagascar | AEZ2 | S58 | 1518 | -19,95 | 47,03 | AVRIL | 18.3 | ST | - | - | - | - | - | - | **-** |
| 2623 | - | 2013 | Madagascar | AEZ2 | S58 | 1518 | -19,95 | 47,03 | AVRIL | 18.3 | ST | - | - | - | - | - | - | **-** |
| 2624 | - | 2013 | Madagascar | AEZ2 | S58 | 1518 | -19,95 | 47,03 | AVRIL | 18.3 | ST | - | - | - | - | - | - | **-** |
| 2625 | - | 2013 | Madagascar | AEZ2 | S58 | 1518 | -19,95 | 47,03 | AVRIL | 18.3 | ST | - | - | - | - | - | - | **-** |
| 2626 | - | 2013 | Madagascar | AEZ2 | S58 | 1518 | -19,95 | 47,03 | AVRIL | 18.3 | ST | - | - | - | - | - | - | **-** |
| 2627 | - | 2013 | Madagascar | AEZ2 | S58 | 1518 | -19,95 | 47,03 | AVRIL | 18.3 | ST | - | - | - | - | - | - | **-** |
| 2628 | - | 2013 | Madagascar | AEZ2 | S57 | 1531 | -19,94 | 47,02 | AVRIL | 18.3 | ST | - | - | - | - | - | - | **-** |
| 2629 | - | 2013 | Madagascar | AEZ2 | S57 | 1531 | -19,94 | 47,02 | AVRIL | 18.3 | ST | - | - | - | - | - | - | **-** |
| 2630 | - | 2013 | Madagascar | AEZ2 | S57 | 1531 | -19,94 | 47,02 | AVRIL | 18.3 | ST | - | - | - | - | - | - | **-** |
| 2631 | - | 2013 | Madagascar | AEZ2 | S57 | 1531 | -19,94 | 47,02 | AVRIL | 18.3 | ST | - | - | - | - | - | - | **-** |
| 2632 | - | 2013 | Madagascar | AEZ2 | S57 | 1531 | -19,94 | 47,02 | AVRIL | 18.3 | ST | - | - | - | - | - | - | **-** |
| 2633 | - | 2013 | Madagascar | AEZ2 | S57 | 1531 | -19,94 | 47,02 | AVRIL | 18.3 | ST | - | - | - | - | - | - | **-** |
| 2634 | - | 2013 | Madagascar | AEZ2 | S57 | 1531 | -19,94 | 47,02 | AVRIL | 18.3 | ST | - | - | - | - | - | - | **-** |
| 2635 | - | 2013 | Madagascar | AEZ2 | S57 | 1531 | -19,94 | 47,02 | AVRIL | 18.3 | ST | - | - | - | - | - | - | **-** |
| 2636 | - | 2013 | Madagascar | AEZ2 | S57 | 1531 | -19,94 | 47,02 | AVRIL | 18.3 | ST | - | - | - | - | - | - | **-** |
| 2637 | - | 2013 | Madagascar | AEZ2 | S57 | 1531 | -19,94 | 47,02 | AVRIL | 18.3 | ST | - | - | - | - | - | - | **-** |
| 2638 | - | 2013 | Madagascar | AEZ2 | S57 | 1531 | -19,94 | 47,02 | AVRIL | 18.3 | ST | - | - | - | - | - | - | **-** |
| 2639 | - | 2013 | Madagascar | AEZ2 | S57 | 1531 | -19,94 | 47,02 | AVRIL | 18.3 | ST | - | - | - | - | - | - | **-** |
| 2640 | - | 2013 | Madagascar | AEZ2 | S57 | 1531 | -19,94 | 47,02 | AVRIL | 18.3 | ST | - | - | - | - | - | - | **-** |
| 2641 | - | 2013 | Madagascar | AEZ2 | S57 | 1531 | -19,94 | 47,02 | AVRIL | 18.3 | ST | - | - | - | - | - | - | **-** |
| 2642 | - | 2013 | Madagascar | AEZ2 | S57 | 1531 | -19,94 | 47,02 | AVRIL | 18.3 | ST | - | - | - | - | - | - | **-** |
| 2643 | - | 2013 | Madagascar | AEZ2 | S57 | 1531 | -19,94 | 47,02 | AVRIL | 18.3 | ST | - | - | - | - | - | - | **-** |
| 2644 | - | 2013 | Madagascar | AEZ2 | S57 | 1531 | -19,94 | 47,02 | AVRIL | 18.3 | ST | - | - | - | - | - | - | **-** |
| 2645 | - | 2013 | Madagascar | AEZ2 | S57 | 1531 | -19,94 | 47,02 | AVRIL | 18.3 | ST | - | - | - | - | - | - | **-** |
| 2646 | - | 2013 | Madagascar | AEZ2 | S55 | 1521 | -19,90 | 47,12 | AVRIL | 18.3 | ST | 1 | RS2-MLVA9 | 4-3-6-13-12-4-9-7-10 | MTII-16 | CCII-A | CLII-3 | CSEQ, CMGII |
| 2647 | - | 2013 | Madagascar | AEZ2 | S55 | 1521 | -19,90 | 47,12 | AVRIL | 18.3 | ST | - | RS2-MLVA9 | 4-3-6-13-12-4-9-7-10 | MTII-16 | CCII-A | CLII-3 | CMGII |
| 2648 | - | 2013 | Madagascar | AEZ2 | S55 | 1521 | -19,90 | 47,12 | AVRIL | 18.3 | ST | - | RS2-MLVA9 | 4-5-6-13-11-4-9-7-10 | MTII-2 | CCII-A | CLII-3 | CMGII |
| 2649 | - | 2013 | Madagascar | AEZ2 | S55 | 1521 | -19,90 | 47,12 | AVRIL | 18.3 | ST | - | RS2-MLVA9 | 4-3-6-13-12-4-9-7-10 | MTII-16 | CCII-A | CLII-3 | CMGII |
| 2650 | - | 2013 | Madagascar | AEZ2 | S55 | 1521 | -19,90 | 47,12 | AVRIL | 18.3 | ST | - | RS2-MLVA9 | 4-3-6-13-12-4-9-7-10 | MTII-16 | CCII-A | CLII-3 | CMGII |
| 2651 | - | 2013 | Madagascar | AEZ2 | S55 | 1521 | -19,90 | 47,12 | AVRIL | 18.3 | ST | - | RS2-MLVA9 | 4-3-6-13-12-4-9-7-10 | MTII-16 | CCII-A | CLII-3 | CMGII |
| 2652 | - | 2013 | Madagascar | AEZ2 | S55 | 1521 | -19,90 | 47,12 | AVRIL | 18.3 | ST | - | RS2-MLVA9 | 4-3-6-13-12-4-9-7-10 | MTII-16 | CCII-A | CLII-3 | CMGII |
| 2653 | - | 2013 | Madagascar | AEZ2 | S55 | 1521 | -19,90 | 47,12 | AVRIL | 18.3 | ST | - | RS2-MLVA9 | 4-3-6-13-12-4-9-7-10 | MTII-16 | CCII-A | CLII-3 | CMGII |
| 2654 | - | 2013 | Madagascar | AEZ2 | S55 | 1521 | -19,90 | 47,12 | AVRIL | 18.3 | ST | - | RS2-MLVA9 | 4-3-6-13-12-4-9-7-10 | MTII-16 | CCII-A | CLII-3 | CMGII |
| 2655 | - | 2013 | Madagascar | AEZ2 | S55 | 1521 | -19,90 | 47,12 | AVRIL | 18.3 | ST | - | RS2-MLVA9 | 4-5-6-13-11-4-9-7-9 | MTII-14 | CCII-A | CLII-3 | CMGII |
| 2656 | - | 2013 | Madagascar | AEZ2 | S55 | 1521 | -19,90 | 47,12 | AVRIL | 18.3 | ST | - | RS2-MLVA9 | 4-3-6-13-12-4-9-7-10 | MTII-16 | CCII-A | CLII-3 | CMGII |
| 2657 | - | 2013 | Madagascar | AEZ2 | S55 | 1521 | -19,90 | 47,12 | AVRIL | 18.3 | ST | - | RS2-MLVA9 | 4-5-7-13-12-4-9-7-10 | MTII-46 | CCII-A | CLII-3 | CMGII |
| 2658 | - | 2013 | Madagascar | AEZ2 | S55 | 1521 | -19,90 | 47,12 | AVRIL | 18.3 | ST | 1 | RS2-MLVA9 | 4-3-6-13-12-4-9-7-10 | MTII-16 | CCII-A | CLII-3 | CSEQ, CMGII |
| 2659 | - | 2013 | Madagascar | AEZ2 | S55 | 1521 | -19,90 | 47,12 | AVRIL | 18.3 | ST | - | RS2-MLVA9 | 4-3-6-13-12-4-9-7-10 | MTII-16 | CCII-A | CLII-3 | CMGII |
| 2660 | - | 2013 | Madagascar | AEZ2 | S55 | 1521 | -19,90 | 47,12 | AVRIL | 18.3 | ST | - | RS2-MLVA9 | 4-3-6-13-12-4-9-7-10 | MTII-16 | CCII-A | CLII-3 | CMGII |
| 2661 | - | 2013 | Madagascar | AEZ2 | S55 | 1521 | -19,90 | 47,12 | AVRIL | 18.3 | ST | - | RS2-MLVA9 | 4-6-6-13-11-4-9-7-10 | MTII-3 | CCII-A | CLII-3 | CMGII |
| 2662 | - | 2013 | Madagascar | AEZ2 | S55 | 1521 | -19,90 | 47,12 | AVRIL | 18.3 | ST | - | RS2-MLVA9 | 4-6-6-13-11-4-9-7-10 | MTII-3 | CCII-A | CLII-3 | CMGII |
| 2663 | - | 2013 | Madagascar | AEZ2 | S55 | 1521 | -19,90 | 47,12 | AVRIL | 18.3 | ST | - | RS2-MLVA9 | 4-3-6-13-12-1-9-7-10 | MTII-45 | CCII-A | CLII-3 | CMGII |
| 2664 | - | 2013 | Madagascar | AEZ2 | S55 | 1521 | -19,90 | 47,12 | AVRIL | 18.3 | ST | - | RS2-MLVA9 | 4-3-6-13-12-4-9-7-10 | MTII-16 | CCII-A | CLII-3 | CMGII |
| 2665 | - | 2013 | Madagascar | AEZ2 | S55 | 1521 | -19,90 | 47,12 | AVRIL | 18.3 | ST | - | RS2-MLVA9 | 4-3-6-13-12-4-9-7-10 | MTII-16 | CCII-A | CLII-3 | CMGII |
| 2666 | - | 2013 | Madagascar | AEZ4 | S1 | 1591 | -19,37 | 47,46 | AVRIL | 17.3 | ST | - | - | - | - | - | - | **-** |
| 2667 | - | 2013 | Madagascar | AEZ4 | S1 | 1591 | -19,37 | 47,46 | AVRIL | 17.3 | ST | - | - | - | - | - | - | **-** |
| 2668 | - | 2013 | Madagascar | AEZ4 | S1 | 1591 | -19,37 | 47,46 | AVRIL | 17.3 | ST | - | - | - | - | - | - | **-** |
| 2669 | - | 2013 | Madagascar | AEZ4 | S1 | 1591 | -19,37 | 47,46 | AVRIL | 17.3 | ST | - | - | - | - | - | - | **-** |
| 2670 | - | 2013 | Madagascar | AEZ4 | S1 | 1591 | -19,37 | 47,46 | AVRIL | 17.3 | ST | - | - | - | - | - | - | **-** |
| 2671 | - | 2013 | Madagascar | AEZ4 | S1 | 1591 | -19,37 | 47,46 | AVRIL | 17.3 | ST | - | - | - | - | - | - | **-** |
| 2672 | - | 2013 | Madagascar | AEZ4 | S1 | 1591 | -19,37 | 47,46 | AVRIL | 17.3 | ST | - | - | - | - | - | - | **-** |
| 2673 | - | 2013 | Madagascar | AEZ4 | S1 | 1591 | -19,37 | 47,46 | AVRIL | 17.3 | ST | - | - | - | - | - | - | **-** |
| 2674 | - | 2013 | Madagascar | AEZ4 | S1 | 1591 | -19,37 | 47,46 | AVRIL | 17.3 | ST | - | - | - | - | - | - | **-** |
| 2675 | - | 2013 | Madagascar | AEZ4 | S1 | 1591 | -19,37 | 47,46 | AVRIL | 17.3 | ST | - | - | - | - | - | - | **-** |
| 2676 | - | 2013 | Madagascar | AEZ4 | S1 | 1591 | -19,37 | 47,46 | AVRIL | 17.3 | ST | - | - | - | - | - | - | **-** |
| 2677 | - | 2013 | Madagascar | AEZ4 | S1 | 1591 | -19,37 | 47,46 | AVRIL | 17.3 | ST | 1 | RS2-MLVA9 | 1-6-6-13-11-1-9-7-10 | MTII-1 | CCII-A | CLII-2 | CSEQ, CMGII |
| 2678 | - | 2013 | Madagascar | AEZ4 | S2 | 1593 | -19,45 | 47,67 | AVRIL | 17.3 | ST | - | - | - | - | - | - | **-** |
| 2679 | - | 2013 | Madagascar | AEZ4 | S2 | 1593 | -19,45 | 47,67 | AVRIL | 17.3 | ST | - | - | - | - | - | - | **-** |
| 2680 | - | 2013 | Madagascar | AEZ4 | S2 | 1593 | -19,45 | 47,67 | AVRIL | 17.3 | ST | - | - | - | - | - | - | **-** |
| 2681 | - | 2013 | Madagascar | AEZ4 | S2 | 1593 | -19,45 | 47,67 | AVRIL | 17.3 | ST | - | - | - | - | - | - | **-** |
| 2682 | - | 2013 | Madagascar | AEZ4 | S2 | 1593 | -19,45 | 47,67 | AVRIL | 17.3 | ST | - | - | - | - | - | - | **-** |
| 2683 | - | 2013 | Madagascar | AEZ4 | S2 | 1593 | -19,45 | 47,67 | AVRIL | 17.3 | ST | - | - | - | - | - | - | **-** |
| 2684 | - | 2013 | Madagascar | AEZ4 | S2 | 1593 | -19,45 | 47,67 | AVRIL | 17.3 | ST | - | - | - | - | - | - | **-** |
| 2685 | - | 2013 | Madagascar | AEZ3 | S65 | 1929 | -19,72 | 47,05 | AVRIL | 17.5 | ST | - | - | - | - | - | - | **-** |
| 2686 | - | 2013 | Madagascar | AEZ3 | S65 | 1929 | -19,72 | 47,05 | AVRIL | 17.5 | ST | - | - | - | - | - | - | **-** |
| 2687 | - | 2013 | Madagascar | AEZ3 | S65 | 1929 | -19,72 | 47,05 | AVRIL | 17.5 | ST | - | - | - | - | - | - | **-** |
| 2688 | - | 2013 | Madagascar | AEZ3 | S65 | 1929 | -19,72 | 47,05 | AVRIL | 17.5 | ST | - | - | - | - | - | - | **-** |
| 2689 | - | 2013 | Madagascar | AEZ3 | S65 | 1929 | -19,72 | 47,05 | AVRIL | 17.5 | ST | - | - | - | - | - | - | **-** |
| 2690 | - | 2013 | Madagascar | AEZ3 | S65 | 1929 | -19,72 | 47,05 | AVRIL | 17.5 | ST | - | - | - | - | - | - | **-** |
| 2691 | - | 2013 | Madagascar | AEZ3 | S65 | 1929 | -19,72 | 47,05 | AVRIL | 17.5 | ST | - | - | - | - | - | - | **-** |
| 2692 | - | 2013 | Madagascar | AEZ3 | S65 | 1929 | -19,72 | 47,05 | AVRIL | 17.5 | ST | - | - | - | - | - | - | **-** |
| 2693 | - | 2013 | Madagascar | AEZ3 | S65 | 1929 | -19,72 | 47,05 | AVRIL | 17.5 | ST | - | - | - | - | - | - | **-** |
| 2694 | - | 2013 | Madagascar | AEZ3 | S65 | 1929 | -19,72 | 47,05 | AVRIL | 17.5 | ST | - | - | - | - | - | - | **-** |
| 2695 | - | 2013 | Madagascar | AEZ3 | S65 | 1929 | -19,72 | 47,05 | AVRIL | 17.5 | ST | - | - | - | - | - | - | **-** |
| 2696 | - | 2013 | Madagascar | AEZ3 | S65 | 1929 | -19,72 | 47,05 | AVRIL | 17.5 | ST | - | - | - | - | - | - | **-** |
| 2697 | - | 2013 | Madagascar | AEZ3 | S65 | 1929 | -19,72 | 47,05 | AVRIL | 17.5 | ST | - | - | - | - | - | - | **-** |
| 2698 | - | 2013 | Madagascar | AEZ3 | S65 | 1929 | -19,72 | 47,05 | AVRIL | 17.5 | ST | - | - | - | - | - | - | **-** |
| 2699 | - | 2013 | Madagascar | AEZ3 | S65 | 1929 | -19,72 | 47,05 | AVRIL | 17.5 | ST | - | - | - | - | - | - | **-** |
| 2700 | - | 2013 | Madagascar | AEZ3 | S65 | 1929 | -19,72 | 47,05 | AVRIL | 17.5 | ST | - | - | - | - | - | - | **-** |
| 2701 | - | 2013 | Madagascar | AEZ3 | S65 | 1929 | -19,72 | 47,05 | AVRIL | 17.5 | ST | - | - | - | - | - | - | **-** |
| 2702 | - | 2013 | Madagascar | AEZ3 | S66 | 1950 | -19,68 | 47,27 | AVRIL | 17.5 | ST | - | - | - | - | - | - | **-** |
| 2703 | - | 2013 | Madagascar | AEZ3 | S66 | 1950 | -19,68 | 47,27 | AVRIL | 17.5 | ST | - | - | - | - | - | - | **-** |
| 2704 | - | 2013 | Madagascar | AEZ3 | S66 | 1950 | -19,68 | 47,27 | AVRIL | 17.5 | ST | - | - | - | - | - | - | **-** |
| 2705 | - | 2013 | Madagascar | AEZ3 | S66 | 1950 | -19,68 | 47,27 | AVRIL | 17.5 | ST | - | - | - | - | - | - | **-** |
| 2707 | - | 2013 | Madagascar | AEZ3 | S66 | 1950 | -19,68 | 47,27 | AVRIL | 17.5 | ST | - | - | - | - | - | - | **-** |
| 2708 | - | 2013 | Madagascar | AEZ3 | S66 | 1950 | -19,68 | 47,27 | AVRIL | 17.5 | ST | - | - | - | - | - | - | **-** |
| 2710 | - | 2013 | Madagascar | AEZ3 | S68 | 1937 | -19,59 | 47,04 | AVRIL | 17.5 | ST | - | - | - | - | - | - | **-** |
| 2711 | - | 2013 | Madagascar | AEZ3 | S66 | 1950 | -19,68 | 47,27 | AVRIL | 17.5 | ST | - | - | - | - | - | - | **-** |
| 2723 | - | 2013 | Madagascar | AEZ3 | S66 | 1950 | -19,68 | 47,27 | AVRIL | 17.5 | ST | - | - | - | - | - | - | **-** |
| 2724 | - | 2013 | Madagascar | AEZ3 | S66 | 1950 | -19,68 | 47,27 | AVRIL | 17.5 | ST | - | - | - | - | - | - | **-** |
| 2728 | - | 2013 | Madagascar | AEZ3 | S66 | 1950 | -19,68 | 47,27 | AVRIL | 17.5 | ST | - | - | - | - | - | - | **-** |
| 2729 | - | 2013 | Madagascar | AEZ3 | S66 | 1950 | -19,68 | 47,27 | AVRIL | 17.5 | ST | - | - | - | - | - | - | **-** |
| 2730 | - | 2013 | Madagascar | AEZ3 | S67 | 1947 | -19,59 | 47,25 | AVRIL | 17.5 | ST | 1 | RS2-MLVA9 | 1-5-6-13-11-1-9-7-8 | MTII-7 | CCII-A | CLII-2 | CSEQ, CMGII |
| 2731 | - | 2013 | Madagascar | AEZ3 | S67 | 1947 | -19,59 | 47,25 | AVRIL | 17.5 | ST | - | RS2-MLVA9 | 4-4-6-13-11-4-9-7-8 | MTII-8 | CCII-A | CLII-2 | CMGII |
| 2732 | - | 2013 | Madagascar | AEZ3 | S67 | 1947 | -19,59 | 47,25 | AVRIL | 17.5 | ST | - | RS2-MLVA9 | 4-4-6-13-11-4-9-7-8 | MTII-8 | CCII-A | CLII-2 | CMGII |
| 2733 | - | 2013 | Madagascar | AEZ3 | S67 | 1947 | -19,59 | 47,25 | AVRIL | 17.5 | ST | - | RS2-MLVA9 | 4-5-6-13-11-4-9-7-8 | MTII-9 | CCII-A | CLII-2 | CMGII |
| 2734 | - | 2013 | Madagascar | AEZ3 | S67 | 1947 | -19,59 | 47,25 | AVRIL | 17.5 | ST | - | RS2-MLVA9 | 4-6-6-13-11-4-9-7-10 | MTII-3 | CCII-A | CLII-2 | CMGII |
| 2735 | - | 2013 | Madagascar | AEZ3 | S67 | 1947 | -19,59 | 47,25 | AVRIL | 17.5 | ST | - | RS2-MLVA9 | 4-4-6-13-11-4-9-7-8 | MTII-8 | CCII-A | CLII-2 | CMGII |
| 2736 | - | 2013 | Madagascar | AEZ3 | S67 | 1947 | -19,59 | 47,25 | AVRIL | 17.5 | ST | 1 | RS2-MLVA9 | 4-6-6-13-11-4-9-7-10 | MTII-3 | CCII-A | CLII-2 | CSEQ, CMGII |
| 2737 | - | 2013 | Madagascar | AEZ3 | S67 | 1947 | -19,59 | 47,25 | AVRIL | 17.5 | ST | - | RS2-MLVA9 | 4-6-6-13-11-4-9-7-10 | MTII-3 | CCII-A | CLII-2 | CMGII |
| 2738 | - | 2013 | Madagascar | AEZ3 | S67 | 1947 | -19,59 | 47,25 | AVRIL | 17.5 | ST | - | RS2-MLVA9 | 4-6-6-13-11-4-9-7-10 | MTII-3 | CCII-A | CLII-2 | CMGII |
| 2739 | - | 2013 | Madagascar | AEZ3 | S67 | 1947 | -19,59 | 47,25 | AVRIL | 17.5 | ST | - | RS2-MLVA9 | 4-5-6-13-11-4-9-7-8 | MTII-9 | CCII-A | CLII-2 | CMGII |
| 2740 | - | 2013 | Madagascar | AEZ3 | S67 | 1947 | -19,59 | 47,25 | AVRIL | 17.5 | ST | - | RS2-MLVA9 | 4-6-6-13-11-4-9-7-10 | MTII-3 | CCII-A | CLII-2 | CMGII |
| 2741 | - | 2013 | Madagascar | AEZ3 | S67 | 1947 | -19,59 | 47,25 | AVRIL | 17.5 | ST | - | RS2-MLVA9 | 4-6-6-13-11-4-9-7-10 | MTII-3 | CCII-A | CLII-2 | CMGII |
| 2742 | - | 2013 | Madagascar | AEZ3 | S67 | 1947 | -19,59 | 47,25 | AVRIL | 17.5 | ST | - | RS2-MLVA9 | 4-6-6-13-11-4-9-7-10 | MTII-3 | CCII-A | CLII-2 | CMGII |
| 2743 | - | 2013 | Madagascar | AEZ3 | S67 | 1947 | -19,59 | 47,25 | AVRIL | 17.5 | ST | - | RS2-MLVA9 | 4-6-6-13-11-4-9-7-10 | MTII-3 | CCII-A | CLII-2 | CMGII |
| 2744 | - | 2013 | Madagascar | AEZ3 | S67 | 1947 | -19,59 | 47,25 | AVRIL | 17.5 | ST | - | RS2-MLVA9 | 4-6-6-13-11-4-9-7-10 | MTII-3 | CCII-A | CLII-2 | CMGII |
| 2745 | - | 2013 | Madagascar | AEZ3 | S67 | 1947 | -19,59 | 47,25 | AVRIL | 17.5 | ST | - | RS2-MLVA9 | 4-6-6-13-11-4-9-7-10 | MTII-3 | CCII-A | CLII-2 | CMGII |
| 2746 | - | 2013 | Madagascar | AEZ3 | S67 | 1947 | -19,59 | 47,25 | AVRIL | 17.5 | ST | - | RS2-MLVA9 | 4-6-6-13-11-4-9-7-10 | MTII-3 | CCII-A | CLII-2 | CMGII |
| 2747 | - | 2013 | Madagascar | AEZ3 | S67 | 1947 | -19,59 | 47,25 | AVRIL | 17.5 | ST | - | RS2-MLVA9 | 4-5-2-10-11-4-9-7-8 | MTII-32 | CCII-A | CLII-2 | CMGII |
| 2748 | - | 2013 | Madagascar | AEZ3 | S67 | 1947 | -19,59 | 47,25 | AVRIL | 17.5 | ST | - | RS2-MLVA9 | 4-5-6-13-11-4-9-7-8 | MTII-9 | CCII-A | CLII-2 | CMGII |
| 2749 | - | 2013 | Madagascar | AEZ3 | S67 | 1947 | -19,59 | 47,25 | AVRIL | 17.5 | ST | - | RS2-MLVA9 | 4-5-6-14-11-4-9-7-8 | MTII-11 | CCII-A | CLII-2 | CMGII |
| 2750 | - | 2013 | Madagascar | AEZ3 | S67 | 1947 | -19,59 | 47,25 | AVRIL | 17.5 | ST | - | RS2-MLVA9 | 4-6-6-13-11-4-9-7-10 | MTII-3 | CCII-A | CLII-2 | CMGII |
| 2751 | - | 2013 | Madagascar | AEZ3 | S67 | 1947 | -19,59 | 47,25 | AVRIL | 17.5 | ST | - | RS2-MLVA9 | 4-4-6-13-11-4-9-7-8 | MTII-8 | CCII-A | CLII-2 | CMGII |
| 2752 | - | 2013 | Madagascar | AEZ3 | S69 | 1941 | -19,59 | 47,03 | AVRIL | 17.5 | ST | - | - | - | - | - | - | **-** |
| 2754 | - | 2013 | Madagascar | AEZ3 | S69 | 1941 | -19,59 | 47,03 | AVRIL | 17.5 | ST | - | - | - | - | - | - | **-** |
| 2755 | - | 2013 | Madagascar | AEZ3 | S69 | 1941 | -19,59 | 47,03 | AVRIL | 17.5 | ST | - | - | - | - | - | - | **-** |
| 2756 | - | 2013 | Madagascar | AEZ3 | S69 | 1941 | -19,59 | 47,03 | AVRIL | 17.5 | ST | - | RS2-MLVA9 | 4-5-6-13-11-4-9-6-8 | MTII-38 | CCII-A | CLII-1 | CMGII |
| 2764 | - | 2013 | Madagascar | AEZ3 | S69 | 1941 | -19,59 | 47,03 | AVRIL | 17.5 | ST | - | - | - | - | - | - | **-** |
| 2765 | - | 2013 | Madagascar | AEZ3 | S69 | 1941 | -19,59 | 47,03 | AVRIL | 17.5 | ST | - | - | - | - | - | - | **-** |
| 2766 | - | 2013 | Madagascar | AEZ3 | S69 | 1941 | -19,59 | 47,03 | AVRIL | 17.5 | ST | - | - | - | - | - | - | **-** |
| 2768 | - | 2013 | Madagascar | AEZ3 | S69 | 1941 | -19,59 | 47,03 | AVRIL | 17.5 | ST | - | - | - | - | - | - | **-** |
| 2769 | - | 2013 | Madagascar | AEZ3 | S69 | 1941 | -19,59 | 47,03 | AVRIL | 17.5 | ST | - | - | - | - | - | - | **-** |
| 2772 | - | 2013 | Madagascar | AEZ3 | S68 | 1937 | -19,59 | 47,04 | AVRIL | 17.5 | ST | - | - | - | - | - | - | **-** |
| 2773 | - | 2013 | Madagascar | AEZ3 | S68 | 1937 | -19,59 | 47,04 | AVRIL | 17.5 | ST | - | - | - | - | - | - | **-** |
| 2775 | - | 2013 | Madagascar | AEZ3 | S68 | 1937 | -19,59 | 47,04 | AVRIL | 17.5 | ST | - | - | - | - | - | - | **-** |
| 2777 | - | 2013 | Madagascar | AEZ3 | S68 | 1937 | -19,59 | 47,04 | AVRIL | 17.5 | ST | - | - | - | - | - | - | **-** |
| 2778 | - | 2013 | Madagascar | AEZ3 | S68 | 1937 | -19,59 | 47,04 | AVRIL | 17.5 | ST | - | - | - | - | - | - | **-** |
| 2779 | - | 2013 | Madagascar | AEZ3 | S68 | 1937 | -19,59 | 47,04 | AVRIL | 17.5 | ST | - | - | - | - | - | - | **-** |
| 2780 | - | 2013 | Madagascar | AEZ3 | S68 | 1937 | -19,59 | 47,04 | AVRIL | 17.5 | ST | - | - | - | - | - | - | **-** |
| 2781 | - | 2013 | Madagascar | AEZ3 | S68 | 1937 | -19,59 | 47,04 | AVRIL | 17.5 | ST | - | - | - | - | - | - | **-** |
| 2782 | - | 2013 | Madagascar | AEZ3 | S68 | 1937 | -19,59 | 47,04 | AVRIL | 17.5 | ST | - | - | - | - | - | - | **-** |
| 2783 | - | 2013 | Madagascar | AEZ3 | S68 | 1937 | -19,59 | 47,04 | AVRIL | 17.5 | ST | - | - | - | - | - | - | **-** |
| 2787 | - | 2013 | Madagascar | AEZ1 | S29 | 1780 | -19,99 | 47,24 | AVRIL | 17.7 | ST | - | - | - | - | - | - | **-** |
| 2788 | - | 2013 | Madagascar | AEZ1 | S29 | 1780 | -19,99 | 47,24 | AVRIL | 17.7 | ST | - | - | - | - | - | - | **-** |
| 2789 | - | 2013 | Madagascar | AEZ1 | S29 | 1780 | -19,99 | 47,24 | AVRIL | 17.7 | ST | - | - | - | - | - | - | **-** |
| 2790 | - | 2013 | Madagascar | AEZ1 | S29 | 1780 | -19,99 | 47,24 | AVRIL | 17.7 | ST | - | - | - | - | - | - | **-** |
| 2791 | - | 2013 | Madagascar | AEZ1 | S29 | 1780 | -19,99 | 47,24 | AVRIL | 17.7 | ST | - | - | - | - | - | - | **-** |
| 2792 | - | 2013 | Madagascar | AEZ1 | S29 | 1780 | -19,99 | 47,24 | AVRIL | 17.7 | ST | - | - | - | - | - | - | **-** |
| 2793 | - | 2013 | Madagascar | AEZ1 | S29 | 1780 | -19,99 | 47,24 | AVRIL | 17.7 | ST | - | - | - | - | - | - | **-** |
| 2794 | - | 2013 | Madagascar | AEZ1 | S29 | 1780 | -19,99 | 47,24 | AVRIL | 17.7 | ST | - | - | - | - | - | - | **-** |
| 2795 | - | 2013 | Madagascar | AEZ1 | S29 | 1780 | -19,99 | 47,24 | AVRIL | 17.7 | ST | - | - | - | - | - | - | **-** |
| 2796 | - | 2013 | Madagascar | AEZ1 | S29 | 1780 | -19,99 | 47,24 | AVRIL | 17.7 | ST | - | - | - | - | - | - | **-** |
| 2797 | - | 2013 | Madagascar | AEZ1 | S29 | 1780 | -19,99 | 47,24 | AVRIL | 17.7 | ST | - | - | - | - | - | - | **-** |
| 2798 | - | 2013 | Madagascar | AEZ1 | S29 | 1780 | -19,99 | 47,24 | AVRIL | 17.7 | ST | - | - | - | - | - | - | **-** |
| 2799 | - | 2013 | Madagascar | AEZ1 | S29 | 1780 | -19,99 | 47,24 | AVRIL | 17.7 | ST | - | - | - | - | - | - | **-** |
| 2800 | - | 2013 | Madagascar | AEZ1 | S29 | 1780 | -19,99 | 47,24 | AVRIL | 17.7 | ST | - | - | - | - | - | - | **-** |
| 2801 | - | 2013 | Madagascar | AEZ1 | S29 | 1780 | -19,99 | 47,24 | AVRIL | 17.7 | ST | - | - | - | - | - | - | **-** |
| 2802 | - | 2013 | Madagascar | AEZ1 | S29 | 1780 | -19,99 | 47,24 | AVRIL | 17.7 | ST | - | - | - | - | - | - | **-** |
| 2803 | - | 2013 | Madagascar | AEZ1 | S29 | 1780 | -19,99 | 47,24 | AVRIL | 17.7 | ST | - | - | - | - | - | - | **-** |
| 2804 | - | 2013 | Madagascar | AEZ1 | S29 | 1780 | -19,99 | 47,24 | AVRIL | 17.7 | ST | - | - | - | - | - | - | **-** |
| 2805 | - | 2013 | Madagascar | AEZ1 | S29 | 1780 | -19,99 | 47,24 | AVRIL | 17.7 | ST | - | - | - | - | - | - | **-** |
| 2806 | - | 2013 | Madagascar | AEZ1 | S29 | 1780 | -19,99 | 47,24 | AVRIL | 17.7 | ST | - | - | - | - | - | - | **-** |
| 2807 | - | 2013 | Madagascar | AEZ1 | S29 | 1780 | -19,99 | 47,24 | AVRIL | 17.7 | ST | - | - | - | - | - | - | **-** |
| 2808 | - | 2013 | Madagascar | AEZ1 | S29 | 1780 | -19,99 | 47,24 | AVRIL | 17.7 | ST | - | - | - | - | - | - | **-** |
| 2809 | - | 2013 | Madagascar | AEZ1 | S29 | 1780 | -19,99 | 47,24 | AVRIL | 17.7 | ST | - | - | - | - | - | - | **-** |
| 2810 | - | 2013 | Madagascar | AEZ1 | S29 | 1780 | -19,99 | 47,24 | AVRIL | 17.7 | ST | - | - | - | - | - | - | **-** |
| 2811 | - | 2013 | Madagascar | AEZ1 | S29 | 1780 | -19,99 | 47,24 | AVRIL | 17.7 | ST | - | - | - | - | - | - | **-** |
| 2812 | - | 2013 | Madagascar | AEZ1 | S29 | 1780 | -19,99 | 47,24 | AVRIL | 17.7 | ST | - | - | - | - | - | - | **-** |
| 2813 | - | 2013 | Madagascar | AEZ1 | S29 | 1780 | -19,99 | 47,24 | AVRIL | 17.7 | ST | - | - | - | - | - | - | **-** |
| 2814 | - | 2013 | Madagascar | AEZ1 | S31 | 1770 | -19,75 | 47,26 | AVRIL | 17.7 | ST | - | - | - | - | - | - | **-** |
| 2815 | - | 2013 | Madagascar | AEZ1 | S31 | 1770 | -19,75 | 47,26 | AVRIL | 17.7 | ST | - | - | - | - | - | - | **-** |
| 2816 | - | 2013 | Madagascar | AEZ1 | S31 | 1770 | -19,75 | 47,26 | AVRIL | 17.7 | ST | - | - | - | - | - | - | **-** |
| 2817 | - | 2013 | Madagascar | AEZ1 | S31 | 1770 | -19,75 | 47,26 | AVRIL | 17.7 | ST | - | - | - | - | - | - | **-** |
| 2818 | - | 2013 | Madagascar | AEZ1 | S31 | 1770 | -19,75 | 47,26 | AVRIL | 17.7 | ST | - | - | - | - | - | - | **-** |
| 2819 | - | 2013 | Madagascar | AEZ1 | S31 | 1770 | -19,75 | 47,26 | AVRIL | 17.7 | ST | - | - | - | - | - | - | **-** |
| 2820 | - | 2013 | Madagascar | AEZ1 | S31 | 1770 | -19,75 | 47,26 | AVRIL | 17.7 | ST | - | - | - | - | - | - | **-** |
| 2821 | - | 2013 | Madagascar | AEZ1 | S31 | 1770 | -19,75 | 47,26 | AVRIL | 17.7 | ST | - | - | - | - | - | - | **-** |
| 2822 | - | 2013 | Madagascar | AEZ1 | S31 | 1770 | -19,75 | 47,26 | AVRIL | 17.7 | ST | - | - | - | - | - | - | **-** |
| 2823 | - | 2013 | Madagascar | AEZ1 | S31 | 1770 | -19,75 | 47,26 | AVRIL | 17.7 | ST | - | - | - | - | - | - | **-** |
| 2824 | - | 2013 | Madagascar | AEZ1 | S31 | 1770 | -19,75 | 47,26 | AVRIL | 17.7 | ST | - | - | - | - | - | - | **-** |
| 2825 | - | 2013 | Madagascar | AEZ1 | S31 | 1770 | -19,75 | 47,26 | AVRIL | 17.7 | ST | - | - | - | - | - | - | **-** |
| 2826 | - | 2013 | Madagascar | AEZ1 | S31 | 1770 | -19,75 | 47,26 | AVRIL | 17.7 | ST | - | - | - | - | - | - | **-** |
| 2827 | - | 2013 | Madagascar | AEZ1 | S31 | 1770 | -19,75 | 47,26 | AVRIL | 17.7 | ST | - | - | - | - | - | - | **-** |
| 2828 | - | 2013 | Madagascar | AEZ1 | S31 | 1770 | -19,75 | 47,26 | AVRIL | 17.7 | ST | - | - | - | - | - | - | **-** |
| 2829 | - | 2013 | Madagascar | AEZ1 | S31 | 1770 | -19,75 | 47,26 | AVRIL | 17.7 | ST | - | - | - | - | - | - | **-** |
| 2830 | - | 2013 | Madagascar | AEZ1 | S31 | 1770 | -19,75 | 47,26 | AVRIL | 17.7 | ST | - | - | - | - | - | - | **-** |
| 2831 | - | 2013 | Madagascar | AEZ1 | S31 | 1770 | -19,75 | 47,26 | AVRIL | 17.7 | ST | - | - | - | - | - | - | **-** |
| 2832 | - | 2013 | Madagascar | AEZ1 | S30 | 1738 | -19,78 | 47,27 | AVRIL | 17.7 | ST | - | - | - | - | - | - | **-** |
| 2833 | - | 2013 | Madagascar | AEZ1 | S30 | 1738 | -19,78 | 47,27 | AVRIL | 17.7 | ST | - | - | - | - | - | - | **-** |
| 2834 | - | 2013 | Madagascar | AEZ1 | S30 | 1738 | -19,78 | 47,27 | AVRIL | 17.7 | ST | - | - | - | - | - | - | **-** |
| 2835 | - | 2013 | Madagascar | AEZ1 | S30 | 1738 | -19,78 | 47,27 | AVRIL | 17.7 | ST | - | - | - | - | - | - | **-** |
| 2836 | - | 2013 | Madagascar | AEZ1 | S30 | 1738 | -19,78 | 47,27 | AVRIL | 17.7 | ST | - | - | - | - | - | - | **-** |
| 2837 | - | 2013 | Madagascar | AEZ1 | S30 | 1738 | -19,78 | 47,27 | AVRIL | 17.7 | ST | - | - | - | - | - | - | **-** |
| 2838 | - | 2013 | Madagascar | AEZ1 | S30 | 1738 | -19,78 | 47,27 | AVRIL | 17.7 | ST | - | - | - | - | - | - | **-** |
| 2839 | - | 2013 | Madagascar | AEZ1 | S30 | 1738 | -19,78 | 47,27 | AVRIL | 17.7 | ST | - | - | - | - | - | - | **-** |
| 2840 | - | 2013 | Madagascar | AEZ1 | S30 | 1738 | -19,78 | 47,27 | AVRIL | 17.7 | ST | - | - | - | - | - | - | **-** |
| 2841 | - | 2013 | Madagascar | AEZ1 | S30 | 1738 | -19,78 | 47,27 | AVRIL | 17.7 | ST | - | - | - | - | - | - | **-** |
| 2842 | - | 2013 | Madagascar | AEZ1 | S30 | 1738 | -19,78 | 47,27 | AVRIL | 17.7 | ST | - | - | - | - | - | - | **-** |
| 2843 | - | 2013 | Madagascar | AEZ1 | S30 | 1738 | -19,78 | 47,27 | AVRIL | 17.7 | ST | - | - | - | - | - | - | **-** |
| 2844 | - | 2013 | Madagascar | AEZ1 | S30 | 1738 | -19,78 | 47,27 | AVRIL | 17.7 | ST | - | - | - | - | - | - | **-** |
| 2845 | - | 2013 | Madagascar | AEZ1 | S30 | 1738 | -19,78 | 47,27 | AVRIL | 17.7 | ST | - | - | - | - | - | - | **-** |
| 2846 | - | 2013 | Madagascar | AEZ1 | S30 | 1738 | -19,78 | 47,27 | AVRIL | 17.7 | ST | - | - | - | - | - | - | **-** |
| 2847 | - | 2013 | Madagascar | AEZ1 | S30 | 1738 | -19,78 | 47,27 | AVRIL | 17.7 | ST | - | - | - | - | - | - | **-** |
| 2848 | - | 2013 | Madagascar | AEZ1 | S30 | 1738 | -19,78 | 47,27 | AVRIL | 17.7 | ST | - | - | - | - | - | - | **-** |
| 2849 | - | 2013 | Madagascar | AEZ1 | S30 | 1738 | -19,78 | 47,27 | AVRIL | 17.7 | ST | - | - | - | - | - | - | **-** |
| 2850 | - | 2013 | Madagascar | AEZ1 | S30 | 1738 | -19,78 | 47,27 | AVRIL | 17.7 | ST | - | - | - | - | - | - | **-** |
| 2851 | - | 2013 | Madagascar | AEZ1 | S30 | 1738 | -19,78 | 47,27 | AVRIL | 17.7 | ST | - | - | - | - | - | - | **-** |
| 2852 | - | 2013 | Madagascar | AEZ1 | S28 | 1730 | -19,82 | 47,27 | AVRIL | 17.7 | ST | - | RS2-MLVA9 | 4-4-6-13-11-4-9-7-8 | MTII-8 | CCII-A | CLII-1 | CMGII |
| 2853 | - | 2013 | Madagascar | AEZ1 | S28 | 1730 | -19,82 | 47,27 | AVRIL | 17.7 | ST | - | RS2-MLVA9 | 4-5-6-13-11-4-9-7-8 | MTII-9 | CCII-A | CLII-1 | CMGII |
| 2854 | - | 2013 | Madagascar | AEZ1 | S28 | 1730 | -19,82 | 47,27 | AVRIL | 17.7 | ST | - | RS2-MLVA9 | 4-5-6-13-11-4-9-7-8 | MTII-9 | CCII-A | CLII-1 | CMGII |
| 2856 | - | 2013 | Madagascar | AEZ1 | S28 | 1730 | -19,82 | 47,27 | AVRIL | 17.7 | ST | - | RS2-MLVA9 | 4-6-6-13-11-4-9-7-10 | MTII-3 | CCII-A | CLII-1 | CMGII |
| 2857 | - | 2013 | Madagascar | AEZ1 | S28 | 1730 | -19,82 | 47,27 | AVRIL | 17.7 | ST | - | RS2-MLVA9 | 4-6-6-13-11-4-9-7-10 | MTII-3 | CCII-A | CLII-1 | CMGII |
| 2858 | - | 2013 | Madagascar | AEZ1 | S28 | 1730 | -19,82 | 47,27 | AVRIL | 17.7 | ST | - | RS2-MLVA9 | 4-5-6-13-11-4-9-7-8 | MTII-9 | CCII-A | CLII-1 | CMGII |
| 2859 | - | 2013 | Madagascar | AEZ1 | S28 | 1730 | -19,82 | 47,27 | AVRIL | 17.7 | ST | - | RS2-MLVA9 | 4-4-6-13-11-4-9-7-8 | MTII-8 | CCII-A | CLII-1 | CMGII |
| 2860 | - | 2013 | Madagascar | AEZ1 | S28 | 1730 | -19,82 | 47,27 | AVRIL | 17.7 | ST | - | RS2-MLVA9 | 4-5-6-13-11-4-9-7-8 | MTII-9 | CCII-A | CLII-1 | CMGII |
| 2861 | - | 2013 | Madagascar | AEZ1 | S28 | 1730 | -19,82 | 47,27 | AVRIL | 17.7 | ST | - | RS2-MLVA9 | 4-5-6-13-11-4-9-7-8 | MTII-9 | CCII-A | CLII-1 | CMGII |
| 2862 | - | 2013 | Madagascar | AEZ1 | S28 | 1730 | -19,82 | 47,27 | AVRIL | 17.7 | ST | - | RS2-MLVA9 | 4-5-6-13-11-4-9-7-8 | MTII-9 | CCII-A | CLII-1 | CMGII |
| 2863 | - | 2013 | Madagascar | AEZ1 | S28 | 1730 | -19,82 | 47,27 | AVRIL | 17.7 | ST | - | RS2-MLVA9 | 4-6-6-13-11-4-9-7-10 | MTII-3 | CCII-A | CLII-1 | CMGII |
| 2864 | - | 2013 | Madagascar | AEZ1 | S28 | 1730 | -19,82 | 47,27 | AVRIL | 17.7 | ST | - | RS2-MLVA9 | 4-5-6-13-11-4-9-7-8 | MTII-9 | CCII-A | CLII-1 | CMGII |
| 2865 | - | 2013 | Madagascar | AEZ1 | S28 | 1730 | -19,82 | 47,27 | AVRIL | 17.7 | ST | - | RS2-MLVA9 | 1-5-6-13-11-1-9-7-8 | MTII-7 | CCII-A | CLII-1 | CMGII |
| 2866 | - | 2013 | Madagascar | AEZ1 | S28 | 1730 | -19,82 | 47,27 | AVRIL | 17.7 | ST | - | RS2-MLVA9 | 4-4-6-13-11-4-9-7-8 | MTII-8 | CCII-A | CLII-1 | CMGII |
| 2867 | - | 2013 | Madagascar | AEZ1 | S28 | 1730 | -19,82 | 47,27 | AVRIL | 17.7 | ST | - | RS2-MLVA9 | 4-5-6-13-11-4-9-7-8 | MTII-9 | CCII-A | CLII-1 | CMGII |
| 2868 | - | 2013 | Madagascar | AEZ1 | S28 | 1730 | -19,82 | 47,27 | AVRIL | 17.7 | ST | - | RS2-MLVA9 | 4-4-6-13-11-4-9-7-8 | MTII-8 | CCII-A | CLII-1 | CMGII |
| 2869 | - | 2013 | Madagascar | AEZ1 | S28 | 1730 | -19,82 | 47,27 | AVRIL | 17.7 | ST | - | RS2-MLVA9 | 4-6-6-13-11-4-9-7-10 | MTII-3 | CCII-A | CLII-1 | CMGII |
| 2870 | - | 2013 | Madagascar | AEZ1 | S28 | 1730 | -19,82 | 47,27 | AVRIL | 17.7 | ST | - | RS2-MLVA9 | 4-6-6-13-11-4-9-7-10 | MTII-3 | CCII-A | CLII-1 | CMGII |
| 2871 | - | 2013 | Madagascar | AEZ1 | S28 | 1730 | -19,82 | 47,27 | AVRIL | 17.7 | ST | 1 | RS2-MLVA9 | 4-4-6-13-11-4-9-7-8 | MTII-8 | CCII-A | CLII-1 | CSEQ, CMGII |
| 2872 | - | 2013 | Madagascar | AEZ1 | S28 | 1730 | -19,82 | 47,27 | AVRIL | 17.7 | ST | - | RS2-MLVA9 | 4-4-6-13-11-4-9-7-8 | MTII-8 | CCII-A | CLII-1 | CMGII |
| 2873 | - | 2013 | Madagascar | AEZ1 | S28 | 1730 | -19,82 | 47,27 | AVRIL | 17.7 | ST | - | RS2-MLVA9 | 4-6-6-13-12-4-9-7-10 | MTII-17 | CCII-A | CLII-1 | CMGII |
| 2874 | - | 2013 | Madagascar | AEZ1 | S28 | 1730 | -19,82 | 47,27 | AVRIL | 17.7 | ST | - | RS2-MLVA9 | 4-4-6-13-11-4-9-7-8 | MTII-8 | CCII-A | CLII-1 | CMGII |
| 2875 | - | 2013 | Madagascar | AEZ1 | S27 | 1725 | -19,82 | 47,27 | AVRIL | 17.7 | ST | - | RS2-MLVA9 | 1-6-6-13-11-1-9-7-10 | MTII-1 | CCII-A | CLII-1 | CMGII |
| 2876 | - | 2013 | Madagascar | AEZ1 | S27 | 1725 | -19,85 | 47,27 | AVRIL | 17.7 | ST | - | RS2-MLVA9 | 4-6-6-13-11-4-9-7-10 | MTII-3 | CCII-A | CLII-2 | CMGII |
| 2877 | - | 2013 | Madagascar | AEZ1 | S27 | 1725 | -19,85 | 47,27 | AVRIL | 17.7 | ST | - | RS2-MLVA9 | 4-6-6-13-11-4-9-7-10 | MTII-3 | CCII-A | CLII-2 | CMGII |
| 2878 | - | 2013 | Madagascar | AEZ1 | S27 | 1725 | -19,85 | 47,27 | AVRIL | 17.7 | ST | - | RS2-MLVA9 | 4-6-6-13-11-4-9-7-10 | MTII-3 | CCII-A | CLII-2 | CMGII |
| 2879 | - | 2013 | Madagascar | AEZ1 | S27 | 1725 | -19,85 | 47,27 | AVRIL | 17.7 | ST | - | RS2-MLVA9 | 4-5-6-13-11-4-9-7-8 | MTII-9 | CCII-A | CLII-2 | CMGII |
| 2880 | - | 2013 | Madagascar | AEZ1 | S27 | 1725 | -19,85 | 47,27 | AVRIL | 17.7 | ST | - | RS2-MLVA9 | 1-6-6-13-11-1-9-7-10 | MTII-1 | CCII-A | CLII-2 | CMGII |
| 2881 | - | 2013 | Madagascar | AEZ1 | S27 | 1725 | -19,85 | 47,27 | AVRIL | 17.7 | ST | 1 | RS2-MLVA9 | 4-4-6-13-11-4-9-7-8 | MTII-8 | CCII-A | CLII-2 | CSEQ, CMGII |
| 2882 | - | 2013 | Madagascar | AEZ1 | S27 | 1725 | -19,85 | 47,27 | AVRIL | 17.7 | ST | - | RS2-MLVA9 | 4-6-6-13-12-4-9-7-10 | MTII-17 | CCII-A | CLII-2 | CMGII |
| 2883 | - | 2013 | Madagascar | AEZ1 | S27 | 1725 | -19,85 | 47,27 | AVRIL | 17.7 | ST | - | RS2-MLVA9 | 4-6-6-13-11-4-9-7-10 | MTII-3 | CCII-A | CLII-2 | CMGII |
| 2885 | - | 2013 | Madagascar | AEZ1 | S27 | 1725 | -19,85 | 47,27 | AVRIL | 17.7 | ST | - | RS2-MLVA9 | 4-6-6-13-12-4-9-7-10 | MTII-17 | CCII-A | CLII-2 | CMGII |
| 2886 | - | 2013 | Madagascar | AEZ1 | S27 | 1725 | -19,85 | 47,27 | AVRIL | 17.7 | ST | - | RS2-MLVA9 | 4-6-6-13-11-4-9-7-10 | MTII-3 | CCII-A | CLII-2 | CMGII |
| 2887 | - | 2013 | Madagascar | AEZ1 | S27 | 1725 | -19,85 | 47,27 | AVRIL | 17.7 | ST | - | RS2-MLVA9 | 4-3-6-13-11-4-9-7-10 | MTII-21 | CCII-A | CLII-2 | CMGII |
| 2888 | - | 2013 | Madagascar | AEZ1 | S27 | 1725 | -19,85 | 47,27 | AVRIL | 17.7 | ST | - | RS2-MLVA9 | 4-7-6-13-11-4-9-7-10 | MTII-5 | CCII-A | CLII-2 | CMGII |
| 2889 | - | 2013 | Madagascar | AEZ1 | S27 | 1725 | -19,85 | 47,27 | AVRIL | 17.7 | ST | - | RS2-MLVA9 | 4-6-6-13-11-4-9-7-10 | MTII-3 | CCII-A | CLII-2 | CMGII |
| 2890 | - | 2013 | Madagascar | AEZ1 | S27 | 1725 | -19,85 | 47,27 | AVRIL | 17.7 | ST | - | RS2-MLVA9 | 1-6-6-13-11-1-9-7-10 | MTII-1 | CCII-A | CLII-2 | CMGII |
| 2891 | - | 2013 | Madagascar | AEZ1 | S27 | 1725 | -19,85 | 47,27 | AVRIL | 17.7 | ST | - | RS2-MLVA9 | 4-4-6-13-11-4-9-7-8 | MTII-8 | CCII-A | CLII-2 | CMGII |
| 2892 | - | 2013 | Madagascar | AEZ1 | S27 | 1725 | -19,85 | 47,27 | AVRIL | 17.7 | ST | - | RS2-MLVA9 | 4-5-6-13-11-4-9-7-8 | MTII-9 | CCII-A | CLII-2 | CMGII |
| 2893 | - | 2013 | Madagascar | AEZ1 | S27 | 1725 | -19,85 | 47,27 | AVRIL | 17.7 | ST | - | RS2-MLVA9 | 4-5-6-13-11-4-9-7-12 | MTII-6 | CCII-A | CLII-2 | CMGII |
| 2894 | - | 2013 | Madagascar | AEZ1 | S27 | 1725 | -19,85 | 47,27 | AVRIL | 17.7 | ST | - | RS2-MLVA9 | 4-6-6-13-11-4-9-7-10 | MTII-3 | CCII-A | CLII-2 | CMGII |
| 2895 | - | 2013 | Madagascar | AEZ1 | S32 | 1797 | -19,95 | 47,21 | AVRIL | 17.7 | ST | - | - | - | - | - | - | **-** |
| 2896 | - | 2013 | Madagascar | AEZ1 | S32 | 1797 | -19,95 | 47,21 | AVRIL | 17.7 | ST | - | - | - | - | - | - | **-** |
| 2898 | - | 2013 | Madagascar | AEZ1 | S32 | 1797 | -19,95 | 47,21 | AVRIL | 17.7 | ST | - | - | - | - | - | - | **-** |
| 2899 | - | 2013 | Madagascar | AEZ1 | S32 | 1797 | -19,95 | 47,21 | AVRIL | 17.7 | ST | - | - | - | - | - | - | **-** |
| 2900 | - | 2013 | Madagascar | AEZ1 | S32 | 1797 | -19,95 | 47,21 | AVRIL | 17.7 | ST | - | - | - | - | - | - | **-** |
| 2901 | - | 2013 | Madagascar | AEZ1 | S32 | 1797 | -19,95 | 47,21 | AVRIL | 17.7 | ST | - | - | - | - | - | - | **-** |
| 2902 | - | 2013 | Madagascar | AEZ1 | S32 | 1797 | -19,95 | 47,21 | AVRIL | 17.7 | ST | - | - | - | - | - | - | **-** |
| 2903 | - | 2013 | Madagascar | AEZ1 | S32 | 1797 | -19,95 | 47,21 | AVRIL | 17.7 | ST | - | - | - | - | - | - | **-** |
| 2904 | - | 2013 | Madagascar | AEZ1 | S32 | 1797 | -19,95 | 47,21 | AVRIL | 17.7 | ST | - | - | - | - | - | - | **-** |
| 2905 | - | 2013 | Madagascar | AEZ1 | S32 | 1797 | -19,95 | 47,21 | AVRIL | 17.7 | ST | - | - | - | - | - | - | **-** |
| 2906 | - | 2013 | Madagascar | AEZ1 | S32 | 1797 | -19,95 | 47,21 | AVRIL | 17.7 | ST | - | - | - | - | - | - | **-** |
| 2907 | - | 2013 | Madagascar | AEZ1 | S32 | 1797 | -19,95 | 47,21 | AVRIL | 17.7 | ST | - | - | - | - | - | - | **-** |
| 2908 | - | 2013 | Madagascar | AEZ1 | S32 | 1797 | -19,95 | 47,21 | AVRIL | 17.7 | ST | - | - | - | - | - | - | **-** |
| 2909 | - | 2013 | Madagascar | AEZ1 | S32 | 1797 | -19,95 | 47,21 | AVRIL | 17.7 | ST | - | - | - | - | - | - | **-** |
| 2911 | - | 2013 | Madagascar | AEZ1 | S32 | 1797 | -19,95 | 47,21 | AVRIL | 17.7 | ST | - | - | - | - | - | - | **-** |
| 2912 | - | 2013 | Madagascar | AEZ1 | S32 | 1797 | -19,95 | 47,21 | AVRIL | 17.7 | ST | - | - | - | - | - | - | **-** |
| 2913 | - | 2013 | Madagascar | AEZ1 | S32 | 1797 | -19,95 | 47,21 | AVRIL | 17.7 | ST | - | - | - | - | - | - | **-** |
| 2914 | - | 2013 | Madagascar | AEZ1 | S32 | 1797 | -19,95 | 47,21 | AVRIL | 17.7 | ST | - | - | - | - | - | - | **-** |
| 2915 | - | 2013 | Madagascar | AEZ1 | S42 | 1392 | -20,07 | 47,26 | AVRIL | 17.7 | ST | - | RS2-MLVA9 | 4-6-6-13-11-4-9-7-10 | MTII-3 | CCII-A | CLII-2 | CMGII |
| 2916 | - | 2013 | Madagascar | AEZ1 | S42 | 1392 | -20,07 | 47,26 | AVRIL | 17.7 | ST | - | RS2-MLVA9 | 4-6-6-14-11-4-9-8-10 | MTII-24 | CCII-A | CLII-2 | CMGII |
| 2917 | - | 2013 | Madagascar | AEZ1 | S42 | 1392 | -20,07 | 47,26 | AVRIL | 17.7 | ST | - | RS2-MLVA9 | 4-6-6-13-11-4-9-7-10 | MTII-3 | CCII-A | CLII-2 | CMGII |
| 2918 | - | 2013 | Madagascar | AEZ1 | S42 | 1392 | -20,07 | 47,26 | AVRIL | 17.7 | ST | - | RS2-MLVA9 | 4-6-6-14-11-4-9-7-10 | MTII-4 | CCII-A | CLII-2 | CMGII |
| 2919 | - | 2013 | Madagascar | AEZ1 | S42 | 1392 | -20,07 | 47,26 | AVRIL | 17.7 | ST | - | RS2-MLVA9 | 4-6-6-13-11-4-9-7-10 | MTII-3 | CCII-A | CLII-2 | CMGII |
| 2920 | - | 2013 | Madagascar | AEZ1 | S42 | 1392 | -20,07 | 47,26 | AVRIL | 17.7 | ST | - | RS2-MLVA9 | 4-6-6-13-11-4-9-7-10 | MTII-3 | CCII-A | CLII-2 | CMGII |
| 2921 | - | 2013 | Madagascar | AEZ1 | S42 | 1392 | -20,07 | 47,26 | AVRIL | 17.7 | ST | - | RS2-MLVA9 | 4-6-6-14-11-4-9-7-10 | MTII-4 | CCII-A | CLII-2 | CMGII |
| 2923 | - | 2013 | Madagascar | AEZ1 | S42 | 1392 | -20,07 | 47,26 | AVRIL | 17.7 | ST | - | RS2-MLVA9 | 4-6-6-13-11-4-9-7-10 | MTII-3 | CCII-A | CLII-2 | CMGII |
| 2924 | - | 2013 | Madagascar | AEZ1 | S42 | 1392 | -20,07 | 47,26 | AVRIL | 17.7 | ST | - | RS2-MLVA9 | 4-6-6-13-11-4-9-7-10 | MTII-3 | CCII-A | CLII-2 | CMGII |
| 2926 | - | 2013 | Madagascar | AEZ1 | S42 | 1392 | -20,07 | 47,26 | AVRIL | 17.7 | ST | - | - | - | - | - | - | **-** |
| 2927 | - | 2013 | Madagascar | AEZ1 | S42 | 1392 | -20,07 | 47,26 | AVRIL | 17.7 | ST | - | RS2-MLVA9 | 4-6-6-13-11-4-9-7-9 | MTII-15 | CCII-A | CLII-2 | CMGII |
| 2928 | - | 2013 | Madagascar | AEZ1 | S42 | 1392 | -20,07 | 47,26 | AVRIL | 17.7 | ST | - | RS2-MLVA9 | 4-6-6-13-11-4-9-7-10 | MTII-3 | CCII-A | CLII-2 | CMGII |
| 2929 | - | 2013 | Madagascar | AEZ1 | S42 | 1392 | -20,07 | 47,26 | AVRIL | 17.7 | ST | - | - | - | - | - | - | **-** |
| 2930 | - | 2013 | Madagascar | AEZ1 | S40 | 1363 | -20,08 | 47,06 | AVRIL | 17.7 | ST | - | - | - | - | - | - | **-** |
| 2931 | - | 2013 | Madagascar | AEZ1 | S40 | 1363 | -20,08 | 47,06 | AVRIL | 17.7 | ST | - | - | - | - | - | - | **-** |
| 2932 | - | 2013 | Madagascar | AEZ1 | S40 | 1363 | -20,08 | 47,06 | AVRIL | 17.7 | ST | - | - | - | - | - | - | **-** |
| 2933 | - | 2013 | Madagascar | AEZ1 | S40 | 1363 | -20,08 | 47,06 | AVRIL | 17.7 | ST | - | - | - | - | - | - | **-** |
| 2934 | - | 2013 | Madagascar | AEZ1 | S40 | 1363 | -20,08 | 47,06 | AVRIL | 17.7 | ST | - | - | - | - | - | - | **-** |
| 2935 | - | 2013 | Madagascar | AEZ1 | S40 | 1363 | -20,08 | 47,06 | AVRIL | 17.7 | ST | - | - | - | - | - | - | **-** |
| 2936 | - | 2013 | Madagascar | AEZ1 | S40 | 1363 | -20,08 | 47,06 | AVRIL | 17.7 | ST | - | - | - | - | - | - | **-** |
| 2937 | - | 2013 | Madagascar | AEZ1 | S40 | 1363 | -20,08 | 47,06 | AVRIL | 17.7 | ST | - | - | - | - | - | - | **-** |
| 2938 | - | 2013 | Madagascar | AEZ1 | S40 | 1363 | -20,08 | 47,06 | AVRIL | 17.7 | ST | - | - | - | - | - | - | **-** |
| 2939 | - | 2013 | Madagascar | AEZ1 | S41 | 1364 | -20,08 | 47,05 | AVRIL | 17.7 | ST | - | - | - | - | - | - | **-** |
| 2940 | - | 2013 | Madagascar | AEZ1 | S41 | 1364 | -20,08 | 47,05 | AVRIL | 17.7 | ST | - | - | - | - | - | - | **-** |
| 2941 | - | 2013 | Madagascar | AEZ1 | S41 | 1364 | -20,08 | 47,05 | AVRIL | 17.7 | ST | - | - | - | - | - | - | **-** |
| 2942 | - | 2013 | Madagascar | AEZ1 | S41 | 1364 | -20,08 | 47,05 | AVRIL | 17.7 | ST | - | - | - | - | - | - | **-** |
| 2943 | - | 2013 | Madagascar | AEZ1 | S41 | 1364 | -20,08 | 47,05 | AVRIL | 17.7 | ST | - | - | - | - | - | - | **-** |
| 2944 | - | 2013 | Madagascar | AEZ1 | S41 | 1364 | -20,08 | 47,05 | AVRIL | 17.7 | ST | - | - | - | - | - | - | **-** |
| 2945 | - | 2013 | Madagascar | AEZ1 | S41 | 1364 | -20,08 | 47,05 | AVRIL | 17.7 | ST | 1 | RS2-MLVA9 | 4-5-6-13-11-4-9-7-8 | MTII-9 | CCII-A | CLII-1 | CSEQ, CMGII |
| 2946 | - | 2013 | Madagascar | AEZ1 | S41 | 1364 | -20,08 | 47,05 | AVRIL | 17.7 | ST | - | - | - | - | - | - | **-** |
| 2947 | - | 2013 | Madagascar | AEZ1 | S41 | 1364 | -20,08 | 47,05 | AVRIL | 17.7 | ST | - | - | - | - | - | - | **-** |
| 2948 | - | 2013 | Madagascar | AEZ1 | S41 | 1364 | -20,08 | 47,05 | AVRIL | 17.7 | ST | - | - | - | - | - | - | **-** |
| 2949 | - | 2013 | Madagascar | AEZ1 | S41 | 1364 | -20,08 | 47,05 | AVRIL | 17.7 | ST | - | - | - | - | - | - | **-** |
| 2950 | - | 2013 | Madagascar | AEZ1 | S41 | 1364 | -20,08 | 47,05 | AVRIL | 17.7 | ST | - | - | - | - | - | - | **-** |
| 2951 | - | 2013 | Madagascar | AEZ1 | S41 | 1364 | -20,08 | 47,05 | AVRIL | 17.7 | ST | - | - | - | - | - | - | **-** |
| 2952 | - | 2013 | Madagascar | AEZ1 | S41 | 1364 | -20,08 | 47,05 | AVRIL | 17.7 | ST | - | - | - | - | - | - | **-** |
| 2953 | - | 2013 | Madagascar | AEZ1 | S41 | 1364 | -20,08 | 47,05 | AVRIL | 17.7 | ST | - | - | - | - | - | - | **-** |
| 2954 | - | 2013 | Madagascar | AEZ1 | S41 | 1364 | -20,08 | 47,05 | AVRIL | 17.7 | ST | - | - | - | - | - | - | **-** |
| 2955 | - | 2013 | Madagascar | AEZ1 | S41 | 1364 | -20,08 | 47,05 | AVRIL | 17.7 | ST | - | RS2-MLVA9 | 4-5-6-13-11-4-9-7-8 | MTII-9 | CCII-A | CLII-1 | CMGII |
| 2956 | - | 2013 | Madagascar | AEZ1 | S41 | 1364 | -20,08 | 47,05 | AVRIL | 17.7 | ST | - | - | - | - | - | - | **-** |
| 2957 | - | 2013 | Madagascar | AEZ1 | S41 | 1364 | -20,08 | 47,05 | AVRIL | 17.7 | ST | - | - | - | - | - | - | **-** |
| 2958 | - | 2013 | Madagascar | AEZ1 | S41 | 1364 | -20,08 | 47,05 | AVRIL | 17.7 | ST | - | - | - | - | - | - | **-** |
| 2959 | - | 2013 | Madagascar | AEZ1 | S39 | 1357 | -20,07 | 47,29 | AVRIL | 17.7 | ST | - | - | - | - | - | - | **-** |
| 2960 | - | 2013 | Madagascar | AEZ1 | S39 | 1357 | -20,07 | 47,29 | AVRIL | 17.7 | ST | - | - | - | - | - | - | **-** |
| 2961 | - | 2013 | Madagascar | AEZ1 | S39 | 1357 | -20,07 | 47,29 | AVRIL | 17.7 | ST | - | - | - | - | - | - | **-** |
| 2962 | - | 2013 | Madagascar | AEZ1 | S39 | 1357 | -20,07 | 47,29 | AVRIL | 17.7 | ST | - | - | - | - | - | - | **-** |
| 2964 | - | 2013 | Madagascar | AEZ1 | S39 | 1357 | -20,07 | 47,29 | AVRIL | 17.7 | ST | - | - | - | - | - | - | **-** |
| 2965 | - | 2013 | Madagascar | AEZ1 | S39 | 1357 | -20,07 | 47,29 | AVRIL | 17.7 | ST | - | - | - | - | - | - | **-** |
| 2969 | - | 2013 | Madagascar | AEZ1 | S39 | 1357 | -20,07 | 47,29 | AVRIL | 17.7 | ST | - | - | - | - | - | - | **-** |
| 2970 | - | 2013 | Madagascar | AEZ1 | S39 | 1357 | -20,07 | 47,29 | AVRIL | 17.7 | ST | - | - | - | - | - | - | **-** |
| 2971 | - | 2013 | Madagascar | AEZ1 | S39 | 1357 | -20,07 | 47,29 | AVRIL | 17.7 | ST | - | - | - | - | - | - | **-** |
| 2972 | - | 2013 | Madagascar | AEZ1 | S39 | 1357 | -20,07 | 47,29 | AVRIL | 17.7 | ST | - | - | - | - | - | - | **-** |
| 2973 | - | 2013 | Madagascar | AEZ1 | S39 | 1357 | -20,07 | 47,29 | AVRIL | 17.7 | ST | - | - | - | - | - | - | **-** |
| 2974 | - | 2013 | Madagascar | AEZ1 | S39 | 1357 | -20,07 | 47,29 | AVRIL | 17.7 | ST | - | - | - | - | - | - | **-** |
| 2978 | - | 2013 | Madagascar | AEZ1 | S39 | 1357 | -20,07 | 47,29 | AVRIL | 17.7 | ST | - | - | - | - | - | - | **-** |
| 2980 | - | 2013 | Madagascar | AEZ1 | S25 | 1650 | -19,95 | 47,21 | AVRIL | 17.7 | W | 1 | RS2-MLVA9 | 4-5-6-13-11-4-9-7-8 | MTII-9 | CCII-A | CLII-1 | CSEQ, CMGII |
| 2982 | - | 2013 | Madagascar | AEZ1 | S25 | 1650 | -19,95 | 47,21 | AVRIL | 17.7 | W | - | RS2-MLVA9 | 4-5-6-13-11-4-9-7-8 | MTII-9 | CCII-A | CLII-1 | CMGII |
| 2991 | - | 2013 | Madagascar | AEZ2 | S60 | 1604 | -19,90 | 47,05 | AVRIL | 18.3 | ST | - | - | - | - | - | - | **-** |
| 2993 | - | 2013 | Madagascar | AEZ3 | S69 | 1941 | -19,59 | 47,03 | AVRIL | 17.5 | ST | - | - | - | - | - | - | **-** |
| 2997 | - | 2013 | Madagascar | AEZ2 | S60 | 1604 | -19,90 | 47,05 | AVRIL | 18.3 | ST | - | - | - | - | - | - | **-** |
| 2998 | - | 2013 | Madagascar | AEZ1 | S31 | 1770 | -19,75 | 47,26 | AVRIL | 17.7 | ST | - | - | - | - | - | - | **-** |
| 2999 | - | 2013 | Madagascar | AEZ2 | S60 | 1604 | -19,90 | 47,05 | AVRIL | 18.3 | ST | - | - | - | - | - | - | **-** |
| 3000 | - | 2013 | Madagascar | AEZ4 | S2 | 1593 | -19,45 | 47,67 | AVRIL | 17.3 | ST | - | - | - | - | - | - | **-** |
| 3001 | - | 2013 | Madagascar | AEZ2 | S61 | 1609 | -19,98 | 47,04 | AVRIL | 18.3 | ST | - | - | - | - | - | - | **-** |
| 3002 | - | 2013 | Madagascar | AEZ4 | S3 | 1569 | -19,62 | 47,43 | AVRIL | 17.3 | ST | - | - | - | - | - | - | **-** |
| 3003 | - | 2013 | Madagascar | AEZ3 | S65 | 1929 | -19,72 | 47,05 | AVRIL | 17.5 | ST | - | - | - | - | - | - | **-** |
| 3004 | - | 2013 | Madagascar | AEZ3 | S69 | 1941 | -19,59 | 47,03 | AVRIL | 17.5 | ST | - | - | - | - | - | - | **-** |
| 3005 | - | 2013 | Madagascar | AEZ2 | S56 | 1599 | -19,87 | 47,02 | AVRIL | 18.3 | ST | - | - | - | - | - | - | **-** |
| 3006 | - | 2013 | Madagascar | AEZ3 | S66 | 1950 | -19,68 | 47,27 | AVRIL | 17.5 | ST | - | - | - | - | - | - | **-** |
| 3024 | - | 2013 | Madagascar | AEZ5 | S54 | 1313 | -19,13 | 47,61 | AVRIL | 18.7 | SL | - | - | - | - | - | - | **-** |
| 3025 | - | 2013 | Madagascar | AEZ5 | S54 | 1313 | -19,13 | 47,61 | AVRIL | 18.7 | SL | - | - | - | - | - | - | **-** |
| 3026 | - | 2013 | Madagascar | AEZ5 | S54 | 1313 | -19,13 | 47,61 | AVRIL | 18.7 | SL | - | - | - | - | - | - | **-** |
| 3027 | - | 2013 | Madagascar | AEZ5 | S54 | 1313 | -19,13 | 47,61 | AVRIL | 18.7 | SL | - | - | - | - | - | - | **-** |
| 3028 | - | 2013 | Madagascar | AEZ5 | S54 | 1313 | -19,13 | 47,61 | AVRIL | 18.7 | SL | - | - | - | - | - | - | **-** |
| 3029 | - | 2013 | Madagascar | AEZ5 | S54 | 1313 | -19,13 | 47,61 | AVRIL | 18.7 | SL | - | - | - | - | - | - | **-** |
| 3030 | - | 2013 | Madagascar | AEZ5 | S54 | 1313 | -19,13 | 47,61 | AVRIL | 18.7 | SL | - | - | - | - | - | - | **-** |
| 3031 | - | 2013 | Madagascar | AEZ5 | S54 | 1313 | -19,13 | 47,61 | AVRIL | 18.7 | SL | - | - | - | - | - | - | **-** |
| 3032 | - | 2013 | Madagascar | AEZ5 | S54 | 1313 | -19,13 | 47,61 | AVRIL | 18.7 | SL | 1 | RS2-MLVA9 | 4-6-6-14-11-4-9-7-10 | MTII-4 | CCII-A | CLII-2 | CSEQ, CMGII |
| 3033 | - | 2013 | Madagascar | AEZ5 | S54 | 1313 | -19,13 | 47,61 | AVRIL | 18.7 | SL | - | - | - | - | - | - | **-** |
| 3034 | - | 2013 | Madagascar | AEZ5 | S54 | 1313 | -19,13 | 47,61 | AVRIL | 18.7 | SL | - | - | - | - | - | - | **-** |
| 3035 | - | 2013 | Madagascar | AEZ7 | S53 | 1347 | -19,02 | 47,25 | DECEMBER | 21.9 | SL | - | RS2-MLVA9 | 4-5-6-13-11-4-9-7-8 | MTII-9 | CCII-A | CLII-1 | CMGII |
| 3036 | - | 2013 | Madagascar | AEZ7 | S53 | 1347 | -19,02 | 47,25 | DECEMBER | 21.9 | SL | - | RS2-MLVA9 | 4-5-6-13-11-4-9-7-8 | MTII-9 | CCII-A | CLII-1 | CMGII |
| 3037 | - | 2013 | Madagascar | AEZ7 | S53 | 1347 | -19,02 | 47,25 | DECEMBER | 21.9 | SL | - | RS2-MLVA9 | 4-5-6-13-11-4-9-7-12 | MTII-6 | CCII-A | CLII-1 | CMGII |
| 3039 | - | 2013 | Madagascar | AEZ7 | S53 | 1347 | -19,02 | 47,25 | DECEMBER | 21.9 | SL | - | RS2-MLVA9 | 4-5-6-13-11-4-9-7-8 | MTII-9 | CCII-A | CLII-1 | CMGII |
| 3040 | - | 2013 | Madagascar | AEZ7 | S53 | 1347 | -19,02 | 47,25 | DECEMBER | 21.9 | SL | 1 | RS2-MLVA9 | 4-5-6-13-11-4-9-7-8 | MTII-9 | CCII-A | CLII-1 | CSEQ, CMGII |
| 3041 | - | 2013 | Madagascar | AEZ7 | S53 | 1347 | -19,02 | 47,25 | DECEMBER | 21.9 | SL | - | RS2-MLVA9 | 4-5-6-13-11-4-9-7-8 | MTII-9 | CCII-A | CLII-1 | CMGII |
| 3042 | - | 2013 | Madagascar | AEZ7 | S53 | 1347 | -19,02 | 47,25 | DECEMBER | 21.9 | SL | - | RS2-MLVA9 | 4-5-6-13-11-4-9-7-8 | MTII-9 | CCII-A | CLII-1 | CMGII |
| 3043 | - | 2013 | Madagascar | AEZ7 | S53 | 1347 | -19,02 | 47,25 | DECEMBER | 21.9 | SL | - | RS2-MLVA9 | 4-6-6-13-11-4-9-7-10 | MTII-3 | CCII-A | CLII-1 | CMGII |
| 3044 | - | 2013 | Madagascar | AEZ7 | S53 | 1347 | -19,02 | 47,25 | DECEMBER | 21.9 | SL | - | RS2-MLVA9 | 4-5-6-13-11-4-9-7-12 | MTII-6 | CCII-A | CLII-1 | CMGII |
| 3045 | - | 2013 | Madagascar | AEZ7 | S53 | 1347 | -19,02 | 47,25 | DECEMBER | 21.9 | SL | - | RS2-MLVA9 | 4-5-6-13-11-4-9-7-8 | MTII-9 | CCII-A | CLII-1 | CMGII |
| 3046 | - | 2013 | Madagascar | AEZ7 | S53 | 1347 | -19,02 | 47,25 | DECEMBER | 21.9 | SL | - | RS2-MLVA9 | 4-5-6-13-11-4-9-7-8 | MTII-9 | CCII-A | CLII-1 | CMGII |
| 3047 | - | 2013 | Madagascar | AEZ7 | S53 | 1347 | -19,02 | 47,25 | DECEMBER | 21.9 | SL | - | RS2-MLVA9 | 4-5-6-13-11-4-9-7-8 | MTII-9 | CCII-A | CLII-1 | CMGII |
| 3048 | - | 2013 | Madagascar | AEZ7 | S49 | 1476 | -19,07 | 47,20 | DECEMBER | 21.9 | ST | - | - | - | - | - | - | **-** |
| 3049 | - | 2013 | Madagascar | AEZ7 | S49 | 1476 | -19,07 | 47,20 | DECEMBER | 21.9 | ST | - | - | - | - | - | - | **-** |
| 3050 | - | 2013 | Madagascar | AEZ7 | S49 | 1476 | -19,07 | 47,20 | DECEMBER | 21.9 | ST | - | - | - | - | - | - | **-** |
| 3051 | - | 2013 | Madagascar | AEZ7 | S49 | 1476 | -19,07 | 47,20 | DECEMBER | 21.9 | ST | - | - | - | - | - | - | **-** |
| 3052 | - | 2013 | Madagascar | AEZ7 | S49 | 1476 | -19,07 | 47,20 | DECEMBER | 21.9 | ST | - | - | - | - | - | - | **-** |
| 3054 | - | 2013 | Madagascar | AEZ7 | S49 | 1476 | -19,07 | 47,20 | DECEMBER | 21.9 | ST | - | - | - | - | - | - | **-** |
| 3055 | - | 2013 | Madagascar | AEZ7 | S49 | 1476 | -19,07 | 47,20 | DECEMBER | 21.9 | ST | - | - | - | - | - | - | **-** |
| 3056 | - | 2013 | Madagascar | AEZ7 | S49 | 1476 | -19,07 | 47,20 | DECEMBER | 21.9 | ST | - | - | - | - | - | - | **-** |
| 3058 | - | 2013 | Madagascar | AEZ7 | S49 | 1476 | -19,07 | 47,20 | DECEMBER | 21.9 | ST | - | - | - | - | - | - | **-** |
| 3059 | - | 2013 | Madagascar | AEZ7 | S49 | 1476 | -19,07 | 47,20 | DECEMBER | 21.9 | ST | - | - | - | - | - | - | **-** |
| 3060 | - | 2013 | Madagascar | AEZ7 | S49 | 1476 | -19,07 | 47,20 | DECEMBER | 21.9 | ST | - | - | - | - | - | - | **-** |
| 3061 | - | 2013 | Madagascar | AEZ7 | S49 | 1476 | -19,07 | 47,20 | DECEMBER | 21.9 | ST | - | - | - | - | - | - | **-** |
| 3062 | - | 2013 | Madagascar | AEZ7 | S49 | 1476 | -19,07 | 47,20 | DECEMBER | 21.9 | ST | - | - | - | - | - | - | **-** |
| 3063 | - | 2013 | Madagascar | AEZ7 | S49 | 1476 | -19,07 | 47,20 | DECEMBER | 21.9 | ST | - | - | - | - | - | - | **-** |
| 3064 | - | 2013 | Madagascar | AEZ7 | S49 | 1476 | -19,07 | 47,20 | DECEMBER | 21.9 | ST | - | - | - | - | - | - | **-** |
| 3065 | - | 2013 | Madagascar | AEZ7 | S49 | 1476 | -19,07 | 47,20 | DECEMBER | 21.9 | ST | - | - | - | - | - | - | **-** |
| 3066 | - | 2013 | Madagascar | AEZ7 | S49 | 1476 | -19,07 | 47,20 | DECEMBER | 21.9 | ST | - | - | - | - | - | - | **-** |
| 3067 | - | 2013 | Madagascar | AEZ7 | S49 | 1476 | -19,07 | 47,20 | DECEMBER | 21.9 | ST | - | - | - | - | - | - | **-** |
| 3068 | - | 2013 | Madagascar | AEZ7 | S49 | 1476 | -19,07 | 47,20 | DECEMBER | 21.9 | ST | - | - | - | - | - | - | **-** |
| 3069 | - | 2013 | Madagascar | AEZ7 | S49 | 1476 | -19,07 | 47,20 | DECEMBER | 21.9 | ST | - | - | - | - | - | - | **-** |
| 3071 | - | 2013 | Madagascar | AEZ7 | S49 | 1476 | -19,07 | 47,20 | DECEMBER | 21.9 | ST | - | - | - | - | - | - | **-** |
| 3072 | - | 2013 | Madagascar | AEZ7 | S49 | 1476 | -19,07 | 47,20 | DECEMBER | 21.9 | ST | - | - | - | - | - | - | **-** |
| 3073 | - | 2013 | Madagascar | AEZ7 | S49 | 1476 | -19,07 | 47,20 | DECEMBER | 21.9 | ST | - | - | - | - | - | - | **-** |
| 3074 | - | 2013 | Madagascar | AEZ7 | S49 | 1476 | -19,07 | 47,20 | DECEMBER | 21.9 | ST | - | - | - | - | - | - | **-** |
| 3079 | - | 2013 | Madagascar | AEZ7 | S49 | 1476 | -19,07 | 47,20 | DECEMBER | 21.9 | ST | - | - | - | - | - | - | **-** |
| 3081 | - | 2013 | Madagascar | AEZ7 | S52 | 1340 | -19,01 | 47,27 | DECEMBER | 21.9 | SL | - | - | - | - | - | - | **-** |
| 3088 | - | 2013 | Madagascar | AEZ7 | S52 | 1340 | -19,01 | 47,27 | DECEMBER | 21.9 | ST | - | - | - | - | - | - | **-** |
| 3090 | - | 2013 | Madagascar | AEZ7 | S52 | 1340 | -19,01 | 47,27 | DECEMBER | 21.9 | ST | - | - | - | - | - | - | **-** |
| 3091 | - | 2013 | Madagascar | AEZ7 | S52 | 1340 | -19,01 | 47,27 | DECEMBER | 21.9 | ST | - | - | - | - | - | - | **-** |
| 3092 | - | 2013 | Madagascar | AEZ7 | S52 | 1340 | -19,01 | 47,27 | DECEMBER | 21.9 | ST | - | - | - | - | - | - | **-** |
| 3093 | - | 2013 | Madagascar | AEZ7 | S52 | 1340 | -19,01 | 47,27 | DECEMBER | 21.9 | ST | - | - | - | - | - | - | **-** |
| 3094 | - | 2013 | Madagascar | AEZ7 | S52 | 1340 | -19,01 | 47,27 | DECEMBER | 21.9 | ST | - | - | - | - | - | - | **-** |
| 3095 | - | 2013 | Madagascar | AEZ7 | S52 | 1340 | -19,01 | 47,27 | DECEMBER | 21.9 | ST | - | - | - | - | - | - | **-** |
| 3096 | - | 2013 | Madagascar | AEZ7 | S52 | 1340 | -19,01 | 47,27 | DECEMBER | 21.9 | ST | - | - | - | - | - | - | **-** |
| 3097 | - | 2013 | Madagascar | AEZ7 | S52 | 1340 | -19,01 | 47,27 | DECEMBER | 21.9 | ST | - | - | - | - | - | - | **-** |
| 3098 | - | 2013 | Madagascar | AEZ7 | S52 | 1340 | -19,01 | 47,27 | DECEMBER | 21.9 | ST | - | - | - | - | - | - | **-** |
| 3099 | - | 2013 | Madagascar | AEZ7 | S52 | 1340 | -19,01 | 47,27 | DECEMBER | 21.9 | ST | - | - | - | - | - | - | **-** |
| 3100 | - | 2013 | Madagascar | AEZ7 | S52 | 1340 | -19,01 | 47,27 | DECEMBER | 21.9 | ST | - | - | - | - | - | - | **-** |
| 3101 | - | 2013 | Madagascar | AEZ7 | S52 | 1340 | -19,01 | 47,27 | DECEMBER | 21.9 | ST | - | - | - | - | - | - | **-** |
| 3102 | - | 2013 | Madagascar | AEZ7 | S52 | 1340 | -19,01 | 47,27 | DECEMBER | 21.9 | ST | - | - | - | - | - | - | **-** |
| 3103 | - | 2013 | Madagascar | AEZ7 | S52 | 1340 | -19,01 | 47,27 | DECEMBER | 21.9 | ST | - | - | - | - | - | - | **-** |
| 3104 | - | 2013 | Madagascar | AEZ7 | S52 | 1340 | -19,01 | 47,27 | DECEMBER | 21.9 | ST | - | - | - | - | - | - | **-** |
| 3105 | - | 2013 | Madagascar | AEZ7 | S52 | 1340 | -19,01 | 47,27 | DECEMBER | 21.9 | ST | - | - | - | - | - | - | **-** |
| 3106 | - | 2013 | Madagascar | AEZ7 | S52 | 1340 | -19,01 | 47,27 | DECEMBER | 21.9 | ST | - | - | - | - | - | - | **-** |
| 3107 | - | 2013 | Madagascar | AEZ7 | S52 | 1340 | -19,01 | 47,27 | DECEMBER | 21.9 | ST | - | - | - | - | - | - | **-** |
| 3123 | - | 2013 | Madagascar | AEZ7 | S52 | 1340 | -19,01 | 47,27 | DECEMBER | 21.9 | SL | - | RS2-MLVA9 | 4-5-6-13-11-4-9-7-8 | MTII-9 | CCII-A | CLII-1 | CMGII |
| 3132 | - | 2013 | Madagascar | AEZ6 | S9 | 1252 | -18,76 | 47,34 | DECEMBER | 20.2 | SL | 1 | - | - | - | - | - | **CSEQ** |
| 3133 | - | 2013 | Madagascar | AEZ6 | S9 | 1252 | -18,76 | 47,34 | DECEMBER | 20.2 | SL | - | - | - | - | - | - | **-** |
| 3139 | - | 2013 | Madagascar | AEZ6 | S7 | 1251 | -18,72 | 47,28 | DECEMBER | 20.2 | ST | - | - | - | - | - | - | **-** |
| 3140 | - | 2013 | Madagascar | AEZ6 | S7 | 1251 | -18,72 | 47,28 | DECEMBER | 20.2 | ST | - | - | - | - | - | - | **-** |
| 3141 | - | 2013 | Madagascar | AEZ7 | S52 | 1340 | -19,01 | 47,27 | DECEMBER | 21.9 | SL | - | - | - | - | - | - | **-** |
| 3143 | - | 2013 | Madagascar | AEZ6 | S7 | 1251 | -18,72 | 47,28 | DECEMBER | 20.2 | ST | - | - | - | - | - | - | **-** |
| 3144 | - | 2013 | Madagascar | AEZ6 | S7 | 1251 | -18,72 | 47,28 | DECEMBER | 20.2 | ST | - | - | - | - | - | - | **-** |
| 3145 | - | 2013 | Madagascar | AEZ6 | S7 | 1251 | -18,72 | 47,28 | DECEMBER | 20.2 | ST | - | - | - | - | - | - | **-** |
| 3146 | - | 2013 | Madagascar | AEZ6 | S7 | 1251 | -18,72 | 47,28 | DECEMBER | 20.2 | ST | - | - | - | - | - | - | **-** |
| 3147 | - | 2013 | Madagascar | AEZ6 | S7 | 1251 | -18,72 | 47,28 | DECEMBER | 20.2 | ST | - | - | - | - | - | - | **-** |
| 3150 | - | 2013 | Madagascar | AEZ6 | S7 | 1251 | -18,72 | 47,28 | DECEMBER | 20.2 | ST | - | - | - | - | - | - | **-** |
| 3152 | - | 2013 | Madagascar | AEZ6 | S8 | 1266 | -18,80 | 47,42 | DECEMBER | 20.2 | SL | - | RS2-MLVA9 | 4-6-6-13-11-4-9-7-12 | MTII-25 | CCII-A | CLII-1 | CMGII |
| 3153 | - | 2013 | Madagascar | AEZ6 | S8 | 1266 | -18,80 | 47,42 | DECEMBER | 20.2 | SL | - | RS2-MLVA9 | 4-5-6-13-11-4-9-7-8 | MTII-9 | CCII-A | CLII-1 | CMGII |
| 3154 | - | 2013 | Madagascar | AEZ6 | S8 | 1266 | -18,80 | 47,42 | DECEMBER | 20.2 | SL | - | RS2-MLVA9 | 3-5-6-13-11-4-9-7-8 | MTII-37 | CCII-A | CLII-1 | CMGII |
| 3155 | - | 2013 | Madagascar | AEZ6 | S8 | 1266 | -18,80 | 47,42 | DECEMBER | 20.2 | SL | - | RS2-MLVA9 | 4-5-6-13-11-4-9-7-8 | MTII-9 | CCII-A | CLII-1 | CMGII |
| 3156 | - | 2013 | Madagascar | AEZ6 | S8 | 1266 | -18,80 | 47,42 | DECEMBER | 20.2 | SL | - | RS2-MLVA9 | 4-5-6-13-11-4-9-7-8 | MTII-9 | CCII-A | CLII-1 | CMGII |
| 3157 | - | 2013 | Madagascar | AEZ6 | S8 | 1266 | -18,80 | 47,42 | DECEMBER | 20.2 | SL | 1 | RS2-MLVA9 | 4-5-6-13-11-4-9-7-8 | MTII-9 | CCII-A | CLII-1 | CSEQ, CMGII |
| 3158 | - | 2013 | Madagascar | AEZ6 | S8 | 1266 | -18,80 | 47,42 | DECEMBER | 20.2 | SL | - | RS2-MLVA9 | 4-5-6-14-11-1-9-7-8 | MTII-28 | CCII-A | CLII-1 | CMGII |
| 3160 | - | 2013 | Madagascar | AEZ6 | S8 | 1266 | -18,80 | 47,42 | DECEMBER | 20.2 | SL | - | RS2-MLVA9 | 4-5-6-13-11-4-9-7-8 | MTII-9 | CCII-A | CLII-1 | CMGII |
| 3161 | - | 2013 | Madagascar | AEZ6 | S8 | 1266 | -18,80 | 47,42 | DECEMBER | 20.2 | SL | - | RS2-MLVA9 | 4-5-6-13-11-4-9-7-8 | MTII-9 | CCII-A | CLII-1 | CMGII |
| 3166 | - | 2013 | Madagascar | AEZ6 | S8 | 1266 | -18,80 | 47,42 | DECEMBER | 20.2 | ST | - | RS2-MLVA9 | 4-5-6-13-11-4-9-7-8 | MTII-9 | CCII-A | CLII-1 | CMGII |
| 3167 | - | 2013 | Madagascar | AEZ6 | S8 | 1266 | -18,80 | 47,42 | DECEMBER | 20.2 | ST | 1 | RS2-MLVA9 | 4-5-6-13-11-4-9-7-8 | MTII-9 | CCII-A | CLII-1 | CSEQ, CMGII |
| 3169 | - | 2013 | Madagascar | AEZ6 | S8 | 1266 | -18,80 | 47,42 | DECEMBER | 20.2 | ST | - | RS2-MLVA9 | 4-5-6-13-11-4-9-7-8 | MTII-9 | CCII-A | CLII-1 | CMGII |
| 3172 | - | 2013 | Madagascar | AEZ7 | S51 | 1477 | -19,08 | 47,25 | DECEMBER | 21.9 | ST | - | - | - | - | - | - | **-** |
| 3173 | - | 2013 | Madagascar | AEZ7 | S51 | 1477 | -19,08 | 47,25 | DECEMBER | 21.9 | ST | - | - | - | - | - | - | **-** |
| 3174 | - | 2013 | Madagascar | AEZ7 | S51 | 1477 | -19,08 | 47,25 | DECEMBER | 21.9 | ST | - | - | - | - | - | - | **-** |
| 3175 | - | 2013 | Madagascar | AEZ7 | S51 | 1477 | -19,08 | 47,25 | DECEMBER | 21.9 | ST | - | RS2-MLVA9 | 4-5-6-13-11-4-9-7-8 | MTII-9 | CCII-A | CLII-1 | CMGII |
| 3176 | - | 2013 | Madagascar | AEZ7 | S51 | 1477 | -19,08 | 47,25 | DECEMBER | 21.9 | ST | 1 | - | - | - | - | - | **CSEQ** |
| 3177 | - | 2013 | Madagascar | AEZ7 | S51 | 1477 | -19,08 | 47,25 | DECEMBER | 21.9 | ST | - | - | - | - | - | - | **-** |
| 3178 | - | 2013 | Madagascar | AEZ7 | S51 | 1477 | -19,08 | 47,25 | DECEMBER | 21.9 | ST | 1 | - | - | - | - | - | **CSEQ** |
| 3179 | - | 2013 | Madagascar | AEZ7 | S51 | 1477 | -19,08 | 47,25 | DECEMBER | 21.9 | ST | - | - | - | - | - | - | **-** |
| 3181 | - | 2013 | Madagascar | AEZ7 | S51 | 1477 | -19,08 | 47,25 | DECEMBER | 21.9 | ST | 1 | - | - | - | - | - | **CSEQ** |
| 3182 | - | 2013 | Madagascar | AEZ7 | S51 | 1477 | -19,08 | 47,25 | DECEMBER | 21.9 | ST | - | - | - | - | - | - | **-** |
| 3183 | - | 2013 | Madagascar | AEZ7 | S51 | 1477 | -19,08 | 47,25 | DECEMBER | 21.9 | ST | 1 | - | - | - | - | - | **CSEQ** |
| 3184 | - | 2013 | Madagascar | AEZ7 | S51 | 1477 | -19,08 | 47,25 | DECEMBER | 21.9 | ST | - | - | - | - | - | - | **-** |
| 3185 | - | 2013 | Madagascar | AEZ7 | S51 | 1477 | -19,08 | 47,25 | DECEMBER | 21.9 | ST | - | - | - | - | - | - | **-** |
| 3186 | - | 2013 | Madagascar | AEZ7 | S51 | 1477 | -19,08 | 47,25 | DECEMBER | 21.9 | ST | 1 | - | - | - | - | - | **CSEQ** |
| 3187 | - | 2013 | Madagascar | AEZ7 | S51 | 1477 | -19,08 | 47,25 | DECEMBER | 21.9 | ST | 1 | RS2-MLVA9 | 4-5-6-13-11-4-9-7-8 | MTII-9 | CCII-A | CLII-1 | CSEQ, CMGII |
| 3188 | - | 2013 | Madagascar | AEZ7 | S51 | 1477 | -19,08 | 47,25 | DECEMBER | 21.9 | ST | - | - | - | - | - | - | **-** |
| 3189 | - | 2013 | Madagascar | AEZ7 | S51 | 1477 | -19,08 | 47,25 | DECEMBER | 21.9 | ST | 1 | - | - | - | - | - | **CSEQ** |
| 3190 | - | 2013 | Madagascar | AEZ7 | S51 | 1477 | -19,08 | 47,25 | DECEMBER | 21.9 | ST | - | - | - | - | - | - | **-** |
| 3191 | - | 2013 | Madagascar | AEZ7 | S51 | 1477 | -19,08 | 47,25 | DECEMBER | 21.9 | ST | - | - | - | - | - | - | **-** |
| 3193 | - | 2013 | Madagascar | AEZ7 | S50 | 1433 | -19,09 | 47,25 | DECEMBER | 21.9 | ST | - | - | - | - | - | - | **-** |
| 3194 | - | 2013 | Madagascar | AEZ7 | S50 | 1433 | -19,09 | 47,25 | DECEMBER | 21.9 | ST | 1 | - | - | - | - | - | **CSEQ** |
| 3195 | - | 2013 | Madagascar | AEZ7 | S50 | 1433 | -19,09 | 47,25 | DECEMBER | 21.9 | ST | - | - | - | - | - | - | **-** |
| 3196 | - | 2013 | Madagascar | AEZ7 | S50 | 1433 | -19,09 | 47,25 | DECEMBER | 21.9 | ST | - | - | - | - | - | - | **-** |
| 3197 | - | 2013 | Madagascar | AEZ7 | S50 | 1433 | -19,09 | 47,25 | DECEMBER | 21.9 | ST | - | - | - | - | - | - | **-** |
| 3198 | - | 2013 | Madagascar | AEZ7 | S50 | 1433 | -19,09 | 47,25 | DECEMBER | 21.9 | ST | - | - | - | - | - | - | **-** |
| 3199 | - | 2013 | Madagascar | AEZ7 | S50 | 1433 | -19,09 | 47,25 | DECEMBER | 21.9 | ST | - | RS2-MLVA9 | 4-5-6-13-11-4-9-7-8 | MTII-9 | CCII-A | CLII-1 | CMGII |
| 3200 | - | 2013 | Madagascar | AEZ7 | S50 | 1433 | -19,09 | 47,25 | DECEMBER | 21.9 | ST | - | - | - | - | - | - | **-** |
| 3201 | - | 2013 | Madagascar | AEZ7 | S50 | 1433 | -19,09 | 47,25 | DECEMBER | 21.9 | ST | - | - | - | - | - | - | **-** |
| 3202 | - | 2013 | Madagascar | AEZ7 | S50 | 1433 | -19,09 | 47,25 | DECEMBER | 21.9 | ST | - | - | - | - | - | - | **-** |
| 3203 | - | 2013 | Madagascar | AEZ7 | S50 | 1433 | -19,09 | 47,25 | DECEMBER | 21.9 | ST | - | - | - | - | - | - | **-** |
| 3204 | - | 2013 | Madagascar | AEZ7 | S50 | 1433 | -19,09 | 47,25 | DECEMBER | 21.9 | ST | - | - | - | - | - | - | **-** |
| 3205 | - | 2013 | Madagascar | AEZ7 | S50 | 1433 | -19,09 | 47,25 | DECEMBER | 21.9 | ST | - | - | - | - | - | - | **-** |
| 3206 | - | 2013 | Madagascar | AEZ7 | S50 | 1433 | -19,09 | 47,25 | DECEMBER | 21.9 | ST | - | - | - | - | - | - | **-** |
| 3207 | - | 2013 | Madagascar | AEZ7 | S50 | 1433 | -19,09 | 47,25 | DECEMBER | 21.9 | ST | 1 | RS2-MLVA9 | 4-5-6-13-11-4-9-7-8 | MTII-9 | CCII-A | CLII-1 | CSEQ, CMGII |
| 3209 | - | 2013 | Madagascar | AEZ7 | S50 | 1433 | -19,09 | 47,25 | DECEMBER | 21.9 | ST | - | - | - | - | - | - | **-** |
| 3225 | - | 2013 | Madagascar | AEZ7 | S50 | 1433 | -19,09 | 47,25 | DECEMBER | 21.9 | ST | - | - | - | - | - | - | **-** |
| 3229 | - | 2013 | Madagascar | AEZ8 | S70 | 1176 | -18,98 | 46,72 | DECEMBER | 21.3 | ST | - | RS2-MLVA9 | 4-5-6-13-11-4-9-7-8 | MTII-9 | CCII-A | CLII-1 | CMGII |
| 3232 | - | 2013 | Madagascar | AEZ8 | S70 | 1176 | -18,98 | 46,72 | DECEMBER | 21.3 | ST | 1 | RS2-MLVA9 | 4-5-6-13-11-4-9-7-8 | MTII-9 | CCII-A | CLII-1 | CSEQ, CMGII |
| 3233 | - | 2013 | Madagascar | AEZ8 | S70 | 1176 | -18,98 | 46,72 | DECEMBER | 21.3 | ST | - | RS2-MLVA9 | 4-5-6-13-11-4-9-7-8 | MTII-9 | CCII-A | CLII-1 | CMGII |
| 3234 | - | 2013 | Madagascar | AEZ8 | S70 | 1176 | -18,98 | 46,72 | DECEMBER | 21.3 | ST | - | RS2-MLVA9 | 4-5-6-13-11-4-9-7-8 | MTII-9 | CCII-A | CLII-1 | CMGII |
| 3238 | - | 2013 | Madagascar | AEZ8 | S70 | 1176 | -18,98 | 46,72 | DECEMBER | 21.3 | ST | - | RS2-MLVA9 | 4-5-6-3-11-4-9-7-8 | MTII-41 | CCII-A | CLII-1 | CMGII |
| 3240 | - | 2013 | Madagascar | AEZ8 | S70 | 1176 | -18,98 | 46,72 | DECEMBER | 21.3 | ST | - | RS2-MLVA9 | 4-5-6-13-11-4-9-7-8 | MTII-9 | CCII-A | CLII-1 | CMGII |
| 3248 | - | 2013 | Madagascar | AEZ9 | S74 | 1377 | -19,13 | 46,73 | DECEMBER | 19.4 | ST | - | - | - | - | - | - | **-** |
| 3249 | - | 2013 | Madagascar | AEZ9 | S74 | 1377 | -19,13 | 46,73 | DECEMBER | 19.4 | ST | - | - | - | - | - | - | **-** |
| 3250 | - | 2013 | Madagascar | AEZ9 | S74 | 1377 | -19,13 | 46,73 | DECEMBER | 19.4 | ST | - | - | - | - | - | - | **-** |
| 3251 | - | 2013 | Madagascar | AEZ9 | S74 | 1377 | -19,13 | 46,73 | DECEMBER | 19.4 | ST | - | - | - | - | - | - | **-** |
| 3252 | - | 2013 | Madagascar | AEZ9 | S74 | 1377 | -19,13 | 46,73 | DECEMBER | 19.4 | ST | - | - | - | - | - | - | **-** |
| 3253 | - | 2013 | Madagascar | AEZ9 | S74 | 1377 | -19,13 | 46,73 | DECEMBER | 19.4 | ST | - | - | - | - | - | - | **-** |
| 3254 | - | 2013 | Madagascar | AEZ9 | S74 | 1377 | -19,13 | 46,73 | DECEMBER | 19.4 | ST | - | - | - | - | - | - | **-** |
| 3255 | - | 2013 | Madagascar | AEZ9 | S74 | 1377 | -19,13 | 46,73 | DECEMBER | 19.4 | ST | - | - | - | - | - | - | **-** |
| 3258 | - | 2013 | Madagascar | AEZ9 | S74 | 1377 | -19,13 | 46,73 | DECEMBER | 19.4 | ST | - | - | - | - | - | - | **-** |
| 3259 | - | 2013 | Madagascar | AEZ9 | S74 | 1377 | -19,13 | 46,73 | DECEMBER | 19.4 | ST | - | - | - | - | - | - | **-** |
| 3260 | - | 2013 | Madagascar | AEZ9 | S74 | 1377 | -19,13 | 46,73 | DECEMBER | 19.4 | ST | - | - | - | - | - | - | **-** |
| 3261 | - | 2013 | Madagascar | AEZ9 | S74 | 1377 | -19,13 | 46,73 | DECEMBER | 19.4 | ST | - | - | - | - | - | - | **-** |
| 3262 | - | 2013 | Madagascar | AEZ9 | S74 | 1377 | -19,13 | 46,73 | DECEMBER | 19.4 | SL | - | - | - | - | - | - | **-** |
| 3263 | - | 2013 | Madagascar | AEZ9 | S74 | 1377 | -19,13 | 46,73 | DECEMBER | 19.4 | SL | - | - | - | - | - | - | **-** |
| 3293 | - | 2013 | Madagascar | AEZ10 | S22 | 1720 | -20,79 | 47,18 | DECEMBER | 20.3 | ST | - | - | - | - | - | - | **-** |
| 3294 | - | 2013 | Madagascar | AEZ10 | S22 | 1720 | -20,79 | 47,18 | DECEMBER | 20.3 | ST | - | - | - | - | - | - | **-** |
| 3295 | - | 2013 | Madagascar | AEZ10 | S22 | 1720 | -20,79 | 47,18 | DECEMBER | 20.3 | ST | - | - | - | - | - | - | **-** |
| 3297 | - | 2013 | Madagascar | AEZ10 | S22 | 1720 | -20,79 | 47,18 | DECEMBER | 20.3 | ST | - | - | - | - | - | - | **-** |
| 3298 | - | 2013 | Madagascar | AEZ10 | S22 | 1720 | -20,79 | 47,18 | DECEMBER | 20.3 | ST | - | - | - | - | - | - | **-** |
| 3299 | - | 2013 | Madagascar | AEZ10 | S22 | 1720 | -20,79 | 47,18 | DECEMBER | 20.3 | ST | - | - | - | - | - | - | **-** |
| 3300 | - | 2013 | Madagascar | AEZ10 | S22 | 1720 | -20,79 | 47,18 | DECEMBER | 20.3 | ST | - | - | - | - | - | - | **-** |
| 3301 | - | 2013 | Madagascar | AEZ10 | S22 | 1720 | -20,79 | 47,18 | DECEMBER | 20.3 | ST | - | - | - | - | - | - | **-** |
| 3305 | - | 2013 | Madagascar | AEZ10 | S21 | 1709 | -20,79 | 47,18 | DECEMBER | 20.3 | ST | - | - | - | - | - | - | **-** |
| 3306 | - | 2013 | Madagascar | AEZ10 | S21 | 1709 | -20,79 | 47,18 | DECEMBER | 20.3 | ST | - | - | - | - | - | - | **-** |
| 3307 | - | 2013 | Madagascar | AEZ10 | S21 | 1709 | -20,79 | 47,18 | DECEMBER | 20.3 | ST | - | - | - | - | - | - | **-** |
| 3308 | - | 2013 | Madagascar | AEZ10 | S21 | 1709 | -20,79 | 47,18 | DECEMBER | 20.3 | SL | - | - | - | - | - | - | **-** |
| 3309 | - | 2013 | Madagascar | AEZ10 | S23 | 1725 | -20,79 | 47,18 | DECEMBER | 20.3 | ST | - | - | - | - | - | - | **-** |
| 3310 | - | 2013 | Madagascar | AEZ10 | S23 | 1725 | -20,79 | 47,18 | DECEMBER | 20.3 | ST | - | - | - | - | - | - | **-** |
| 3313 | - | 2013 | Madagascar | AEZ10 | S23 | 1725 | -20,79 | 47,18 | DECEMBER | 20.3 | ST | - | - | - | - | - | - | **-** |
| 3315 | - | 2013 | Madagascar | AEZ10 | S23 | 1725 | -20,79 | 47,18 | DECEMBER | 20.3 | ST | - | - | - | - | - | - | **-** |
| 3318 | - | 2013 | Madagascar | AEZ10 | S23 | 1725 | -20,79 | 47,18 | DECEMBER | 20.3 | ST | - | - | - | - | - | - | **-** |
| 3319 | - | 2013 | Madagascar | AEZ10 | S23 | 1725 | -20,79 | 47,18 | DECEMBER | 20.3 | ST | - | - | - | - | - | - | **-** |
| 3320 | - | 2013 | Madagascar | AEZ10 | S23 | 1725 | -20,79 | 47,18 | DECEMBER | 20.3 | ST | - | - | - | - | - | - | **-** |
| 3322 | - | 2013 | Madagascar | AEZ10 | S23 | 1725 | -20,79 | 47,18 | DECEMBER | 20.3 | ST | - | - | - | - | - | - | **-** |
| 3323 | - | 2013 | Madagascar | AEZ10 | S23 | 1725 | -20,79 | 47,18 | DECEMBER | 20.3 | ST | - | - | - | - | - | - | **-** |
| 3332 | - | 2013 | Madagascar | AEZ10 | S18 | 1637 | -20,82 | 47,18 | DECEMBER | 20.3 | ST | - | - | - | - | - | - | **-** |
| 3333 | - | 2013 | Madagascar | AEZ10 | S19 | 1642 | -20,82 | 47,18 | DECEMBER | 20.3 | ST | - | - | - | - | - | - | **-** |
| 3350 | - | 2013 | Madagascar | AEZ10 | S15 | 1639 | -20,82 | 47,18 | DECEMBER | 20.3 | ST | - | RS2-MLVA9 | 4-5-6-13-11-4-9-7-8 | MTII-9 | CCII-A | CLII-1 | CMGII |
| 3351 | - | 2013 | Madagascar | AEZ10 | S15 | 1639 | -20,82 | 47,18 | DECEMBER | 20.3 | ST | - | RS2-MLVA9 | 4-5-6-13-11-4-9-7-8 | MTII-9 | CCII-A | CLII-1 | CMGII |
| 3352 | - | 2013 | Madagascar | AEZ10 | S15 | 1639 | -20,82 | 47,18 | DECEMBER | 20.3 | ST | - | RS2-MLVA9 | 4-5-6-13-11-4-9-7-8 | MTII-9 | CCII-A | CLII-1 | CMGII |
| 3353 | - | 2013 | Madagascar | AEZ10 | S15 | 1639 | -20,82 | 47,18 | DECEMBER | 20.3 | ST | - | RS2-MLVA9 | 4-5-6-2-11-4-9-7-8 | MTII-40 | CCII-A | CLII-1 | CMGII |
| 3354 | - | 2013 | Madagascar | AEZ10 | S15 | 1639 | -20,82 | 47,18 | DECEMBER | 20.3 | ST | 1 | RS2-MLVA9 | 4-5-6-13-11-4-9-7-8 | MTII-9 | CCII-A | CLII-1 | CSEQ, CMGII |
| 3357 | - | 2013 | Madagascar | AEZ10 | S13 | 1579 | -20,83 | 47,19 | DECEMBER | 20.3 | ST | - | - | - | - | - | - | **-** |
| 3359 | - | 2013 | Madagascar | AEZ10 | S13 | 1579 | -20,83 | 47,19 | DECEMBER | 20.3 | ST | - | - | - | - | - | - | **-** |
| 3369 | - | 2013 | Madagascar | AEZ11 | S10 | 1442 | -20,97 | 47,14 | DECEMBER | 19.9 | ST | - | - | - | - | - | - | **-** |
| 3370 | - | 2013 | Madagascar | AEZ11 | S10 | 1442 | -20,97 | 47,14 | DECEMBER | 19.9 | ST | - | - | - | - | - | - | **-** |
| 3371 | - | 2013 | Madagascar | AEZ11 | S10 | 1442 | -20,97 | 47,14 | DECEMBER | 19.9 | ST | - | - | - | - | - | - | **-** |
| 3372 | - | 2013 | Madagascar | AEZ11 | S10 | 1442 | -20,97 | 47,14 | DECEMBER | 19.9 | ST | - | - | - | - | - | - | **-** |
| 3373 | - | 2013 | Madagascar | AEZ11 | S10 | 1442 | -20,97 | 47,14 | DECEMBER | 19.9 | ST | - | - | - | - | - | - | **-** |
| 3374 | - | 2013 | Madagascar | AEZ11 | S10 | 1442 | -20,97 | 47,14 | DECEMBER | 19.9 | ST | - | - | - | - | - | - | **-** |
| 3376 | - | 2013 | Madagascar | AEZ11 | S10 | 1442 | -20,97 | 47,14 | DECEMBER | 19.9 | ST | - | - | - | - | - | - | **-** |
| 3378 | - | 2013 | Madagascar | AEZ11 | S10 | 1442 | -20,97 | 47,14 | DECEMBER | 19.9 | ST | - | - | - | - | - | - | **-** |
| 3379 | - | 2013 | Madagascar | AEZ11 | S10 | 1442 | -20,97 | 47,14 | DECEMBER | 19.9 | ST | - | - | - | - | - | - | **-** |
| 3382 | - | 2013 | Madagascar | AEZ11 | S11 | 1338 | -20,88 | 47,17 | DECEMBER | 19.9 | SL | - | - | - | - | - | - | **-** |
| 3386 | - | 2013 | Madagascar | AEZ11 | S11 | 1338 | -20,88 | 47,17 | DECEMBER | 19.9 | BP | - | - | - | - | - | - | **-** |
| 3388 | - | 2013 | Madagascar | AEZ10 | S15 | 1639 | -20,82 | 47,18 | DECEMBER | 20.3 | ST | - | - | - | - | - | - | **-** |
| 3393 | - | 2013 | Madagascar | AEZ11 | S12 | 1351 | -20,88 | 47,17 | DECEMBER | 19.9 | SL | - | - | - | - | - | - | **-** |
| 3394 | - | 2013 | Madagascar | AEZ11 | S12 | 1351 | -20,88 | 47,17 | DECEMBER | 19.9 | SL | - | - | - | - | - | - | **-** |
| 3396 | - | 2013 | Madagascar | AEZ11 | S12 | 1351 | -20,88 | 47,17 | DECEMBER | 19.9 | SL | - | - | - | - | - | - | **-** |
| 3398 | - | 2013 | Madagascar | AEZ11 | S12 | 1351 | -20,88 | 47,17 | DECEMBER | 19.9 | SL | - | - | - | - | - | - | **-** |
| 3400 | - | 2013 | Madagascar | AEZ11 | S12 | 1351 | -20,88 | 47,17 | DECEMBER | 19.9 | SL | - | - | - | - | - | - | **-** |
| 3406 | - | 2013 | Madagascar | AEZ11 | S12 | 1351 | -20,88 | 47,17 | DECEMBER | 19.9 | SL | - | - | - | - | - | - | **-** |
| 3408 | - | 2013 | Madagascar | AEZ11 | S12 | 1351 | -20,88 | 47,17 | DECEMBER | 19.9 | SL | - | - | - | - | - | - | **-** |
| 3410 | - | 2013 | Madagascar | AEZ11 | S12 | 1351 | -20,88 | 47,17 | DECEMBER | 19.9 | ST | - | - | - | - | - | - | **-** |
| 3413 | - | 2013 | Madagascar | AEZ7 | S47 | 1379 | -19,08 | 47,17 | DECEMBER | 21.9 | ST | - | - | - | - | - | - | **-** |
| 3417 | - | 2013 | Madagascar | AEZ7 | S47 | 1379 | -19,08 | 47,17 | DECEMBER | 21.9 | PV | - | RS2-MLVA9 | 4-5-6-11-11-4-9-7-10 | MTII-23 | CCII-A | CLII-1 | CMGII |
| 3418 | - | 2013 | Madagascar | AEZ7 | S47 | 1379 | -19,08 | 47,17 | DECEMBER | 21.9 | ST | - | - | - | - | - | - | **-** |
| 3419 | - | 2013 | Madagascar | AEZ7 | S47 | 1379 | -19,08 | 47,17 | DECEMBER | 21.9 | ST | - | - | - | - | - | - | **-** |
| 3420 | - | 2013 | Madagascar | AEZ7 | S47 | 1379 | -19,08 | 47,17 | DECEMBER | 21.9 | ST | - | RS2-MLVA9 | 4-5-6-14-11-4-9-7-8 | MTII-11 | CCII-A | CLII-1 | CMGII |
| 3422 | - | 2013 | Madagascar | AEZ7 | S47 | 1379 | -19,08 | 47,17 | DECEMBER | 21.9 | ST | - | - | - | - | - | - | **-** |
| 3423 | - | 2013 | Madagascar | AEZ7 | S47 | 1379 | -19,08 | 47,17 | DECEMBER | 21.9 | ST | - | - | - | - | - | - | **-** |
| 3426 | - | 2013 | Madagascar | AEZ7 | S47 | 1379 | -19,08 | 47,17 | DECEMBER | 21.9 | ST | - | - | - | - | - | - | **-** |
| 3427 | - | 2013 | Madagascar | AEZ7 | S47 | 1379 | -19,08 | 47,17 | DECEMBER | 21.9 | ST | 1 | RS2-MLVA9 | 4-5-6-13-11-4-9-7-8 | MTII-9 | CCII-A | CLII-1 | CSEQ, CMGII |
| 3428 | - | 2013 | Madagascar | AEZ7 | S47 | 1379 | -19,08 | 47,17 | DECEMBER | 21.9 | ST | - | - | - | - | - | - | **-** |
| 3429 | - | 2013 | Madagascar | AEZ7 | S45 | 1390 | -19,08 | 47,17 | DECEMBER | 21.9 | PV | - | RS2-MLVA9 | 4-5-6-13-11-4-9-7-8 | MTII-9 | CCII-A | CLII-1 | CMGII |
| 3431 | - | 2013 | Madagascar | AEZ7 | S47 | 1379 | -19,08 | 47,17 | DECEMBER | 21.9 | ST | - | - | - | - | - | - | **-** |
| 3432 | - | 2013 | Madagascar | AEZ7 | S47 | 1379 | -19,08 | 47,17 | DECEMBER | 21.9 | ST | - | - | - | - | - | - | **-** |
| 3433 | - | 2013 | Madagascar | AEZ7 | S47 | 1379 | -19,08 | 47,17 | DECEMBER | 21.9 | ST | - | - | - | - | - | - | **-** |
| 3434 | - | 2013 | Madagascar | AEZ7 | S47 | 1379 | -19,08 | 47,17 | DECEMBER | 21.9 | ST | - | - | - | - | - | - | **-** |
| 3435 | - | 2013 | Madagascar | AEZ7 | S47 | 1379 | -19,08 | 47,17 | DECEMBER | 21.9 | ST | - | - | - | - | - | - | **-** |
| 3437 | - | 2013 | Madagascar | AEZ7 | S47 | 1379 | -19,08 | 47,17 | DECEMBER | 21.9 | ST | - | - | - | - | - | - | **-** |
| 3439 | - | 2013 | Madagascar | AEZ7 | S47 | 1379 | -19,08 | 47,17 | DECEMBER | 21.9 | ST | - | - | - | - | - | - | **-** |
| 3440 | - | 2013 | Madagascar | AEZ7 | S47 | 1379 | -19,08 | 47,17 | DECEMBER | 21.9 | ST | - | - | - | - | - | - | **-** |
| 3443 | - | 2013 | Madagascar | AEZ7 | S47 | 1379 | -19,08 | 47,17 | DECEMBER | 21.9 | ST | - | - | - | - | - | - | **-** |
| 3445 | - | 2013 | Madagascar | AEZ7 | S47 | 1379 | -19,08 | 47,17 | DECEMBER | 21.9 | ST | - | - | - | - | - | - | **-** |
| 3446 | - | 2013 | Madagascar | AEZ7 | S48 | 1575 | -19,17 | 47,17 | DECEMBER | 21.9 | ST | - | - | - | - | - | - | **-** |
| 3447 | - | 2013 | Madagascar | AEZ7 | S46 | 1456 | -19,11 | 47,17 | DECEMBER | 21.9 | ST | - | RS2-MLVA9 | 4-5-6-13-11-4-9-7-12 | MTII-6 | CCII-A | CLII-1 | CMGII |
| 3448 | - | 2013 | Madagascar | AEZ7 | S46 | 1456 | -19,11 | 47,17 | DECEMBER | 21.9 | ST | - | - | - | - | - | - | **-** |
| 3449 | - | 2013 | Madagascar | AEZ7 | S46 | 1456 | -19,11 | 47,17 | DECEMBER | 21.9 | ST | - | - | - | - | - | - | **-** |
| 3450 | - | 2013 | Madagascar | AEZ7 | S46 | 1456 | -19,11 | 47,17 | DECEMBER | 21.9 | ST | - | - | - | - | - | - | **-** |
| 3451 | - | 2013 | Madagascar | AEZ7 | S46 | 1456 | -19,11 | 47,17 | DECEMBER | 21.9 | ST | - | - | - | - | - | - | **-** |
| 3452 | - | 2013 | Madagascar | AEZ7 | S46 | 1456 | -19,11 | 47,17 | DECEMBER | 21.9 | ST | - | - | - | - | - | - | **-** |
| 3453 | - | 2013 | Madagascar | AEZ7 | S46 | 1456 | -19,11 | 47,17 | DECEMBER | 21.9 | ST | 1 | RS2-MLVA9 | 4-5-6-13-11-4-9-7-12 | MTII-6 | CCII-A | CLII-1 | CSEQ, CMGII |
| 3454 | - | 2013 | Madagascar | AEZ7 | S46 | 1456 | -19,11 | 47,17 | DECEMBER | 21.9 | ST | - | - | - | - | - | - | **-** |
| 3455 | - | 2013 | Madagascar | AEZ7 | S46 | 1456 | -19,11 | 47,17 | DECEMBER | 21.9 | ST | - | - | - | - | - | - | **-** |
| 3456 | - | 2013 | Madagascar | AEZ7 | S46 | 1456 | -19,11 | 47,17 | DECEMBER | 21.9 | ST | - | - | - | - | - | - | **-** |
| 3473 | - | 2013 | Madagascar | AEZ9 | S74 | 1377 | -19,13 | 46,73 | DECEMBER | 19.4 | ST | - | - | - | - | - | - | **-** |
| 3474 | - | 2013 | Madagascar | AEZ9 | S74 | 1377 | -19,13 | 46,73 | DECEMBER | 19.4 | ST | - | - | - | - | - | - | **-** |
| 3475 | - | 2013 | Madagascar | AEZ9 | S74 | 1377 | -19,13 | 46,73 | DECEMBER | 19.4 | ST | - | - | - | - | - | - | **-** |
| 3476 | - | 2013 | Madagascar | AEZ9 | S74 | 1377 | -19,13 | 46,73 | DECEMBER | 19.4 | ST | - | - | - | - | - | - | **-** |
| 3477 | - | 2013 | Madagascar | AEZ9 | S74 | 1377 | -19,13 | 46,73 | DECEMBER | 19.4 | ST | - | - | - | - | - | - | **-** |
| 3478 | - | 2013 | Madagascar | AEZ9 | S74 | 1377 | -19,13 | 46,73 | DECEMBER | 19.4 | ST | - | - | - | - | - | - | **-** |
| 3479 | - | 2013 | Madagascar | AEZ9 | S74 | 1377 | -19,13 | 46,73 | DECEMBER | 19.4 | ST | - | - | - | - | - | - | **-** |
| 3481 | - | 2013 | Madagascar | AEZ10 | S22 | 1720 | -20,79 | 47,18 | DECEMBER | 20.3 | ST | - | - | - | - | - | - | **-** |
| 3482 | - | 2013 | Madagascar | AEZ10 | S22 | 1720 | -20,79 | 47,18 | DECEMBER | 20.3 | ST | - | - | - | - | - | - | **-** |
| 3483 | - | 2013 | Madagascar | AEZ10 | S22 | 1720 | -20,79 | 47,18 | DECEMBER | 20.3 | ST | - | - | - | - | - | - | **-** |
| 3485 | - | 2013 | Madagascar | AEZ10 | S22 | 1720 | -20,79 | 47,18 | DECEMBER | 20.3 | ST | - | - | - | - | - | - | **-** |
| 3486 | - | 2013 | Madagascar | AEZ10 | S22 | 1720 | -20,79 | 47,18 | DECEMBER | 20.3 | ST | - | - | - | - | - | - | **-** |
| 3487 | - | 2013 | Madagascar | AEZ10 | S22 | 1720 | -20,79 | 47,18 | DECEMBER | 20.3 | ST | - | - | - | - | - | - | **-** |
| 3488 | - | 2013 | Madagascar | AEZ10 | S22 | 1720 | -20,79 | 47,18 | DECEMBER | 20.3 | ST | - | - | - | - | - | - | **-** |
| 3502 | - | 2013 | Madagascar | AEZ7 | S49 | 1476 | -19,07 | 47,20 | DECEMBER | 21.9 | ST | - | - | - | - | - | - | **-** |
| 3504 | - | 2013 | Madagascar | AEZ7 | S46 | 1456 | -19,11 | 47,17 | DECEMBER | 21.9 | ST | - | - | - | - | - | - | **-** |
| 3511 | - | 2013 | Madagascar | AEZ7 | S47 | 1379 | -19,08 | 47,17 | DECEMBER | 21.9 | ST | - | - | - | - | - | - | **-** |
| 3512 | - | 2013 | Madagascar | AEZ11 | S10 | 1442 | -20,97 | 47,14 | DECEMBER | 19.9 | ST | 1 | RS2-MLVA9 | 4-5-6-13-11-4-9-7-8 | MTII-9 | CCII-A | CLII-1 | CSEQ, CMGII |
| 3516 | - | 2013 | Madagascar | AEZ10 | S13 | 1579 | -20,83 | 47,19 | DECEMBER | 20.3 | ST | 1 | RS2-MLVA9 | 4-5-6-13-11-4-9-7-8 | MTII-9 | CCII-A | CLII-1 | CSEQ, CMGII |
| 3517 | - | 2013 | Madagascar | AEZ10 | S13 | 1579 | -20,83 | 47,19 | DECEMBER | 20.3 | ST | - | RS2-MLVA9 | 4-5-6-13-11-4-9-7-8 | MTII-9 | CCII-A | CLII-1 | CMGII |
|  |  |  |  |  |  |  |  |  |  |  |  |  |  |  |  |  |  |  |
| **PHYLOTYPE III** | |  |  |  |  |  |  |  |  |  |  |  |  |  |  |  |  |  |
| 39 | CFBP3059 | 1990 | Burkina Faso | - | - | - | - | - |  | - | SM | 23 | RS3-MLVA16 | 5-3-2-3-3-3-2-2-8-4-2-5-3-4-13-2 | MTIII-23 | - | - | CREF-III |
| 46 | CIP358 | 1989 | Cameroon | - | - | - | - | - |  | - | ST | 29 | RS3-MLVA16 | 7-3-2-9-3-3-2-2-16-9-3-5-4-4-4-12 | MTIII-43 | - | - | CREF-III |
| 53 | CFBP7022 | 2005 | Cameroon | - | - | - | - | - |  | - | SS | 29 | RS3-MLVA16 | 14-3-2-13-8-3-2-2-12-10-3-5-3-4-11-15 | MTIII-15 | - | - | CREF-III |
| 56 | J25 | 1998 | Kenya | - | - | - | - | - |  | - | ST | 20 | RS3-MLVA16 | 38-3-2-14-6-3-2-2-15-9-3-5-7-4-13-16 | MTIII-22 | - | - | CREF-III |
| 60 | JT525 | 1993 | Kenya | - | - | - | - | - |  | - | P | 19 | RS3-MLVA16 | 11-3-3-10-7-26-2-2-19-9-3-5-10-3-5-9 | MTIII-7 | - | - | CREF-III |
| 61 | JT528 | 1993 | Kenya | - | - | - | - | - |  | - | ST | 19 | RS3-MLVA16 | 11-3-3-9-7-26-2-2-19-9-3-5-10-3-5-9 | MTIII-9 | - | - | CREF-III |
| 75 | NCPPB0332 | 1954 | Zimbabwe | - | - | - | - | - |  | - | ST | 22 | RS3-MLVA16 | 14-3-2-10-7-19-2-2-20-9-3-5-3-4-14-9 | MTIII-14 | - | - | CREF-III |
| 76 | NCPPB0342 | - | Zimbabwe | - | - | - | - | - |  | - | NT | 20 | RS3-MLVA16 | 3-3-2-7-7-3-2-2-12-9-3-9-7-4-11-12 | MTIII-20 | - | - | CREF-III |
| 98 | K179 | 1959 | Zimbabwe | - | - | - | - | - |  | - | NT | 21 | RS3-MLVA16 | 5-3-3-12-9-26-2-2-9-6-3-5-5-4-4-13 | MTIII-24 | - | - | CREF-III |
| 133 | CMR15 | 2005 | Cameroon | - | - | - | - | - |  | - | SL | 29 | RS3-MLVA16 | 10-4-3-9-7-12-6-3-12-9-3-5-6-4-15-12 | MTIII-3 | - | - | CREF-III |
| 137 | CMR20 | 2005 | Cameroon | - | - | - | - | - |  | - | SL | 29 | RS3-MLVA16 | 10-4-3-9-7-12-6-3-12-10-3-5-6-4-16-16 | MTIII-4 | - | - | CREF-III |
| 143 | CFBP7028 | 2005 | Cameroon | - | - | - | - | - |  | - | SL | 29 | RS3-MLVA16 | 14-3-2-13-8-3-2-3-19-10-3-5-3-4-11-10 | MTIII-18 | - | - | CREF-III |
| 145 | CMR32 | 2005 | Cameroon | - | - | - | - | - |  | - | SS | 29 | RS3-MLVA16 | 6-3-2-8-7-3-2-2-15-9-3-5-4-4-4-11 | MTIII-29 | - | - | CREF-III |
| 146 | CMR33 | 2005 | Cameroon | - | - | - | - | - |  | - | SL | 20 | RS3-MLVA16 | 9-4-2-0-6-3-2-2-16-9-3-5-6-4-5-15 | MTIII-45 | - | - | CREF-III |
| 149 | CFBP7031 | 2005 | Cameroon | - | - | - | - | - |  | - | SL | 29 | RS3-MLVA16 | 14-3-2-13-8-3-2-2-12-10-3-5-3-4-12-10 | MTIII-16 | - | - | CREF-III |
| 151 | CFBP7033 | 2005 | Cameroon | - | - | - | - | - |  | - | ST | 29 | RS3-MLVA16 | 6-3-2-8-7-8-2-2-12-9-3-5-3-4-4-12 | MTIII-30 | - | - | CREF-III |
| 164 | CFBP7036 | 2005 | Cameroon | - | - | - | - | - |  | - | SL | 29 | RS3-MLVA16 | 14-3-2-13-8-8-2-2-12-10-3-5-3-4-11-14 | MTIII-19 | - | - | CREF-III |
| 165 | CFBP7037 | 2005 | Cameroon | - | - | - | - | - |  | - | SL | 29 | RS3-MLVA16 | 14-3-2-13-8-3-2-2-19-10-3-2-3-4-11-13 | MTIII-17 | - | - | CREF-III |
| 166 | CMR66 | 2005 | Cameroon | - | - | - | - | - |  | - | SS | 49 | RS3-MLVA16 | 9-4-2-12-4-12-2-2-22-10-3-5-3-4-9-16 | MTIII-46 | - | - | CREF-III |
| 171 | CMR75 | 2005 | Cameroon | - | - | - | - | - |  | - | SL | 29 | RS3-MLVA16 | 6-3-2-15-7-8-2-2-15-8-3-5-4-4-5-12 | MTIII-26 | - | - | CREF-III |
| 172 | CFBP7041 | 2005 | Cameroon | - | - | - | - | - |  | - | SL | 29 | RS3-MLVA16 | 6-3-2-15-7-8-2-2-15-8-3-5-5-4-5-12 | MTIII-27 | - | - | CREF-III |
| 173 | CMR77 | 2005 | Cameroon | - | - | - | - | - |  | - | SL | - | RS3-MLVA16 | 6-3-2-15-7-8-2-3-12-8-3-5-3-4-5-12 | MTIII-28 | - | - | CREF-III |
| 174 | CMR78 | 2005 | Cameroon | - | - | - | - | - |  | - | CA | 29 | RS3-MLVA16 | 6-3-2-15-7-3-2-2-13-8-3-5-4-4-5-12 | MTIII-25 | - | - | CREF-III |
| 175 | CMR79 | 2005 | Cameroon | - | - | - | - | - |  | - | SL | 29 | RS3-MLVA16 | 7-3-2-15-9-8-2-2-12-10-3-4-6-4-5-18 | MTIII-34 | - | - | CREF-III |
| 232 | CFBP7060 | 2005 | Cameroon | - | - | - | - | - |  | - | SL | 29 | RS3-MLVA16 | 7-3-2-11-7-8-2-2-15-9-3-5-3-4-6-10 | MTIII-37 | - | - | CREF-III |
| 233 | CFBP7061 | 2005 | Cameroon | - | - | - | - | - |  | - | SL | 29 | RS3-MLVA16 | 7-3-2-11-7-3-2-2-12-9-3-5-3-4-6-10 | MTIII-36 | - | - | CREF-III |
| 234 | CMR52 | 2005 | Cameroon | - | - | - | - | - |  | - | ST | 29 | RS3-MLVA16 | 6-3-2-9-7-3-2-2-18-9-3-5-4-4-4-12 | MTIII-32 | - | - | CREF-III |
| 235 | CMR53 | 2005 | Cameroon | - | - | - | - | - |  | - | ST | 29 | RS3-MLVA16 | 6-3-2-9-7-3-2-2-12-9-3-5-3-4-4-12 | MTIII-31 | - | - | CREF-III |
| 332 | MAD029 | 2006 | Madagascar | - | - | - | - | - |  | - | ST | 19 | RS3-MLVA16 | 9-3-2-8-15-3-2-3-16-9-3-7-5-3-4-15 | MTIII-44 | - | - | CREF-III |
| 361 | DGBBC1139 | - | Guinea | - | - | - | - | - |  | - | ST | 42 | RS3-MLVA16 | 7-3-2-18-9-12-2-3-12-9-3-6-5-9-13-15 | MTIII-39 | - | - | CREF-III |
| 362 | DGBBC1138 | - | Guinea | - | - | - | - | - |  | - | ST | 44 | RS3-MLVA16 | 12-2-3-20-6-12-2-2-6-9-3-5-7-9-11-16 | MTIII-10 | - | - | CREF-III |
| 364 | DGBBC1227 | - | Guinea | - | - | - | - | - |  | - | ST | 42 | RS3-MLVA16 | 10-2-2-11-5-12-2-2-13-7-3-5-11-5-37-22 | MTIII-2 | - | - | CREF-III |
| 365 | DGBBC1136 | - | Guinea | - | - | - | - | - |  | - | ST | 42 | RS3-MLVA16 | 7-3-2-20-7-12-2-3-12-9-3-6-5-9-13-16 | MTIII-42 | - | - | CREF-III |
| 366 | DGBBC1222 | - | Guinea | - | - | - | - | - |  | - | ST | 42 | RS3-MLVA16 | 7-3-2-19-7-40-2-3-12-9-3-6-5-9-13-16 | MTIII-41 | - | - | CREF-III |
| 367 | DGBBC1223 | - | Guinea | - | - | - | - | - |  | - | ST | 42 | RS3-MLVA16 | 10-2-2-11-5-12-2-2-13-7-3-5-11-5-37-22 | MTIII-2 | - | - | CREF-III |
| 368 | DGBBC1259 | - | Guinea | - | - | - | - | - |  | - | ST | 42 | RS3-MLVA16 | 7-1-2-9-10-13-2-3-35-10-3-5-6-5-9-12 | MTIII-33 | - | - | CREF-III |
| 369 | DGBBC1125 | - | Guinea | - | - | - | - | - |  | - | ST | 43 | RS3-MLVA16 | 12-2-3-15-6-34-2-3-27-8-3-5-8-8-31-15 | MTIII-11 | - | - | CREF-III |
| 477 | CFBP734 | 1997 | Madagascar | - | - | - | - | - |  | - | ST | 19 | RS3-MLVA16 | 7-3-2-19-4-30-2-2-26-9-3-5-3-3-4-13 | MTIII-40 | - | - | CREF-III |
| 478 | NCPPB0283 | 1950 | Zimbabwe | - | - | - | - | - |  | - | SP | 22 | RS3-MLVA16 | 7-3-2-10-4-3-2-2-16-9-3-5-6-3-11-13 | MTIII-35 | - | - | CREF-III |
| 479 | NCPPB1018 | 1961 | Angola | - | - | - | - | - |  | - | ST | 21 | RS3-MLVA16 | 3-3-3-12-3-25-2-2-16-7-3-5-3-4-4-17 | MTIII-21 | - | - | CREF-III |
| 480 | NCPPB0505 | 1957 | Angola | - | - | - | - | - |  | - | SO | 20 | RS3-MLVA16 | 7-3-2-12-6-3-2-3-15-9-3-5-6-4-7-16 | MTIII-38 | - | - | CREF-III |
| 657 | CFBP2146 | 1980 | Reunion | - | - | - | - | - |  | - | P | 19 | RS3-MLVA16 | 10-3-3-9-7-26-2-2-19-9-3-5-10-3-5-9 | MTIII-48 | - | - | CREF-III |
| 842 | CFBP4809 | 1992 | Reunion | - | - | - | - | - |  | - | P | 19 | RS3-MLVA16 | 11-3-3-8-7-27-2-2-20-9-3-5-10-3-5-9 | MTIII-8 | - | - | CREF-III |
| 1238 | CFBP4963 | 1999 | Reunion | - | - | - | - | - |  | - | ST | 19 | RS3-MLVA16 | 12-3-3-9-7-27-2-2-19-9-3-5-10-3-5-9 | MTIII-12 | - | - | CREF-III |
| 1298 | NCPPB0332 | - | Zimbabwe | - | - | - | - | - |  | - | ST | 22 | RS3-MLVA16 | 14-3-2-10-3-19-2-2-12-9-3-5-3-4-14-17 | MTIII-13 | - | - | CREF-III |
| 1793 | CIV56 | 2010 | Ivory Coast | - | - | - | - | - |  | - | SM | 48 | RS3-MLVA16 | 11-3-2-3-5-4-2-2-12-6-2-5-2-5-35-2 | MTIII-1 | - | - | CREF-III |
| 1794 | CIV57 | 2010 | Ivory Coast | - | - | - | - | - |  | - | SM | 48 | RS3-MLVA16 | 11-3-2-3-5-4-2-2-12-6-2-5-2-5-35-2 | MTIII-1 | - | - | CREF-III |
| 1796 | CIV59 | 2010 | Ivory Coast | - | - | - | - | - |  | - | SM | 48 | RS3-MLVA16 | 11-3-2-3-5-4-2-2-12-6-2-5-2-5-35-2 | MTIII-1 | - | - | CREF-III |
| 2276 | - | 2013 | Madagascar | AEZ1 | S36 | 1665 | -19,84 | 47,29 | AVRIL | 17.7 | ST | 19 | RS3-MLVA16 | 7-3-2-12-5-7-2-2-21-7-3-5-6-3-5-13 | MTIII-129 | CC MTIII-2 | CLIII-9 | CMGIII |
| 2278 | - | 2013 | Madagascar | AEZ1 | S36 | 1665 | -19,84 | 47,29 | AVRIL | 17.7 | BP | - | RS3-MLVA16 | 7-3-2-10-4-6-2-2-26-9-3-5-6-3-3-12 | MTIII-64 | CC MTIII-11 | CLIII-9 | CMGIII |
| 2279 | - | 2013 | Madagascar | AEZ1 | S36 | 1665 | -19,84 | 47,29 | AVRIL | 17.7 | ST | 60 | RS3-MLVA16 | 7-3-2-10-4-6-2-2-26-9-3-5-6-3-3-12 | MTIII-64 | CC MTIII-11 | CLIII-9 | CMGIII |
| 2280 | - | 2013 | Madagascar | AEZ1 | S36 | 1665 | -19,84 | 47,29 | AVRIL | 17.7 | ST | 59 | RS3-MLVA16 | 8-3-2-8-11-9-2-2-12-9-3-7-6-4-5-12 | MTIII-87 | - | CLIII-9 | CMGIII |
| 2285 | - | 2013 | Madagascar | AEZ1 | S35 | 1657 | -19,93 | 47,27 | AVRIL | 17.7 | ST | - | RS3-MLVA16 | 7-3-2-8-4-6-2-2-23-9-3-5-6-3-3-13 | MTIII-51 | CC MTIII-1 | CLIII-2 | CMGIII |
| 2288 | - | 2013 | Madagascar | AEZ1 | S35 | 1657 | -19,93 | 47,27 | AVRIL | 17.7 | ST | - | RS3-MLVA16 | 7-3-2-8-4-6-2-2-23-9-3-5-6-3-3-13 | MTIII-51 | CC MTIII-1 | CLIII-2 | CMGIII |
| 2289 | - | 2013 | Madagascar | AEZ1 | S35 | 1657 | -19,93 | 47,27 | AVRIL | 17.7 | ST | - | - | - | - | - | - | **-** |
| 2290 | - | 2013 | Madagascar | AEZ1 | S35 | 1657 | -19,93 | 47,27 | AVRIL | 17.7 | ST | - | RS3-MLVA16 | 7-3-2-12-5-7-2-2-19-7-3-5-6-3-5-13 | MTIII-59 | CC MTIII-2 | CLIII-2 | CMGIII |
| 2291 | - | 2013 | Madagascar | AEZ1 | S35 | 1657 | -19,93 | 47,27 | AVRIL | 17.7 | ST | 19 | RS3-MLVA16 | 7-3-2-12-5-7-2-2-19-7-3-5-5-3-5-13 | MTIII-133 | CC MTIII-2 | CLIII-2 | CMGIII |
| 2292 | - | 2013 | Madagascar | AEZ1 | S35 | 1657 | -19,93 | 47,27 | AVRIL | 17.7 | ST | - | RS3-MLVA16 | 7-3-2-12-5-7-2-2-19-7-3-5-6-3-5-13 | MTIII-59 | CC MTIII-2 | CLIII-2 | CMGIII |
| 2293 | - | 2013 | Madagascar | AEZ1 | S35 | 1657 | -19,93 | 47,27 | AVRIL | 17.7 | ST | 19 | RS3-MLVA16 | 7-3-2-12-5-8-2-2-19-7-3-5-6-3-5-13 | MTIII-127 | CC MTIII-2 | CLIII-2 | CMGIII |
| 2294 | - | 2013 | Madagascar | AEZ1 | S35 | 1657 | -19,93 | 47,27 | AVRIL | 17.7 | ST | - | RS3-MLVA16 | 7-3-2-12-5-7-2-2-19-7-3-5-6-3-5-13 | MTIII-59 | CC MTIII-2 | CLIII-2 | CMGIII |
| 2296 | - | 2013 | Madagascar | AEZ1 | S35 | 1657 | -19,93 | 47,27 | AVRIL | 17.7 | ST | 60 | RS3-MLVA16 | 7-3-2-8-4-6-2-2-24-9-3-5-6-3-3-13 | MTIII-49 | CC MTIII-1 | CLIII-2 | CMGIII |
| 2298 | - | 2013 | Madagascar | AEZ1 | S35 | 1657 | -19,93 | 47,27 | AVRIL | 17.7 | ST | - | RS3-MLVA16 | 7-3-2-8-4-6-2-2-24-9-3-5-6-3-3-13 | MTIII-49 | CC MTIII-1 | CLIII-2 | CMGIII |
| 2301 | - | 2013 | Madagascar | AEZ1 | S35 | 1657 | -19,93 | 47,27 | AVRIL | 17.7 | ST | - | RS3-MLVA16 | 7-3-2-8-4-6-2-2-23-9-3-5-6-3-3-13 | MTIII-51 | CC MTIII-1 | CLIII-2 | CMGIII |
| 2304 | - | 2013 | Madagascar | AEZ1 | S35 | 1657 | -19,93 | 47,27 | AVRIL | 17.7 | ST | 60 | RS3-MLVA16 | 7-3-2-8-4-6-2-2-23-9-3-5-6-3-3-13 | MTIII-51 | CC MTIII-1 | CLIII-2 | CMGIII |
| 2305 | - | 2013 | Madagascar | AEZ1 | S35 | 1657 | -19,93 | 47,27 | AVRIL | 17.7 | ST | - | RS3-MLVA16 | 7-3-2-9-4-7-2-2-23-9-3-5-6-3-3-13 | MTIII-104 | CC MTIII-1 | CLIII-2 | CMGIII |
| 2306 | - | 2013 | Madagascar | AEZ1 | S35 | 1657 | -19,93 | 47,27 | AVRIL | 17.7 | ST | - | RS3-MLVA16 | 7-3-2-8-4-6-2-2-23-9-3-5-6-3-3-13 | MTIII-51 | CC MTIII-1 | CLIII-2 | CMGIII |
| 2308 | - | 2013 | Madagascar | AEZ1 | S35 | 1657 | -19,93 | 47,27 | AVRIL | 17.7 | ST | - | RS3-MLVA16 | 7-3-2-8-4-6-2-2-23-9-3-5-6-3-3-13 | MTIII-51 | CC MTIII-1 | CLIII-2 | CMGIII |
| 2309 | - | 2013 | Madagascar | AEZ1 | S35 | 1657 | -19,93 | 47,27 | AVRIL | 17.7 | ST | 60 | RS3-MLVA16 | 7-3-2-9-4-7-2-2-23-9-3-5-6-4-3-13 | MTIII-103 | CC MTIII-1 | CLIII-2 | CMGIII |
| 2310 | - | 2013 | Madagascar | AEZ1 | S35 | 1657 | -19,93 | 47,27 | AVRIL | 17.7 | PV | 60 | RS3-MLVA16 | 7-3-2-8-4-6-2-2-23-9-3-5-6-3-3-13 | MTIII-51 | CC MTIII-1 | CLIII-2 | CMGIII |
| 2311 | - | 2013 | Madagascar | AEZ1 | S37 | 1678 | -19,73 | 47,27 | AVRIL | 17.7 | ST | - | RS3-MLVA16 | 7-3-2-8-4-6-2-2-23-9-3-5-6-3-3-13 | MTIII-51 | CC MTIII-1 | CLIII-8 | CMGIII |
| 2312 | - | 2013 | Madagascar | AEZ1 | S37 | 1678 | -19,73 | 47,27 | AVRIL | 17.7 | ST | - | RS3-MLVA16 | 7-3-2-8-4-6-2-2-23-9-3-5-6-3-3-13 | MTIII-51 | CC MTIII-1 | CLIII-8 | CMGIII |
| 2318 | - | 2013 | Madagascar | AEZ1 | S33 | 1660 | -19,87 | 47,21 | AVRIL | 17.7 | ST | 19 | RS3-MLVA16 | 7-3-2-12-5-7-2-2-21-6-3-5-6-3-5-13 | MTIII-130 | CC MTIII-2 | CLIII-7 | CMGIII |
| 2320 | - | 2013 | Madagascar | AEZ1 | S33 | 1660 | -19,87 | 47,21 | AVRIL | 17.7 | ST | 19 | RS3-MLVA16 | 7-3-2-12-5-7-2-2-19-6-3-5-6-3-5-13 | MTIII-60 | CC MTIII-2 | CLIII-7 | CMGIII |
| 2322 | - | 2013 | Madagascar | AEZ1 | S33 | 1660 | -19,87 | 47,21 | AVRIL | 17.7 | ST | 60 | RS3-MLVA16 | 7-3-2-8-4-6-2-2-23-9-3-5-6-4-3-13 | MTIII-109 | CC MTIII-1 | CLIII-7 | CMGIII |
| 2327 | - | 2013 | Madagascar | AEZ1 | S33 | 1660 | -19,87 | 47,21 | AVRIL | 17.7 | ST | 60 | RS3-MLVA16 | 7-3-2-8-4-6-2-2-24-9-3-5-6-3-3-13 | MTIII-49 | CC MTIII-1 | CLIII-7 | CMGIII |
| 2329 | - | 2013 | Madagascar | AEZ1 | S33 | 1660 | -19,87 | 47,21 | AVRIL | 17.7 | ST | 60 | RS3-MLVA16 | 7-3-2-8-4-6-2-2-23-9-3-5-6-3-3-13 | MTIII-51 | CC MTIII-1 | CLIII-7 | CMGIII |
| 2330 | - | 2013 | Madagascar | AEZ1 | S33 | 1660 | -19,87 | 47,21 | AVRIL | 17.7 | ST | 60 | RS3-MLVA16 | 7-3-2-8-4-6-2-2-23-9-3-5-6-3-3-13 | MTIII-51 | CC MTIII-1 | CLIII-7 | CMGIII |
| 2334 | - | 2013 | Madagascar | AEZ1 | S33 | 1660 | -19,87 | 47,21 | AVRIL | 17.7 | ST | 19 | RS3-MLVA16 | 7-3-2-12-5-7-2-2-22-7-3-5-6-3-5-14 | MTIII-128 | CC MTIII-2 | CLIII-7 | CMGIII |
| 2335 | - | 2013 | Madagascar | AEZ1 | S33 | 1660 | -19,87 | 47,21 | AVRIL | 17.7 | ST | 19 | RS3-MLVA16 | 10-3-2-8-14-3-2-3-16-9-3-7-2-3-3-15 | MTIII-162 | - | CLIII-7 | CMGIII |
| 2340 | - | 2013 | Madagascar | AEZ1 | S34 | 1663 | -19,86 | 47,22 | AVRIL | 17.7 | ST | 58 | RS3-MLVA16 | 9-3-2-9-7-4-2-2-13-9-3-5-5-4-6-12 | MTIII-63 | - | CLIII-7 | CMGIII |
| 2344 | - | 2013 | Madagascar | AEZ1 | S34 | 1663 | -19,86 | 47,22 | AVRIL | 17.7 | ST | 58 | RS3-MLVA16 | 9-3-2-9-7-4-2-2-13-9-3-5-5-4-6-12 | MTIII-63 | - | CLIII-7 | CMGIII |
| 2345 | - | 2013 | Madagascar | AEZ1 | S34 | 1663 | -19,86 | 47,22 | AVRIL | 17.7 | ST | - | RS3-MLVA16 | 9-3-2-9-7-4-2-2-13-9-3-5-5-4-6-12 | MTIII-63 | - | CLIII-7 | CMGIII |
| 2347 | - | 2013 | Madagascar | AEZ1 | S34 | 1663 | -19,86 | 47,22 | AVRIL | 17.7 | ST | - | RS3-MLVA16 | 9-3-2-9-7-4-2-2-13-9-3-5-5-4-6-12 | MTIII-63 | - | CLIII-7 | CMGIII |
| 2349 | - | 2013 | Madagascar | AEZ1 | S34 | 1663 | -19,86 | 47,22 | AVRIL | 17.7 | ST | - | RS3-MLVA16 | 9-3-2-9-7-4-2-2-13-9-3-5-5-4-6-12 | MTIII-63 | - | CLIII-7 | CMGIII |
| 2352 | - | 2013 | Madagascar | AEZ1 | S34 | 1663 | -19,86 | 47,22 | AVRIL | 17.7 | ST | 60 | RS3-MLVA16 | 7-3-2-8-4-6-2-2-24-9-3-5-6-3-3-13 | MTIII-49 | CC MTIII-1 | CLIII-7 | CMGIII |
| 2354 | - | 2013 | Madagascar | AEZ1 | S34 | 1663 | -19,86 | 47,22 | AVRIL | 17.7 | ST | 60 | RS3-MLVA16 | 7-3-2-8-4-6-2-2-24-9-3-5-6-3-3-14 | MTIII-108 | CC MTIII-1 | CLIII-7 | CMGIII |
| 2355 | - | 2013 | Madagascar | AEZ1 | S34 | 1663 | -19,86 | 47,22 | AVRIL | 17.7 | ST | 60 | RS3-MLVA16 | 7-3-2-8-4-6-2-2-23-9-3-5-6-3-3-13 | MTIII-51 | CC MTIII-1 | CLIII-7 | CMGIII |
| 2357 | - | 2013 | Madagascar | AEZ1 | S34 | 1663 | -19,86 | 47,22 | AVRIL | 17.7 | ST | 60 | RS3-MLVA16 | 7-3-2-8-4-6-2-2-24-9-3-5-6-3-3-13 | MTIII-49 | CC MTIII-1 | CLIII-7 | CMGIII |
| 2358 | - | 2013 | Madagascar | AEZ1 | S34 | 1663 | -19,86 | 47,22 | AVRIL | 17.7 | ST | - | RS3-MLVA16 | 7-3-2-8-4-6-2-2-23-9-3-5-6-3-3-13 | MTIII-51 | CC MTIII-1 | CLIII-7 | CMGIII |
| 2370 | - | 2013 | Madagascar | AEZ1 | S44 | 1659 | -19,79 | 47,31 | AVRIL | 17.7 | ST | 19 | RS3-MLVA16 | 7-3-2-12-5-7-2-2-19-7-3-5-6-3-5-13 | MTIII-59 | CC MTIII-2 | CLIII-9 | CMGIII |
| 2372 | - | 2013 | Madagascar | AEZ1 | S44 | 1659 | -19,79 | 47,31 | AVRIL | 17.7 | ST | - | RS3-MLVA16 | 7-3-2-12-5-7-2-2-19-7-3-5-6-3-5-13 | MTIII-59 | CC MTIII-2 | CLIII-9 | CMGIII |
| 2373 | - | 2013 | Madagascar | AEZ1 | S44 | 1659 | -19,79 | 47,31 | AVRIL | 17.7 | ST | - | RS3-MLVA16 | 7-3-2-12-5-7-2-2-20-7-3-5-6-3-5-13 | MTIII-57 | CC MTIII-2 | CLIII-9 | CMGIII |
| 2374 | - | 2013 | Madagascar | AEZ1 | S44 | 1659 | -19,79 | 47,31 | AVRIL | 17.7 | ST | - | RS3-MLVA16 | 7-3-2-12-5-7-2-2-20-7-3-5-6-3-5-13 | MTIII-57 | CC MTIII-2 | CLIII-9 | CMGIII |
| 2375 | - | 2013 | Madagascar | AEZ1 | S44 | 1659 | -19,79 | 47,31 | AVRIL | 17.7 | ST | 19 | RS3-MLVA16 | 7-3-2-12-5-7-2-2-20-7-3-5-6-3-5-13 | MTIII-57 | CC MTIII-2 | CLIII-9 | CMGIII |
| 2376 | - | 2013 | Madagascar | AEZ1 | S44 | 1659 | -19,79 | 47,31 | AVRIL | 17.7 | ST | - | RS3-MLVA16 | 7-3-2-12-5-7-2-2-20-7-3-5-6-3-5-13 | MTIII-57 | CC MTIII-2 | CLIII-9 | CMGIII |
| 2377 | - | 2013 | Madagascar | AEZ1 | S44 | 1659 | -19,79 | 47,31 | AVRIL | 17.7 | ST | 19 | RS3-MLVA16 | 7-3-2-12-5-7-2-2-20-7-3-5-6-3-5-13 | MTIII-57 | CC MTIII-2 | CLIII-9 | CMGIII |
| 2378 | - | 2013 | Madagascar | AEZ1 | S44 | 1659 | -19,79 | 47,31 | AVRIL | 17.7 | ST | - | - | - | - | - | - | **-** |
| 2383 | - | 2013 | Madagascar | AEZ1 | S44 | 1659 | -19,79 | 47,31 | AVRIL | 17.7 | ST | 60 | RS3-MLVA16 | 7-3-2-10-4-6-2-2-25-9-3-5-6-3-3-12 | MTIII-143 | CC MTIII-11 | CLIII-9 | CMGIII |
| 2386 | - | 2013 | Madagascar | AEZ1 | S44 | 1659 | -19,79 | 47,31 | AVRIL | 17.7 | ST | - | RS3-MLVA16 | 7-3-2-12-5-7-2-2-20-7-3-5-6-4-5-13 | MTIII-132 | CC MTIII-2 | CLIII-9 | CMGIII |
| 2389 | - | 2013 | Madagascar | AEZ1 | S44 | 1659 | -19,79 | 47,31 | AVRIL | 17.7 | ST | - | RS3-MLVA16 | 7-3-2-12-5-8-2-2-20-6-3-5-6-3-5-13 | MTIII-126 | CC MTIII-2 | CLIII-9 | CMGIII |
| 2474 | - | 2013 | Madagascar | AEZ2 | S60 | 1604 | -19,90 | 47,05 | AVRIL | 18.3 | ST | 19 | RS3-MLVA16 | 7-3-2-12-5-7-2-2-21-7-3-5-6-3-5-14 | MTIII-55 | CC MTIII-2 | CLIII-5 | CMGIII |
| 2510 | - | 2013 | Madagascar | AEZ2 | S56 | 1599 | -19,87 | 47,02 | AVRIL | 18.3 | ST | 60 | RS3-MLVA16 | 7-3-3-12-6-4-2-2-24-9-3-7-6-4-4-14 | MTIII-102 | - | CLIII-5 | CMGIII |
| 2514 | - | 2013 | Madagascar | AEZ2 | S59 | 1605 | -19,94 | 47,08 | AVRIL | 18.3 | ST | - | - | - | - | - | - | **-** |
| 2558 | - | 2013 | Madagascar | AEZ4 | S3 | 1569 | -19,62 | 47,43 | AVRIL | 17.3 | ST | 60 | RS3-MLVA16 | 7-3-2-8-4-6-2-2-23-9-3-5-6-3-3-13 | MTIII-51 | CC MTIII-1 | CLIII-1 | CMGIII |
| 2565 | - | 2013 | Madagascar | AEZ4 | S3 | 1569 | -19,62 | 47,43 | AVRIL | 17.3 | ST | 19 | RS3-MLVA16 | 11-3-2-17-5-25-2-2-58-9-3-5-7-3-17-15 | MTIII-159 | CC MTIII-9 | CLIII-1 | CMGIII |
| 2574 | - | 2013 | Madagascar | AEZ4 | S4 | 1596 | -19,49 | 47,58 | AVRIL | 17.3 | ST | 19 | RS3-MLVA16 | 8-3-2-12-5-8-2-2-22-9-3-5-6-3-18-13 | MTIII-67 | CC MTIII-3 | CLIII-1 | CMGIII |
| 2575 | - | 2013 | Madagascar | AEZ4 | S4 | 1596 | -19,49 | 47,58 | AVRIL | 17.3 | ST | - | RS3-MLVA16 | 8-3-2-12-5-9-2-2-23-9-3-5-6-3-18-13 | MTIII-94 | CC MTIII-3 | CLIII-1 | CMGIII |
| 2576 | - | 2013 | Madagascar | AEZ4 | S4 | 1596 | -19,49 | 47,58 | AVRIL | 17.3 | ST | 60 | RS3-MLVA16 | 7-3-2-8-4-6-2-2-23-9-3-5-6-3-3-13 | MTIII-51 | CC MTIII-1 | CLIII-1 | CMGIII |
| 2577 | - | 2013 | Madagascar | AEZ4 | S4 | 1596 | -19,49 | 47,58 | AVRIL | 17.3 | ST | 19 | RS3-MLVA16 | 8-3-2-12-5-8-2-2-22-9-3-5-6-3-18-14 | MTIII-65 | CC MTIII-3 | CLIII-1 | CMGIII |
| 2578 | - | 2013 | Madagascar | AEZ4 | S4 | 1596 | -19,49 | 47,58 | AVRIL | 17.3 | ST | 19 | RS3-MLVA16 | 8-3-2-12-5-9-2-2-22-9-3-5-6-3-18-13 | MTIII-95 | CC MTIII-3 | CLIII-1 | CMGIII |
| 2579 | - | 2013 | Madagascar | AEZ4 | S4 | 1596 | -19,49 | 47,58 | AVRIL | 17.3 | ST | - | RS3-MLVA16 | 8-3-2-12-5-8-2-2-22-9-3-5-6-3-18-13 | MTIII-67 | CC MTIII-3 | CLIII-1 | CMGIII |
| 2580 | - | 2013 | Madagascar | AEZ4 | S4 | 1596 | -19,49 | 47,58 | AVRIL | 17.3 | ST | 19 | RS3-MLVA16 | 8-3-2-12-5-9-2-2-23-9-3-5-6-3-18-14 | MTIII-93 | CC MTIII-3 | CLIII-1 | CMGIII |
| 2581 | - | 2013 | Madagascar | AEZ4 | S4 | 1596 | -19,49 | 47,58 | AVRIL | 17.3 | ST | 19 | RS3-MLVA16 | 8-3-2-12-5-8-2-2-22-9-3-5-6-4-18-14 | MTIII-96 | CC MTIII-3 | CLIII-1 | CMGIII |
| 2582 | - | 2013 | Madagascar | AEZ4 | S4 | 1596 | -19,49 | 47,58 | AVRIL | 17.3 | ST | 19 | RS3-MLVA16 | 11-3-2-22-5-25-2-2-58-9-3-5-7-3-17-15 | MTIII-77 | CC MTIII-9 | CLIII-1 | CMGIII |
| 2583 | - | 2013 | Madagascar | AEZ4 | S4 | 1596 | -19,49 | 47,58 | AVRIL | 17.3 | ST | 19 | RS3-MLVA16 | 13-3-2-9-8-10-2-2-19-9-3-5-10-3-9-9 | MTIII-148 | - | CLIII-1 | CMGIII |
| 2584 | - | 2013 | Madagascar | AEZ4 | S4 | 1596 | -19,49 | 47,58 | AVRIL | 17.3 | ST | - | RS3-MLVA16 | 8-3-2-12-5-8-2-2-22-9-3-5-6-3-18-14 | MTIII-65 | CC MTIII-3 | CLIII-1 | CMGIII |
| 2585 | - | 2013 | Madagascar | AEZ4 | S4 | 1596 | -19,49 | 47,58 | AVRIL | 17.3 | ST | - | RS3-MLVA16 | 8-3-2-12-5-8-2-2-22-9-3-5-6-3-18-14 | MTIII-65 | CC MTIII-3 | CLIII-1 | CMGIII |
| 2586 | - | 2013 | Madagascar | AEZ4 | S4 | 1596 | -19,49 | 47,58 | AVRIL | 17.3 | ST | - | RS3-MLVA16 | 11-3-2-22-5-25-2-2-58-9-3-5-7-3-17-15 | MTIII-77 | CC MTIII-9 | CLIII-1 | CMGIII |
| 2587 | - | 2013 | Madagascar | AEZ4 | S4 | 1596 | -19,49 | 47,58 | AVRIL | 17.3 | ST | 19 | RS3-MLVA16 | 11-3-2-22-5-25-2-2-58-9-3-5-7-3-17-15 | MTIII-77 | CC MTIII-9 | CLIII-1 | CMGIII |
| 2592 | - | 2013 | Madagascar | AEZ4 | S5 | 1573 | -19,59 | 47,53 | AVRIL | 17.3 | ST | - | RS3-MLVA16 | 11-3-2-22-5-25-2-2-58-9-3-5-7-3-17-15 | MTIII-77 | CC MTIII-9 | CLIII-1 | CMGIII |
| 2594 | - | 2013 | Madagascar | AEZ4 | S5 | 1573 | -19,59 | 47,53 | AVRIL | 17.3 | ST | - | RS3-MLVA16 | 8-3-2-12-5-8-2-2-22-9-3-5-6-3-18-14 | MTIII-65 | CC MTIII-3 | CLIII-1 | CMGIII |
| 2706 | - | 2013 | Madagascar | AEZ3 | S66 | 1950 | -19,68 | 47,27 | AVRIL | 17.5 | ST | 19 | RS3-MLVA16 | 9-3-2-8-14-3-2-3-15-9-3-7-4-3-3-15 | MTIII-84 | CC MTIII-10 | CLIII-8 | CMGIII |
| 2709 | - | 2013 | Madagascar | AEZ3 | S66 | 1950 | -19,68 | 47,27 | AVRIL | 17.5 | ST | 19 | RS3-MLVA16 | 7-3-2-12-5-7-2-2-21-7-3-5-6-3-5-14 | MTIII-55 | CC MTIII-2 | CLIII-8 | CMGIII |
| 2712 | - | 2013 | Madagascar | AEZ3 | S66 | 1950 | -19,68 | 47,27 | AVRIL | 17.5 | ST | 19 | RS3-MLVA16 | 7-3-2-13-5-7-2-2-21-7-3-5-6-3-5-14 | MTIII-121 | CC MTIII-2 | CLIII-8 | CMGIII |
| 2713 | - | 2013 | Madagascar | AEZ3 | S66 | 1950 | -19,68 | 47,27 | AVRIL | 17.5 | ST | 19 | RS3-MLVA16 | 7-3-2-11-5-7-2-2-20-7-3-5-6-3-5-14 | MTIII-137 | CC MTIII-2 | CLIII-8 | CMGIII |
| 2714 | - | 2013 | Madagascar | AEZ3 | S66 | 1950 | -19,68 | 47,27 | AVRIL | 17.5 | ST | 19 | RS3-MLVA16 | 8-3-2-11-5-7-2-2-20-6-3-5-6-3-5-13 | MTIII-97 | - | CLIII-8 | CMGIII |
| 2715 | - | 2013 | Madagascar | AEZ3 | S66 | 1950 | -19,68 | 47,27 | AVRIL | 17.5 | ST | 19 | RS3-MLVA16 | 7-3-2-12-5-6-2-2-21-7-3-5-6-3-5-14 | MTIII-135 | CC MTIII-2 | CLIII-8 | CMGIII |
| 2716 | - | 2013 | Madagascar | AEZ3 | S66 | 1950 | -19,68 | 47,27 | AVRIL | 17.5 | ST | 60 | RS3-MLVA16 | 12-3-2-9-8-5-2-2-34-7-3-5-11-4-5-6 | MTIII-153 | - | CLIII-8 | CMGIII |
| 2717 | - | 2013 | Madagascar | AEZ3 | S66 | 1950 | -19,68 | 47,27 | AVRIL | 17.5 | ST | 60 | RS3-MLVA16 | 7-3-2-7-4-22-2-2-30-7-3-5-5-3-5-9 | MTIII-52 | CC MTIII-5 | CLIII-8 | CMGIII |
| 2718 | - | 2013 | Madagascar | AEZ3 | S66 | 1950 | -19,68 | 47,27 | AVRIL | 17.5 | ST | 60 | RS3-MLVA16 | 7-3-2-8-4-6-2-2-24-9-3-5-6-4-3-13 | MTIII-107 | CC MTIII-1 | CLIII-8 | CMGIII |
| 2719 | - | 2013 | Madagascar | AEZ3 | S66 | 1950 | -19,68 | 47,27 | AVRIL | 17.5 | ST | 19 | RS3-MLVA16 | 7-3-2-12-6-7-2-2-20-7-3-5-6-3-5-14 | MTIII-123 | CC MTIII-2 | CLIII-8 | CMGIII |
| 2720 | - | 2013 | Madagascar | AEZ3 | S66 | 1950 | -19,68 | 47,27 | AVRIL | 17.5 | ST | 19 | RS3-MLVA16 | 7-3-2-12-5-7-2-2-20-7-3-5-6-3-5-14 | MTIII-56 | CC MTIII-2 | CLIII-8 | CMGIII |
| 2721 | - | 2013 | Madagascar | AEZ3 | S66 | 1950 | -19,68 | 47,27 | AVRIL | 17.5 | ST | 19 | RS3-MLVA16 | 7-3-2-13-5-8-2-2-20-7-3-5-6-3-5-14 | MTIII-120 | CC MTIII-2 | CLIII-8 | CMGIII |
| 2722 | - | 2013 | Madagascar | AEZ3 | S66 | 1950 | -19,68 | 47,27 | AVRIL | 17.5 | ST | 60 | RS3-MLVA16 | 13-3-2-13-7-4-2-2-34-7-3-5-11-4-5-9 | MTIII-149 | - | CLIII-8 | CMGIII |
| 2725 | - | 2013 | Madagascar | AEZ3 | S66 | 1950 | -19,68 | 47,27 | AVRIL | 17.5 | ST | 19 | RS3-MLVA16 | 7-3-2-12-5-7-2-2-20-7-3-5-6-3-5-14 | MTIII-56 | CC MTIII-2 | CLIII-8 | CMGIII |
| 2726 | - | 2013 | Madagascar | AEZ3 | S66 | 1950 | -19,68 | 47,27 | AVRIL | 17.5 | ST | - | RS3-MLVA16 | 7-3-2-7-4-22-2-2-30-7-3-5-5-3-5-9 | MTIII-52 | CC MTIII-5 | CLIII-8 | CMGIII |
| 2727 | - | 2013 | Madagascar | AEZ3 | S66 | 1950 | -19,68 | 47,27 | AVRIL | 17.5 | ST | 19 | RS3-MLVA16 | 7-3-2-12-5-7-2-2-19-6-3-5-6-3-5-13 | MTIII-60 | CC MTIII-2 | CLIII-8 | CMGIII |
| 2753 | - | 2013 | Madagascar | AEZ3 | S68 | 1937 | -19,59 | 47,04 | AVRIL | 17.5 | ST | 60 | RS3-MLVA16 | 7-3-2-7-4-23-2-2-30-7-3-5-5-3-5-9 | MTIII-111 | CC MTIII-5 | CLIII-8 | CMGIII |
| 2757 | - | 2013 | Madagascar | AEZ3 | S69 | 1941 | -19,59 | 47,03 | AVRIL | 17.5 | ST | - | RS3-MLVA16 | 7-3-2-12-5-7-2-2-20-6-3-5-6-3-5-13 | MTIII-58 | CC MTIII-2 | CLIII-8 | CMGIII |
| 2758 | - | 2013 | Madagascar | AEZ3 | S69 | 1941 | -19,59 | 47,03 | AVRIL | 17.5 | ST | 60 | RS3-MLVA16 | 7-3-2-7-4-19-2-2-30-7-3-5-5-3-5-9 | MTIII-114 | CC MTIII-5 | CLIII-8 | CMGIII |
| 2759 | - | 2013 | Madagascar | AEZ3 | S69 | 1941 | -19,59 | 47,03 | AVRIL | 17.5 | ST | - | RS3-MLVA16 | 7-3-2-12-5-7-2-2-20-7-3-5-6-3-5-14 | MTIII-56 | CC MTIII-2 | CLIII-8 | CMGIII |
| 2760 | - | 2013 | Madagascar | AEZ3 | S69 | 1941 | -19,59 | 47,03 | AVRIL | 17.5 | ST | 19 | RS3-MLVA16 | 7-3-2-12-5-7-2-2-20-7-3-5-6-3-5-14 | MTIII-56 | CC MTIII-2 | CLIII-8 | CMGIII |
| 2761 | - | 2013 | Madagascar | AEZ3 | S69 | 1941 | -19,59 | 47,03 | AVRIL | 17.5 | ST | - | RS3-MLVA16 | 7-3-2-12-5-7-2-2-20-7-3-5-6-3-5-14 | MTIII-56 | CC MTIII-2 | CLIII-8 | CMGIII |
| 2762 | - | 2013 | Madagascar | AEZ3 | S69 | 1941 | -19,59 | 47,03 | AVRIL | 17.5 | ST | 19 | RS3-MLVA16 | 7-3-2-12-5-7-2-2-20-7-3-5-6-4-5-14 | MTIII-131 | CC MTIII-2 | CLIII-8 | CMGIII |
| 2763 | - | 2013 | Madagascar | AEZ3 | S69 | 1941 | -19,59 | 47,03 | AVRIL | 17.5 | ST | 19 | RS3-MLVA16 | 9-3-2-8-14-3-2-3-16-9-3-7-4-3-3-15 | MTIII-83 | CC MTIII-10 | CLIII-8 | CMGIII |
| 2767 | - | 2013 | Madagascar | AEZ3 | S69 | 1941 | -19,59 | 47,03 | AVRIL | 17.5 | ST | 19 | RS3-MLVA16 | 7-3-2-12-5-9-2-2-20-6-3-5-6-3-5-14 | MTIII-125 | - | CLIII-8 | CMGIII |
| 2770 | - | 2013 | Madagascar | AEZ3 | S68 | 1937 | -19,59 | 47,04 | AVRIL | 17.5 | ST | 60 | RS3-MLVA16 | 7-3-2-7-4-23-2-2-31-7-3-5-5-3-5-9 | MTIII-110 | CC MTIII-5 | CLIII-8 | CMGIII |
| 2771 | - | 2013 | Madagascar | AEZ3 | S68 | 1937 | -19,59 | 47,04 | AVRIL | 17.5 | ST | 19 | RS3-MLVA16 | 7-3-2-14-5-7-2-2-20-6-3-5-6-3-4-13 | MTIII-119 | - | CLIII-8 | CMGIII |
| 2774 | - | 2013 | Madagascar | AEZ3 | S68 | 1937 | -19,59 | 47,04 | AVRIL | 17.5 | ST | - | RS3-MLVA16 | 7-3-2-12-5-7-2-2-20-6-3-5-6-3-5-13 | MTIII-58 | CC MTIII-2 | CLIII-8 | CMGIII |
| 2776 | - | 2013 | Madagascar | AEZ3 | S68 | 1937 | -19,59 | 47,04 | AVRIL | 17.5 | ST | 60 | RS3-MLVA16 | 7-3-2-7-4-21-2-2-31-8-3-5-5-3-5-9 | MTIII-113 | CC MTIII-5 | CLIII-8 | CMGIII |
| 2784 | - | 2013 | Madagascar | AEZ3 | S68 | 1937 | -19,59 | 47,04 | AVRIL | 17.5 | ST | - | RS3-MLVA16 | 7-3-2-13-5-7-2-2-20-7-3-5-6-3-5-14 | MTIII-53 | CC MTIII-2 | CLIII-8 | CMGIII |
| 2785 | - | 2013 | Madagascar | AEZ3 | S68 | 1937 | -19,59 | 47,04 | AVRIL | 17.5 | ST | - | RS3-MLVA16 | 7-3-2-7-4-22-2-2-30-8-3-5-5-3-5-9 | MTIII-112 | CC MTIII-5 | CLIII-8 | CMGIII |
| 2786 | - | 2013 | Madagascar | AEZ3 | S68 | 1937 | -19,59 | 47,04 | AVRIL | 17.5 | ST | 19 | RS3-MLVA16 | 11-3-3-7-6-6-2-2-19-9-3-5-13-4-8-12 | MTIII-154 | - | CLIII-8 | CMGIII |
| 2897 | - | 2013 | Madagascar | AEZ1 | S32 | 1797 | -19,95 | 47,21 | AVRIL | 17.7 | ST | 19 | RS3-MLVA16 | 7-3-2-13-5-7-2-2-20-7-3-5-6-3-5-14 | MTIII-53 | CC MTIII-2 | CLIII-7 | CMGIII |
| 2910 | - | 2013 | Madagascar | AEZ1 | S32 | 1797 | -19,95 | 47,21 | AVRIL | 17.7 | ST | 19 | RS3-MLVA16 | 7-3-2-13-5-7-2-2-20-7-3-5-6-3-5-14 | MTIII-53 | CC MTIII-2 | CLIII-7 | CMGIII |
| 2922 | - | 2013 | Madagascar | AEZ1 | S42 | 1392 | -20,07 | 47,26 | AVRIL | 17.7 | SL | 60 | RS3-MLVA16 | 7-3-2-8-4-6-2-2-23-9-3-5-6-3-3-13 | MTIII-51 | CC MTIII-1 | CLIII-3 | CMGIII |
| 2925 | - | 2013 | Madagascar | AEZ1 | S42 | 1392 | -20,07 | 47,26 | AVRIL | 17.7 | ST | 19 | RS3-MLVA16 | 11-3-3-6-6-3-2-2-18-9-3-5-12-4-12-9 | MTIII-155 | - | CLIII-3 | CMGIII |
| 2963 | - | 2013 | Madagascar | AEZ1 | S39 | 1357 | -20,07 | 47,29 | AVRIL | 17.7 | ST | 60 | RS3-MLVA16 | 7-3-2-8-4-6-2-2-23-9-3-5-6-3-4-13 | MTIII-50 | CC MTIII-1 | CLIII-3 | CMGIII |
| 2967 | - | 2013 | Madagascar | AEZ1 | S39 | 1357 | -20,07 | 47,29 | AVRIL | 17.7 | ST | - | RS3-MLVA16 | 7-3-2-8-4-6-2-2-23-9-3-5-6-3-4-13 | MTIII-50 | CC MTIII-1 | CLIII-3 | CMGIII |
| 2975 | - | 2013 | Madagascar | AEZ1 | S39 | 1357 | -20,07 | 47,29 | AVRIL | 17.7 | ST | 19 | RS3-MLVA16 | 10-3-2-10-5-15-2-2-45-9-3-5-6-3-14-16 | MTIII-152 | CC MTIII-8 | CLIII-3 | CMGIII |
| 2976 | - | 2013 | Madagascar | AEZ1 | S39 | 1357 | -20,07 | 47,29 | AVRIL | 17.7 | ST | 60 | RS3-MLVA16 | 7-3-2-8-4-6-2-2-23-9-3-5-6-3-4-13 | MTIII-50 | CC MTIII-1 | CLIII-3 | CMGIII |
| 2977 | - | 2013 | Madagascar | AEZ1 | S39 | 1357 | -20,07 | 47,29 | AVRIL | 17.7 | ST | 19 | RS3-MLVA16 | 10-3-2-10-5-15-2-2-45-9-3-5-6-3-15-16 | MTIII-163 | CC MTIII-8 | CLIII-3 | CMGIII |
| 2979 | - | 2013 | Madagascar | AEZ1 | S25 | 1650 | -19,95 | 47,21 | AVRIL | 17.7 | W | 19 | RS3-MLVA16 | 7-3-2-13-4-11-2-2-21-9-3-5-6-4-18-10 | MTIII-54 | - | CLIII-7 | CMGIII |
| 2981 | - | 2013 | Madagascar | AEZ1 | S25 | 1650 | -19,95 | 47,21 | AVRIL | 17.7 | W | 19 | RS3-MLVA16 | 7-3-2-13-4-11-2-2-21-9-3-5-6-4-18-10 | MTIII-54 | - | CLIII-7 | CMGIII |
| 2983 | - | 2013 | Madagascar | AEZ1 | S25 | 1650 | -19,95 | 47,21 | AVRIL | 17.7 | W | - | RS3-MLVA16 | 7-3-2-13-4-11-2-2-21-9-3-5-6-4-18-10 | MTIII-54 | - | CLIII-7 | CMGIII |
| 2984 | - | 2013 | Madagascar | AEZ1 | S25 | 1650 | -19,95 | 47,21 | AVRIL | 17.7 | W | 19 | RS3-MLVA16 | 7-3-2-13-4-11-2-2-21-9-3-5-6-4-18-10 | MTIII-54 | - | CLIII-7 | CMGIII |
| 2985 | - | 2013 | Madagascar | AEZ1 | S25 | 1650 | -19,95 | 47,21 | AVRIL | 17.7 | W | 19 | RS3-MLVA16 | 7-3-2-13-4-11-2-2-21-9-3-5-6-5-17-10 | MTIII-122 | - | CLIII-7 | CMGIII |
| 2986 | - | 2013 | Madagascar | AEZ1 | S33 | 1660 | -19,87 | 47,21 | AVRIL | 17.7 | ST | 60 | RS3-MLVA16 | 7-3-2-8-4-6-2-2-23-9-3-5-6-3-3-13 | MTIII-51 | CC MTIII-1 | CLIII-7 | CMGIII |
| 2987 | - | 2013 | Madagascar | AEZ1 | S33 | 1660 | -19,87 | 47,21 | AVRIL | 17.7 | ST | 60 | RS3-MLVA16 | 8-3-2-8-4-6-2-2-23-9-3-5-6-3-3-13 | MTIII-86 | CC MTIII-1 | CLIII-7 | CMGIII |
| 2988 | - | 2013 | Madagascar | AEZ1 | S33 | 1660 | -19,87 | 47,21 | AVRIL | 17.7 | ST | 19 | RS3-MLVA16 | 7-3-2-12-5-7-2-2-20-7-3-5-6-3-5-14 | MTIII-56 | CC MTIII-2 | CLIII-7 | CMGIII |
| 2990 | - | 2013 | Madagascar | AEZ1 | S44 | 1659 | -19,79 | 47,31 | AVRIL | 17.7 | ST | 19 | RS3-MLVA16 | 7-3-2-12-5-7-2-2-19-6-3-5-6-4-5-13 | MTIII-134 | CC MTIII-2 | CLIII-9 | CMGIII |
| 2994 | - | 2013 | Madagascar | AEZ12 | S24 | 1550 | -18,85 | 47,72 | AVRIL | 17.0 | ST | - | - | - | - | - | - | **-** |
| 2995 | - | 2013 | Madagascar | AEZ12 | S24 | 1550 | -18,85 | 47,72 | AVRIL | 17.0 | ST | 19 | RS3-MLVA16 | 7-3-2-12-5-26-2-2-19-7-3-5-7-3-4-14 | MTIII-136 | - | CLIII-4 | CMGIII |
| 3053 | - | 2013 | Madagascar | AEZ7 | S49 | 1476 | -19,07 | 47,20 | DECEMBER | 21.9 | ST | 19 | RS3-MLVA16 | 11-3-3-9-7-31-2-2-19-9-3-5-10-3-5-9 | MTIII-72 | - | CLIII-4 | CMGIII |
| 3057 | - | 2013 | Madagascar | AEZ7 | S49 | 1476 | -19,07 | 47,20 | DECEMBER | 21.9 | ST | 19 | RS3-MLVA16 | 11-3-3-9-7-31-2-2-19-9-3-5-10-3-5-9 | MTIII-72 | - | CLIII-4 | CMGIII |
| 3070 | - | 2013 | Madagascar | AEZ7 | S49 | 1476 | -19,07 | 47,20 | DECEMBER | 21.9 | ST | 19 | RS3-MLVA16 | 7-3-2-14-6-20-2-2-28-9-3-5-6-3-18-11 | MTIII-118 | - | CLIII-4 | CMGIII |
| 3192 | - | 2013 | Madagascar | AEZ7 | S50 | 1433 | -19,09 | 47,25 | DECEMBER | 21.9 | ST | 19 | - | - | - | - | - | **-** |
| 3230 | - | 2013 | Madagascar | AEZ8 | S70 | 1176 | -18,98 | 46,72 | DECEMBER | 21.3 | ST | 60 | RS3-MLVA16 | 8-3-2-12-6-4-2-2-30-9-3-7-6-3-7-11 | MTIII-89 | CC MTIII-4 | CLIII-11 | CMGIII |
| 3231 | - | 2013 | Madagascar | AEZ8 | S70 | 1176 | -18,98 | 46,72 | DECEMBER | 21.3 | ST | 60 | RS3-MLVA16 | 8-3-2-13-6-4-2-2-28-9-3-7-6-3-7-11 | MTIII-88 | CC MTIII-4 | CLIII-11 | CMGIII |
| 3235 | - | 2013 | Madagascar | AEZ8 | S70 | 1176 | -18,98 | 46,72 | DECEMBER | 21.3 | ST | 60 | RS3-MLVA16 | 8-3-2-12-6-4-2-2-28-9-3-7-6-3-7-11 | MTIII-92 | CC MTIII-4 | CLIII-11 | CMGIII |
| 3236 | - | 2013 | Madagascar | AEZ8 | S70 | 1176 | -18,98 | 46,72 | DECEMBER | 21.3 | ST | 60 | RS3-MLVA16 | 8-3-2-12-6-4-2-2-29-9-3-7-6-3-7-11 | MTIII-91 | CC MTIII-4 | CLIII-11 | CMGIII |
| 3237 | - | 2013 | Madagascar | AEZ8 | S70 | 1176 | -18,98 | 46,72 | DECEMBER | 21.3 | ST | 60 | RS3-MLVA16 | 7-3-2-7-4-17-2-2-27-7-3-5-5-3-5-9 | MTIII-116 | - | CLIII-11 | CMGIII |
| 3239 | - | 2013 | Madagascar | AEZ8 | S70 | 1176 | -18,98 | 46,72 | DECEMBER | 21.3 | ST | 60 | RS3-MLVA16 | 8-3-2-12-6-4-2-2-30-9-3-5-6-3-7-11 | MTIII-90 | CC MTIII-4 | CLIII-11 | CMGIII |
| 3241 | - | 2013 | Madagascar | AEZ8 | S71 | 1194 | -18,98 | 46,73 | DECEMBER | 21.3 | CA | 60 | RS3-MLVA16 | 7-3-2-7-4-18-2-2-27-7-3-5-5-3-4-8 | MTIII-115 | - | CLIII-11 | CMGIII |
| 3242 | - | 2013 | Madagascar | AEZ8 | S71 | 1194 | -18,98 | 46,73 | DECEMBER | 21.3 | CA | 60 | RS3-MLVA16 | 7-3-2-7-4-15-2-2-28-7-3-5-5-4-5-9 | MTIII-117 | - | CLIII-11 | CMGIII |
| 3244 | - | 2013 | Madagascar | AEZ8 | S71 | 1194 | -18,98 | 46,73 | DECEMBER | 21.3 | CA | 60 | RS3-MLVA16 | 6-3-2-7-4-17-2-2-28-7-3-5-5-3-5-9 | MTIII-146 | - | CLIII-11 | CMGIII |
| 3296 | - | 2013 | Madagascar | AEZ10 | S22 | 1720 | -20,79 | 47,18 | DECEMBER | 20.3 | ST | 19 | RS3-MLVA16 | 7-3-2-8-5-7-2-2-20-7-3-5-6-3-5-14 | MTIII-106 | CC MTIII-2 | CLIII-5 | CMGIII |
| 3302 | - | 2013 | Madagascar | AEZ10 | S21 | 1709 | -20,79 | 47,18 | DECEMBER | 20.3 | ST | 60 | RS3-MLVA16 | 7-3-2-10-4-6-2-2-30-9-3-5-6-3-3-13 | MTIII-141 | CC MTIII-1 | CLIII-5 | CMGIII |
| 3303 | - | 2013 | Madagascar | AEZ10 | S21 | 1709 | -20,79 | 47,18 | DECEMBER | 20.3 | ST | 19 | RS3-MLVA16 | 12-3-3-8-6-4-2-2-19-9-3-5-6-4-5-10 | MTIII-150 | - | CLIII-5 | CMGIII |
| 3304 | - | 2013 | Madagascar | AEZ10 | S21 | 1709 | -20,79 | 47,18 | DECEMBER | 20.3 | ST | 59 | RS3-MLVA16 | 9-3-2-11-11-7-2-2-13-9-3-7-6-3-5-11 | MTIII-85 | - | CLIII-5 | CMGIII |
| 3311 | - | 2013 | Madagascar | AEZ10 | S23 | 1725 | -20,79 | 47,18 | DECEMBER | 20.3 | ST | - | RS3-MLVA16 | 7-3-3-13-5-3-2-2-25-9-3-7-6-4-4-13 | MTIII-69 | CC MTIII-6 | CLIII-5 | CMGIII |
| 3312 | - | 2013 | Madagascar | AEZ10 | S23 | 1725 | -20,79 | 47,18 | DECEMBER | 20.3 | ST | 60 | RS3-MLVA16 | 7-3-2-9-4-6-2-2-29-9-3-5-6-3-3-13 | MTIII-74 | CC MTIII-1 | CLIII-5 | CMGIII |
| 3314 | - | 2013 | Madagascar | AEZ10 | S23 | 1725 | -20,79 | 47,18 | DECEMBER | 20.3 | ST | 60 | RS3-MLVA16 | 7-3-2-9-4-6-2-2-29-9-3-5-6-3-3-13 | MTIII-74 | CC MTIII-1 | CLIII-5 | CMGIII |
| 3316 | - | 2013 | Madagascar | AEZ10 | S23 | 1725 | -20,79 | 47,18 | DECEMBER | 20.3 | ST | - | RS3-MLVA16 | 7-3-3-13-5-3-2-2-25-9-3-7-6-4-4-13 | MTIII-69 | CC MTIII-6 | CLIII-5 | CMGIII |
| 3317 | - | 2013 | Madagascar | AEZ10 | S23 | 1725 | -20,79 | 47,18 | DECEMBER | 20.3 | ST | - | RS3-MLVA16 | 7-3-2-12-5-7-2-2-20-6-3-5-6-3-5-13 | MTIII-58 | CC MTIII-2 | CLIII-5 | CMGIII |
| 3321 | - | 2013 | Madagascar | AEZ10 | S23 | 1725 | -20,79 | 47,18 | DECEMBER | 20.3 | ST | 60 | RS3-MLVA16 | 7-3-3-13-5-3-2-2-26-9-3-7-5-4-4-13 | MTIII-100 | CC MTIII-6 | CLIII-5 | CMGIII |
| 3324 | - | 2013 | Madagascar | AEZ10 | S23 | 1725 | -20,79 | 47,18 | DECEMBER | 20.3 | P | 60 | RS3-MLVA16 | 7-3-3-13-5-3-2-2-25-9-3-7-6-4-4-13 | MTIII-69 | CC MTIII-6 | CLIII-5 | CMGIII |
| 3325 | - | 2013 | Madagascar | AEZ10 | S17 | 1634 | -20,82 | 47,18 | DECEMBER | 20.3 | ST | - | RS3-MLVA16 | 7-3-2-9-4-6-2-2-23-9-3-5-6-4-3-13 | MTIII-80 | CC MTIII-1 | CLIII-5 | CMGIII |
| 3326 | - | 2013 | Madagascar | AEZ10 | S18 | 1637 | -20,82 | 47,18 | DECEMBER | 20.3 | ST | - | RS3-MLVA16 | 7-3-2-9-4-6-2-2-23-9-3-5-6-3-3-13 | MTIII-82 | CC MTIII-1 | CLIII-5 | CMGIII |
| 3327 | - | 2013 | Madagascar | AEZ10 | S18 | 1637 | -20,82 | 47,18 | DECEMBER | 20.3 | ST | - | RS3-MLVA16 | 7-3-2-9-4-6-2-2-23-9-3-5-6-3-3-13 | MTIII-82 | CC MTIII-1 | CLIII-5 | CMGIII |
| 3328 | - | 2013 | Madagascar | AEZ10 | S18 | 1637 | -20,82 | 47,18 | DECEMBER | 20.3 | ST | - | RS3-MLVA16 | 7-3-2-9-4-6-2-2-23-9-3-5-6-3-3-13 | MTIII-82 | CC MTIII-1 | CLIII-5 | CMGIII |
| 3329 | - | 2013 | Madagascar | AEZ10 | S18 | 1637 | -20,82 | 47,18 | DECEMBER | 20.3 | ST | - | RS3-MLVA16 | 7-3-2-9-4-6-2-2-24-9-3-5-6-3-3-13 | MTIII-78 | CC MTIII-1 | CLIII-5 | CMGIII |
| 3330 | - | 2013 | Madagascar | AEZ10 | S18 | 1637 | -20,82 | 47,18 | DECEMBER | 20.3 | ST | 60 | RS3-MLVA16 | 7-3-2-9-4-6-2-2-23-9-3-5-6-3-3-13 | MTIII-82 | CC MTIII-1 | CLIII-5 | CMGIII |
| 3331 | - | 2013 | Madagascar | AEZ10 | S18 | 1637 | -20,82 | 47,18 | DECEMBER | 20.3 | ST | 60 | RS3-MLVA16 | 7-3-2-9-4-6-2-2-23-9-3-5-6-4-3-13 | MTIII-80 | CC MTIII-1 | CLIII-5 | CMGIII |
| 3334 | - | 2013 | Madagascar | AEZ10 | S19 | 1642 | -20,82 | 47,18 | DECEMBER | 20.3 | ST | 60 | RS3-MLVA16 | 7-3-2-9-4-6-2-2-24-9-3-5-6-3-3-12 | MTIII-79 | CC MTIII-1 | CLIII-5 | CMGIII |
| 3335 | - | 2013 | Madagascar | AEZ10 | S19 | 1642 | -20,82 | 47,18 | DECEMBER | 20.3 | ST | 60 | RS3-MLVA16 | 7-3-2-9-4-6-2-2-23-9-3-5-6-3-3-13 | MTIII-82 | CC MTIII-1 | CLIII-5 | CMGIII |
| 3336 | - | 2013 | Madagascar | AEZ10 | S19 | 1642 | -20,82 | 47,18 | DECEMBER | 20.3 | PV | - | RS3-MLVA16 | 7-3-2-9-4-6-2-2-23-9-3-5-6-3-3-13 | MTIII-82 | CC MTIII-1 | CLIII-5 | CMGIII |
| 3339 | - | 2013 | Madagascar | AEZ10 | S14 | 1629 | -20,83 | 47,18 | DECEMBER | 20.3 | ST | - | RS3-MLVA16 | 7-3-2-9-4-6-2-2-24-9-3-5-6-3-3-13 | MTIII-78 | CC MTIII-1 | CLIII-5 | CMGIII |
| 3340 | - | 2013 | Madagascar | AEZ10 | S14 | 1629 | -20,83 | 47,18 | DECEMBER | 20.3 | ST | 60 | RS3-MLVA16 | 7-3-3-13-5-3-2-2-24-9-3-7-6-4-4-13 | MTIII-71 | CC MTIII-6 | CLIII-5 | CMGIII |
| 3341 | - | 2013 | Madagascar | AEZ10 | S14 | 1629 | -20,83 | 47,18 | DECEMBER | 20.3 | ST | - | RS3-MLVA16 | 7-3-2-9-4-6-2-2-24-9-3-5-6-3-3-12 | MTIII-79 | CC MTIII-1 | CLIII-5 | CMGIII |
| 3342 | - | 2013 | Madagascar | AEZ10 | S14 | 1629 | -20,83 | 47,18 | DECEMBER | 20.3 | ST | - | RS3-MLVA16 | 7-3-2-9-4-6-2-2-24-9-3-5-6-4-3-13 | MTIII-76 | CC MTIII-1 | CLIII-5 | CMGIII |
| 3343 | - | 2013 | Madagascar | AEZ10 | S14 | 1629 | -20,83 | 47,18 | DECEMBER | 20.3 | ST | - | RS3-MLVA16 | 7-3-2-9-4-6-2-2-24-9-3-5-6-3-3-13 | MTIII-78 | CC MTIII-1 | CLIII-5 | CMGIII |
| 3344 | - | 2013 | Madagascar | AEZ10 | S14 | 1629 | -20,83 | 47,18 | DECEMBER | 20.3 | ST | - | RS3-MLVA16 | 7-3-2-9-4-6-2-2-24-9-3-5-6-4-3-13 | MTIII-76 | CC MTIII-1 | CLIII-5 | CMGIII |
| 3345 | - | 2013 | Madagascar | AEZ10 | S14 | 1629 | -20,83 | 47,18 | DECEMBER | 20.3 | ST | - | RS3-MLVA16 | 7-3-2-9-4-6-2-2-24-9-3-5-6-3-3-13 | MTIII-78 | CC MTIII-1 | CLIII-5 | CMGIII |
| 3346 | - | 2013 | Madagascar | AEZ10 | S14 | 1629 | -20,83 | 47,18 | DECEMBER | 20.3 | ST | - | RS3-MLVA16 | 7-3-3-13-5-3-2-2-24-9-3-7-6-4-4-13 | MTIII-71 | CC MTIII-6 | CLIII-5 | CMGIII |
| 3347 | - | 2013 | Madagascar | AEZ10 | S14 | 1629 | -20,83 | 47,18 | DECEMBER | 20.3 | ST | - | RS3-MLVA16 | 7-3-2-9-4-6-2-2-24-9-3-5-6-3-3-13 | MTIII-78 | CC MTIII-1 | CLIII-5 | CMGIII |
| 3348 | - | 2013 | Madagascar | AEZ10 | S14 | 1629 | -20,83 | 47,18 | DECEMBER | 20.3 | ST | 60 | RS3-MLVA16 | 7-3-2-9-4-6-2-2-24-9-3-5-6-3-3-14 | MTIII-105 | CC MTIII-1 | CLIII-5 | CMGIII |
| 3349 | - | 2013 | Madagascar | AEZ10 | S14 | 1629 | -20,83 | 47,18 | DECEMBER | 20.3 | ST | - | RS3-MLVA16 | 7-3-2-9-4-6-2-2-24-9-3-5-6-3-3-13 | MTIII-78 | CC MTIII-1 | CLIII-5 | CMGIII |
| 3355 | - | 2013 | Madagascar | AEZ10 | S13 | 1579 | -20,83 | 47,19 | DECEMBER | 20.3 | ST | - | RS3-MLVA16 | 7-3-3-13-5-3-2-2-24-9-3-7-6-4-4-13 | MTIII-71 | CC MTIII-6 | CLIII-5 | CMGIII |
| 3356 | - | 2013 | Madagascar | AEZ10 | S13 | 1579 | -20,83 | 47,19 | DECEMBER | 20.3 | ST | 60 | RS3-MLVA16 | 7-3-3-13-5-3-2-2-24-9-3-7-6-4-3-13 | MTIII-73 | CC MTIII-6 | CLIII-5 | CMGIII |
| 3358 | - | 2013 | Madagascar | AEZ10 | S13 | 1579 | -20,83 | 47,19 | DECEMBER | 20.3 | ST | - | RS3-MLVA16 | 7-3-3-13-5-3-2-2-24-9-3-7-6-4-3-13 | MTIII-73 | CC MTIII-6 | CLIII-5 | CMGIII |
| 3360 | - | 2013 | Madagascar | AEZ10 | S13 | 1579 | -20,83 | 47,19 | DECEMBER | 20.3 | ST | 60 | RS3-MLVA16 | 7-3-3-13-5-3-2-2-24-9-3-7-6-4-4-13 | MTIII-71 | CC MTIII-6 | CLIII-5 | CMGIII |
| 3361 | - | 2013 | Madagascar | AEZ10 | S13 | 1579 | -20,83 | 47,19 | DECEMBER | 20.3 | ST | 60 | RS3-MLVA16 | 7-3-3-13-5-3-2-2-23-9-3-7-6-4-3-13 | MTIII-101 | CC MTIII-11 | CLIII-5 | CMGIII |
| 3362 | - | 2013 | Madagascar | AEZ10 | S16 | 1641 | -20,84 | 47,18 | DECEMBER | 20.3 | P | 60 | RS3-MLVA16 | 7-3-2-10-4-6-2-2-24-9-3-5-6-3-3-13 | MTIII-66 | CC MTIII-1 | CLIII-5 | CMGIII |
| 3363 | - | 2013 | Madagascar | AEZ10 | S16 | 1641 | -20,84 | 47,18 | DECEMBER | 20.3 | P | - | RS3-MLVA16 | 7-3-2-10-4-6-2-2-23-9-3-5-6-3-3-13 | MTIII-68 | CC MTIII-1 | CLIII-5 | CMGIII |
| 3364 | - | 2013 | Madagascar | AEZ10 | S16 | 1641 | -20,84 | 47,18 | DECEMBER | 20.3 | P | 60 | RS3-MLVA16 | 7-3-2-10-4-6-2-2-24-9-3-5-6-3-2-13 | MTIII-145 | CC MTIII-1 | CLIII-5 | CMGIII |
| 3365 | - | 2013 | Madagascar | AEZ10 | S16 | 1641 | -20,84 | 47,18 | DECEMBER | 20.3 | P | 60 | RS3-MLVA16 | 7-3-2-10-4-7-2-2-23-9-3-5-6-3-3-13 | MTIII-140 | CC MTIII-1 | CLIII-5 | CMGIII |
| 3366 | - | 2013 | Madagascar | AEZ10 | S16 | 1641 | -20,84 | 47,18 | DECEMBER | 20.3 | P | 60 | RS3-MLVA16 | 7-3-2-10-4-6-2-2-24-9-3-5-6-4-3-13 | MTIII-144 | CC MTIII-1 | CLIII-5 | CMGIII |
| 3367 | - | 2013 | Madagascar | AEZ10 | S16 | 1641 | -20,84 | 47,18 | DECEMBER | 20.3 | P | - | RS3-MLVA16 | 7-3-2-10-4-6-2-2-24-9-3-5-6-3-3-13 | MTIII-66 | CC MTIII-1 | CLIII-5 | CMGIII |
| 3368 | - | 2013 | Madagascar | AEZ10 | S16 | 1641 | -20,84 | 47,18 | DECEMBER | 20.3 | P | 60 | RS3-MLVA16 | 7-3-2-10-4-6-2-2-24-9-3-5-6-3-3-13 | MTIII-66 | CC MTIII-1 | CLIII-5 | CMGIII |
| 3375 | - | 2013 | Madagascar | AEZ11 | S10 | 1442 | -20,97 | 47,14 | DECEMBER | 19.9 | ST | 19 | RS3-MLVA16 | 7-3-3-18-6-3-2-2-24-9-3-7-6-4-4-15 | MTIII-98 | - | CLIII-6 | CMGIII |
| 3377 | - | 2013 | Madagascar | AEZ11 | S10 | 1442 | -20,97 | 47,14 | DECEMBER | 19.9 | ST | 19 | RS3-MLVA16 | 7-3-3-17-6-3-2-2-24-9-3-7-6-4-3-15 | MTIII-99 | - | CLIII-6 | CMGIII |
| 3380 | - | 2013 | Madagascar | AEZ11 | S11 | 1338 | -20,88 | 47,17 | DECEMBER | 19.9 | SL | - | RS3-MLVA16 | 7-3-2-10-4-6-2-2-22-9-3-5-6-3-3-13 | MTIII-70 | CC MTIII-1 | CLIII-10 | CMGIII |
| 3381 | - | 2013 | Madagascar | AEZ11 | S11 | 1338 | -20,88 | 47,17 | DECEMBER | 19.9 | SL | 60 | RS3-MLVA16 | 7-3-2-10-4-6-2-2-22-9-3-5-6-3-3-13 | MTIII-70 | CC MTIII-1 | CLIII-10 | CMGIII |
| 3383 | - | 2013 | Madagascar | AEZ11 | S11 | 1338 | -20,88 | 47,17 | DECEMBER | 19.9 | SN | 60 | RS3-MLVA16 | 7-3-2-11-4-6-2-2-27-9-3-5-6-3-3-13 | MTIII-61 | CC MTIII-1 | CLIII-10 | CMGIII |
| 3385 | - | 2013 | Madagascar | AEZ11 | S11 | 1338 | -20,88 | 47,17 | DECEMBER | 19.9 | ST | - | RS3-MLVA16 | 7-3-2-10-4-6-2-2-27-9-3-5-6-3-3-13 | MTIII-62 | CC MTIII-1 | CLIII-10 | CMGIII |
| 3387 | - | 2013 | Madagascar | AEZ11 | S11 | 1338 | -20,88 | 47,17 | DECEMBER | 19.9 | BP | 60 | RS3-MLVA16 | 7-3-2-10-4-6-2-2-23-9-3-5-6-3-3-13 | MTIII-68 | CC MTIII-1 | CLIII-10 | CMGIII |
| 3389 | - | 2013 | Madagascar | AEZ11 | S11 | 1338 | -20,88 | 47,17 | DECEMBER | 19.9 | BP | - | RS3-MLVA16 | 7-3-2-10-4-6-2-2-23-9-3-5-6-3-3-13 | MTIII-68 | CC MTIII-1 | CLIII-10 | CMGIII |
| 3390 | - | 2013 | Madagascar | AEZ11 | S11 | 1338 | -20,88 | 47,17 | DECEMBER | 19.9 | BP | 60 | RS3-MLVA16 | 7-3-2-10-4-6-2-2-27-9-3-5-6-3-3-13 | MTIII-62 | CC MTIII-1 | CLIII-10 | CMGIII |
| 3391 | - | 2013 | Madagascar | AEZ11 | S11 | 1338 | -20,88 | 47,17 | DECEMBER | 19.9 | BP | - | RS3-MLVA16 | 7-3-2-10-4-6-2-2-23-9-3-5-6-3-3-13 | MTIII-68 | CC MTIII-1 | CLIII-10 | CMGIII |
| 3392 | - | 2013 | Madagascar | AEZ11 | S12 | 1351 | -20,88 | 47,17 | DECEMBER | 19.9 | SL | - | RS3-MLVA16 | 7-3-2-10-4-6-2-2-23-9-3-5-6-3-3-13 | MTIII-68 | CC MTIII-1 | CLIII-10 | CMGIII |
| 3395 | - | 2013 | Madagascar | AEZ11 | S12 | 1351 | -20,88 | 47,17 | DECEMBER | 19.9 | SL | - | RS3-MLVA16 | 7-3-2-10-4-6-2-2-23-9-3-5-6-3-3-13 | MTIII-68 | CC MTIII-1 | CLIII-10 | CMGIII |
| 3399 | - | 2013 | Madagascar | AEZ11 | S12 | 1351 | -20,88 | 47,17 | DECEMBER | 19.9 | SL | 60 | RS3-MLVA16 | 7-3-2-10-4-6-2-2-22-9-3-5-6-3-3-13 | MTIII-70 | CC MTIII-1 | CLIII-10 | CMGIII |
| 3401 | - | 2013 | Madagascar | AEZ11 | S12 | 1351 | -20,88 | 47,17 | DECEMBER | 19.9 | BP | - | RS3-MLVA16 | 7-3-2-10-4-6-2-2-23-9-3-5-6-3-3-13 | MTIII-68 | CC MTIII-1 | CLIII-10 | CMGIII |
| 3402 | - | 2013 | Madagascar | AEZ11 | S12 | 1351 | -20,88 | 47,17 | DECEMBER | 19.9 | SL | - | RS3-MLVA16 | 7-3-2-10-4-6-2-2-27-9-3-5-6-3-3-13 | MTIII-62 | CC MTIII-1 | CLIII-10 | CMGIII |
| 3403 | - | 2013 | Madagascar | AEZ11 | S12 | 1351 | -20,88 | 47,17 | DECEMBER | 19.9 | SL | 60 | RS3-MLVA16 | 7-3-2-10-4-6-2-2-28-9-3-5-6-3-3-13 | MTIII-142 | CC MTIII-1 | CLIII-10 | CMGIII |
| 3404 | - | 2013 | Madagascar | AEZ11 | S12 | 1351 | -20,88 | 47,17 | DECEMBER | 19.9 | SL | 60 | RS3-MLVA16 | 6-3-2-11-4-6-2-2-27-9-3-5-6-3-3-13 | MTIII-147 | CC MTIII-1 | CLIII-10 | CMGIII |
| 3405 | - | 2013 | Madagascar | AEZ11 | S12 | 1351 | -20,88 | 47,17 | DECEMBER | 19.9 | SL | - | RS3-MLVA16 | 7-3-2-10-4-6-2-2-27-9-3-5-6-3-3-13 | MTIII-62 | CC MTIII-1 | CLIII-10 | CMGIII |
| 3407 | - | 2013 | Madagascar | AEZ11 | S12 | 1351 | -20,88 | 47,17 | DECEMBER | 19.9 | SL | - | RS3-MLVA16 | 7-3-2-11-4-6-2-2-27-9-3-5-6-3-3-13 | MTIII-61 | CC MTIII-1 | CLIII-10 | CMGIII |
| 3409 | - | 2013 | Madagascar | AEZ11 | S12 | 1351 | -20,88 | 47,17 | DECEMBER | 19.9 | SL | - | RS3-MLVA16 | 7-3-2-10-4-6-2-2-27-9-3-5-6-3-3-13 | MTIII-62 | CC MTIII-1 | CLIII-10 | CMGIII |
| 3411 | - | 2013 | Madagascar | AEZ11 | S12 | 1351 | -20,88 | 47,17 | DECEMBER | 19.9 | ST | 60 | RS3-MLVA16 | 7-3-2-10-4-6-2-2-23-9-3-5-6-3-3-13 | MTIII-68 | CC MTIII-1 | CLIII-10 | CMGIII |
| 3412 | - | 2013 | Madagascar | AEZ7 | S47 | 1379 | -19,08 | 47,17 | DECEMBER | 21.9 | ST | 19 | RS3-MLVA16 | 11-3-2-21-5-25-2-2-59-9-3-5-7-3-17-15 | MTIII-158 | - | CLIII-4 | CMGIII |
| 3414 | - | 2013 | Madagascar | AEZ7 | S47 | 1379 | -19,08 | 47,17 | DECEMBER | 21.9 | ST | 19 | RS3-MLVA16 | 11-3-2-22-5-26-2-2-55-9-3-5-7-3-17-15 | MTIII-157 | - | CLIII-4 | CMGIII |
| 3415 | - | 2013 | Madagascar | AEZ7 | S47 | 1379 | -19,08 | 47,17 | DECEMBER | 21.9 | ST | - | RS3-MLVA16 | 11-3-2-11-5-24-2-2-57-9-3-5-7-3-17-15 | MTIII-161 | - | CLIII-4 | CMGIII |
| 3416 | - | 2013 | Madagascar | AEZ7 | S47 | 1379 | -19,08 | 47,17 | DECEMBER | 21.9 | ST | 19 | RS3-MLVA16 | 7-3-2-12-6-21-2-2-21-9-3-5-6-3-18-14 | MTIII-124 | - | CLIII-4 | CMGIII |
| 3421 | - | 2013 | Madagascar | AEZ7 | S47 | 1379 | -19,08 | 47,17 | DECEMBER | 21.9 | ST | 19 | RS3-MLVA16 | 10-3-2-25-5-34-2-2-55-9-3-5-6-3-16-15 | MTIII-81 | - | CLIII-4 | CMGIII |
| 3424 | - | 2013 | Madagascar | AEZ7 | S47 | 1379 | -19,08 | 47,17 | DECEMBER | 21.9 | ST | 19 | RS3-MLVA16 | 11-3-2-22-5-32-2-2-58-9-3-5-7-3-17-15 | MTIII-75 | CC MTIII-9 | CLIII-4 | CMGIII |
| 3425 | - | 2013 | Madagascar | AEZ7 | S47 | 1379 | -19,08 | 47,17 | DECEMBER | 21.9 | ST | 19 | RS3-MLVA16 | 11-3-2-22-5-32-2-2-58-9-3-5-7-3-17-15 | MTIII-75 | CC MTIII-9 | CLIII-4 | CMGIII |
| 3430 | - | 2013 | Madagascar | AEZ7 | S45 | 1390 | -19,08 | 47,17 | DECEMBER | 21.9 | PV | 19 | RS3-MLVA16 | 11-3-2-14-5-26-2-2-57-9-3-5-7-3-17-14 | MTIII-160 | - | CLIII-4 | CMGIII |
| 3436 | - | 2013 | Madagascar | AEZ7 | S47 | 1379 | -19,08 | 47,17 | DECEMBER | 21.9 | ST | 19 | RS3-MLVA16 | 10-3-2-25-5-34-2-2-55-9-3-5-6-3-16-15 | MTIII-81 | - | CLIII-4 | CMGIII |
| 3438 | - | 2013 | Madagascar | AEZ7 | S47 | 1379 | -19,08 | 47,17 | DECEMBER | 21.9 | ST | 19 | RS3-MLVA16 | 10-3-2-25-5-34-2-2-55-9-3-5-6-3-16-15 | MTIII-81 | - | CLIII-4 | CMGIII |
| 3441 | - | 2013 | Madagascar | AEZ7 | S47 | 1379 | -19,08 | 47,17 | DECEMBER | 21.9 | ST | 19 | RS3-MLVA16 | 7-3-2-10-5-27-2-2-23-9-3-5-6-3-20-10 | MTIII-139 | CC MTIII-7 | CLIII-4 | CMGIII |
| 3442 | - | 2013 | Madagascar | AEZ7 | S47 | 1379 | -19,08 | 47,17 | DECEMBER | 21.9 | ST | 19 | RS3-MLVA16 | 7-3-2-10-5-29-2-2-23-9-3-5-6-3-20-10 | MTIII-138 | CC MTIII-7 | CLIII-4 | CMGIII |
| 3444 | - | 2013 | Madagascar | AEZ7 | S47 | 1379 | -19,08 | 47,17 | DECEMBER | 21.9 | ST | 19 | RS3-MLVA16 | 11-3-2-23-5-25-2-2-56-9-3-5-7-3-21-15 | MTIII-156 | - | CLIII-4 | CMGIII |
| 3503 | - | 2013 | Madagascar | AEZ7 | S49 | 1476 | -19,07 | 47,20 | DECEMBER | 21.9 | ST | 19 | RS3-MLVA16 | 12-3-3-7-6-6-2-2-16-9-3-5-5-3-5-12 | MTIII-151 | - | CLIII-4 | CMGIII |
| 3508 | - | 2013 | Madagascar | AEZ10 | S18 | 1637 | -20,82 | 47,18 | DECEMBER | 20.3 | ST | - | RS3-MLVA16 | 7-3-2-9-4-6-2-2-23-9-3-5-6-3-3-13 | MTIII-82 | CC MTIII-1 | CLIII-5 | CMGIII |
| 3509 | - | 2013 | Madagascar | AEZ10 | S13 | 1579 | -20,83 | 47,19 | DECEMBER | 20.3 | ST | - | RS3-MLVA16 | 7-3-3-13-5-3-2-2-24-9-3-7-6-4-4-13 | MTIII-71 | CC MTIII-6 | CLIII-5 | CMGIII |
| 3514 | - | 2013 | Madagascar | AEZ10 | S20 | 1653 | -20,82 | 47,18 | DECEMBER | 20.3 | ST | - | RS3-MLVA16 | 7-3-2-9-4-6-2-2-23-9-3-5-6-3-3-13 | MTIII-82 | CC MTIII-1 | CLIII-5 | CMGIII |
| 3592 | TM-T1-CM01A | 2014 | Cameroon | - | - | - | - | - |  | - | SL | 29 | RS3-MLVA16 | 10-4-3-9-7-12-6-3-12-10-3-5-6-4-16-16 | MTIII-4 | - | - | CREF-III |
| 3593 | TM-T1-CM01B | 2014 | Cameroon | - | - | - | - | - |  | - | SL | 29 | RS3-MLVA16 | 10-4-3-9-7-12-6-3-12-10-3-5-6-4-16-16 | MTIII-4 | - | - | CREF-III |
| 3594 | TM-T1-CM02A | 2014 | Cameroon | - | - | - | - | - |  | - | SL | 29 | RS3-MLVA16 | 10-4-3-9-7-12-6-3-12-10-3-5-6-4-16-16 | MTIII-4 | - | - | CREF-III |
| 3595 | TM-T1-CM02B | 2014 | Cameroon | - | - | - | - | - |  | - | SL | 29 | RS3-MLVA16 | 10-4-3-9-7-12-6-3-12-10-3-5-6-4-16-16 | MTIII-4 | - | - | CREF-III |
| 3596 | TM-T1-CM06A | 2014 | Cameroon | - | - | - | - | - |  | - | SL | 29 | RS3-MLVA16 | 10-4-3-9-7-12-6-3-12-10-3-5-6-4-16-16 | MTIII-4 | - | - | CREF-III |
| 3597 | TM-T1-CM06B | 2014 | Cameroon | - | - | - | - | - |  | - | SL | 29 | RS3-MLVA16 | 10-4-3-9-7-12-6-3-12-10-3-5-6-4-16-16 | MTIII-4 | - | - | CREF-III |
| 3598 | TM-T1-CM11A | 2014 | Cameroon | - | - | - | - | - |  | - | SL | 29 | RS3-MLVA16 | 10-4-3-9-7-12-6-3-12-10-3-5-6-4-16-16 | MTIII-4 | - | - | CREF-III |
| 3599 | TM-T1-CM11B | 2014 | Cameroon | - | - | - | - | - |  | - | SL | 29 | RS3-MLVA16 | 10-4-3-9-7-12-6-3-12-10-3-5-6-4-16-16 | MTIII-4 | - | - | CREF-III |
| 3600 | TM-T1-CM20 | 2014 | Cameroon | - | - | - | - | - |  | - | SL | 29 | RS3-MLVA16 | 10-4-3-9-7-12-6-3-12-10-3-5-6-4-16-16 | MTIII-4 | - | - | CREF-III |
| 3601 | TM-T1-CM21 | 2014 | Cameroon | - | - | - | - | - |  | - | SL | 29 | RS3-MLVA16 | 10-4-3-9-7-12-6-3-12-10-3-5-6-4-16-16 | MTIII-4 | - | - | CREF-III |
| 3602 | TM-T2-CM06 | 2014 | Cameroon | - | - | - | - | - |  | - | SL | 29 | RS3-MLVA16 | 10-4-3-9-7-12-6-3-12-10-3-5-6-4-16-16 | MTIII-4 | - | - | CREF-III |
| 3603 | TM-T2-CM07A | 2014 | Cameroon | - | - | - | - | - |  | - | SL | 29 | RS3-MLVA16 | 10-4-3-9-7-12-6-3-12-10-3-5-6-4-16-16 | MTIII-4 | - | - | CREF-III |
| 3604 | TM-T2-CM07B | 2014 | Cameroon | - | - | - | - | - |  | - | SL | 29 | RS3-MLVA16 | 10-4-3-9-7-13-6-3-12-10-3-5-6-4-16-16 | MTIII-5 | - | - | CREF-III |
| 3605 | TM-T2-CM16 | 2014 | Cameroon | - | - | - | - | - |  | - | SL | 29 | RS3-MLVA16 | 10-3-3-9-7-12-6-3-12-10-3-5-6-4-16-16 | MTIII-47 | - | - | CREF-III |
| 3606 | TM-T2-CM17 | 2014 | Cameroon | - | - | - | - | - |  | - | SL | 29 | RS3-MLVA16 | 10-4-3-9-7-12-6-3-12-10-3-5-6-4-16-16 | MTIII-4 | - | - | CREF-III |
| 3607 | TM-T2-CM18A | 2014 | Cameroon | - | - | - | - | - |  | - | SL | 29 | RS3-MLVA16 | 10-4-3-9-7-9-6-3-12-10-3-5-6-4-16-16 | MTIII-6 | - | - | CREF-III |
| 3608 | TM-T2-CM18B | 2014 | Cameroon | - | - | - | - | - |  | - | SL | 29 | RS3-MLVA16 | 10-4-3-9-7-9-6-3-12-10-3-5-6-4-16-16 | MTIII-6 | - | - | CREF-III |
