# Supplementary material for: Molecular Epidemiology of Bacterial Wilt in the Madagascar Highlands Caused by Andean (Phylotype IIB-1) and African (Phylotype III) Brown Rot Strains of the Ralstonia solanacearum Species Complex
Source: Front Plant Sci. 2018 Jan 15;8:2258. doi: 10.3389/fpls.2017.02258 (PMC5775269; doi:10.3389/fpls.2017.02258)
Supplement: Supplementary file 3 [file Table_2.DOCX]

**Supplementary Table 2. GenBank accession numbers for *egl* sequences used in this study.** Accession numbers of sequences retrieved from GenBank are in italics. Accession numbers of new sequences are in bold.

| RUNID | SEQUEVAR | GENBANK ACCESSION NUMBER |  | RUNID | SEQUEVAR | GENBANK ACCESSION NUMBER |
| --- | --- | --- | --- | --- | --- | --- |
| RUN0001 | 1 | *EF371814* |  | RUN0981 | 25 | *GU295017* |
| RUN0461 | 2 | *GU294936* |  | RUN1340 | 25 | *GU295050* |
| RUN0628 | 2 | *GU294954* |  | RUN0482 | 26 | *EF647739* |
| RUN0894 | 2 | *GU294981* |  | RUN0299 | 27 | *EF371833* |
| RUN0074 | 3 | *EF371841* |  | RUN2221 | 27 | *KF154706* |
| RUN0265 | 3 | *GU295005* |  | RUN0079 | 28 | *AF295261* |
| RUN0459 | 3 | *GU294937* |  | RUN0896 | 28 | *GU294982* |
| RUN1366 | 3 | *GU295054* |  | RUN0053 | 29 | *EF439758* |
| RUN1980 | 4 | *KF154629* |  | RUN0133 | 29 | *JF702319* |
| RUN2227 | 4 | *KF154711* |  | RUN0137 | 29 | *GU295006* |
| RUN0009 | 6 | *EF371812* |  | RUN0143 | 29 | *EF439748* |
| RUN0097 | 6 | *DQ011546* |  | RUN0145 | 29 | *EF439749* |
| RUN0055 | 7 | *AF295263* |  | RUN0149 | 29 | *EF439752* |
| RUN0646 | 7 | *GU294993* |  | RUN0151 | 29 | *EF439753* |
| RUN1527 | 7 | *JF702315* |  | RUN0164 | 29 | *EF439759* |
| RUN0070 | 8 | *GU295042* |  | RUN0165 | 29 | *EF439763* |
| RUN0089 | 9 | *DQ011552* |  | RUN0171 | 29 | *EF439762* |
| RUN1360 | 9A | *JF702320* |  | RUN0172 | 29 | *EF439763* |
| RUN0088 | 9B | *JF702321* |  | RUN0471 | 31 | *GU295032* |
| RUN0062 | 10 | *GU295045* |  | RUN1801 | 31 | *JN798801* |
| RUN0083 | 10 | *EF371804* |  | RUN3151 | 33 | MF134836 |
| RUN0703 | 10 | *GU294996* |  | RUN0257 | 34 | *FJ561167* |
| RUN0014 | 11 | *GQ907150* |  | RUN0030 | 35 | *AF295264* |
| RUN1361 | 11 | *JF702313* |  | RUN0169 | 35 | *EF439761* |
| RUN0090 | 12 | *GQ907153* |  | RUN0189 | 35 | *EF439730* |
| RUN0091 | 12 | *AF295255* |  | RUN0027 | 36 | *AF295265* |
| RUN0183 | 13 | *EF439768* |  | RUN0549 | 37 | *JF702309* |
| RUN0215 | 13 | *EF439740* |  | RUN0042 | 38 | *GQ907152* |
| RUN0608 | 13 | *AF295252* |  | RUN0279 | 38 | *EF371836* |
| RUN1798 | 13 | *JN798800* |  | RUN2210 | 38 | *KF154696* |
| RUN0258 | 14 | *FJ561066* |  | RUN0028 | 39 | *AF295266* |
| RUN1528 | 14 | *JF702316* |  | RUN0058 | 39 | *EF371806* |
| RUN1919 | 14 | *JN798794* |  | RUN2187 | 39 | *KF154674* |
| RUN2241 | 14 | *KF154722* |  | RUN0043 | 40 | *AF295269* |
| RUN0069 | 15 | *AF295250* |  | RUN0109 | 40 | *DQ657612* |
| RUN0085 | 15 | *EU407285* |  | RUN0150 | 41 | *EF439726* |
| RUN0155 | 15 | *EU407299* |  | RUN0364 | 42 | *GU295011* |
| RUN0044 | 16 | *AF295254* |  | RUN0364 | 42 | *GU295011* |
| RUN0337 | 17 | *FJ561068* |  | RUN0362 | 43 | *GU295009* |
| RUN1948 | 17 | *KF154597* |  | RUN0369 | 43 | *GU295008* |
| RUN2231 | 17 | *KF154714* |  | RUN0135 | 44 | *EF439744* |
| RUN0054 | 18 | *AF295251* |  | RUN1833 | 44 | *JN798833* |
| RUN0466 | 18 | *EU726820* |  | RUN0047 | 45 | *GQ907151* |
| RUN0060 | 19 | *KU255920* |  | RUN0320 | 46 | *GU295040* |
| RUN0332 | 19 | *GU295041* |  | RUN1880 | 46 | *JN798762* |
| RUN0477 | 19 | *KU255934* |  | RUN2177 | 47 | *KF154665* |
| RUN0056 | 20 | *AF295279* |  | RUN0343 | 48 | *FJ561067* |
| RUN0076 | 20 | *JF702305* |  | RUN1793 | 48 | *JN798797* |
| RUN0146 | 20 | *GU295007* |  | RUN1794 | 48 | *JN798798* |
| RUN0098 | 21 | *JF702306* |  | RUN1796 | 48 | *JN798799* |
| RUN0479 | 21 | *AF295271* |  | RUN0166 | 49 | *EF439729* |
| RUN0075 | 22 | *AF295276* |  | RUN0448 | 50 | *GU295049* |
| RUN1298 | 22 | *AF295276* |  | RUN0297 | 51 | *AF371831* |
| RUN0039 | 23 | *AF295270* |  | RUN0203 | 52 | *EF439725* |
| RUN0022 | 24 | *GQ907154* |  | **RUN2340** | **58** | **MF134833** |
| RUN0301 | 24 | *EF371839* |  | **RUN2280** | **59** | **MF134834** |
| RUN0110 | 25 | *AF295260* |  | **RUN2987** | **60** | **MF134835** |
