# Supplementary material for: Molecular Epidemiology of Bacterial Wilt in the Madagascar Highlands Caused by Andean (Phylotype IIB-1) and African (Phylotype III) Brown Rot Strains of the Ralstonia solanacearum Species Complex
Source: Front Plant Sci. 2018 Jan 15;8:2258. doi: 10.3389/fpls.2017.02258 (PMC5775269; doi:10.3389/fpls.2017.02258)
Supplement: Supplementary file 4 [file Table_3.DOCX]

**Supplementary Table 3. Description of TR markers, corresponding oligonucleotide primers and multiplex combinations, and PCR conditions used in this study. (A)** Nomenclature of TR locus was as follows: marker alias_replicon of origin (ch: chromosome or mp: megaplasmid)_start physical position expressed in kilobases in the genome of strain origin_TR unit size (bp)_amplicon size in the genome of strain origin (bp)_number of repeats (units) (e.g., RS1L12_1774_6bp_196bp_12u) (Le Fleche et al., 2002; N’Guessan et al., 2013). Marker alias is RS: R. *solanacearum*; followed by phylotype number of origin: 1, 2, 3, or 4; and reference of locus (e.g., RS3L12). **(B)** All positions are in the genome of strain origin. Accession numbers: CMR15 chromosome (FP885895), CMR15 megaplasmid (FP885896), Molk2 genome (GCF_000212635.3), UW551 genome (GCF_000167955.1), IPO1609 genome (GCF_001050995.1), CFBP2957 genome (FP885897.1). **(C)** Fluorescent dye labelled at 5′-end forward primer.

| Official nomenclature^(A)^ | Locus genomic position (strain)^(B)^ | Location (coding sequence)^(B)^ | Tandem repeat sequence^(B)^ | Oligonucleotide primer (5' to 3') ^(C)^ | Multiplex prrimer mix | Primer concentration (mM) | Note and Reference |
| --- | --- | --- | --- | --- | --- | --- | --- |
|  |  |  |  |  |  |  |  |
| **RS3-MLVA16** | | | | | | | |
| ***Chromosome*** |  |  |  |  |  |  |  |
| RS3L27_ch622_8bp_240bp_10u | 622140--622218 (CMR15) | intergenic | CGGCTCAG | F: **VIC-**GATCCGGCCCTCATCAAGCG | mix1 | 2 | this study |
|  |  |  |  | R: CACCCGTGAGCTGATCGACC |  | 2 |  |
| RS3L28_ch1093_11bp_273bp_4u | 1093103--1093136 (CMR15) | intergenic | GGCCCCGATTT | F: **VIC-**GGATTCGCCCTAGGTCTGCC | mix4 | 2 | this study |
|  |  |  |  | R: ATGTCGATGATCTGCGGGGC |  | 2 |  |
| RS3L29_ch1169_9bp_150bp_3u | 1169503--1169531 (CMR15) | CDS (putative hemagglutinin-related autotransporter protein) | CAGCCCGAT | F: **PET-**CGAAATCCGCAGCCTCGCC | mix3 | 2 | this study |
|  |  |  |  | R: GCCGACAACAGGGGCGAAGA |  | 2 |  |
| RS3L17_ch1269_6bp_299bp_9u | 1269368--1269423 (CMR15) | intergenic | CGCAGC | F: **PET-**CGCTGCACATCGCCTCCTGCACCTCG | mix1 | 2 | CMR026 (N'Guessan et al., 2013) |
|  |  |  |  | R: TGCCGGGAAGTTGCTGCGCCCGTT |  | 2 |  |
| RS3L19_ch2111_9bp_245bp_7u | 2111048--2111107 (CMR15) | CDS (protein of unknown function) | TGCTTGAAG | F: **VIC-**GCTCGCTCTCGCCTGTTCCGCCC | mix2 | 2 | CMR2699 (N'Guessan et al., 2013) |
|  |  |  |  | R: ACATCCAGGTCCGCACGCCCGC |  | 2 |  |
| RS3L30_ch2610_5bp_115bp_12u | 2610379--2610437 (CMR15) | CDS ( putative F pilus assembly protein TraF) | GCCGA | F: **6FAM-**ATCGGAATCTTTGTGGCGAT | mix1 | 4 | this study |
|  |  |  |  | R: GCCAAGCACCACCACC |  | 4 |  |
| RS3L31_ch2618_6bp_13bp_6u | 2618421--2618454 (CMR15) | intergenic | CTTGAC | F: **6FAM-**AGCAAAACAAGAGCAATACCAGCC | mix3 | 2 | this study |
|  |  |  |  | R: TCGTGTTCAGGTCGCTATCCA |  | 2 |  |
| RS3L32_ch2852_10bp_292bp_3u | 2852333--2852362 (CMR15) | intergenic | ACGCACACGC | F: **NED**-GTTTCAGCAACGACTACAAGCAG | mix3 | 4 | this study |
|  |  |  |  | R: AGGCTTCGATTCCCTTCTGATG |  | 4 |  |
|  |  |  |  |  |  |  |  |
| ***Megaplasmid*** |  |  |  |  |  |  |  |
| RS3L33_mp131_6bp_128bp_12u | 131842--131911 (CMR15) | intergenic | TGCGGA | F: **6FAM-**GTGCCTGCCCGATCTCAACG | mix2 | 4 | this study |
|  |  |  |  | R: GGTGGGCCGTGTGACCATTG |  | 4 |  |
| RS3L34_mp233_6bp_276bp_9u | 233468--233517 (CMR15) | CDS (putative secreted protein popf1) | GCCGGA | F: **NED**-CGTTCCAGGTCACGTCGCTG | mix2 | 2 | this study |
|  |  |  |  | R: CGGGTTCGCTGTTCTTCCA |  | 2 |  |
| RS1L05_mp444_18bp_194bp_3u | 444568--444620 (CMR15) | CDS (Conserved exported protein of unknown function; putative lipoprotein) | GCGTACCCGATTCTGCGG | F: **NED**-GCGCGGCATCGTGCTGTGGCGATT | mix4 | 2 | ch127 (N'Guessan et al., 2013) |
|  |  |  |  | R: GCTGGCGGGTCGGTTCGAGCGT |  | 2 |  |
| RS3L35_mp741_9bp_275bp_5u | 741526--741568 (CMR15) | CDS (flgD) | CACGACCAG | F: **VIC-**GTCGCGCTCGTACCAGGCC | mix3 | 4 | this study |
|  |  |  |  | R: AGCTGCGAGTTGTCCATCGGG |  | 4 |  |
| RS3L36_mp922_12bp_355bp_6u | 922063--922134 (CMR15) | CDS (conserved membrane protein of unknown function, putative DnaJ-class chaperone) | CAGCAGGACGAG | F: **PET-**CGTGGGCGGTGCTGAACATC | mix4 | 4 | this study |
|  |  |  |  | R: CGTGGGGAGTAGTCGGGCGA |  | 4 |  |
| RS1L10_mp1076_9bp_375bp_4u | 1076440--1076464 (CMR15) | CDS (atpH:ATP synthase, F1 sector, delta subunit) | GGCGATGAG | F: **6FAM-**CGGCGGTTGAGACCGGGGAGAGGG | mix4 | 2 | ch827 (N'Guessan et al., 2013) |
|  |  |  |  | R: GCTGCGCCAGGGCTACGAGGCG |  | 2 |  |
| RS3L37_mp1438_6bp_211bp_15u | 1438487--1438533 (CMR15) | intergenic | CTGGCA | F: **NED**-CTCAACGCCCACGAAACC | mix1 | 4 | this study |
|  |  |  |  | R: ATTGTTCAGGGGTCGATGC |  | 4 |  |
| RS1L12_mp1774_6bp_196bp_12u | 1774159--1774225 (CMR15) | intergenic | GCGGCT | F: **PET-**GGACCGGGGACGCATGGAAACGAACGA | mix2 | 2 | ch1459 (N'Guessan et al., 2013) |
|  |  |  |  | R: ATGGGGGACCCGCTTCCAAGCTACTGC |  | 2 |  |
|  |  |  |  |  |  |  |  |
| **RS2-MLVA9 SCHEME** | | | | | | | |
| ***Chromosome*** |  |  |  |  |  |  |  |
| RS2BL25_ch5321_11bp_218bp_5u | 5321171--5321226 (Molk2) | intergene | GGCCGGCTGAG | F: **VIC-**CGGTGCGGATGTGTTGGTGGACGGCT | mix5 | 2 | (N'Guessan et al. 2013) |
|  |  |  |  | R: CCTGATCCTGGTCGAGGGGCGCGATG |  | 2 |  |
| L539_ch424_15bp_561bp_5u | 424668--424742 (UW551) | CDS (Ribosomal large subunit pseudouridine synthase B) | TTGCTGCCCTGCGCA | F: **PET-**GCGCAACGCCCATATCATTCGC | mix6 | 2 | (Parkinson et al. 2013) |
|  |  |  |  | R: CGAGGCACAGCCGATGCTGATG |  | 2 |  |
| RS2BL22_ch4134_6bp_282bp_7u | 4134101--4134138 (IPO1609) | CDS (Luminbactin biosynthesis. Probable bifunctional enzyme salicyl-AMP ligase and salicylate synthetase) | GTAGCC | F: **NED**-TGCGCGAGGCGAGACCCAGCG | mix6 | 2 | (N'Guessan et al. 2013) |
|  |  |  |  | R: GCCTTCGTGCGGTGGCAAGGCGG |  | 2 |  |
| L540_ch1275_8bp_550bp_13u | 1275591--1275689(UW551) | intergene | GGTGAGTC | F: **NED**-GTTGTCCATGGCAGTCCTGGTGAGATC | mix5 | 2 | (Parkinson et al. 2013) |
|  |  |  |  | R: ACCATCGACCTGGAGCACGATC |  | 2 |  |
| RS2BL21_ch100_7bp_193bp_11u | 100720--100796 (IPO1609) | intergene | GTAGCC | F: **NED**-CGGCATGGAGGGTCGGGCTTGAGGTG | mix7 | 2 | (N'Guessan et al. 2013) |
|  |  |  |  | R: GGCACCTGGCCGGAAGAGAGAGAGCGT |  | 2 |  |
| RS2AL01_ch386_9bp_305bp_7u | 386992--387049 (CFBP2957) | CDS (conserved exported protein of unknown function) | GGCCGCATT | F: **PET-**CGGCGTGCCACTGGGCGACGTAGA | mix5 | 2 | (N'Guessan et al. 2013) |
|  |  |  |  | R: CCGCTTGACCCCATCCGCCTTCCTG |  | 2 |  |
| RS2BL24_ch1936_6bp_374bp_10u | 1936756--1936813 (Molk2) | CDS (putative peptidase, M50 family) | AACATG | F: **6FAM-**CGGGCGAAGGCTCGCAGGCCAA | mix5 | 2 | (N'Guessan et al. 2013) |
|  |  |  |  | R: GGCCGGACGATACATGCCACCGCTCAC |  | 2 |  |
| L563_ch3300_7bp_408bp_8u | 3300703--3300759 (UW551) | intergene | CCTCTAG | F : **VIC-**ACGTTCGCGTAGGCGTGGACAAG | mix7 | 2 | (Parkinson et al. 2013) |
|  |  |  |  | R: TACACGGCGGCGATGTGCTGAC |  | 2 |  |
| L504_ch3773_7bp_502bp_9u | 3773859--3773920 (UW551) | CDS (putative 6-phosphofructokinase - fragment) | CAAGTGG | F: **6FAM-**GGCCGACCAGCGCTCCACAAG | mix7 | 2 | (Parkinson et al. 2013) |
|  |  |  |  | R: CGGACGGTTTGATGGTCATCAACATGA |  | 2 |  |
